# A gold nanoparticle/peptide vaccine designed to induce SARS-CoV-2-specific CD8 T cells: a double-blind, randomized, phase 1 study in Switzerland

**DOI:** 10.1186/s12879-025-10844-3

**Published:** 2025-04-07

**Authors:** Juliette Besson, Régine Audran, Maxime Karlen, Alix Miauton, Hélène Maby-El Hajjami, Loane Warpelin-Decrausaz, Loredana Sene, Sylvain Schaufelberger, Vincent Faivre, Mohamed Faouzi, Mary-Anne Hartley, François Spertini, Blaise Genton

**Affiliations:** 1 Tropical, Travel and Vaccination Clinic, Center for Primary Care and Public Health (Unisanté), University of Lausanne, Lausanne, Switzerland; 2 Division of Immunology and Allergy, Centre Hospitalier Universitaire Vaudois (CHUV), University of Lausanne, Lausanne, Switzerland; 3 Clinical Trial Unit, Centre Hospitalier Universitaire Vaudois (CHUV), University of Lausanne, Lausanne, Switzerland; 4 Research Support Unit, Center for Primary Care and Public Health (Unisanté), University of Lausanne, Lausanne, Switzerland; 5 Information Systems and Digital Transformation, Center for Primary Care and Public Health (Unisanté), University of Lausanne, Lausanne, Switzerland; 6 Division of Biostatistics, Center for Primary Care and Public Health (Unisanté), University of Lausanne, Lausanne, Switzerland

**Keywords:** COVID-19, SARS-CoV-2, Vaccine, Nanoparticle, Cellular, Immunity

## Abstract

**Background:**

New vaccines with broader protection against SARS-CoV-2 are needed to reduce the risk of immune escape and provide broad and long-lasting cellular immunity. The objectives of the naNO-COVID trial were to evaluate the safety and immunogenicity of a CD8 + T cell, gold nanoparticle-based, peptide COVID-19 vaccine.

**Methods:**

A randomized, double-blind, vehicle-controlled, phase 1 trial in healthy adults to receive PepGNP-Covid19 or Vehicle-GNP, followed over 180 days, using a dose-escalation strategy.

**Results:**

Twenty participants received PepGNP-Covid19 (low dose [LD] or high dose [HD], *n* = 10 each) and six Vehicle-GNP (LD or HD, *n* = 3 each).

Vaccinations were safe. No serious adverse events were reported. Most of the adverse events were mild, two adverse events of special interest related to the product (fever and fatigue). Reactogenicity was similar overall between vaccine, comparator, and doses.

Virus-specific humoral responses in LD PepGNP-Covid19 and Vehicle-GNP groups coincided with SARS-CoV-2 infections. PepGNP-Covid19 vaccination induced the modulation of Covid19-specific CD137 + CD69 + CD8 + , and an increase at day 35 particularly in central and effector memory T cells in LD group, and in late effector memory cells in HD group.

**Conclusions:**

The favourable safety profile and cellular responses observed support further development of PepGNP-Covid19.

**Trial registration:**

ClinicalTrials.gov, NCT05113862, approved 09.11.2021.

**Supplementary Information:**

The online version contains supplementary material available at 10.1186/s12879-025-10844-3.

## Introduction

The Coronavirus disease (COVID-19) pandemic led to considerable morbidity and mortality. Development of vaccines has been very fast in response to the need of protection against severe disease and prevention of transmission. Many vaccines are now effective and available. However, the World Health Organization still recommends developing new vaccine products with durable, broader protection and improved access in order to reduce virus transmission and thus the risk of immune escape with new variants of concern [1]. At the time of writing this article, the available mRNA vaccines still fail to induce long-term immunity due to the lack of induction of long-lived plasma cells [2].

The pathophysiology of COVID-19 and its complex process leading to acute respiratory distress syndrome have now been well assessed [3]. Concerns were initially raised that the activity of T cells could contribute to inflammation in the lungs and hence severe disease [4]. In fact, many studies demonstrated better outcomes related to T cell adaptive responses [5]. In the airways of severe COVID-19 patients, higher frequencies of T cells correlated with survival [6], which justifies the development of vaccines inducing strong T cell responses. Moreover, T cells are generally directed at epitopes from conserved viral regions and T cells memory is established to be long-lasting [7], which are needed properties of anti-viral immunity according to the epidemiology of SARS-CoV-2.

To specifically induce CD8 + responses, the candidate vaccine PepGNP-Covid19 was developed, composed of synthetic class I major histocompatibility complex (MHC-I)-selective SARS-CoV-2 peptides anchored on self-adjuvanted gold nanoparticles (GNPs) that serve as a vehicle for peptide delivery. The safety and immunogenicity of PepGNP-Covid19 have been demonstrated in preclinical in vitro and in vivo studies. The same vaccine platform used in a phase 1 trial against dengue (naNO-DENGUE) gave favourable results [8].

This new vaccine concept has multiple advantages: not only the induction of cellular immunity that is known to be more effective against severe disease and long-lasting compared to the humoral immunity, but such a vaccine could have more potential for sustainability compared to the currently available ones. Indeed, the vaccine is designed to induce broader protection, with the selected peptides being less prone to mutations, being mainly selected from the nucleocapsid (seven peptides) rather than the spike proteins (one peptide). Importantly, the platform could also be rapidly adapted as needed to newly emerging threats.

**Fig. 1:**
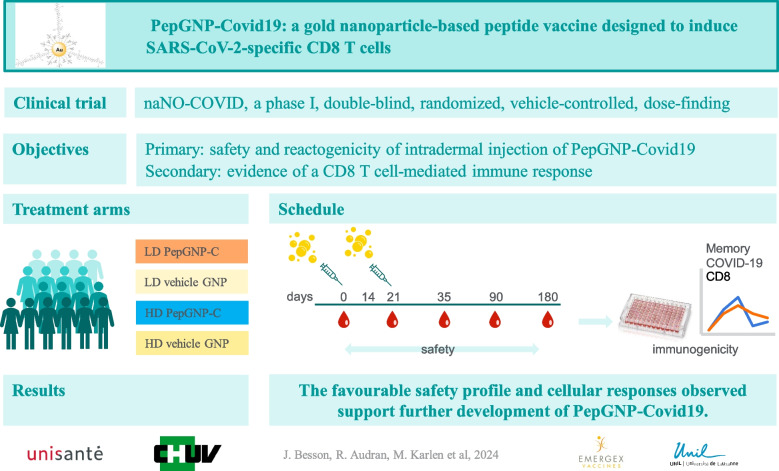
Graphical abstract. Overview of the naNO-COVID trial

The naNO-COVID trial aimed to evaluate the safety and reactogenicity of two different doses of PepGNP-Covid19 administered for the first time to healthy participants. A secondary objective was to assess its immunogenicity, by evaluating the CD8 + T cell mediated immune response as a surrogate of protection against severe COVID-19 (Fig. 1).

## Material and methods

### Study design and participants

naNO-COVID was a single-center, vehicle-controlled, randomized, double-blind, dose-finding phase 1 trial to assess the safety and immunogenicity of PepGNP-Covid19 (low-dose [LD] and high-dose [HD]). The study took place at the Center for Primary Care and Public Health and the Clinical Trial Unit of the University Hospital, Lausanne, Switzerland, during the COVID-19 Omicron variant wave. Participants were recruited through advertisements posted in the local academic and hospital communication networks. Eligible participants were healthy adults aged 18–45 years. Exclusion criteria included, notably, another COVID-19 vaccination within four weeks before and after the trial vaccinations, or a documented COVID-19 within four weeks of the first trial vaccination (appendix A). If there was a suspicion of COVID-19 on the days of vaccination, the injection was delayed until a negative result was confirmed by PCR and clinical manifestations resolved. All participants provided written informed consent. The questionnaires for the interviews were developed for this study specifically and the codebook is available online 10.16909/dataset/54).

### Randomization, masking and safety controls

Eligible participants were enrolled and randomly assigned by computer-generated stratified randomization lists to receive either PepGNP-Covid19 or Vehicle-GNP the day of the first vaccination. Vehicle-GNP was chosen as control to better isolate the effect of the virus-specific peptides from that of the GNPs. Only the pharmacists responsible for vaccine reconstitution had access to the allocation list. The investigators, sponsor and participants were masked to group assignment. The naNO-COVID trial was initiated upon the favourable outcome of an interim analysis, conducted by an independent data safety and monitoring committee (DSMC), of safety data from the naNO-DENGUE trial of a similar vaccine construct [8]. Since this was a first-in-human trial, a risk-minimizing strategy was used; one participant was vaccinated per day for the first three ‘pioneer’ participants in each dosage group (block of three, 2:1, intervention:comparator). After a first safety review of the three LD ‘pioneers’, ten LD ‘followers’ were randomized by block of five (8:2, intervention:comparator) and vaccinated. The DSMC performed a planned safety analysis prior to the second injection and prior to dose escalation. The same process was applied for the HD group.

### Candidate vaccine

SARS-CoV-2 peptides were selected as indicated in appendix B (sequence in table A1). The base particle carrier, α-galactose and N-acetylglucosamine-passivated GNP core of approximately 1.6 nm in diameter were manufactured according to current good manufacturing practice by Ardena-OSS (Netherlands). Peptides were attached via a thiolated tripeptide by ligand exchange to the GNP. The final drug products (PepGNP-Covid19 and Vehicle-GNP) were lyophilized and packed by Symbiosis Pharmaceutical Services Limited (UK) and stored at −20 °C. Pharmacists at the Lausanne University Hospital reconstituted the freeze-dried powder products with water for injection.

The LD formulations contained GNP with a gold content of 12.8 µg, alone (LD-Vehicle-GNP) or with 2.5 nmol of SARS-CoV-2 peptides (LD-PepGNP-Covid19); the HD formulations contained GNP with a gold content of 38.3 µg, alone (HD-Vehicle-GNP) or with 7.5 nmol of SARS-CoV-2 peptides (HD-PepGNP-Covid19). Dosages were selected based on preclinical testing.

### Procedures

Two doses (50 µL each) were administered intradermally 21 days apart (D0 and D21), in the deltoid region using Nanopass MicronJet600 microneedles.

Participants were followed at regular intervals for up to six months after the first injection (D1, D7, D14, D22, D28, D35, D60, D90 and D180). Visits at D1, D22 and D60 were performed remotely (by phone), while others consisted of on-site interviews with blood sample collections.

Reactogenicity and safety were assessed one hour post-injection and at above-mentioned time points. Solicited local and systemic reactions occurring up to 7 and 14 days after each injection, respectively, were recorded by participants in diary cards. Safety laboratory analyses were performed at D7 and D14 after each injection. Unsolicited AEs were recorded during the entire study period. The investigators assessed all AEs in terms of timing, severity, seriousness and relatedness with the study product according to predefined scales and guidelines (appendix C).

Immunological analyses were performed on D0, D21, D35, D90 and D180 (detailed in appendix D). Humoral immune responses against SARS-CoV-2 spike and nucleoprotein IgG were determined by Luminex. CD8 + T cell responses induced by PepGNP-Covid19 were determined by cytometry: i) by measurement of activation-induced markers (AIM) at baseline and at D21, D35, D90 and D180 and ii) using HLA class I dextramers at baseline, at D35 and D180.

### Outcomes

Primary outcomes were frequency and severity of solicited local and systemic adverse events (AEs), 7 and 14 days after each vaccination respectively; occurrence of unsolicited AEs, adverse events of special interest (AESIs) and serious adverse events (SAEs) during the entire trial period; and change from baseline for safety laboratory measures.

Secondary outcomes were the frequency of circulating CD8 + T cells specific to PepGNP-Covid19 peptides at baseline, D21, D35, D90 and D180 after the first vaccination, and titres of anti-SARS-CoV-2 nucleoprotein and spike antibodies. Exploratory outcomes were the frequency of specific CD8 T cell subsets, CXCR3 + , naïve and memory: stem cell memory (Tscm), central memory (Tcm), effector memory (Tem) and, late differentiated effector memory (TemRA).

### Statistics

Since this was a first-in-human study, the number of participants needed to be limited and sample size was computed to detect AEs with a high incidence rate. Having ten participants per group exposed to PepGNP-Covid19 allowed 80% power of detecting an AE with a true incidence of 7.5% across all exposed participants (LD and HD combined) or 20% within a single dose group. Analysis was by intention-to-treat (ITT) for safety and both ITT and per protocol (PP) for immunogenicity.

AEs are described with absolute and relative frequencies (%) according to the group to which participants were randomized. Mean, standard deviation, minimum, maximum, median and quartiles were used for continuous safety laboratory variables. Safety analyses are descriptive only; because of the small sample size, no formal statistical hypotheses were tested.

Median and 95% confidence interval (CI) are presented for immunological analyses, along with non-parametric intra- and inter-group comparisons.

Demographic, clinical and safety data were collected with REDCap Electronic Data Capture Tool (RRID:SCR_003445) hosted at Unisanté and analysed with STATA 16 (RRID:SCR_012763) and Excel (RRID:SCR_016137). Immunogenicity data were analysed with GraphPad Prism v9.1.0 (RRID:SCR_002798).

## Results

### Safety

**Fig. 2:**
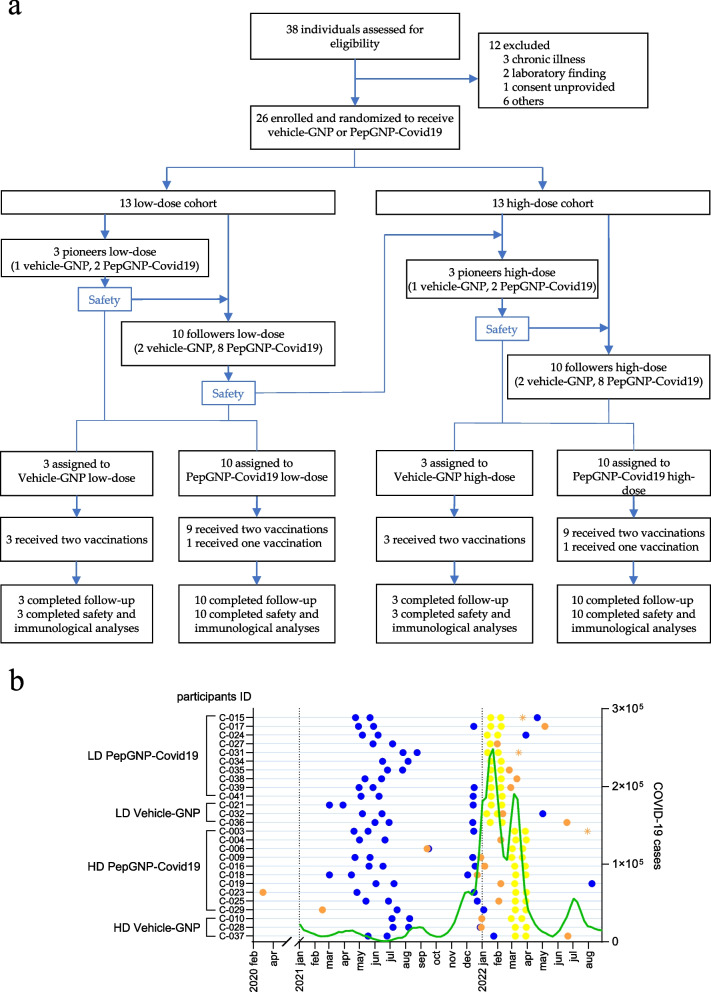
naNO-COVID trial profile and COVID-19 epidemiological context. **a** Participants were enrolled according to a dose-escalation protocol and randomly assigned within each dose group to receive two doses of PepGNP-Covid19 (gold nanoparticles and SARS-CoV-2 peptides) or Vehicle-GNP (gold nanoparticles only). Two participants did not receive the second vaccination: one HD PepGNP-Covid19-recipient due to an allergic reaction (rash, pruritus and CRP elevation) after the first injection and one LD PepGNP-Covid19-recipient due to confirmed COVID-19 preceding the second vaccination. All participants completed 180 days of follow-up. GNP = gold nanoparticle. **b** Time of events: time of first COVID-19 vaccination (if any) in blue; time of first COVID-19 (if any) in orange, orange asterisks refer to 3 probable COVID-19 infections (cases of flu-like symptoms followed by a raise in anti-N IgG); time of first and second PepGNP-Covid19 injections in yellow; the green line represents the number of weekly COVID-19 cases in Switzerland

From 38 individuals screened, 26 participants were enrolled from 10th January to 30th March 2022 to receive two doses of either PepGNP-Covid19 LD (*n* = 10), Vehicle-GNP LD (*n* = 3), PepGNP-Covid19 HD (*n* = 10) or Vehicle-GNP HD (*n* = 3) (Fig. 2a). All subjects were Caucasian, 19 [73%] were males, and mean age was 27 (range 20–39) years (Table 1). Almost all (25/26 [96%]) participants had been vaccinated with another COVID-19 vaccine (at least 2 doses) before the trial vaccinations (Fig. 2b). The HD group had a significantly higher proportion of documented COVID-19 before the first trial vaccination (11/13 [85%] overall, including 8/13 [62%] within three months) compared to the LD group (no cases) owing to timings of the trial conduct during the epidemic wave. Two participants did not receive the second vaccination due to exclusion criteria: one HD PepGNP-Covid19-recipient due to a moderate allergic reaction after the first injection (rash, pruritus and C-reactive protein [CRP] elevation) and one LD PepGNP-Covid19-recipient due to a confirmed COVID-19 seven days before the planned second injection. Both remained in the study. All participants completed the 180-days study follow-up.

**Table 1:** Summary of demographic and baseline characteristics by treatment group

	**LD vehicle-GNP (*n* = 3)**	**LD PepGNP-Covid19 (*n* = 10)**	**HD vehicle-GNP (*n* = 3)**	**HD PepGNP-Covid19 (*n* = 10)**	**All participants (*n* = 26)**
Sex					
Female	1 (33%)	2 (20%)	1 (33%)	3 (30%)	7 (27%)
Male	2 (67%)	8 (80%)	2 (67%)	7 (70%)	19 (73%)
Ethnicity					
Caucasian	3 (100%)	10 (100%)	3 (100%)	10 (100%)	26 (100%)
Other	0 (0%)	0 (0%)	0 (0%)	0 (0%)	0 (0%)
Age, years					
Mean	30.3 (4)	27.7 (7)	27.7 (9)	26 (3.8)	27.4 (5.7)
Range	28–35	21–32	22–38	21–32	20–39
BMI, kg/m^2^					
Mean	22.9 (1.4)	22.8 (3.1)	22.2 (2.2)	23.5 (3)	23 (2.7)
Range	21.7–24.4	18.9–28.7	20.2–24.5	18.9–29	18.9–29
Current smokers	1 (33%)	0 (0%)	1 (33%)	0 (0%)	2 (7.7%)
COVID-19 before D0	0 (0%)	0 (0%)	2 (67%)	9 (90%)	11 (42%)

Data are n (%) or mean (SD), unless stated otherwise

*BMI* Body-mass index, *LD* Low-dose, *HD* High-dose, *GNP* Gold nanoparticles

Overall, vaccinations were safe and well tolerated. No SAEs were recorded (table A4). Two severe (Grade 3) AEs (considered to be AESIs) of short duration were related to the product. Overall reactogenicity was similar between PepGNP-Covid19 and Vehicle-GNP groups and across the two dosages tested. All participants experienced at least one related AE including at least one unsolicited AE. Most (88%) of the related AEs were mild (Grade 1).

#### Local AEs

**Fig. 3:**
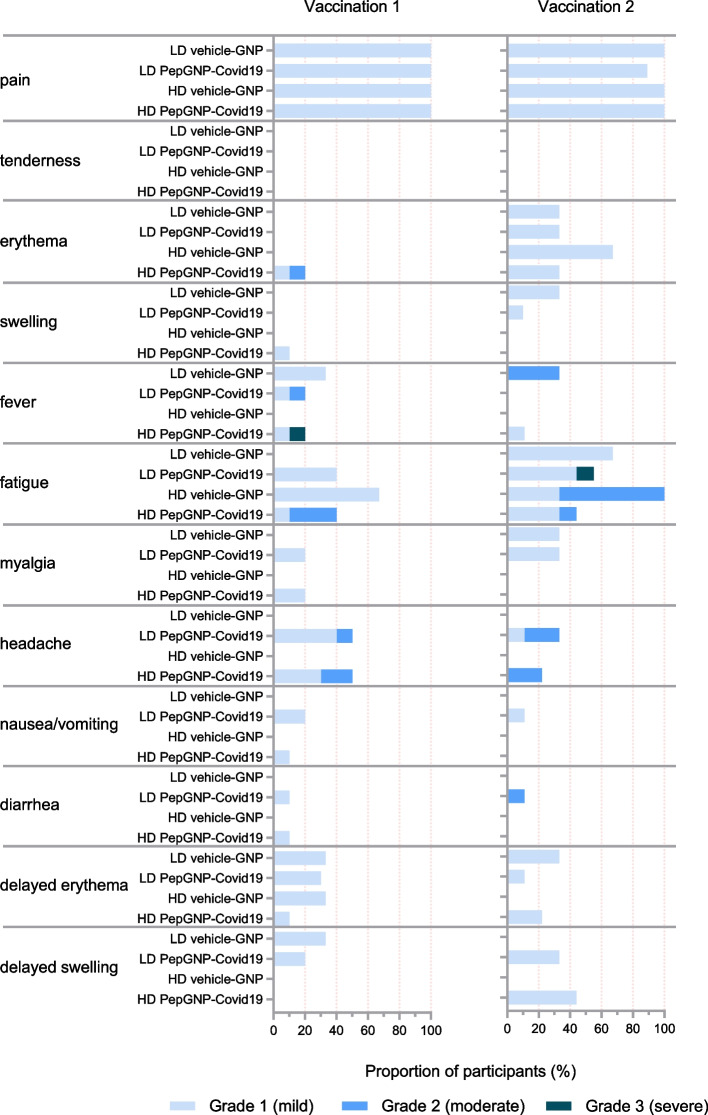
Local and systemic solicited reactogenicity* and delayed erythema and swelling. *n* = 3 in the LD and HD vehicle-GNP groups. *n* = 10 in the LD and HD PepGNP-Covid19 groups, except for the second vaccination in which *n* = 9. *Solicited local and systemic reactogenicity: signs/symptoms occurring within 7 and 14 days post-vaccination, respectively. GNP = gold nanoparticles; LD = low-dose; HD = high-dose

No severe (Grade 3) local AEs were recorded during the trial (Fig. 3). In the HD-PepGNP-Covid19 group, one participant experienced a moderate (Grade 2) solicited local AE, an early injection site erythema (within 7 days). All other solicited and all unsolicited local AEs were mild (Grade 1).

The most common unsolicited related local AE was a discoloration at the injection site observed in 22 out of 26 participants (85%). Indeed, gold nanoparticles give a brownish colour to the vaccine product that induced a transient slight greyish skin discoloration approximately 1 cm in diameter. The median duration of the discoloration was one day and then completely resolved. Early injection site erythema was observed in 38% (10/26) of participants (1/3 [33%] of LD-Vehicle-GNP, 3/10 [30%] of LD-PepGNP-Covid19, 2/3 [67%] of HD-Vehicle-GNP and 4/10 [40%] of HD-PepGNP-Covid19). Early swelling occurred in 12% (3/26) of participants (1/3 [33%] of LD-Vehicle-GNP, 1/10 [10%] of LD-PepGNP-Covid19 and 1/10 [10%] of HD-PepGNP-Covid19).

A notable number of participants experienced a delayed injection site erythema (11/26 [42%]) and/or swelling (11/26 [42%]), occurring after the 7-day post-vaccination follow-up, and sometimes even several weeks after. These events were mostly mild (Grade 1) and were reported both in the Vehicle-GNP and in the PepGNP-Covid19 groups (3/6 [50%] and 8/20 [40%] respectively), with a higher rate in the LD group (7/13 [54%] vs 4/13 [31%] in the HD group). The mean duration was extensive, 111 days in total, and some were still ongoing at the end of the trial (8/26 [31%] with erythema and 10/26 [38%] with swelling), but all improving or stable. The occurrence of these delayed local reactions slightly increased with the second dose (6/11 [55%] vs 5/11 [45%] after the first dose).

#### Systemic AEs

Four cases of severe (Grade 3) solicited AEs were reported, corresponding to AESIs. Only two were considered study product-related (Fig. 3): one case of fever > 38.5 °C (38.8 °C) observed in a HD-PepGNP-Covid19 participant after the first injection that resolved within one day, and one case of fatigue observed in a LD-PepGNP-Covid19 participant after the second injection that resolved after five days. The two unrelated AESIs consisted of severe back pain complicating an abnormal movement at D144 after vaccination in a LD-PepGNP-Covid19 candidate and a vaso-vagal reaction during a blood draw, before the second dose was administered, at D22 in a HD-PepGNP-Covid19 candidate.

Moderate (Grade 2) solicited systemic AEs were more common in the HD (9/13 [69%]) than in the LD group (5/13 [38%]), with a similar frequency between the PepGNP-Covid19 (11/20 [55%]) and Vehicle-GNP groups (3/6 [50%]). These events included seven cases of fatigue, four headaches, two fevers and one diarrhea. There were three cases of moderate (Grade 2) related unsolicited systemic AEs that all occurred in the PepGNP-Covid19 groups (one abdominal pain, one rhinitis and one urticaria).

The vast majority of solicited systemic AEs were mild (Grade 1). More participants in the PepGNP-Covid19 groups had at least one solicited systemic AE compared with the Vehicle-GNP group (17/20 [85%] vs 4/6 [67%]) but there was no difference between dosage groups. The most common solicited systemic AEs were fatigue followed by headache and myalgia. Fever developed in 27% of participants after injection, slightly more frequently in Vehicle-GNP participants (2/6 [33%]) compared to PepGNP-Covid19 participants (5/20 [25%]).

The most common unsolicited related systemic AEs were lymphopenia (7/26 [27%]) and blood creatinine level increase (6/26 [23%]). Lymphopenia was observed only in PepGNP-Covid19 participants (4/10 [40%] of the LD group and 3/10 [30%] of the HD group). Blood creatinine level increase occurred similarly in all groups (PepGNP-Covid19 5/20 [25%], Vehicle-GNP 1/6 [17%], LD and HD 3/13 [23%] in each group). All abnormal values that were considered as AEs related to the study intervention were mild (Grade 1). There were one case of anaemia and one case of alanine aminotransferase increase reported in LD-PepGNP-Covid19 participants.

Regarding COVID-19, 11/26 (42%) participants, all in the HD group, had documented COVID-19 prior to the trial vaccinations and did not show any clinical symptoms or signs of reinfection during the time of vaccination and follow-up, although a rise in anti-nucleoprotein (anti-N) immunoglobulins G (IgG) at D21 in one participant was noted. For the remaining 15 that had not been infected before the trial, there were 11 cases of COVID-19 post-trial vaccinations (73%), eight diagnosed by antigenic or polymerase chain reaction (PCR) test (2/3 in the LD-Vehicle-GNP group; 5/10 in the LD-PepGNP-Covid19 group; 1/1 in the HD-Vehicle-GNP group and 0/1 in the HD-PepGNP-Covid19 group), and three by anti-N IgG seroconversion accompanied by a flu-like illness, and either no test or negative antigenic test, no PCR done (2/10 in the LD-PepGNP-Covid19 group and 1/1 in the HD-PepGNP-Covid19 group). Overall, regarding post-vaccination SARS-CoV-2 infection, there was no significant difference between vaccine and comparator groups, with a higher proportion of infections in the HD compared with the LD group likely due to fewer participants in the HD group not having already been infected prior to trial vaccination (2/3 [67%] in the LD-Vehicle-GNP group; 7/10 [70%] in the LD-PepGNP-Covid19 group; 1/1 [100%] in the HD-Vehicle-GNP group and 1/1 [100%] in the HD-PepGNP-Covid19 group). There was no case of severe COVID-19.

### Immunogenicity

#### Humoral responses

**Fig. 4:**
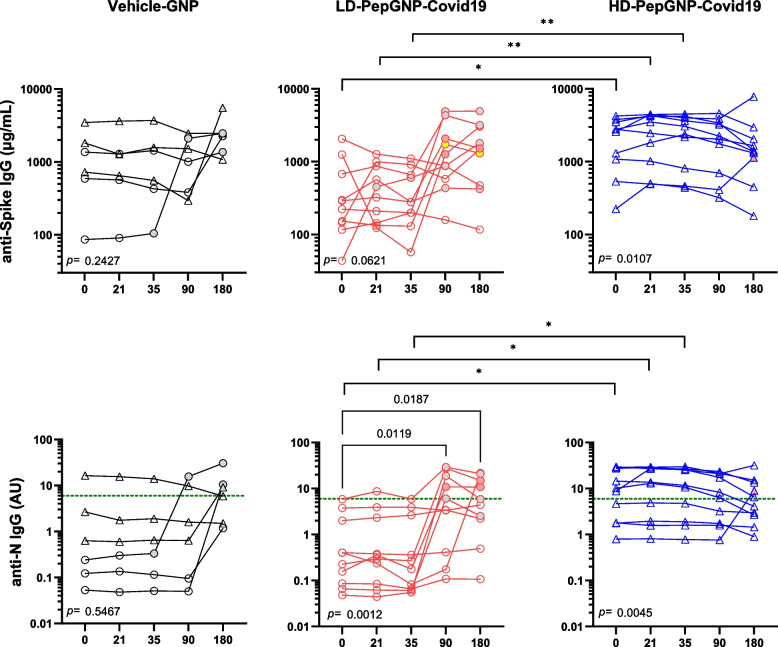
Anti-SARS-CoV-2 serology. Kinetics of anti-S (top panels) and anti-N (bottom panels) IgG levels are shown in Vehicle-GNP (*n* = 6), LD-PepGNP-Covid19 (*n* = 10) and HD-PepGNP-Covid19 (*n* = 10) groups. Intra-group comparisons using Friedman tests, *p* values < 0.05 are indicated below each panel. Inter-group comparison using Kruskal–Wallis tests at each time-point; *, *p* < 0.05; **, *p* < 0.01. Anti-N IgG titres above 6 AU (green line) are positive. Symbols: grey, COVID-19; pink, flu-like symptoms; yellow, Spike SARS-CoV-2 vaccination. N, nucleoprotein

Formulated with short MHC class I peptides mainly from SARS-CoV-2 N-protein, PepGNP-Covid19 was not designed to induce a humoral response. At baseline, all participants presented high anti-spike protein (anti-S) IgG titres, assumed to be due to previous vaccination (100% seropositivity, AU > 6). Due to a high COVID-19 incidence before D0, the HD-PepGNP-Covid19 group presented significantly higher levels of anti-S and anti-N IgG than the LD-PepGNP-Covid19 group at D0 (2676 [534.5;3796] vs 255.7 [116;1250] µg/mL, *p* = 0.021 and 9.505 [1.769;28.9] vs 0.315 [0.066;3.757] AU, *p* = 0.022, respectively) and a higher number of anti-N positivity (HD: 6/10, LD: 0/10) (Fig. 4). Post vaccination, the difference between the two PepGNP-Covid19 groups remained highly significant until D35, after which anti-S and anti-N IgG increased in the LD group reaching the level of responses in HD group from D90, with six anti-N seroconversions. At D180, the level of increase in anti-SARS-CoV-2 IgG was similar in the Vehicle-GNP and LD groups, whereas, in the HD group, the anti-SARS-CoV-2 titres tended to decrease (figure A3). No significant modulation of anti-SARS-CoV-2 immunoglobulin M (IgM) was observed in PepGNP-Covid19 groups. Positive anti-N IgG levels were observed in seven out of 11 pre-exposed participants at D0 (6/9 HD-PepGNP-Covid19 and 1/2 Vehicle-GNP participants). Two participants from the LD-PepGNP-Covid19 group who were SARS-CoV-2 exposed at the early phase of the study (positive PCR at D0 without symptoms or at D10 with signs for a clinical disease) did not mount an anti-N IgG response. Seven anti-N seroconversions occurred after eight cases of COVID-19 reported during the trial. Of six participants without reported COVID-19 (one HD-PepGNP-Covid19, one Vehicle-GNP, four LD-PepGNP-Covid19), three presented an anti-N seroconversion at D90 (*n* = 2, LD) and at D180 (*n* = 1, HD) that correlated with an episode of flu-like symptoms (at D63, D71 and D146, respectively). Therefore, the predominant increase in the LD-PepGNP-Covid19 group was due to infections.

#### Cellular responses

We analysed the induction of vaccine-specific CD8 T lymphocytes in peripheral blood mononuclear cells by measuring activation-induced markers (AIM), CD137 + CD69 + and CD107a + CD25 + , CD8 + and Covid19-dextramer + CD8 + , C-dextr + CD8, in parallel.

**Fig. 5:**
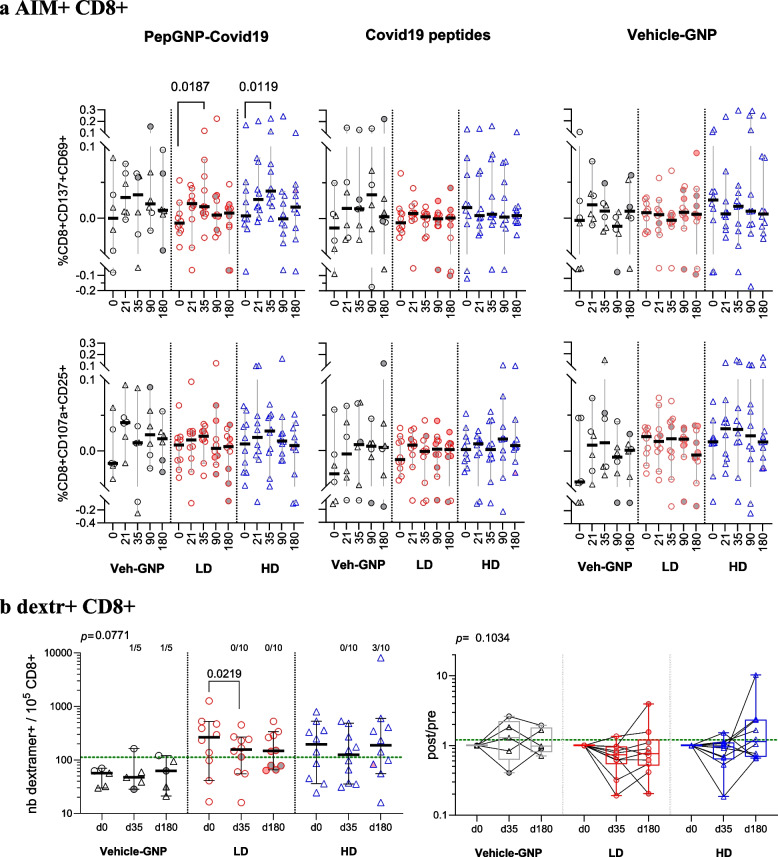
PepGNP-Covid19-elicited specific CD8 T cells. **a** AIM + CD8 responses. Kinetics of CD8 responses were evaluated in groups Vehicle-GNP (*n* = 6), LD-PepGNP-Covid19 (*n* = 10) and HD-PepGNP-Covid19 (*n* = 10). Results are expressed as percentage of Covid-specific CD8 + over total CD8 + , stimulated minus control (Vehicle-GNP or unstimulated), specific CD8 + defined as co-expressing CD137 and CD69 (top) or CD107a and CD25 (bottom) upon stimulation with PepGNP-Covid19 (left) or Covid19 peptides (right). **b** Dextramer CD8 responses were evaluated using eight Covid19 dextramers (C-dextr) in groups Vehicle-GNP (*n* = 5), LD-PepGNP-Covid19 (*n* = 10) and HD-PepGNP-Covid19 (*n* = 10) at D0, D35 and D180. Results are expressed as number of C-dextr + CD8 + T cells over 10^5^ total CD8 + T cells, as the sum of C-dextr + responses for each volunteer and number of responders (left), and as change from baseline as the ratio of post-/ pre-vaccination response (right). Responses to one to four C-dextr per volunteer were assessed. Green lines indicate cut-offs used to define responder: 112.2 C-dextr + CD8 + / 10.^5^ CD8 + T cells and a post/pre ratio of 1.2. a and b) intra-group comparison with D0 using Friedman tests; inter group comparisons using Kruskal–Wallis tests. Bars indicate medians and 95% CI, boxes indicate IQR. *p* values < 0.05 are indicated. Symbols: grey, reported COVID-19; pink, probable COVID-19

AIM analysis was performed on all participants, upon stimulation of PBMC with PepGNP-Covid19, peptides without particles (soluble peptides) or nanoparticles alone (Vehicle-GNP). PepGNP-Covid19 vaccination induced an increase from baseline in frequencies of PepGNP-Covid19-specific CD137 + CD69 + CD8 + in LD-PepGNP-Covid19 and HD-PepGNP-Covid19 groups at D35 (+ 0.0279% [−0.0035;0.0563] *p* = 0.0187 and + 0.0295% [0.0149;0.0747] *p* = 0.0119, respectively, Fig. 5a, figure A4) that decreased from D90. For the other AIM parameters measured, i.e., CD107a + CD25 + CD8 and response to stimulation with soluble peptides, PepGNP-Covid19 did not induce significant modulations of Covid-specific CD8 + in any of the two PepGNP-Covid19 groups. Moreover, the distribution of post-infection responses (grey and pink symbols) was homogeneous, indicating that infections occurring after D0 had little influence. If we consider Covid19 positive responses (Table 2), we observe 60–70% of responders in the HD-PepGNP-Covid19 (*n* = 6–7/10), 20–40% in LD-PepGNP-Covid19 (*n* = 2–4/10), and 67–83% in Vehicle-GNP (*n* = 4–5/6) groups. The application of a different cut-off for volunteers infected or not by SARS-CoV-2 before D0 gave 20–60% responders in HD-PepGNP-Covid19 (*n* = 2–6/10) (table A5).

**Table 2:** Covid19-specific responders in AIM, ITT analysis

Group	Vehicle-GNP, *n* = 6					LD PepGNP-Covid19, *n* = 10					HD PepGNP-Covid19, *n* = 10				
Day	21	35	90	180	any^a^	21	35	90	180	any	21	35	90	180	any
CD8 + CD107a + CD25 +	2 (33)	3 (50)	1 (17)	1 (17)	4 (67)	1 (10)	0 (0)	2 (20)	0 (0)	2 (20)	2 (20)	1 (10)	3 (30)	1 (10)	6 (60)
CD8 + CD137 + CD69 +	3 (50)	2 (33)	3 (50)	3 (50)	4 (67)	0 (0)	2 (20)	1 (10)	0 (0)	3 (30)	4 (40)	4 (40)	3 (30)	1 (10)	7 (70)
At least one co-marker^b^	3 (50)	4 (67)	3 (50)	2 (33)	5 (83)	0 (0)	2 (20)	1 (10)	0 (0)	3 (30)	4 (40)	3 (30)	4 (40)	1 (10)	7 (70)
Any marker +	4 (67)	4 (67)	3 (50)	3 (50)	5 (83)	1 (10)	2 (20)	2 (20)	0 (0)	4 (40)	5 (50)	5 (50)	4 (40)	1 (10)	7 (70)

Numbers (%) of responders per group are indicated for each Covid19-specific CD8 T cell parameter measured by AIM upon stimulation with pep-C or GNPC. Responders had a response above the cut-off and a positive change from baseline. Cut-off calculated based on mean response of uninfected volunteers at D0 (*n* = 15). ITT analysis

^a^At any time post vaccination

^b﻿^CD107a + CD25 + and/or CD137 + CD69 +

In parallel to Covid19-specific responses, we analyzed the kinetics of responses to nanoparticles. HD-PepGNP-Covid19 induced a Vehicle-GNP-specific CD107a + CD25 + CD8 + response of the same intensity as that specific to Covid-19 (Fig. 5a). Higher Vehicle-GNP-specific CD8 + and CD4 + responses were induced in subjects that received the high dose of GNP (figure A5a). Moreover, these responses were generally positively correlated with early local AEs for HD and with late local AEs for LD (figure A5b).

C-dextr + CD8 + T cell responses to vaccination were evaluated in 25 human leukocyte antigens (HLA)–matched volunteers (table A6, figure A6a) at D0, D35 and D180, using eight different Covid19 dextramers and one to four dextramers per volunteer (figure A6b). We observed a decrease from baseline in circulating C-dextr + CD8 + T cells levels at D35 in vaccinees, particularly in the LD-PepGNP-Covid19 group (ratio post/pre = 0.7335 [0.319;0.965] *p* = 0.0219, Fig. 5b). In this group, a higher baseline frequency of Covid-specific CD8 + T cells was associated with higher response decrease over 180 days post-vaccination (AUC analysis, appendix C, figure A7, Spearman r = −0.7818, *p* = 0.0105). Some late positive responses at D180 were observed in the HD-PepGNP-Covid19 group (n = 3/10), none in the LD group (Fig. 5b), and not related to COVID-19 infection. Inter-group comparisons of cumulative Covid19 responses showed an absence of significant difference between the median frequencies of C-dextr + CD8 + pre- and post-vaccination (Fig. 5b).

Therefore, vaccination induced PepGNP-Covid19-specific CD137 + CD69 + CD8 + and a decrease of C-dextr + CD8 + at D35. Moreover, as opposed to humoral responses, late CD8 + responses were not increased with post-vaccination infection.

**Fig. 6:**
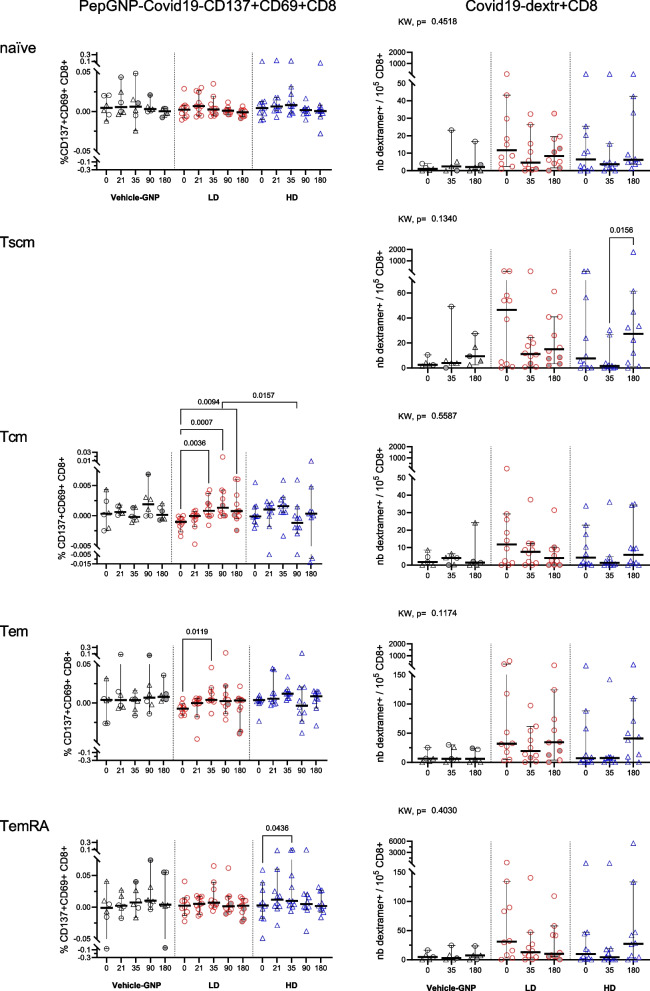
PepGNP-Covid19-specific CD8 + memory subsets. Covid19-specific responses were assessed in volunteers from group Vehicle-GNP (n = 5), LD-PepGNP-Covid19 (n = 10) and HD-PepGNP-Covid19 (n = 10), from D0 to D180, by measuring the frequency of specific AIM + CD8 + (PepGNP-Covid19-CD137 + CD69 + CD8 + , left panels) or using HLA class I compatible Covid19 dextramers from a list of eight peptide/flurochrome combinations (Covid19-dextr + CD8, right panels). Results are expressed as percentage of AIM + CD8 + and number of C-dextramer + CD8 + T cells + over 10.^5^ total CD8 + T cells for each memory subset for each volunteer. CD8 + T cells subsets were defined as naïve (CD45RA + CCR7 + CD95-), stem cell memory (Tscm, CD45RA + CCR7 + CD95 +), central memory (Tcm, CD45RA-CCR7 +), effector memory (Tem, CD45RA-CCR7-) and differentiated effector memory (TemRA, CD45RA + CCR7-). Responses to one to four C-dextramers minus negative control were summed per volunteer. Intra-group comparison with D0 used Friedman tests. Inter-group comparison used Kruskal–Wallis tests. *p* values < 0.05 are indicated. Bars indicate medians and 95% CI. Tscm not done for AIM. Symbols: grey, reported COVID-19; pink, probable COVID-19

Vaccine-induced T cells were characterized by analyzing naïve and memory subsets (Fig. 6). Early after vaccination, at D35, we observed an increase in AIM + Covid19-specific Tcm and Tem in LD-PepGNP-Covid19 vaccinees (+ 0.0021% [0.001;0.004] *p* = 0.0036 and + 0.0136% [0.005;0.0275] *p* = 0.0119, respectively), and in AIM + Covid19-specific TemRA (+ 0.023% [0.0031;0.0474] *p* = 0.0436; *p* > 0.05 with PP analysis) in HD-PepGNP-Covid19, while no significant change in the various dextr + CD8 + T cell memory subsets, except a decrease in C-dextr + Tscm at D35 in LD-PepGNP-Covid19 vaccinees (−28.36 [−36.33;−0.12] over 10^5^ CD8 + *p* = 0.0286, figure A8, PP analysis). Summed C-dextr + CD8 + memory T cells decreased in LD-PepGNP-Covid19 and also across all PepGNP-Covid19 vaccinees (−42 [−354.2;0] over 10^5^ CD8 + *p* = 0.0417 and −31.3 [−79.6;0] *p* = 0.0342, respectively, figure A9), as observed with total C-dextr + CD8 + T cells.

After D35, AIM + Covid19-Tcm remained at the plateau until D180 such that their rate was higher than that observed in the HD group at D90 (+ 0.0031% [0.0009;0.0055] vs −0.0008% [−0.0028;0.0029] *p* = 0.0157). At D180, dextr + CD8 + Tscm increased in HD-PepGNP-Covid19 vaccinees (27.35 [1.31;61.49] vs 1.42 [0;26.56] over 10^5^ CD8 + *p* = 0.0156). No modulation of CD8 + T cell subsets was observed in the Vehicle-GNP group.

Additionally, the functionality of C-dextr + CD8 + T cells was assessed with the expression of the CC-chemokine receptor CXCR3. CXCR3 directs migration to peripheral inflamed tissues and is a marker of Tc1 CD8 + , meaning cytotoxic CTL that secrete IFNγ, IL-2 and/or TNFα. The overall profile of CXCR3 + C-dextr + CD8 + memory cells mirrors that of C-dextr + CD8 + memory cells except the absence of Tscm modulation in HD group (figure A10).

At baseline, SARS-CoV-2 specific CD8 T cells are shown in a high proportion of uninfected subjects as seen previously [7, 9, 10]. Frequencies of SARS-CoV-2 specific CD8 T cells were comparable in uninfected and infected participants (figure A11a) but with differences in profiles, i.e., a tendency for a higher proportion of Covid19-specific CD137 + CD69 + CD8 + Tem in infected participants (*p* = 0.0727, figure A11c).

Vaccination induced Tem and Tcm in LD-PepGNP-Covid19 vaccinees, all uninfected and therefore with less Tem at baseline, and TemRA in HD-PepGNP-Covid19 vaccinees, who were generally previously infected.

## Discussion

The distinctive feature of PepGNP-Covid19 lies in its design – a synthetic peptide nanoparticle-based vaccine – which aims to specifically elicit, or boost, a CD8 + T cell response. In general, PepGNP-Covid19 appeared to be safe and well tolerated. Despite the relatively small number of subjects included in the trial, there was overall no clear difference in terms of reactogenicity between the vaccine and the comparator groups, and across the two dosages tested.

Frequent local reactogenicity was noted, in line with previous reports of intradermal COVID-19 vaccine administration [11, 12]. Participants in the Vehicle-GNP groups also reported local AEs, indicating that GNPs contributed to reactogenicity. There were more cases of early reactions in the HD group. An a- or pauci-symptomatic delayed local erythema and/or swelling was experienced by almost half of the participants, both in vaccine and comparator groups, as it was observed in the naNO-DENGUE trial using the same technology [8]. By contrast, late reactions occurred more frequently in the LD group. Early and delayed reactions faded over time but were still visible at the end of the six-month follow-up period for the majority of the participants concerned. Although in our study reactions could be delayed for several weeks, similar delayed reactions (appearing within a few days or weeks post-injection) were reported in a recent study involving patients with diabetes [13]. In this study, they used intradermal injection of GNP loaded with proinsulin peptide (C19A3-GNP) and passivated with glutathione and glucose, different from α-galactose and N-acetylglucosamine in PepGNP-Covid19. They found gold-specific T cells with frequencies similar to that specific to proinsulin at injection site [14]. Similarly, we obtained Vehicle-GNP-specific peripheral CD8 + and CD4 + T cells, especially with the high dose, and with frequencies similar to that specific to PepGNP-Covid19. In our study, early and late local reactions were correlated with Vehicle-GNP-specific T cell responses and the GNP dose. This suggests a reaction to GNP and/or α-galactose and N-acetylglucosamine. As these are bacterial mimetics that stimulate acquired immune recall and have adjuvant activity, it is not unexpected that the vehicle might contribute to local AEs. Nevertheless, since similar GNP but with different passivation and peptides, induced similar local reactions and GNP-specific T cells after intradermal injection, it is likely that gold itself contributes to local AEs. Besides, gold-specific T cells were shown to be associated with gold hypersensitivity reactions in rheumatoid arthritis patient after gold treatment [15]. It is noted that in contrast to the human study reported here, there were no indicators of local intolerance in studies of intradermal PepGNP-Covid19 vaccination performed in murine, rabbit and porcine animal models.

Systemic reactogenicity was not clearly dose-dependent either, since mild (Grade 1) and severe (Grade 3; two cases) reactions were similarly observed in LD and HD groups and only moderate (Grade 2) reactions were more frequent in the HD group. From the safety data, there is thus no obvious reason to choose one or the other dose. Regarding COVID-19 events during the whole trial period, 73% of the subjects who were not previously exposed got infected due to the epidemiological situation, and there was overall no significant difference between the groups. No case of severe COVID-19 was reported after PepGNP-Covid19, suggesting no enhancement of the disease due to the trial vaccine immune response, which is in line with other studies regarding vaccinations against COVID-19 [16, 17].

Whether PepGNP-Covid19 induced a humoral response was questioned given the increased anti-S and anti-N titres observed in the LD group. However, five cases of COVID-19 were reported in this group during the trial and there were two participants for whom SARS-CoV-2 infection likely went undetected (anti-N seroconversion that correlated with an episode of flu-like symptoms). Consequently, all anti-N seroconversions were linked to exposure to SARS-CoV-2. Therefore PepGNP-Covid19 vaccination probably does not induce any humoral response.

PepGNP-Covid19 vaccination induced an increase in frequencies of PepGNP-Covid19-specific CD137 + CD69 + CD8 + in LD-PepGNP-Covid19 and HD-PepGNP-Covid19 groups at D35, particularly Tcm and Tem in the LD group and TemRA in the HD group. In parallel, we observed a decrease in the frequency of C-dextr + CD8 + T cells, total memory and Tscm, at D35 in LD-PepGNP-Covid19, and an increase in C-dextr + CD8 + Tscm at D180 in HD-PepGNP-Covid19 vaccinees. Different CD8 + T cell responses, Tem and Tcm vs TemRA, are probably related to a difference in the SARS-CoV-2–specific profiles of volunteers at baseline, i.e. infected/uninfected. Dextramer technology detects T cells based on their TCR specificity even with low affinity, but it does not account for functional changes. AIM + CD8 + T cells indicate activation and functional response, The decrease in C-dextr + CD8 + may reflect tissue migration and sequestration of total specific CD8 + , and a homeostatic regulation following an initial expansion post-vaccination, while the population of functional and activable specific CD8 + were maintained in circulation or increased. However, both AIM + and dextramer + CD8 + T cell responses were increased following PepGNP-Dengue vaccination. Differences between the two formulations (peptides, peptide/gold ratio, passivation) could explain the difference in immune response. Moreover, the increase in C-dextr + CD8 Tscm at D180 could be due to the recirculation of these cells after release from the tissues. The ability of Tscm to recirculate is crucial for their function in providing long-term immunity and rapid response to re-infection or re-exposure to the same antigen.

Our study encountered some limitations. SARS-CoV-2-specific CD8 T cells were detected at baseline in both previously COVID-19 infected and non-infected volunteers, cells that were primed by natural infection and exposure to SARS-CoV-2 or to other coronaviruses within the beta sarbecoCoV group (or may suggest previous sterile immunity) [18]. The detection of dextramer-positive cells at baseline suggests that natural infection generates T cells with specificities that are the same as those that PepGNP-Covid19 is designed to prime/boost. Although the frequencies of CD8 + specific to PepGNP-Covid19 were comparable in participants regardless of their primary infection with SARS-CoV-2, at baseline, exposed participants were already primed to the nucleoprotein and tended to present a higher proportion of AIM + CD8 + Tem than uninfected participants. In the context of PepGNP-Covid19 vaccination, we were comparing the cellular responses to a priming dose (uninfected/no previous COVID-19 infection) with those to a boost (previously infected/COVID-19). The fact that exposed participants received the high dose of PepGNP-Covid19 vaccine while uninfected ones mainly received the low dose complicated the interpretation of the modulation of the CD8 + T cell responses. The complexity was intensified by the natural infections that occurred either between D0 and D35 (*n* = 3) and mostly after D35 (*n* = 9), although not correlated with vaccine-specific CD8 + responses. An analysis of T cell responses to antigens absent from Spike and this vaccine would have allowed assessment of the proportion of responses generated by natural infections. The LD and HD groups should have been homogeneous regarding COVID19 infection, and therefore the volunteers should have been immunized after the epidemic wave, one to two months later. Since there was no re-infection identified in the HD PepGNP-Covid19 group, the SARS-CoV-2-specific CD8 response post-D0 could most likely be the direct consequence of the vaccination protocol. Overall, the comparison of the responses induced by the doses of vaccine, LD and HD, remains difficult to interpret in view of the challenges faced in the context of the pandemic.

## Conclusions

The results of this first clinical trial of a synthetic nanoparticle-based T cell priming peptide vaccine against a viral pathogen are promising, with data demonstrating that the vaccine is safe and well tolerated. Participants experienced various degrees of humoral and CD8 + T cell responses. These findings encourage further clinical trials, involving larger numbers of subjects, to investigate this innovative SARS-CoV-2 vaccine candidate in appropriate epidemiological settings to better disentangle the effect of the vaccine versus that of natural infection on immunological responses.

## Supplementary Information

Supplementary Material 1. Appendix A: Inclusion and exclusion criteria. Appendix B: Candidate vaccine. Appendix C: Assessment of adverse events. Appendix D: Immunological analysis methods. Table A1. Covid19 peptides. Table A2. Dextramers. Table A3. List of markers for flow cytometry. Figure A1. Gating strategy, AIM analysis. Figure A2. Gating strategy, dextramer analysis. Appendix E: Results. Table A4: Safety profile. Table A5. Covid19-specific responders in AIM, differential cut-off, ITT analysis. Table A6. HLA Typing. Figure A3. Change from baseline of anti-Covid19 serology. Figure A4. Change from baseline of PepGNP-Covid19-elicited specific CD8 T cells using AIM. Figure A5. Vehicle-GNP-specific T cell response according to GNP dose. Figure A6. HLA-A and HLA-B allele results. Figure A7. C-dextr+CD8+ responses: correlation between AUC and baseline. Figure A8. Covid19-dextramer+ CD8+ memory subsets (PP analysis). Figure A9. All memory C-dextr+CD8+. Figure A10. CXCR3+C-dextr+CD8+ memory subsets. Figure A11. Frequency and profile of Covid19-specific CD8+ T cells in infected and uninfected participants at baseline. Figure A12. Dextramer controls.

Supplementary Material 2. Data Dictionary Codebook.

## Data Availability

The clinical study protocol is available online. According to the research protocol, the participants’ consent, and the Swiss data protection law, the coded data can be shared with the editor and the peer-reviewers during the peer-reviewing process to replicate the results of this paper only. However, data cannot be made available for the readers or for other research teams due to restrictions in the previously cited documents. Data dictionary and metadata related to the datasets are available for the readers through the Unisanté data repository, under the DOI: 10.16909/dataset/54.

## Abbreviations

AE: Adverse event
AESI: Adverse event of special interest
AIM: Activation-induced marker
Anti-N: Anti-nucleoprotein
Anti-S: Anti-spike protein
CD: Cluster of differentiation
CI: Confidence interval
COVID-19: Coronavirus disease
CRP: C-reactive protein
D: Days
DSMC: Data safety and monitoring committee
GNP: Gold nanoparticle
HD: High dose
HLA: Human leukocyte antigen
Ig: Immunoglobulin
ITT: Intention-to-treat
LD: Low dose
MHC-1: Class I major histocompatibility complex
PCR: Polymerase chain reaction
PP: Per protocol
SAE: Serious adverse event
Tcm: Central memory T lymphocyte
Tem: Effector memory T lymphocyte
TemRA: Late differentiated effector memory T lymphocyte
Tscm: Stem cell memory T lymphocyte
