# Supplementary material for: A gold nanoparticle/peptide vaccine designed to induce SARS-CoV-2-specific CD8 T cells: a double-blind, randomized, phase 1 study in Switzerland
Source: BMC Infect Dis. 2025 Apr 7;25:472. doi: 10.1186/s12879-025-10844-3 (PMC11974014; doi:10.1186/s12879-025-10844-3)
Supplement: Supplementary file 1 — Supplementary Material 1. Appendix A: Inclusion and exclusion criteria. Appendix B: Candidate vaccine. Appendix C: Assessment of adverse events. Appendix D: Immunological analysis methods. Table A1. Covid19 peptides. Table A2. Dextramers. Table A3. List of markers for flow cytometry. Figure A1. Gating strategy, AIM analysis. Figure A2. Gating strategy, dextramer analysis. Appendix E: Results. Table A4: Safety profile. Table A5. Covid19-specific responders in AIM, differential cut-off, ITT analysis. Table A6. HLA Typing. Figure A3. Change from baseline of anti-Covid19 serology. Figure A4. Change from baseline of PepGNP-Covid19-elicited specific CD8 T cells using AIM. Figure A5. Vehicle-GNP-specific T cell response according to GNP dose. Figure A6. HLA-A and HLA-B allele results. Figure A7. C-dextr+CD8+ responses: correlation between AUC and baseline. Figure A8. Covid19-dextramer+ CD8+ memory subsets (PP analysis). Figure A9. All memory C-dextr+CD8+. Figure A10. CXCR3+C-dextr+CD8+ memory subsets. Figure A11. Frequency and profile of Covid19-specific CD8+ T cells in infected and uninfected participants at baseline. Figure A12. Dextramer controls. [file 12879_2025_10844_MOESM1_ESM.pdf]

Supplementary material

Contents

Appendix A: Inclusion and exclusion criteria....2
Appendix B: Candidate vaccine....4
Peptide selection....4
Appendix C: Assessment of adverse events....5
Assessment of causality....5
Definition of Adverse Event of Special Interest (AESI)....5
Definition of Serious Adverse Event (SAE)....5
Definition of Suspected Unexpected Serious Adverse Reaction (SUSAR)....6
Appendix D: Immunological analysis methods....7
Vaccine-specific antibody response using Luminex....7
Cell mediated immunity....7
Covid19 peptides....7
Dextramers....8
HLA-typing....8
Covid-19–specific CD8+ T cells by AIM (activation-induced markers)....8
Dextramer+ CD8+ T cells....9
Memory subsets....9
Appendix E: Results....12
Serology anti-SARS-CoV-2....13
AIM....14
HLA typing....17
Dextramers....18
Covid19 Memory CD8....19
Baseline profile....24
Appendix F: References....26

Appendix A: Inclusion and exclusion criteria

This study included healthy men and healthy, non-pregnant, non-breastfeeding women between the ages of 18 and 45 years old who were living in Switzerland.

**Inclusion Criteria**	An individual must fulfil all of the following criteria in order to be eligible for trial enrolment:<br>1. Aged 18 to 45 years on the day of inclusion<br>2. Participant signed informed consent<br>3. Living in Switzerland
**Exclusion Criteria**	An individual fulfilling any of the following criteria was excluded from enrolment:<br>1. Participant is pregnant, lactating, or of childbearing potential*<br>2. Participation in the 4 weeks preceding the first trial vaccination or planned participation during the present trial period in another clinical trial investigating a vaccine, drug, medical device, or medical procedure<br>3. Receipt of any vaccine (including vaccination against COVID) in the 4 weeks preceding the first trial vaccination (excepting influenza vaccination, which may be received up to 2 weeks before first study vaccine) or planned receipt of any vaccine in the 4 weeks following each trial vaccination<br>4. Documented COVID-19 in the 4 weeks preceding the first trial injection<br>5. Receipt of immunoglobulins, blood or blood-derived products in the past 3 months<br>6. Known or suspected congenital or acquired immunodeficiency; or receipt of immunosuppressive therapy**<br>7. Self-reported or documented seropositivity for human immunodeficiency virus (HIV), hepatitis B natural infection (HBcAb positive serology), or hepatitis C<br>8. Known systemic hypersensitivity to any of the vaccine components (e.g., gold), or history of a life-threatening reaction to vaccines<br>9. Current alcohol abuse or drug addiction (reported or suspected)<br>10. Chronic illness that, in the opinion of the investigator, is at a stage where it might interfere with trial conduct or completion (i.e., any risk factor for severe COVID-19 including BMI > 30 kg/m^2)<br>11. Thrombocytopenia or any coagulation disorder<br>12. Identified as an Investigator or employee of the Investigator or study center with direct involvement in the proposed study, or identified as an immediate family member (i.e., parent, spouse, natural or adopted child) of the Investigator or employee with direct involvement in the proposed study (i.e., in the employment of the Tropivac clinic or DFRI unit at Unisanté)<br>13. Refusal to be informed in the event that relevant results concerning the participant's health are revealed

* An individual who does **not** have childbearing potential is defined as a female who is:

- Pre-menarche or post-menopausal for at least 1 year
- Surgically sterile
- Using an effective method of contraception from at least 4 weeks prior to the first vaccination until at least 10 weeks after the last vaccination (up to day 90). Effective contraception methods are defined as established oral, injected or implanted hormonal methods (for more than 1 month), placement of an intra-uterine device (for more than 1 month), barrier methods of contraception (condom or occlusive cap with spermicide), male (partner) or female (self sterilization) or abstinence of heterosexual intercourses.
- Has no heterosexual intercourses

** Such as anti-cancer chemotherapy or radiation therapy, within the preceding 6 months; or long-term systemic corticosteroid therapy (prednisone or equivalent for more than 2 consecutive weeks within the past 3 months)

**Exclusion Criteria at the Time of Vaccination**<br>*(where delayed administration is possible)*	The following events constituted contraindications to the administration of the investigational product on the day of planned vaccination.<br>The participant must have been followed until resolution of the event as with any medical event and may have been considered for vaccination at a later date (maximum 14 days later) or withdrawn at the discretion of the Investigator. Delays due to these events did not constitute a protocol deviation.<br>• Temperature of >37.5°C at the time of vaccination<br>• "Acute disease"*** at the time of vaccination<br>• If there was a clinical suspicion of COVID-19 (according to the clinician's judgement), the clinical team would have needed to wait for the result of the PCR test for SARS-CoV-2, even if the rapid test was negative, and the vaccination would have been delayed until the result came back negative, the symptoms had resolved, and 4 weeks had elapsed since the first positive test result.

***"Acute disease" is defined as the presence of a moderate or severe illness with or without fever according to the investigator's judgment. All vaccines can be administered to persons with a minor illness such as diarrhea, mild upper respiratory infection, with or without low-grade febrile illness, i.e., axillary temperature of ≤37.5°C.

Appendix B: Candidate vaccine

Peptide selection

For an accelerated pathway due to the ongoing pandemic at the time, the coronavirus peptides were selected via homology. SARS-CoV-1 virus-derived T cell epitopes associated with long-term memory were mapped to the SARS-CoV-1 virus proteome and then aligned to the SARS-CoV-2 sequence. SARS-CoV-2 homologues were confirmed to be present in the SARS-CoV-2 MHC class I expression library and tested for recognition by T cells of SARS-CoV-2-convalescent individuals. A vaccine candidate was subsequently designed, with eight peptides chosen for inclusion in the final construct, with certain criteria considered during the selection process, e.g., strain cross-reactivity (peptides from internal proteins that are better conserved), high population coverage (inclusion of epitopes binding particular HLA supertypes) and ease of manufacture. The vaccine was designed to enable universal protection against Betacoronavirus family members. The sequences of the eight peptides are described in table A1.

Appendix C: Assessment of adverse events

Assessment of causality

The relationship assessment criteria used in this trial are adapted from the International Conference on Harmonisation E2A guidelines.

**Not related**	**No Relationship**	No temporal relationship to study product ***and***<br>Alternate etiology (clinical state, environmental or other interventions)<br>***and***<br>Does not follow known pattern of response to study product
**Related**	**Possible**	Reasonable temporal relationship to study product; ***or***<br>Event not readily produced by clinical state, environmental or other interventions; ***or***<br>Similar pattern of response to that seen with other vaccines
	**Probable**	Reasonable temporal relationship to study product; ***and***<br>Event not readily produced by clinical state, environment, or other interventions ***or***<br>Known pattern of response seen with other vaccines
	**Definite**	Reasonable temporal relationship to study product; ***and***<br>Event not readily produced by clinical state, environment, or otherinterventions; ***and***<br>Known pattern of response seen with other vaccines

Definition of Adverse Event of Special Interest (AESI)

The list of AEs considered of special interest is adapted from the *D2.3Priority List of Adverse Events of Special Interest: COVID-19* document, published by the SPEAC (Safety Platform for Emergency vACcines), version 2.0, issued on 25th May 2020.

AESIs include (exhaustive list):

- Generalized convulsion
- Guillain-Barré Syndrome (GBS)
- Acute disseminated encephalomyelitis (ADEM)
- Thrombocytopenia
- Anaphylaxis
- Vasculitis
- AE grade 3

Definition of Serious Adverse Event (SAE)

A SAE is an AE that results in any of the following outcomes, whether considered related to the study intervention or not.

- Death
- Life-threatening event (i.e., the volunteer was, in the view of the Investigator, at immediate risk of death from the event that occurred).
    - This does not include an AE that, if it occurred in a more severe form, may have caused death.
- Persistent or significant disability or incapacity (i.e., substantial disruption of one's ability to carry out normal life functions).
- Hospitalisation, regardless of length of stay, even if it is a precautionary measure for continued observation, or prolongation of existing hospitalisation. Hospitalisation (including inpatient or outpatient hospitalisation for an elective procedure) for a pre-existing condition that has not worsened unexpectedly does not constitute a SAE.

- An important medical event (that may not cause death, be life threatening, or require hospitalisation) that may, based upon appropriate medical judgement, jeopardise the volunteer and/or require medical or surgical intervention to prevent one of the outcomes listed above.
    - Examples of such medical events include allergic reaction requiring intensive treatment in an emergency room or clinic, blood dyscrasias, or convulsions that do not result in inpatient hospitalisation.
- Congenital anomaly or birth defect. Rigorous testing, counselling and medical history taking worked to best ensure that pregnancies did not occur within the timeframe of this study.

Definition of Suspected Unexpected Serious Adverse Reaction (SUSAR)

A SUSAR was defined as an event thought to be possibly, probably or definitely related to the investigational product and both serious and unexpected. No category of SAE had been defined as "expected".

Appendix D: Immunological analysis methods

Vaccine-specific antibody response using Luminex

The humoral immune response induced by the candidate vaccine PepGNP-Covid19 was measured at IAL-CHUV by Luminex against the Spike protein (S) and the nucleoprotein (N) at Day 0, 21, 35, 90 and 180 in sera from all volunteers.

The method used for the diagnostic is semi-quantitative. Analysis of anti-S and anti-N IgG is performed on sera at a dilution of 1:300 and gives the results as the ratio of sample MFI on control MFI, with a positivity cut-off of 6 and a negative cut-off of 4[1]. We added standard curves using monoclonal IgG and IgM anti-S and anti-N (huIgG anti-S P5C3[1]; huIgM anti-S CR3022 from InvivoGen; hu anti-N CR3018 from Absolute antibody) and additional serial dilution of sera to convert the MFI signal to µg/mL of anti-S IgG , anti-S IgM and anti-N IgM or AU/mL of anti-N IgG.

Cell mediated immunity

The frequency of circulating CD8+ T cells specific to PepGNP-Covid19 peptides was assessed by measuring activation-induced markers (AIM) positive CD8+ T cells and dextramer positive CD8+ T cells [2,3]. For AIM analyses, the frequency of CD8+ cells expressing the activation co-markers CD137+CD69+ and/or CD107a+CD25+ was measured upon stimulation of peripheral blood mononuclear cells (PBMCs) with PepGNP-Covid19, pooled SARS-CoV-2 peptides present in PepGNP-Covid19 (table A1), Vehicle-GNP or unstimulated. For dextramer analyses, the response to SARS-CoV-2 peptides was assessed individually using eight fluorochrome-labelled COVID-19 dextramers (table A2). Additionally, we used anti-CD3, CD8, CD4, CCR7, CD45RA, CD95, and CXCR3 antibodies for phenotyping of antigen-specific cells (table A3).

Covid19 peptides

Eight peptides of nine to ten amino acids, from SARS-CoV-2 and present in the PepGNP-Covid19 formulation, were synthesized by JPT and supplied by Immudex (table A1). Covid19 peptides were pooled for in vitro stimulation of PBMC and assessment of AIM+CD8+ T cells (pep-C-specific responses).

Table A1. Covid19 peptides

Peptide identifier	Sequence	Viral origin	Virus protein	Position		HLA type
LLL	LLLDRLNQL	SARS-CoV-2	N	222	230	A*02
QFA	QFAPSASAFF	SARS-CoV-2	N	306	315	A*24
APS	APSASAFFGM	SARS-CoV-2	N	308	317	B*07
MEV	MEVTPSGTW	SARS-CoV-2	N	322	330	B*44
VTP	VTPSGTWLTY	SARS-CoV-2	N	324	333	A*01, A*29, A*30
TPS	TPSGTWLTY	SARS-CoV-2	N	325	333	A*11, B*35
LLN	LLNKHIDAYK	SARS-CoV-2	N	352	361	A*03, A*30
RLN	RLNEVAKNL	SARS-CoV-2	S	1185	1193	A*02

N, nucleoprotein; S; Spike protein.

Dextramers

Eight Covid19 dextramers and 14 control dextramers (CMV, EBV and Flu as positive controls), labeled in PE or APC (MHC Dextramer®, Immudex, DK) were used for this series of analysis (table A2).

Table A2. Dextramers

Target	Protein	Sequence	HLA	Fluorochrome	Acronym
Covid19-dextramers, C-dextramers					
SARS-CoV-2	N	LLLDRLNQL	A*0201	APC	**LLL**
SARS-CoV-2	S	RLNEVAKNL	A*0201	APC	**RLN**
SARS-CoV-2	N	LLNKHIDAYK	A*0301	APC	**LLN**
SARS-CoV-2	N	QFAPSASAFF	A*2402	APC	**QFA**
SARS-CoV-2	N	VTPSGTWLTY	A*2902	APC	**VTP**
SARS-CoV-2	N	VTPSGTWLTY	A*0101	PE	**VTP**
SARS-CoV-2	N	APSASAFFGM	B*0702	PE	**APS**
SARS-CoV-2	N	TPSGTWLTY	B*3501	PE	**TPS**
Control dextramers					
CMV	PP65	NLVPMVATV	A*0201	APC	**NLV**
EBV	BRLF1	GLCTLVAML	A*0201	APC	**GLC**
CMV	IE-1	KLGGALQAK	A*0301	APC	**KLG**
EBV	EMNA 3A	RLRAEAQVK	A*0301	APC	**RLR**
EBV	LMP-2	TYGPVFMCL	A*2402	APC	**TYG**
CMV	pp65	QYDPVAALF	A*2402	APC	**QYD**
CMV	UL44	VTEHDTLLY	A*0101	PE	**VTE**
Flu	NP	CTELKLSDY	A*0101	PE	**CTE**
CMV	pp65	TPRVTGGGAM	B*0702	PE	**TPR**
EBV	EBNA 3A	RPPIFIRRL	B*0702	PE	**RPP**
CMV	PP65	IPSINVHHY	B*3501	PE	**IPS**
EBV	EBNA 1	HPVGEADYFEY	B*3501	PE	**HPV**
neg				APC	**neg**
neg				PE	**neg**

N, nucleoprotein; S; Spike protein. APC, allophycocyanin. PE, R-phycoerythrin.

HLA-typing

Class I HLA typing was performed by RT-PCR, using the kit LinkSeq HLA ABCDRDQDP SABR (Linkage Bioscience)**.**

Covid-19–specific CD8^{+} T cells by AIM (activation-induced markers)

The cell-mediated immune (CMI) response induced by the candidate vaccine PepGNP-Covid19 was determined by cytometry assessment of the induction of Activation induced markers (AIM)[4,5]. The frequency of peripheral blood mononuclear cells (PBMC) positive for CD69, CD107a, CD137, CD25 in response to in vitro stimulation using vaccine synthetic free peptides or whole vaccine at Day 0, 21, 35, 90 and 180 (visits 2, 6, 9, 11 and 12) was assessed in all participants.
After thawing, PBMC were distributed in U bottom plates, in duplicate, 1 to 2 million cells per stimulation. Cells were stimulated with a pool of eight Covid19 peptides (2 µg/ml) present in vaccine PepGNP-Covid19 (Covid19 peptides), 0.6 µM of PepGNP-Covid19, an equivalent concentration of Vehicle-GNP (3.6 µg/ml of gold), 5 µg/ml of staphylococcal enterotoxin B (SEB, S4881, Sigma, as positive control) or unstimulated (X-vivo-15), in the presence of anti-CD107a-PE (eBiosciences, 1:100). After 24h of culture, cells were transferred to a V-bottom plate and washed in PBS 0.1% BSA 2mM

EDTA. Cells were stained with Live/dead, anti-CD3, anti-CD4, anti-CD8, anti-CD137, anti-CD25, anti-CD69, anti-CCR7 and anti-CD45RA (table A3) in the presence of 50 µg/ml of human IgG (Privigen, CSL Behring) for 20 min at 4°C. After washing, cells were resuspended in FACS Lysing buffer (BD) (10 min, at RT) then resuspended in PBS 0.1% BSA 2mM EDTA and stored at 4°C before acquisition. Acquisition was performed on a LSR Fortessa and analysis performed on Flow-Jo v10.8.1 (RRID:SCR_008520). Gating strategy is shown in figure A1. Results are expressed as frequencies of CD8+ T cells expressing 1) the co-markers CD107a+CD25+, 2) the co-markers CD137+CD69+ or 3) at least one of the two co-marker combinations; stimulated minus negative control (unstimulated for Covid19 peptides, Vehicle-GNP and SEB; Vehicle-GNP for PepGNP-Covid19). Individual change from baseline was defined as the delta post minus pre i.e., frequency of specific T cells post-vaccination minus frequency of specific T cells pre-vaccination (D0). Responders had a positive delta post-pre response and a T cell response above a cut-off defined as the mean Covid-specific response in all uninfected volunteers at D0 + 2 SD (n=15). A differential cut-off defined as the mean Covid-specific response in all previously infected volunteers at D0 + 2 SD (n=11) was calculated for the analysis shown in table A6.

Dextramer+ CD8+ T cells

The panel of SARS-CoV-2 and control dextramers was used according to the HLA type of volunteers.
The kinetics of the specific CD8+ T cell response to seven Covid19 peptides were assessed individually at D0, D35 and D180 using eight Covid19 dextramers (C-dextr) labelled with PE or APC and described in table A2. After thawing, PBMCs taken at various time-points were distributed in 96-well V bottom plates, 3 million cells per well, one to 3 wells per time-point, depending on the HLA type of the individual. For each volunteer, a pool of PBMCs from different time-points was used for positive and negative control wells. First, cells were incubated (30 min at 37°C) with 25 µL of dasatinib 50nM. Then, 55 µL of PBS-0.5% BSA containing one or two C-dextr in two fluorochromes, two negative dextramers (PE and APC) or two to six positive control dextramers were added and cells stained for 10 min at RT. Pools of dextramers were buffered with PBS 10x and completed with 1·78 µM biotin. Finally, 20 µL of a 5x concentrated surface staining mix containing Live/Dead, anti-CD3, anti-CD8, anti-CCR7, anti-CD45RA, anti-CD95, anti-CXCR3 (table A3) in the presence of 50 µg/mL of human IgG (Privigen, CSL Behring) was added per well (final volume of 100 µL per well). Cells were stained for 20 min at 4°C. After washing, cells were fixed (BD Cell Fix) for 5 min at RT then resuspended in PBS 0.1% BSA 2mM EDTA and stored at 4°C before acquisition. Acquisition was performed on an LSR Fortessa and analysis performed on Flow-Jo v10.8.1 (RRID:SCR_008520). Gating strategy is shown in figure A2. For results expressed as frequencies of CD8+ T cells dextramer positive (number per 10^5 CD8 T cells), change from baseline was defined as the ratio of post-vaccination on pre-vaccination response, and a cut-off for positive responses with a high likelihood ratio was defined based on a ROC analysis between negative and positive control dextramers (figure A12). Volunteers with a ratio post/pre vaccination above 1.2 and a frequency of C-dextr+CD8+ above the cut-off (112.2 dextr+CD8+ / 10^5 CD8+ T cells) were identified as responders. For results expressed as frequencies of C-dextr+CD8+ T cells minus frequency of negative control dextr+ CD8+ T cells (number per 10^5 CD8 T cells), change from baseline was defined as the delta of post-vaccination minus pre-vaccination response.

Memory subsets

As part of an exploratory analysis, vaccine-induced AIM+ or dextr+CD8+ T cells were further characterized by examining naïve (CD45RA+CCR7+CD95-), stem cell (Tscm, CD45RA+CCR7+CD95+), central (Tcm, CD45RA-CCR7+), effector (Tem, CD45RA-CCR7-), and terminally differentiated (TemRA, CD45RA+CCR7-) memory subsets.

Table A3. List of markers for flow cytometry

Marker	Clone	Fluorophore	Supplier	cat #	RRID	Dilution
CD3	UCHT1	A700	BD	557943	AB_396952	20
CCR7	150503	FITC	R&D	FAB197F	AB_2259847	32
CD45RA	HI100	BV711	BD	563733	AB_2738392	100
LiveDead		vivid aqua	Thermo Fisher Scientific	L34957		800
AIM staining						
CD8	SK1	BV605	BD	564116	AB_2869551	100
CD4	RPA-T4	PB	BD	558116	AB_397037	50
CD137	4B4-1	APC	biolegend	309810	AB_830672	100
CD69	L78	PerCP	BD	340548	AB_400054	20
CD25	M-A251	PE-CY7	BD	557741	AB_396847	20
CD107a	H4A3	PE	eBioscience	12-179-42	AB_10853326	100
Dextramer staining						
CD8	RPA-T8	PB	BD	558207	AB_397058	20
Dextramer		PE	Immudex			
Dextramer		APC	Immudex			
CD95	DX2	PE-cy7	BD	561633	AB_10894384	50
CXCR3	g025h7	PE/Dazzle 594	biolegend	353736	AB_2564288	50

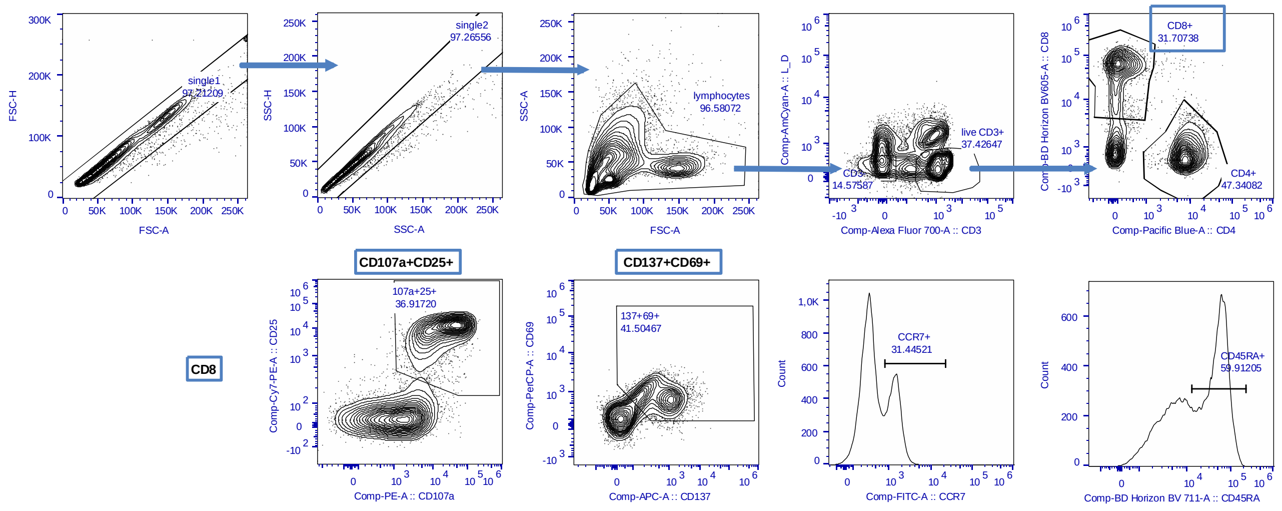

Figure A1. Gating strategy, AIM analysis. PBMC stimulated with SEB

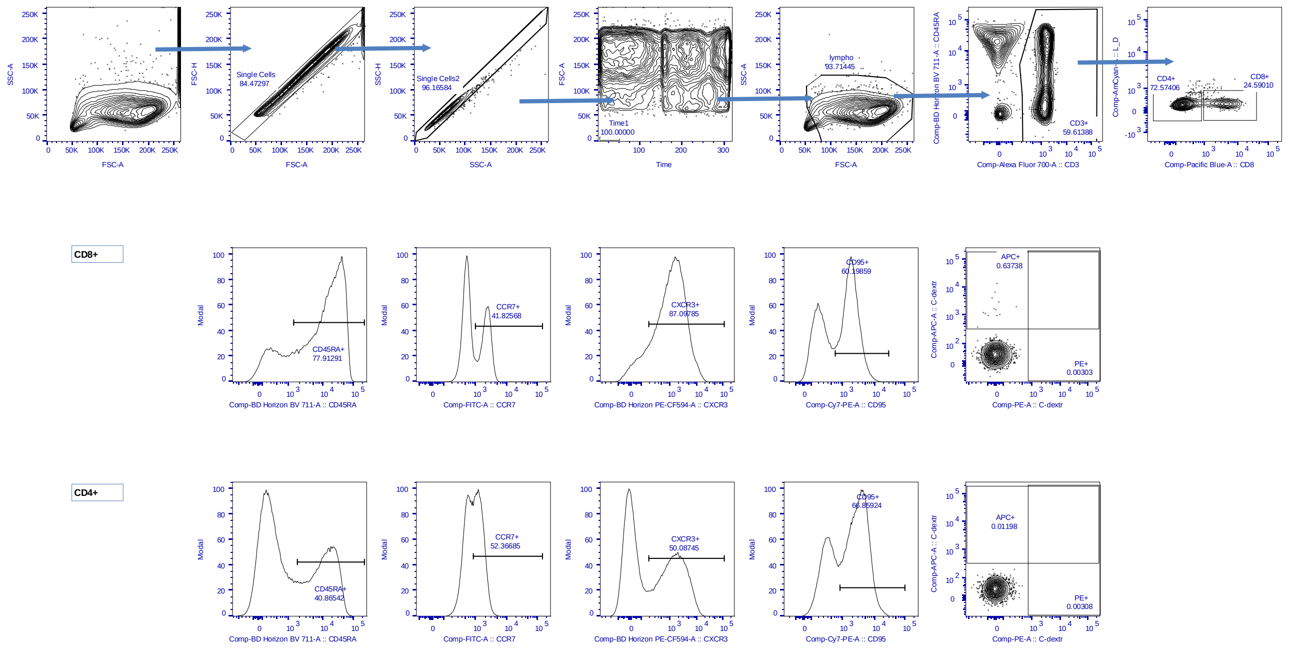

Figure A2. Gating strategy, dextramer analysis. PBMC stained with positive control class-I dextramers labelled with APC.

Appendix E: Results

Safety

Table A4: Safety profile

	LD vehicle-GNP (n=3)	LD PepGNP-Covid19 (n=10)	HD vehicle-GNP (n=3)	HD PepGNP-Covid19 (n=10)
**Solicited related local AE***				
Mild (Grade 1)	3/3 (100%)	10/10 (100%)	3/3 (100%)	10/10 (100%)
Moderate (Grade 2)	-	-	-	1/10 (10%)
Severe (Grade 3)	-	-	-	-
**Solicited related systemic AE****				
Mild (Grade 1)	2/3 (67%)	9/10 (90%)	2/3 (67%)	8/10 (80%)
Moderate (Grade 2)	1/3 (33%)	2/10 (20%)	2/3 (67%)	5/10 (50%)
Severe (Grade 3)	-	1/10 (10%)	-	1/10 (10%)
**Unsolicited related local AE#**				
Mild (Grade 1)	3/3 (100%)	8/10 (80%)	3/3 (100%)	10/10 (100%)
Moderate (Grade 2)	-	-	-	-
Severe (Grade 3)	-	-	-	-
**Unsolicited related systemic AE#**				
Mild (Grade 1)	3/3 (100%)	8/10 (80%)	1/3 (33%)	9/10 (90%)
Moderate (Grade 2)	-	1/10 (10%)	-	2/10 (20%)
Severe (Grade 3)	-	-	-	-
**Adverse event of special interest related#**	-	1/10 (10%)	-	1/10 (10%)
**Serious adverse event#**	-	-	-	-
**Death#**	-	-	-	-

Includes participants with at least one clinical or laboratory adverse event. Data are n (%). *Within 7 days of any injection. **Within 14 days of any injection. #During the 180-day study follow-up. LD=low-dose; HD=high-dose; GNP=gold nanoparticles.

Serology anti-SARS-CoV-2

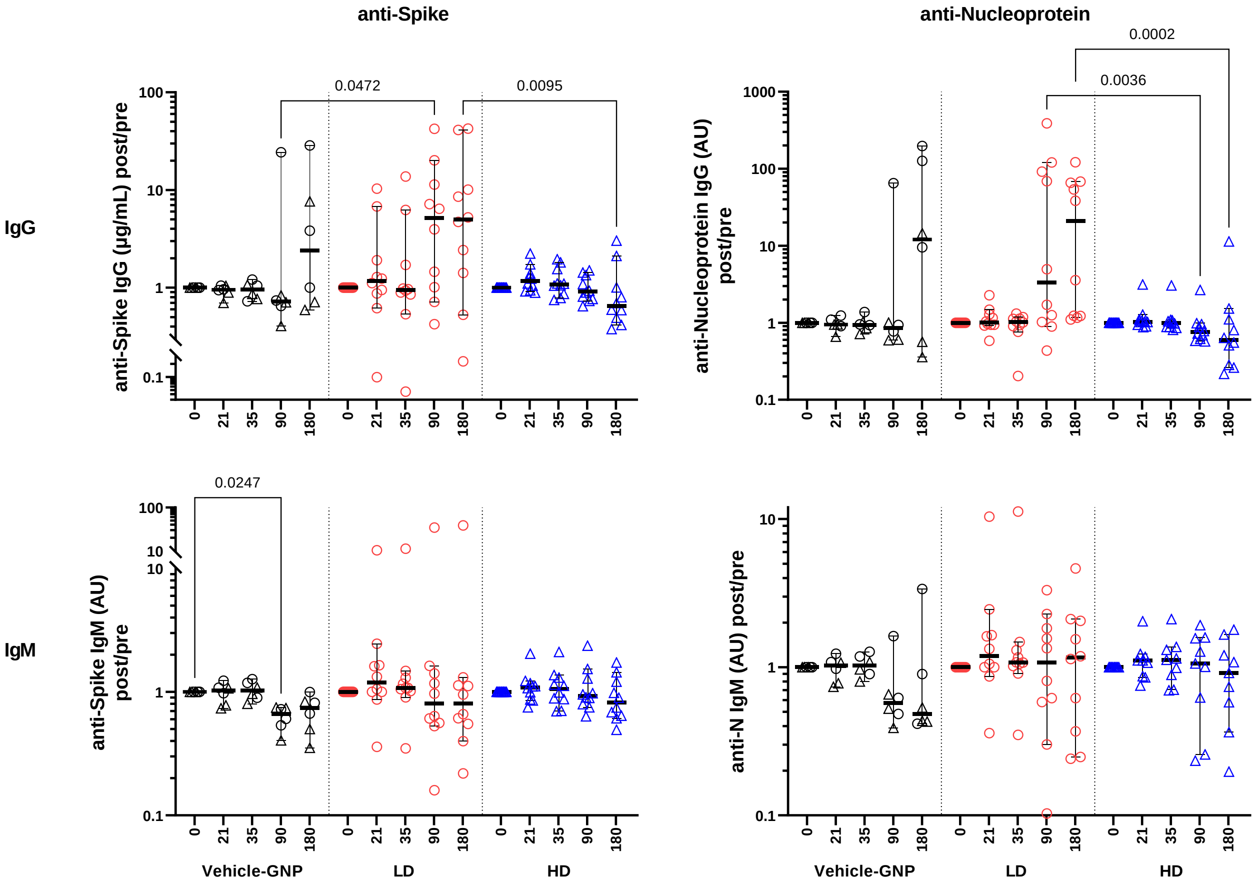

Figure A3. Change from baseline of anti-Covid19 serology.

Kinetics of anti-Spike (left panels) and anti-Nucleoprotein (right panels) IgG (top) and IgM (bottom) levels are shown in groups Vehicle-GNP (n=6), LD-PepGNP-Covid19 (n=10) and HD-PepGNP-Covid19 (n=10). Results are expressed as fold change from baseline, from D0 to D180. Bars indicate medians and 95% CI. Intra-group comparisons using Friedman tests. Inter-group comparison using Kruskal-Wallis tests at each time-point. *p* values <0.05 are indicated.

AIM

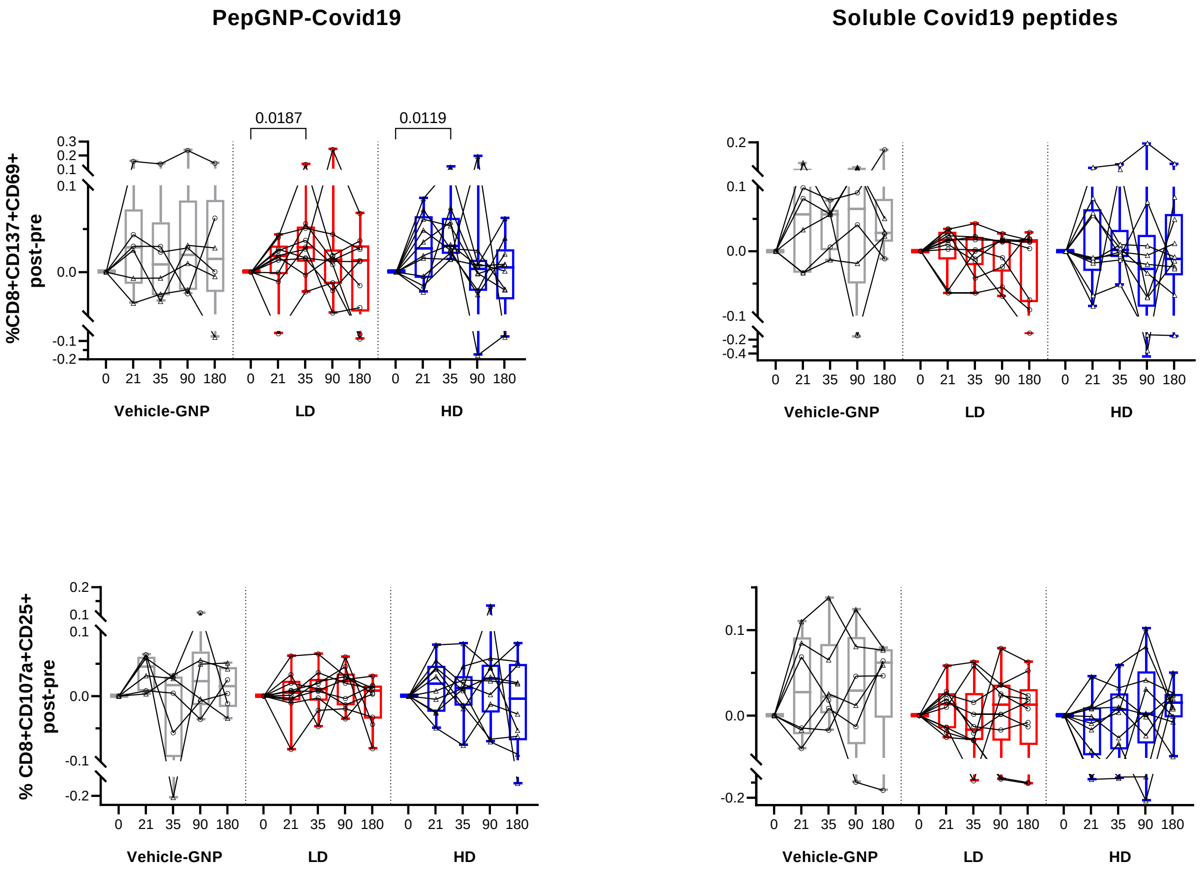

Figure A4. Change from baseline of PepGNP-Covid19-elicited specific CD8 T cells using AIM. Kinetics of CD8 responses were evaluated in groups Vehicle-GNP (n=6), LD-PepGNP-Covid19 (n=10) and HD-PepGNP-Covid19 (n=10). Results are expressed as change from baseline as the delta of post- minus pre-vaccination response of Covid-specific CD8+ over total CD8+, stimulated minus Vehicle-GNP/unstimulated, specific CD8+ defined as expressing co-markers CD137+CD69+ (top) or CD107a+CD25+ (bottom) upon stimulation with PepGNP-Covid19 (left) or Covid19 peptides (right). Intra-group comparison with D0 using Friedman tests; inter group comparisons using Kruskal-Wallis tests. Bars or boxes indicate medians and interquartiles. *p* values <0.05 are indicated.

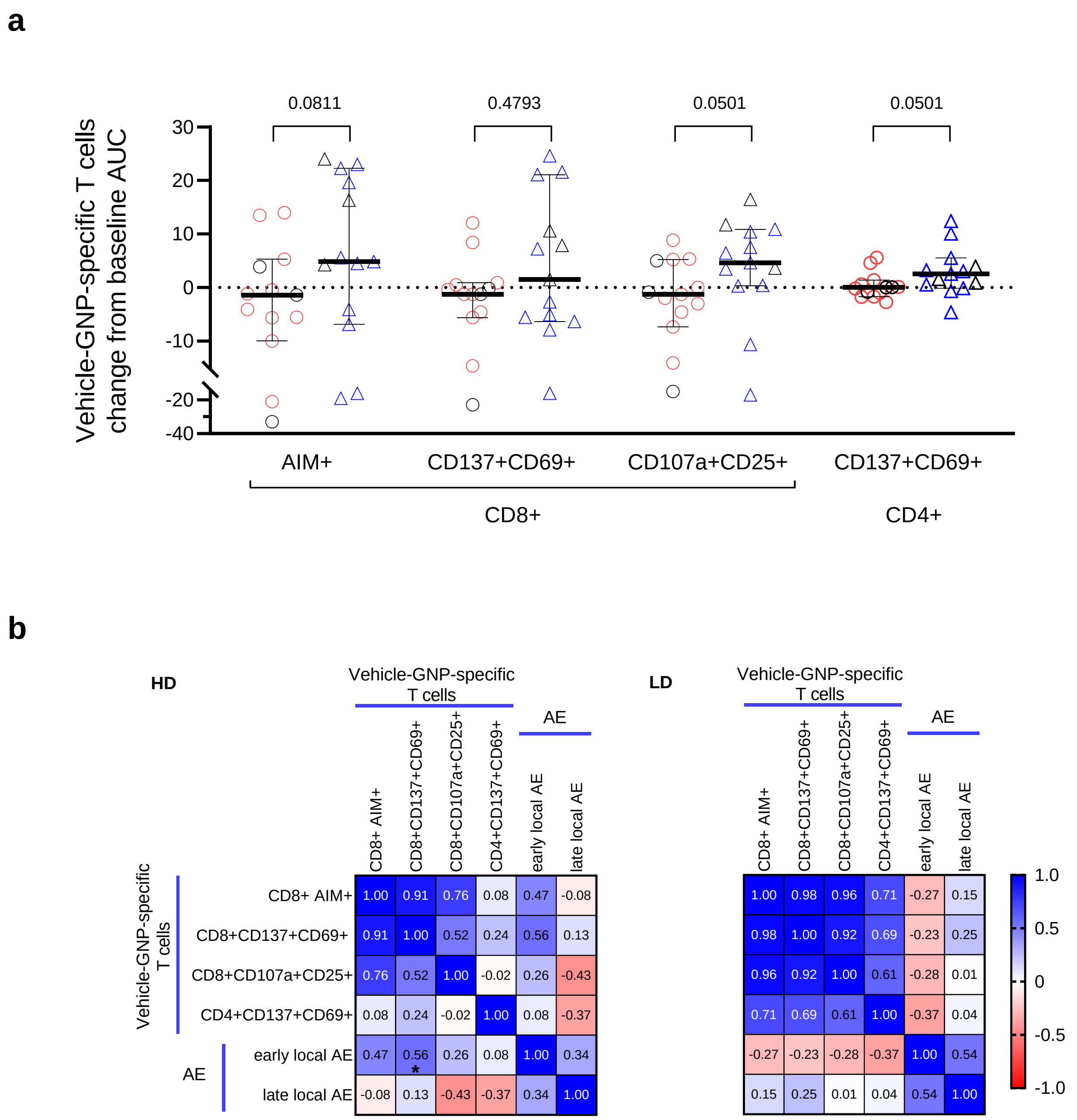

Figure A5. Vehicle-GNP-specific T cell response according to GNP dose. Kinetics of CD8 and CD4 responses were evaluated using AIM in LD GNP group, LD-PepGNP-Covid19 (red circle, n=10) and LD-Vehicle-GNP (black circle, n=3), and HD GNP group, HD-PepGNP-Covid19 (blue triangle, n=10) and HD-Vehicle-GNP (black triangle, n=3). (a) Results are expressed as the AUC of the change from baseline as the delta of post- minus pre-vaccination frequency of specific CD8+ or CD4+ over total population. Specific T cells were defined as expressing co-markers CD137+CD69+, CD107a+CD25+ or at least one of the two co-markers (AIM+) upon stimulation with Vehicle-GNP, stimulated minus unstimulated. Co-marker CD107a+CD25+ was not used for CD4+ T cells since subsets of CD4 constitutively express CD25. Inter group comparisons used Mann-Whitney tests. Bars indicate medians and 95% CI. (b) Spearman correlations between Vehicle-GNP-specific T cell responses (AUC) and number of vaccine-related local adverse events (AE), erythema and swelling, that occurred early (D0-D7) or late (D8-D180) in HD and LD GNP groups, * p=0.051.

Table A5. Covid19-specific responders in AIM, differential cut-off, ITT analysis

Group	Vehicle-GNP, n=6					LD PepGNP-Covid19, n=10					HD PepGNP-Covid19, n=10				
**Day**	21	35	90	180	any[§]	21	35	90	180	any	21	35	90	180	any
**CD8+CD107a+CD25+**	2	2	1	1	4	1	0	2	0	2	2	0	2	1	5
**CD8+CD137+CD69+**	3	2	3	3	4	0	2	1	0	3	1	2	1	0	3
**At least one co-marker[$]**	3	3	3	2	4	0	2	1	0	3	1	1	1	0	2
**Any marker+**	4	3	3	3	5	1	2	2	0	4	3	2	2	1	6

Numbers of responders per group are indicated for each Covid19-specific CD8 T cell parameter measured by AIM upon stimulation with pep-C or GNPC. Responders had a response above the cut-off and a positive change from baseline. Differential cut-offs were applied for those not infected at D0 (n=15) and for those Covid19-infected before D0 (n=11). [§] at any time post vaccination. [$] CD107a+CD25+ and/or CD137+CD69+. ITT analysis

HLA typing

Table A6. HLA Typing

HLA I	Total	Vehicle-GNP	LD PepGNP-Covid19	HD PepGNP-Covid19
	26	6	10	10
**A*01**	5	1	1	3
**A*02**	14	2	6	6
**A*03**	8	0	5	3
**A*24**	7	2	2	3
**A*29**	1	0	1	0
A*30	1	0	1	0
**B*07**	7	0	4	3
**B*35**	5	1	1	3
B*44	5	1	2	1
**(-) dextramer**	1	1	0	0

Results expressed as number of participants. In bold, results considered for dextramer assessment of Covid19-specific CD8+ T cells, restricted to HLA-A*01, A*02, A*03, A*24, A*29, B*07 and B*35. For the AIM evaluation, all participants presented at least one HLA susceptible to bind one peptide from PepGNP- Covid19, i.e., HLA-A*01, A*02, A*03, A*11, A*24, A*29, A*30, B*07, B*35 and B*44.

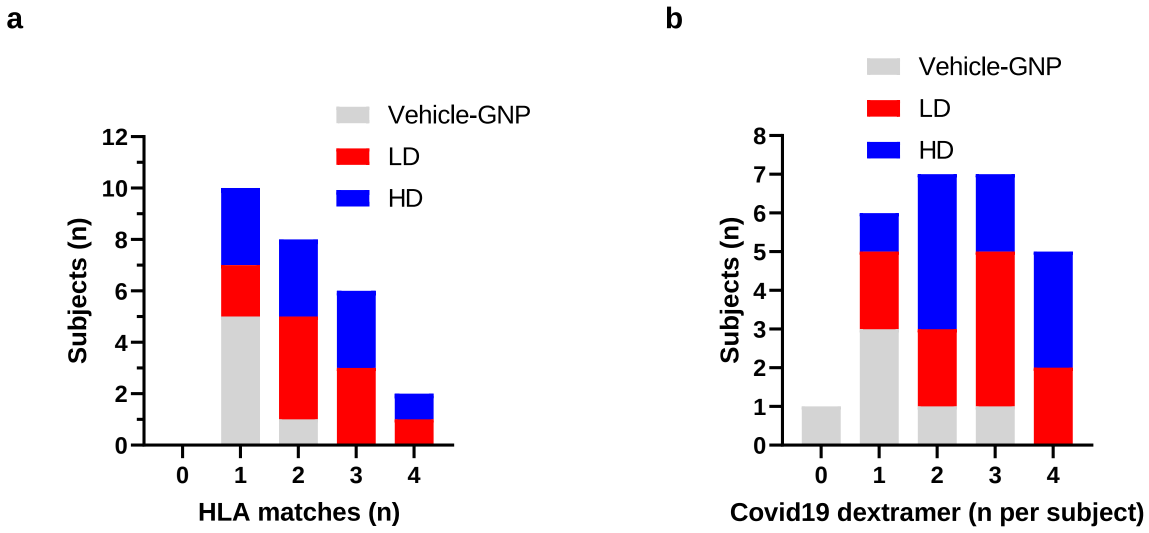

Figure A6. HLA-A and HLA-B allele results

(a) HLA-A and HLA-B matches. For each subject in the PepGNP-Covid19 LD and HD (n=10 each) or in Vehicle-GNP (n=6), the number of HLA-A and HLA-B alleles with known/potential binding to vaccine peptides was determined and the number of subjects with zero to four HLA matches plotted as a distribution chart. (b) Number of HLA-class I Covid19 dextramers (table A2) evaluated per volunteer according to their HLA typing. One to four Covid19 dextramers were evaluated per volunteer. HLA class I typing was performed by RT-PCR on blood samples drawn on Day 0. HLA, human leukocyte antigen; RT-PCR=reverse transcription polymerase chain reaction; LD=low-dose; HD=high-dose; GNP=gold nanoparticles.

Dextramers

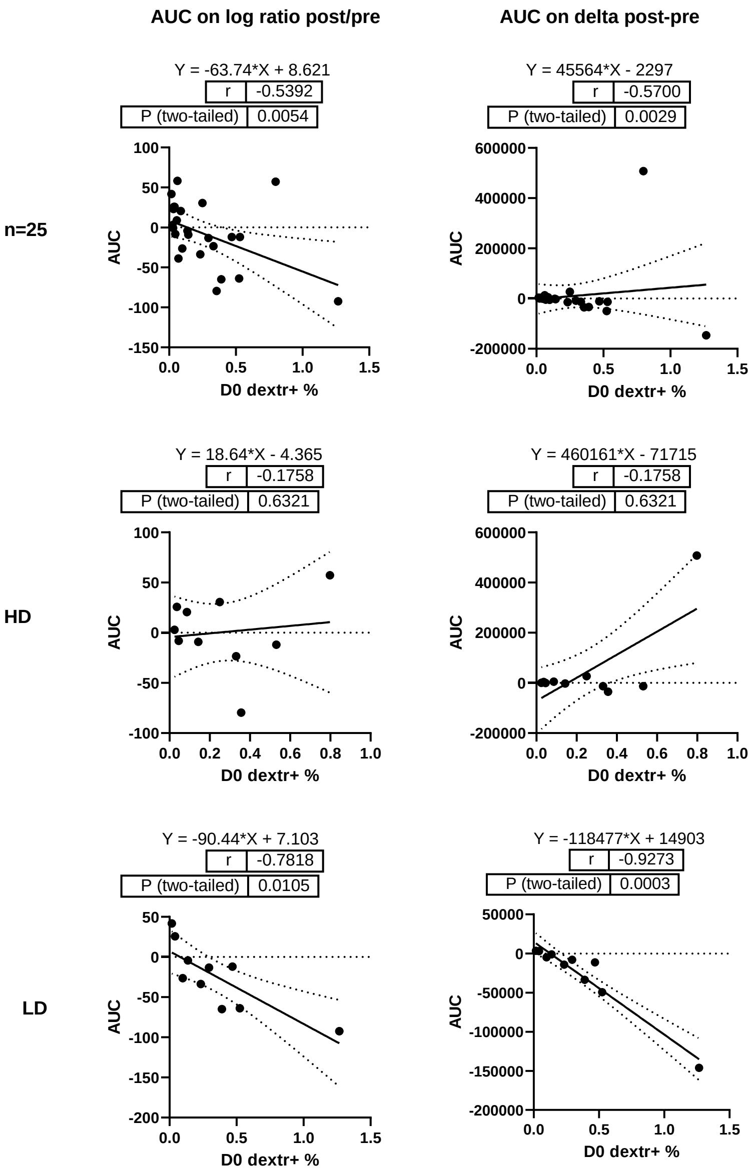

Figure A7. C-dextr+CD8+ responses: correlation between AUC and baseline.

Peptide-specific responses were assessed in volunteers from group Vehicle-GNP (n=5), LD-PepGNP-Covid19 (n=10) and HD-PepGNP-Covid19 (n=10), using HLA class I-compatible Covid19 dextramers. Spearman correlation between AUC of response over time (D0 to D180) and baseline frequency of dextr+CD8 was performed. AUC was calculated on change from baseline response using the log of post/pre ratio and post- pre delta

Covid19 Memory CD8

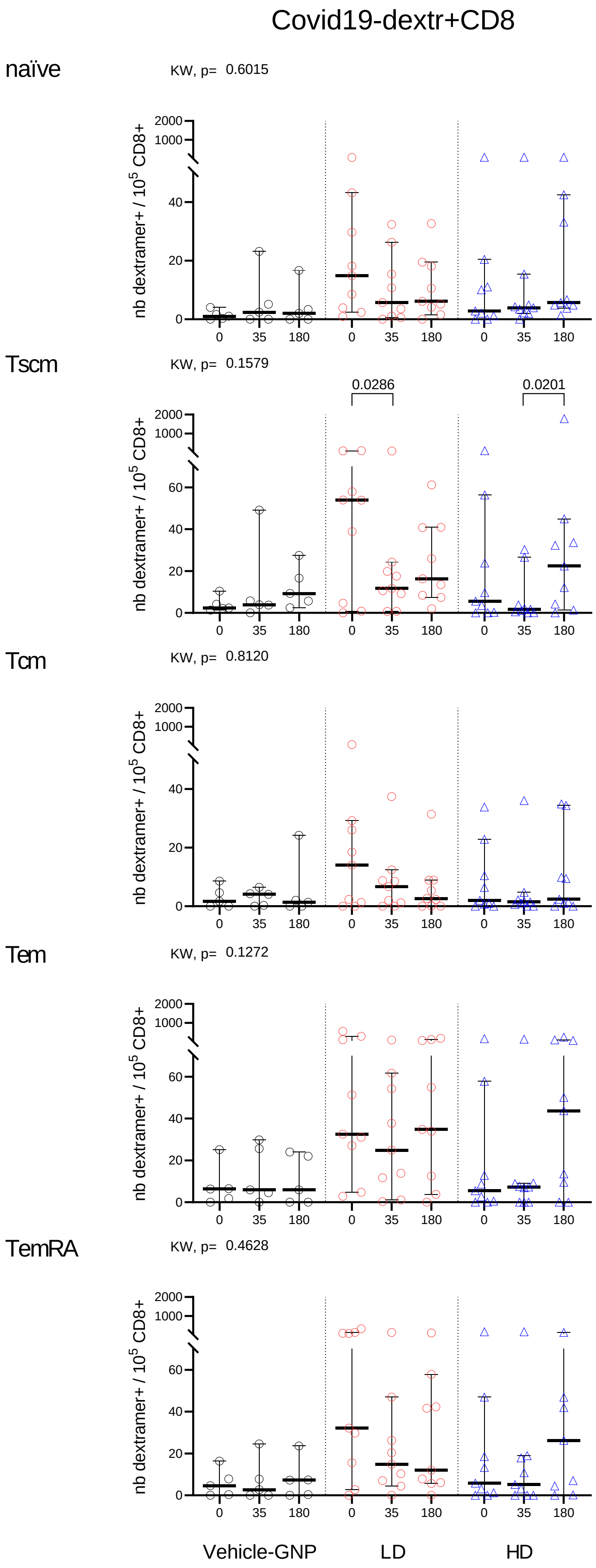

Figure A8. Covid19-dextramer+ CD8+ memory subsets (PP analysis). Covid19-specific responses were assessed in volunteers from group Vehicle-GNP (n=5), LD-PepGNP-Covid19 (n=9) and HD-PepGNP-Covid19 (n=9), at D0, D35 and D180, by measuring the frequency of specific HLA class I compatible Covid19 dextramers from a list of eight combinations. Results are expressed as number of subsets of C-dextramer+ CD8+ T cells + over 10^5 total CD8+ T cells for each volunteer. CD8+ T cells subsets were defined as naïve (CD45RA+CCR7+CD95-), stem cell memory (Tscm, CD45RA+CCR7+CD95+), central memory (Tcm, CD45RA-CCR7+), effector memory (Tem, CD45RA-CCR7-) and differentiated effector memory (TemRA, CD45RA+CCR7-). Responses to one to four C-dextramers minus negative control were summed per volunteer. Intra-group comparison with D0 used Friedman tests. Inter-group comparison used Kruskal-Wallis tests. *p* values <0.05 are indicated. Bars indicate medians and 95% CI.

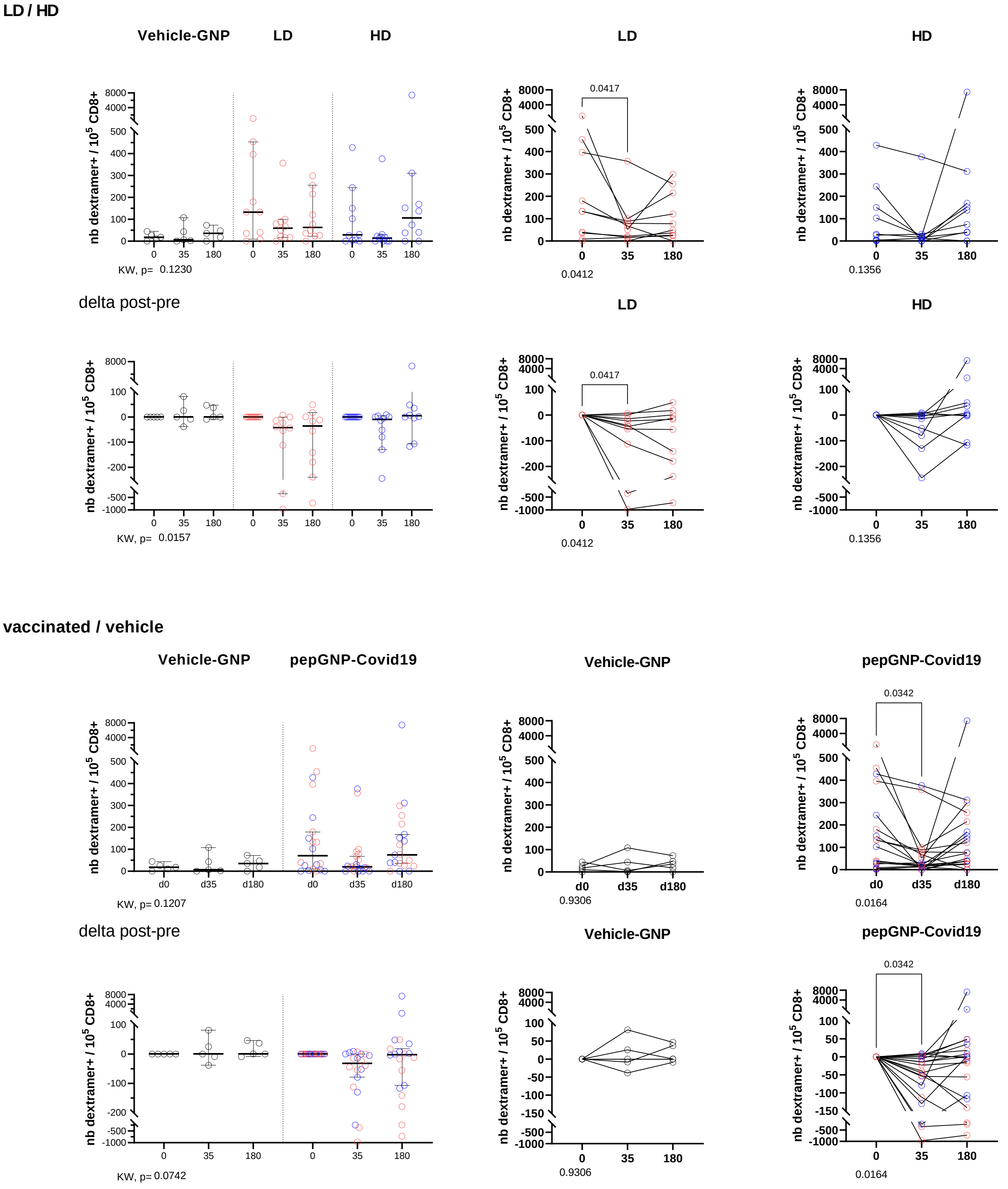

Figure A9. All memory C-dextr+CD8+. Comparisons LD- vs HD-PepGNP-Covid19 groups and PepGNP-Covid19 (vaccinees, n=20) vs Vehicle-GNP (n=5). Tscm, Tcm, Tem and TemRA subsets were summed. Results are expressed as number of C-dextr+ memory CD8+ T cells over 10^5 total CD8+ T cells for each volunteer (n=19). Left panels, inter-group comparison using Kruskal-Wallis tests. Bars indicate medians and 95% CI. Panels on the right, comparison with D0 using Friedman tests and Dunn's post tests; *p* values <0.05 are indicated.

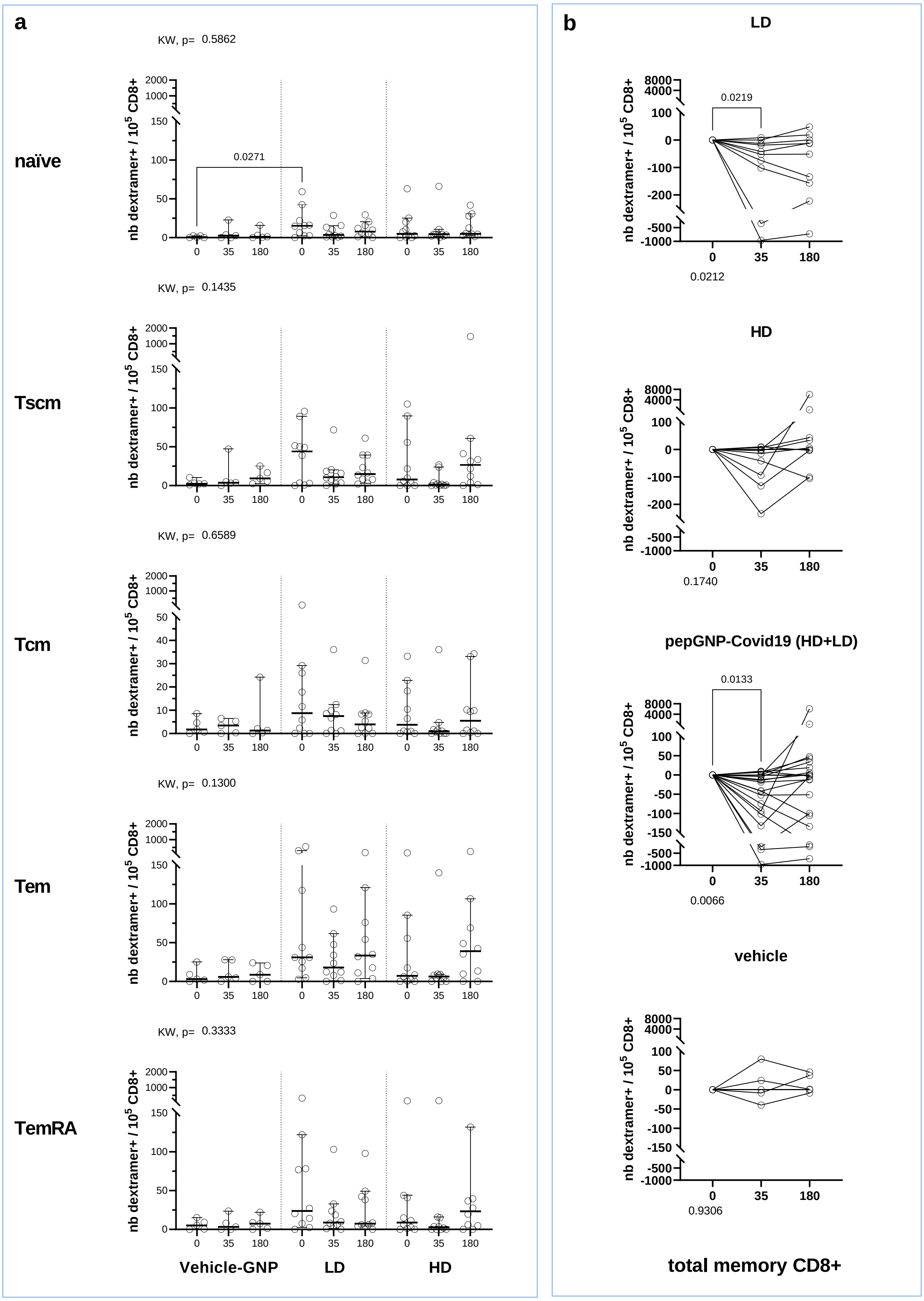
a
naïve
Tscm
Tcm
Tem
TemRA
KW, p= 0.5862
KW, p= 0.1435
KW, p= 0.6589
KW, p= 0.1300
KW, p= 0.3333
0.0271
nb dextramer+ / 10^5 CD8+
0
35
180
Vehicle-GNP
LD
HD
b
LD
0.0219
0.0212
HD
0.1740
pepGNP-Covid19 (HD+LD)
0.0133
0.0066
vehicle
0.9306
total memory CD8+

Figure A10. CXCR3+C-dextr+CD8+ memory subsets. Covid19-specific responses were assessed in volunteers from group Vehicle-GNP (n=5), LD-PepGNP-Covid19 (n=10) and HD-PepGNP-Covid19 (n=10), using HLA class I compatible Covid19 dextramers from a list of eight peptide-fluorochrome combinations. Results are expressed as number of CXCR3+ C-dextramer+CD8+ T cells from various subsets of CD8 + over 10^5 total CD8+ T cells for each volunteer. (a) memory subsets of dextr+CD8. (b) total memory dextr+ CD8 change from baseline (delta post-pre vaccination). CD8+ T cells subsets were defined as naïve (CD45RA+CCR7+CD95-), stem cell memory (Tscm, CD45RA+CCR7+CD95+), central memory (Tcm, CD45RA-CCR7+), effector memory (Tem, CD45RA-CCR7-) and differentiated effector memory (TemRA, CD45RA+CCR7-). Responses to one to four C-dextramers minus negative control were summed per volunteer. Intra-group comparison with d0 using Friedman tests; inter-group comparison using Kruskal-Wallis tests. p values <0.05 are indicated. Bars indicate medians and 95% CI.

Baseline profile

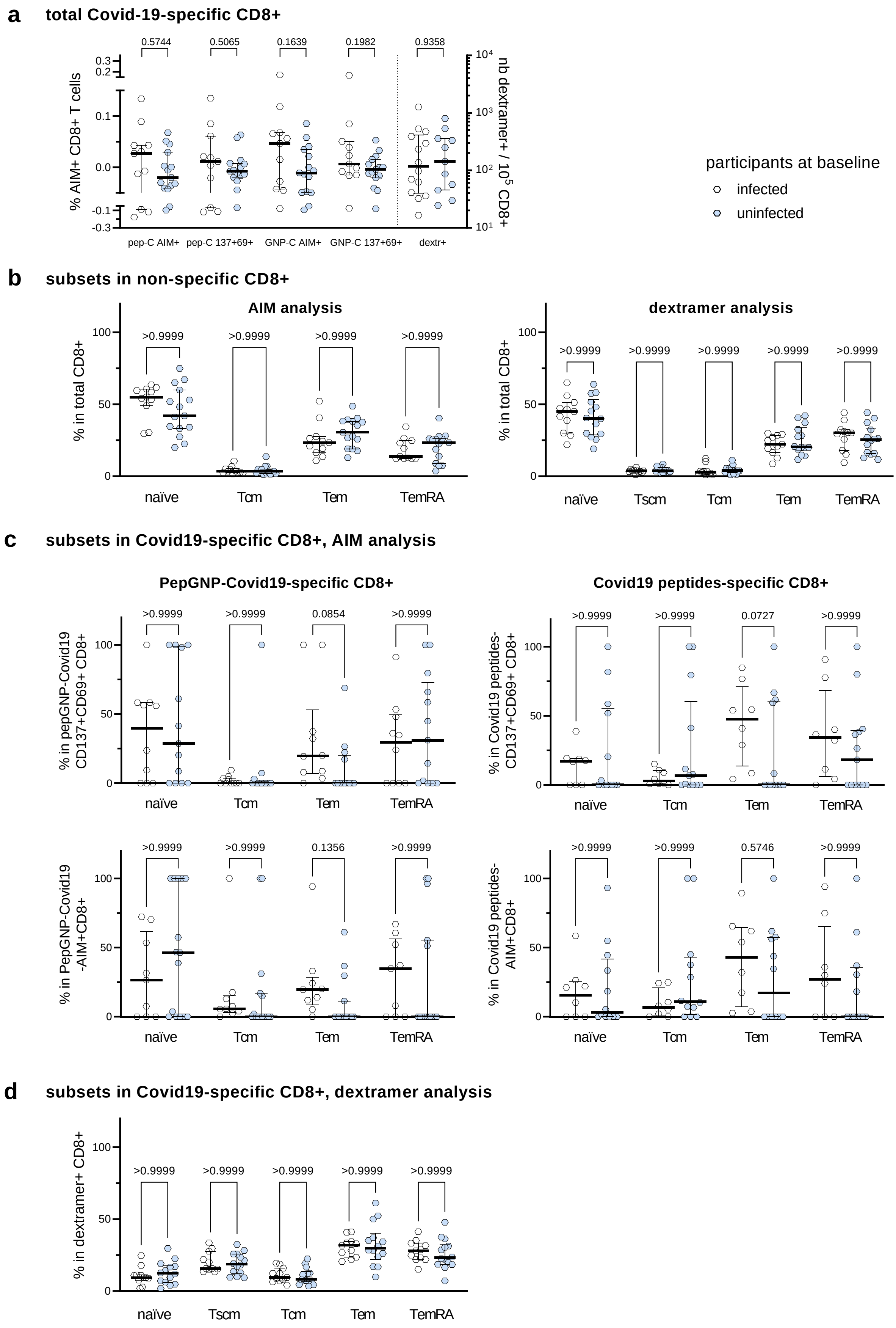

Figure A11. Frequency and profile of Covid19-specific CD8+ T cells in infected and uninfected participants at baseline. Covid19-specific responses were assessed at D0 in volunteers previously Covid19-infected (n=11, white circles) or uninfected (n=15, blue circles) using AIM (panels a, b, c) or C-dextramer (panels a, b and d) to identify Covid19-specific cells. (a) Results expressed as frequency of Covid-specific CD8+. (b) Percentage of subsets over total CD8+, in AIM (unstimulated cells) or during dextramer staining. (c) Percentage of subsets over Covid-specific CD8+ using AIM, CD137+CD69+ or CD107a+CD25+ and/or CD137+CD69+ (AIM+) upon stimulation with PepGNP-Covid19 or Covid19 peptides, stimulated minus control. (d) Percentage of subsets over C-dextramer CD8+, using HLA class I compatible Covid19 dextramers from a list of eight combinations. (b-d) CD8+ T cells subsets were defined as naïve (CD45RA+CCR7+CD95-), stem cell memory (Tscm, CD45RA+CCR7+CD95+), central memory (Tcm, CD45RA-CCR7+), effector memory (Tem, CD45RA-CCR7-) and differentiated effector memory (TemRA, CD45RA+CCR7-). For the profile of response, only volunteers with responses are presented. Inter-group comparison using Mann-Whiney (a) and Kruskal-Wallis tests (b-d). p values are indicated. Bars indicate medians and 95% CI.

Extra figure

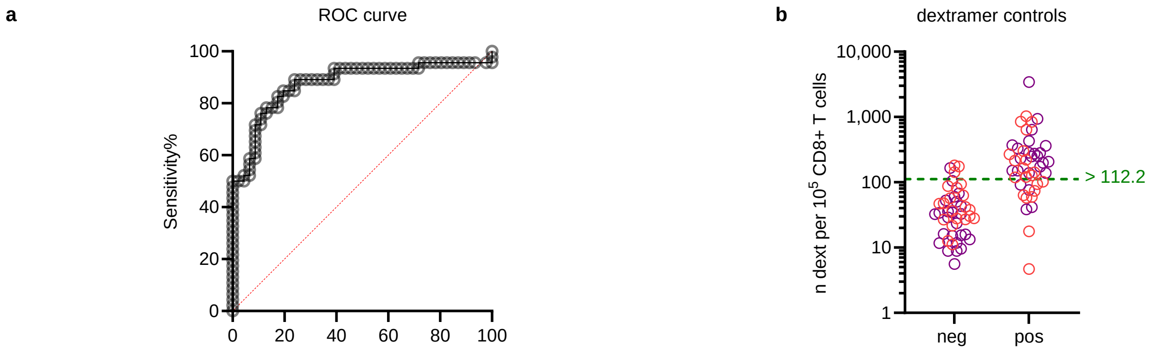

	Sensitivity%	95% CI	Specificity%	95% CI	Likelihood ratio
> 112.2	71.74	57.45% to 82.68%	91.30	79.68% to 96.57%	8.250

Figure A12. Dextramer controls. (a) ROC analysis between negative and positive control dextramers using the frequencies obtained from PepGNP-Covid19 volunteers. (b) Frequencies of negative and positive dextramer control per 10^5 CD8+ T cells, n=46 each, 25 in APC (purple), 21 in PE (red). 112.2: cut-off for positive responses with a high likelihood ratio. Some volunteers showed weak responses to positive dextramers, EBV, CMV or Flu.

Appendix F: References

1. Fenwick C, Turelli P, Perez L, et al. A highly potent antibody effective against SARS-CoV-2 variants of concern. *Cell Rep* 2021; **37**(2): 109814.
2. Poloni C, Schonhofer C, Ivison S, Levings MK, Steiner TS, Cook L. T-cell activation-induced marker assays in health and disease. *Immunol Cell Biol* 2023; **101**(6): 491-503.
3. Takahama S, Nogimori T, Higashiguchi M, Murakami H, Akita H, Yamamoto T. Simultaneous monitoring assay for T-cell receptor stimulation-dependent activation of CD4 and CD8 T cells using inducible markers on the cell surface. *Biochem Biophys Res Commun* 2021; **571**: 53-9.
4. Grifoni A, Weiskopf D, Ramirez SI, et al. Targets of T Cell Responses to SARS-CoV-2 Coronavirus in Humans with COVID-19 Disease and Unexposed Individuals. *Cell* 2020; **181**(7): 1489-501 e15.
5. Bowyer G, Rampling T, Powlson J, et al. Activation-induced Markers Detect Vaccine-Specific CD4(+) T Cell Responses Not Measured by Assays Conventionally Used in Clinical Trials. *Vaccines (Basel)* 2018; **6**(3).
