# Supplementary material for: A gold nanoparticle/peptide vaccine designed to induce SARS-CoV-2-specific CD8 T cells: a double-blind, randomized, phase 1 study in Switzerland
Source: BMC Infect Dis. 2025 Apr 7;25:472. doi: 10.1186/s12879-025-10844-3 (PMC11974014; doi:10.1186/s12879-025-10844-3)
Supplement: Supplementary file 2 — Supplementary Material 2. Data Dictionary Codebook. [file 12879_2025_10844_MOESM2_ESM.pdf]

Data Dictionary Codebook

naNO-COVID - Personal data (PID: 340)

01/10/2024 9:07am

#	Variable / Field Name	Field Label *Field Note*	Field Attributes (Field Type, Validation, Choices, Calculations, etc.)
**Instrument: Personal identification (personal_identification)**			
1	[nano_pi_record_id]	Screening ID	text, Required
2	[nano_pi_id]	Inclusion ID	text<br>Field Annotation: @READONLY
3	[nano_pi_ipp]	IPP	text, Required
4	[nano_pi_firstname]	First Name	text, Required
5	[nano_pi_lastname]	Last Name	text, Required
6	[nano_pi_dob]	Date of birth	text (date_dmy), Required
7	[nano_pi_yearob]	Year of Birth	calc, Required<br>Calculation:<br>rounddown(datediff([nano_pi_dob], "01-01-1901", "y","dmy")) + 1901<br>Field Annotation: @HIDDEN
8	[nano_pi_street_name]	Address (street name)	text, Required
9	[nano_pi_street_number]	Address (number)	text (integer), Required
10	[nano_pi_postal]	Address (postal code)	text (integer), Required
11	[nano_pi_town]	Address (town)	text, Required
12	[nano_pi_phone]	Phone number (Format exemple : 0791234567@sms.unisante)	text (email), Required
13	[nano_pi_email]	E-mail	text (email), Required
14	[nano_pi_doc_name]	General Practitioner<br>*NAME First Name*	text, Required
15	[nano_pi_doc_phone]	Doctor's phone	text, Required
16	[personal_identification_complete]	Section Header: *Form Status*<br>Complete?	dropdown<br>0 Incomplete<br>1 Unverified<br>2 Complete
**Instrument: Informed consent (informed_consent)**			
17	[nano_ic_purpose]	Have you explained the nature and the purpose of the study?	yesno, Required<br>1 Yes<br>0 No

18	[nano_ic_date_delivery]	Date of information sheet delivery	text (datetime_dmy), Required
19	[nano_ic_consent_signed]	Have the informed consent been signed ?	yesno, Required<br>1 Yes<br>0 No
20	[nano_ic_date_signature]	Date of informed consent signature	text (datetime_dmy), Required
21	[nano_ic_stop]<br>Show the field ONLY if:<br>[nano_ic_consent_signed] = '0' OR [nano_ic_purpose] = '0'	Only subjects having been informed and having provided signed consent for participating in the trial and willing to follow all planned trial assessments can be included in the trial.	descriptive
22	[informed_consent_complete]	Section Header: *Form Status*<br>Complete?	dropdown<br>0 Incomplete<br>1 Unverified<br>2 Complete

Data Dictionary Codebook

naNO-COVID - Research data (PID: 341)

01/10/2024 9:09am

#	Variable / Field Name	Field Label<br>*Field Note*	Field Attributes (Field Type, Validation, Choices, Calculations, etc.)
**Instrument: Date of visit (date_of_visit)**			
1	[nano_vi_id]	Screening ID	text, Required
2	[dep_name1]<br>Show the field ONLY if:<br>[event-name] = "screening_arm_1" OR [event-name] = "1st_vaccination_arm_1" OR [event-name] = "2nd_vaccination_arm_1"	Research physician	descriptive
3	[nano_vi_date_visit]	Date of visit	text (date_dmy), Required
4	[date_of_visit_complete]	Section Header: *Form Status*<br>Complete?	dropdown<br>0 Incomplete<br>1 Unverified<br>2 Complete
**Instrument: Volunteer Informations (volunteer_informations)**			
5	[dep_name2]	Research Physician	descriptive
6	[nano_vi_full_id]	Inclusion ID	text<br>Field Annotation: @READONLY
7	[nano_vi_ipp]	IPP	text, Required, Identifier
8	[nano_vi_phone]	Phone	text (email), Required, Identifier<br>Field Annotation: @HIDDEN
9	[nano_vi_yearob]	Section Header: *Demographic informations*<br>Year of birth	text (integer, Min: 1800, Max: 2200), Required
10	[nano_vi_gender]	Gender	radio, Required<br>1 Male<br>2 Female
11	[nano_vi_ethnicity]	Ethnicity	radio, Required<br>1 Caucasian<br>2 Black<br>3 Latino<br>4 Asian<br>5 Pacific Islander<br>6 Other
12	[nano_vi_ethnicity_specify]<br>Show the field ONLY if:<br>[nano_vi_ethnicity]='6'	Specify	text, Required
13	[nano_vi_smoke]	Section Header: *Habits*	yesno, Required

		Does the volunteer smoke or use tobacco products?	1 Yes<br>0 No
14	[nano_vi_years_smoke]<br>Show the field ONLY if:<br>[nano_vi_smoke] = '1'	How many years of smoking ?	text (number, Min: 0, Max: 100), Required
15	[nano_vi_cig_per_day]<br>Show the field ONLY if:<br>[nano_vi_smoke] = '1'	How many cigarettes per day ?	text (integer, Min: 0, Max: 100), Required
16	[nano_vi_alcohol]	Does the volunteer consum alcohol?	yesno, Required<br>1 Yes<br>0 No
17	[nano_vi_drink_per_week]<br>Show the field ONLY if:<br>[nano_vi_alcohol] = '1'	How many standard alcoholic drinks per week ?	text (integer, Min: 0, Max: 100), Required
18	[nano_vi_drug_consume]	Has the volunteer used drugs (like opiates, cocaine or amphetamin) in the last 5 years ?	yesno, Required<br>1 Yes<br>0 No
19	[nano_vi_drugs_spec]<br>Show the field ONLY if:<br>[nano_vi_drug_consume] = '1'	Please specify drug(s)	text, Required
20	[nano_vi_med_problem]	Section Header: *Medical history*<br>Has the volunteer any medical problem ?	yesno, Required<br>1 Yes<br>0 No
21	[nano_vi_med_condition]	Relevant medical condition	text
22	[nano_vi_med_condition_1]	Relevant medical condition	text
23	[nano_vi_med_condition_2]	Relevant medical condition	text
24	[nano_vi_med_condition_3]	Relevant medical condition	text
25	[nano_vi_med_condition_4]	Relevant medical condition	text
26	[nano_vi_med_condition_5]	Relevant medical condition	text
27	[nano_vi_med_condition_6]	Relevant medical condition	text
28	[nano_vi_table]<br>Show the field ONLY if:	relevant medical condition day month year<br>{nano_vi_med_condition}<br>{nano_vi_med_condition_day}<br>{nano_vi_med_condition_month}	descriptive

	[nano_vi_med_problem] = '1'	{nano_vi_med_condition_year}<br>{nano_vi_med_condition_1}<br>{nano_vi_med_condition_day_1}<br>{nano_vi_med_condition_month_1}<br>{nano_vi_med_condition_year_1}<br>{nano_vi_med_condition_2}<br>{nano_vi_med_condition_day_2}<br>{nano_vi_med_condition_month_2}<br>{nano_vi_med_condition_year_2}<br>{nano_vi_med_condition_3}<br>{nano_vi_med_condition_day_3}<br>{nano_vi_med_condition_month_3}<br>{nano_vi_med_condition_year_3}<br>{nano_vi_med_condition_4}<br>{nano_vi_med_condition_day_4}<br>{nano_vi_med_condition_month_4}<br>{nano_vi_med_condition_year_4}<br>{nano_vi_med_condition_5}<br>{nano_vi_med_condition_day_5}<br>{nano_vi_med_condition_month_5}<br>{nano_vi_med_condition_year_5}<br>{nano_vi_med_condition_6}<br>{nano_vi_med_condition_day_6}<br>{nano_vi_med_condition_month_6}<br>{nano_vi_med_condition_year_6}	
29	[nano_vi_table_2]<br>Show the field ONLY if:<br>[nano_vi_med_problem] = '1'	relevant past medical history day month year<br>{nano_vi_past_med_his}<br>{nano_vi_past_med_his_day}<br>{nano_vi_past_med_his_month}<br>{nano_vi_past_med_his_year}<br>{nano_vi_past_med_his_1}<br>{nano_vi_past_med_his_day_1}<br>{nano_vi_past_med_his_month_1}<br>{nano_vi_past_med_his_year_1}<br>{nano_vi_past_med_his_2}<br>{nano_vi_past_med_his_day_2}<br>{nano_vi_past_med_his_month_2}<br>{nano_vi_past_med_his_year_2}<br>{nano_vi_past_med_his_3}<br>{nano_vi_past_med_his_day_3}<br>{nano_vi_past_med_his_month_3}<br>{nano_vi_past_med_his_year_3}<br>{nano_vi_past_med_his_4}<br>{nano_vi_past_med_his_day_4}<br>{nano_vi_past_med_his_month_4}<br>{nano_vi_past_med_his_year_4}<br>{nano_vi_past_med_his_5}<br>{nano_vi_past_med_his_day_5}<br>{nano_vi_past_med_his_month_5}<br>{nano_vi_past_med_his_year_5}<br>{nano_vi_past_med_his_6}<br>{nano_vi_past_med_his_day_6}<br>{nano_vi_past_med_his_month_6}<br>{nano_vi_past_med_his_year_6}	descriptive
30	[nano_vi_med_condition_day]	day	text (integer, Min: 0, Max: 31)
31	[nano_vi_med_condition_month]	month	text (integer, Min: 0, Max: 12)
32	[nano_vi_med_condition_year]	year	text (integer, Min: 0, Max: 2050)

33	[nano_vi_med_condition_day_1]	day	text (integer, Min: 0, Max: 31)
34	[nano_vi_med_condition_month_1]	month	text (integer, Min: 0, Max: 12)
35	[nano_vi_med_condition_year_1]	year	text (integer, Min: 0, Max: 2050)
36	[nano_vi_med_condition_day_2]	day	text (integer, Min: 0, Max: 31)
37	[nano_vi_med_condition_month_2]	month	text (integer, Min: 0, Max: 12)
38	[nano_vi_med_condition_year_2]	year	text (integer, Min: 0, Max: 2050)
39	[nano_vi_med_condition_day_3]	day	text (integer, Min: 0, Max: 31)
40	[nano_vi_med_condition_month_3]	month	text (integer, Min: 0, Max: 12)
41	[nano_vi_med_condition_year_3]	year	text (integer, Min: 0, Max: 2050)
42	[nano_vi_med_condition_day_4]	day	text (integer, Min: 0, Max: 31)
43	[nano_vi_med_condition_month_4]	month	text (integer, Min: 0, Max: 12)
44	[nano_vi_med_condition_year_4]	year	text (integer, Min: 0, Max: 2050)
45	[nano_vi_med_condition_day_5]	day	text (integer, Min: 0, Max: 31)
46	[nano_vi_med_condition_month_5]	month	text (integer, Min: 0, Max: 12)
47	[nano_vi_med_condition_year_5]	year	text (integer, Min: 0, Max: 2050)
48	[nano_vi_med_condition_day_6]	day	text (integer, Min: 0, Max: 31)
49	[nano_vi_med_condition_month_6]	month	text (integer, Min: 0, Max: 12)
50	[nano_vi_med_condition_year_6]	year	text (integer, Min: 0, Max: 2050)
51	[nano_vi_past_med_his]	Relevant past medical history	text
52	[nano_vi_past_med_his_1]	Relevant past medical history	text
53	[nano_vi_past_med_his_2]	Relevant past medical history	text
54	[nano_vi_past_med_his_3]	Relevant past medical history	text
55	[nano_vi_past_med_his_4]	Relevant past medical history	text
56	[nano_vi_past_med_his_5]	Relevant past medical history	text
57	[nano_vi_past_med_his_6]	Relevant past medical history	text

58	[nano_vi_past_med_his_day]	day	text (integer, Min: 0, Max: 31)
59	[nano_vi_past_med_his_month]	month	text (integer, Min: 0, Max: 12)
60	[nano_vi_past_med_his_year]	year	text (integer, Min: 1920, Max: 2050)
61	[nano_vi_past_med_his_day_1]	day	text (integer, Min: 0, Max: 31)
62	[nano_vi_past_med_his_month_1]	month	text (integer, Min: 0, Max: 12)
63	[nano_vi_past_med_his_year_1]	year	text (integer, Min: 1920, Max: 2050)
64	[nano_vi_past_med_his_day_2]	day	text (integer, Min: 0, Max: 31)
65	[nano_vi_past_med_his_month_2]	month	text (integer, Min: 0, Max: 12)
66	[nano_vi_past_med_his_year_2]	year	text (integer, Min: 1920, Max: 2050)
67	[nano_vi_past_med_his_day_3]	day	text (integer, Min: 0, Max: 31)
68	[nano_vi_past_med_his_month_3]	month	text (integer, Min: 0, Max: 12)
69	[nano_vi_past_med_his_year_3]	year	text (integer, Min: 1920, Max: 2050)
70	[nano_vi_past_med_his_day_4]	day	text (integer, Min: 0, Max: 31)
71	[nano_vi_past_med_his_month_4]	month	text (integer, Min: 0, Max: 12)
72	[nano_vi_past_med_his_year_4]	year	text (integer, Min: 1920, Max: 2050)
73	[nano_vi_past_med_his_day_5]	day	text (integer, Min: 0, Max: 31)
74	[nano_vi_past_med_his_month_5]	month	text (integer, Min: 0, Max: 12)
75	[nano_vi_past_med_his_year_5]	year	text (integer, Min: 1920, Max: 2050)
76	[nano_vi_past_med_his_day_6]	day	text (integer, Min: 0, Max: 31)
77	[nano_vi_past_med_his_month_6]	month	text (integer, Min: 0, Max: 12)
78	[nano_vi_past_med_his_year_6]	year	text (integer, Min: 1920, Max: 2050)
79	[nano_vi_allergies]	Allergie(s) ?	yesno, Required<br>1 Yes<br>0 No
80	[nano_vi_allergies_specify]<br>Show the field ONLY if:<br>[nano_vi_allergies] = '1'	Please specify allergie(s)	text, Required

81	[nano_vi_past_covid]	Section Header: *COVID-19 history (natural disease and vaccination)*<br><br>Past COVID-19 history	yesno<br>1 Yes<br>0 No
82	[nano_vi_past_covid_no_ep]<br><br>Show the field ONLY if:<br>[nano_vi_past_covid] = "1"	Number of episodes	radio<br>1 1<br>2 2<br>3 3
83	[nano_vi_which_test]<br><br>Show the field ONLY if:<br>[nano_vi_past_covid_no_ep] = '1' OR [nano_vi_past_covid_no_ep] = '2' OR [nano_vi_past_covid_no_ep] = '3'	Which test has been performed ? (1st episode)	radio<br>1 Serologic test<br>2 Antigenic test<br>3 PCR test
84	[nano_vi_covid_test_date]<br><br>Show the field ONLY if:<br>[nano_vi_past_covid_no_ep] = '1' OR [nano_vi_past_covid_no_ep] = '2' OR [nano_vi_past_covid_no_ep] = '3'	Date of the test (1st episode)	text (date_dmy)
85	[nano_vi_which_test_2]<br><br>Show the field ONLY if:<br>[nano_vi_past_covid_no_ep] = '2' OR [nano_vi_past_covid_no_ep] = '3'	Which test has been performed ? (2nd episode)	radio<br>1 Serologic test<br>2 Antigenic test<br>3 PCR test
86	[nano_vi_covid_test_date_2]<br><br>Show the field ONLY if:<br>[nano_vi_past_covid_no_ep] = '2' OR [nano_vi_past_covid_no_ep] = '3'	Date of the test (2nd episode)	text (date_dmy)
87	[nano_vi_which_test_3]<br><br>Show the field ONLY if:<br>[nano_vi_past_covid_no_ep] = '3'	Which test has been performed ? (3rd episode)	radio<br>1 Serologic test<br>2 Antigenic test<br>3 PCR test
88	[nano_vi_covid_test_date_3]<br><br>Show the field ONLY if:<br>[nano_vi_past_covid_no_ep] = '3'	Date of the test (3rd episode)	text (date_dmy)

89	[nano_vi_covid_vacc]	COVID-19 vaccination	yesno<br>1 Yes<br>0 No
90	[nano_vi_dose_number]<br>Show the field ONLY if:<br>[nano_vi_covid_vacc] = "1"	Number of doses	radio<br>1 1<br>2 2<br>3 3<br>4 4
91	[nano_vi_date_dose]<br>Show the field ONLY if:<br>[nano_vi_dose_number] > 0	Date dose 1	text (date_dmy)
92	[nano_vi_dose_brand]<br>Show the field ONLY if:<br>[nano_vi_dose_number] > 0	Brand of dose 1	radio<br>1 Pfizer<br>2 Moderna<br>3 AZ<br>4 Janssen<br>5 Sinovac<br>6 Sinopharm<br>7 Sputnik
93	[nano_vi_date_dose_2]<br>Show the field ONLY if:<br>[nano_vi_dose_number] > 1	Date dose 2	text (date_dmy)
94	[nano_vi_dose_brand_2]<br>Show the field ONLY if:<br>[nano_vi_dose_number] > 1	Brand of dose 2	radio<br>1 Pfizer<br>2 Moderna<br>3 AZ<br>4 Janssen<br>5 Sinovac<br>6 Sinopharm<br>7 Sputnik
95	[nano_vi_date_dose_3]<br>Show the field ONLY if:<br>[nano_vi_dose_number] > 2	Date dose 3	text (date_dmy)
96	[nano_vi_dose_brand_3]<br>Show the field ONLY if:	Brand of dose 3	radio<br>1 Pfizer<br>2 Moderna<br>3 AZ

	[nano_vi_dose_number] > 2		4 Janssen<br>5 Sinovac<br>6 Sinopharm<br>7 Sputnik
97	[nano_vi_date_dose_4]<br>Show the field ONLY if:<br>[nano_vi_dose_number] > 3	Date dose 4	text (date_dmy)
98	[nano_vi_dose_brand_4]<br>Show the field ONLY if:<br>[nano_vi_dose_number] > 3	Brand of dose 4	radio<br>1 Pfizer<br>2 Moderna<br>3 AZ<br>4 Janssen<br>5 Sinovac<br>6 Sinopharm<br>7 Sputnik
99	[volunteer_informations_complete]	Section Header: *Form Status*<br>Complete?	dropdown<br>0 Incomplete<br>1 Unverified<br>2 Complete
**Instrument: Informed Consent (informed_consent)**			
100	[dep_name3]	Research physician	descriptive
101	[nano_ic_purpose]	Have you explained the nature and the purpose of the study?	yesno, Required<br>1 Yes<br>0 No
102	[nano_ic_date_delivery]	Date of information sheet delivery	text (datetime_dmy), Required
103	[nano_ic_consent_signed]	Have the informed consent been signed ?	yesno, Required<br>1 Yes<br>0 No
104	[nano_ic_date_signature]	Date of informed consent signature	text (datetime_dmy), Required
105	[nano_ic_stop]<br>Show the field ONLY if:<br>[nano_ic_consent_signed] = '0' OR [nano_ic_purpose] = '0'	Only subjects having been informed and having provided signed consent for participating in the trial and willing to follow all planned trial assessments can be included in the trial.	descriptive
106	[informed_consent_complete]	Section Header: *Form Status*<br>Complete?	dropdown<br>0 Incomplete<br>1 Unverified<br>2 Complete

Instrument: Medical History (medical_history)

107	[dep_name4]<br>Show the field ONLY if:<br>[event-name] = "screening_arm_1" OR [event-name] = "1st_vaccination_arm_1" OR [event-name] = "2nd_vaccination_arm_1"	Research Physician	descriptive
108	[nano_mh_consent_signed]<br>Show the field ONLY if:<br>[event-name] = 'screening_arm_1'	Section Header: *Verifying informations*<br>Have the informed consent been signed ?	yesno, Required<br>1 Yes<br>0 No
109	[nano_mh_consent_changed]<br>Show the field ONLY if:<br>[event-name] = '1st_vaccination_arm_1'	Changes in informed consent ?	yesno, Required<br>1 Yes<br>0 No
110	[nano_mh_consent_changed_message]<br>Show the field ONLY if:<br>[nano_mh_consent_changed] = '1'	Only subjects having been informed and having provided signed consent for participating in the trial and willing to follow all planned trial assessments can be included in the trial. If consent is withdraw, please complete the SCREEN FAILURE page.	descriptive
111	[nano_mh_abnormalities]	Abnormalities/new medical event reported since the last visit?	yesno, Required<br>1 Yes<br>0 No
112	[nano_mh_abnormalities_spec]<br>Show the field ONLY if:<br>[nano_mh_abnormalities] = '1'	Specify	notes, Required
113	[nano_mh_medication_changed]<br>Show the field ONLY if:<br>[event-name] <> 'visit_10_phone_arm_1' and [event-name] <> 'visit_11_arm_1' and [event-name] <> 'visit_12_arm_1'	Has medication changed since the last visit?	yesno, Required<br>1 Yes<br>0 No
114	[nano_mh_fill_med]<br>Show the field ONLY if:<br>[nano_mh_medication_changed] = '1'	Please fill the medication form	descriptive
115	[nano_mh_new_vac]	Any vaccination received since the last visit?	yesno, Required

	Show the field ONLY if:<br>[event-name] <> 'visit_10_phone_arm_1' and [event-name] <> 'visit_11_arm_1' and [event-name] <> 'visit_12_arm_1'		1 Yes<br>0 No
116	[nano_mh_date_vac]<br>Show the field ONLY if:<br>[nano_mh_new_vac] = '1'	Date of vaccination	text (date_dmy), Required
117	[nano_mh_postpone]<br>Show the field ONLY if:<br>[nano_mh_new_vac] = '1'	If the subject has been vaccinated within 14 days (influenza vaccine) or within 28 days (other vaccines) prior to Visit 2, the subject cannot be vaccinated. Please consider postponement of vaccination or complete the SCREEN FAILURE page	descriptive
118	[medical_history_complete]	Section Header: *Form Status*<br>Complete?	dropdown<br>0 Incomplete<br>1 Unverified<br>2 Complete
**Instrument: Adverse Events (adverse_events)**			
119	[dep_name5]<br>Show the field ONLY if:<br>[event-name] = "screening_arm_1" OR [event-name] = "1st_vaccination_arm_1" OR [event-name] = "2nd_vaccination_arm_1"	Study nurse	descriptive
120	[nano_ae_date_visit]	Date of visit	text (datetime_dmy), Required
121	[nano_ae_list]	Section Header: *Updating reports*<br>List of current AE reports to be updated	descriptive
122	[nano_sae_list]	List of current SAE reports to be updated	descriptive
123	[nano_ae_new_ae]	Section Header: *Serious Adverse event*<br>Is there any serious adverse event (SAE) since last visit?	yesno, Required<br>1 Yes<br>0 No
124	[nano_sae_definition]		descriptive<br>(Attachment: Definition_Serious Adverse Event.pdf, Display format: Link)
125	[nano_sae_button]<br>Show the field ONLY if:<br>[nano_ae_new_ae] = '1'	Please fill a SAE form	descriptive
126	[nano_sae_new_pain]<br>Show the field ONLY if:	Section Header: *Solicited adverse event at injection site*<br>New pain	yesno, Required<br>1 Yes<br>0 No

	[event-name] = "1st_vaccination_arm_1" OR [event-name] = "visit_3_phone_arm_1" OR [event-name] = "visit_7_phone_arm_1" OR [event-name] = "visit_8_arm_1" or [event-name] = "visit_4_arm_1"		
127	[nano_sae_new_tenderness]<br>Show the field ONLY if:<br>[event-name] = "1st_vaccination_arm_1" OR [event-name] = "visit_3_phone_arm_1" OR [event-name] = "visit_7_phone_arm_1" OR [event-name] = "visit_8_arm_1" or [event-name] = "visit_4_arm_1"	New tenderness	yesno, Required<br>1 Yes<br>0 No
128	[nano_sae_new_erythema]<br>Show the field ONLY if:<br>[event-name] = "1st_vaccination_arm_1" OR [event-name] = "visit_3_phone_arm_1" OR [event-name] = "visit_7_phone_arm_1" OR [event-name] = "visit_8_arm_1" or [event-name] = "visit_4_arm_1"	New erythema	yesno, Required<br>1 Yes<br>0 No
129	[nano_sae_new_swelling]<br>Show the field ONLY if:<br>[event-name] = "1st_vaccination_arm_1" OR [event-name] = "visit_3_phone_arm_1" OR [event-name] = "visit_7_phone_arm_1" OR [event-name] = "visit_8_arm_1" or [event-name] = "visit_4_arm_1"	New swelling	yesno, Required<br>1 Yes<br>0 No
130	[nano_sae_button_2]<br>Show the field ONLY if:<br>[nano_sae_new_pain] = '1' or [nano_sae_new_tenderness] = '1' or [nano_sae_new_erythema] = '1' or [nano_sae_new_swelling] = '1'	Please fill a SAE form - Please fill a AE form	descriptive

131	[nano_sae_unsolicited_ae_inj]<br>Show the field ONLY if:<br>[event-name] <> 'visit_11_arm_1' and [event-name] <> 'visit_12_arm_1' and [event-name] <> 'visit_10_phone_arm_1'	Section Header: *Unsolicited adverse event at injection site*<br>Is there any new unsolicited adverse event at injection site?	yesno, Required<br>1 Yes<br>0 No
132	[nano_sae_button_3]<br>Show the field ONLY if:<br>[nano_sae_unsolicited_ae_inj] = '1'	Please fill a SAE form - Please fill a AE form	descriptive
133	[nano_sae_new_headache]<br>Show the field ONLY if:<br>[event-name] <> 'visit_11_arm_1' and [event-name] <> 'visit_12_arm_1' and [event-name] <> 'visit_10_phone_arm_1' and [event-name] <> '2nd_vaccination_arm_1'	Section Header: *Solicited systemic adverse event*<br>New headache	yesno, Required<br>1 Yes<br>0 No
134	[nano_sae_new_malaise]<br>Show the field ONLY if:<br>[event-name] <> 'visit_11_arm_1' and [event-name] <> 'visit_12_arm_1' and [event-name] <> 'visit_10_phone_arm_1' and [event-name] <> '2nd_vaccination_arm_1'	New nausea/vomiting	yesno, Required<br>1 Yes<br>0 No
135	[nano_sae_new_diarrhea]<br>Show the field ONLY if:<br>[event-name] <> 'visit_11_arm_1' and [event-name] <> 'visit_12_arm_1' and [event-name] <> 'visit_10_phone_arm_1' and [event-name] <> '2nd_vaccination_arm_1'	New diarrhea	yesno<br>1 Yes<br>0 No
136	[nano_sae_new_myalgia]<br>Show the field ONLY if:<br>[event-name] <> 'visit_11_arm_1' and [event-	New Myalgia	yesno, Required<br>1 Yes<br>0 No

	name] <> 'visit_12_arm_1' and [event-name] <> 'visit_10_phone_arm_1' and [event-name] <> '2nd_vaccination_arm_1'		
137	[nano_sae_new_asthenia]<br>Show the field ONLY if:<br>[event-name] <> 'visit_11_arm_1' and [event-name] <> 'visit_12_arm_1' and [event-name] <> 'visit_10_phone_arm_1' and [event-name] <> '2nd_vaccination_arm_1'	New Fatigue	yesno, Required<br>1 Yes<br>0 No
138	[nano_sae_new_temp]<br>Show the field ONLY if:<br>[event-name] <> 'visit_11_arm_1' and [event-name] <> 'visit_12_arm_1' and [event-name] <> 'visit_10_phone_arm_1' and [event-name] <> '2nd_vaccination_arm_1'	New Fever	yesno, Required<br>1 Yes<br>0 No
139	[nano_sae_new_fever]<br>Show the field ONLY if:<br>[nano_sae_new_temp] = "1"	Axillary temperature	text (number_1dp, Min: 33, Max: 43), Required
140	[nano_sae_button_4]<br>Show the field ONLY if:<br>[nano_sae_new_temp] = '1' or [nano_sae_new_headache] = '1' or [nano_sae_new_malaise] = '1' or [nano_sae_new_myalgia] = '1' or [nano_sae_new_asthenia] = '1' or [nano_sae_new_temp] = '1'	Please fill a SAE form - Please fill a AE form	descriptive
141	[nano_sae_unsolicited_sys_ae]<br>Show the field ONLY if:<br>[event-name] <> 'visit_11_arm_1' and [event-name] <> 'visit_12_arm_1' and [event-name] <> 'visit_10_phone_arm_1'	Section Header: *Unsolicited systemic adverse event*<br>Is there any new unsolicited systemic adverse event?	yesno, Required<br>1 Yes<br>0 No
142	[nano_sae_button_5]	Please fill a SAE form - Please fill a AE form	descriptive

	Show the field ONLY if: [nano_sae_unsolicited_sys_ae] = "1"		
143	[nano_sae_unsolicited_ae] Show the field ONLY if: [event-name] = "visit_10_phone_arm_1" OR [event-name] = "visit_11_arm_1" OR [event-name] = "visit_12_arm_1"	Section Header: *Unsolicited adverse event* Is there any new unsolicited adverse event ?	yesno<br>1 Yes<br>0 No
144	[nano_sae_button_9] Show the field ONLY if: [nano_sae_unsolicited_ae] = "1"	Please fill a SAE form - Please fill a AE form	descriptive
145	[adverse_events_complete]	Section Header: *Form Status* Complete?	dropdown<br>0 Incomplete<br>1 Unverified<br>2 Complete
**Instrument: Current medication (current_medication)**			
146	[dep_name6] Show the field ONLY if: [event-name] = "screening_arm_1" OR [event-name] = "1st_vaccination_arm_1" OR [event-name] = "2nd_vaccination_arm_1"	Research Physician	descriptive
147	[nano_cm_curr_medication] Show the field ONLY if: [event-name] = "screening_arm_1"	Is the volunteer currently taking medication (prescribed medication, over-the-counter drugs, complementary medicinal products) ?	yesno, Required<br>1 Yes<br>0 No
148	[nano_cm_fill_med] Show the field ONLY if: [nano_cm_curr_medication] = "1"	Please fill the medication form	descriptive
149	[nano_cm_contraceptive] Show the field ONLY if: [event-name] = "screening_arm_1" and [screening_arm_1][nano_vi_gender] = '2'	Has the volunteer used a continuous effective contraception since 4 weeks prior to screening)?	yesno, Required<br>1 Yes<br>0 No
150	[nano_cm_contraceptive_spec]	Please specify	radio, Required

	Show the field ONLY if: [nano_cm_contraceptive] = '1'		1 Postmenopausal since at least 1 year<br>2 Surgically sterile (female or male partner)<br>3 Use of oral, injected or implanted hormonal contraception<br>4 Placement of an intrauterine device or intrauterine system<br>5 Barrier method of contraception (condom or occlusive cap with spermicide)<br>6 True abstinence
151	[nano_cm_fill_med_2]<br>Show the field ONLY if: [nano_cm_contraceptive_spec] = "3" OR [nano_cm_contraceptive_spec] = "4"	Please fill the medication form	descriptive
152	[nano_cm_fill_med_3]<br>Show the field ONLY if: [nano_cm_contraceptive] = '0'	Please either consider postponement of vaccination if subject is willing to start a continuous effective contraception. Otherwise,complete the SCREEN FAILURE page	descriptive
153	[nano_sae_change_medication]<br>Show the field ONLY if: [event-name] <> "screening_arm_1"	Is there any new or changed medication since the last visit?	yesno, Required<br>1 Yes<br>0 No
154	[nano_sae_button_6]<br>Show the field ONLY if: [nano_sae_change_medication] = "1"	Please fill the medication form	descriptive
155	[nano_sae_vaccination]<br>Show the field ONLY if: [event-name] <> "screening_arm_1"	Is there any vaccination since the last visit?	yesno, Required<br>1 Yes<br>0 No
156	[nano_sae_vaccination_name]<br>Show the field ONLY if: [nano_sae_vaccination] = '1'	Specify vaccine name	text, Required
157	[nano_sae_vaccination_date]<br>Show the field ONLY if:	Date of vaccination	text (date_dmy), Required

	[nano_sae_vaccination] = '1'		
158	[current_medication_complete]	Section Header: *Form Status*<br>Complete?	dropdown<br>0 Incomplete<br>1 Unverified<br>2 Complete
**Instrument: Physical observations (physical_observations)**			
159	[dep_name7]<br>Show the field ONLY if:<br>[event-name] = "screening_arm_1" OR [event-name] = "1st_vaccination_arm_1" OR [event-name] = "2nd_vaccination_arm_1"	Research Physician	descriptive
160	[nano_po_physical_exam_done]<br>Show the field ONLY if:<br>[event-name] <> '2nd_vaccination_arm_1' and [event-name] <> "screening_arm_1" and [event-name] <> '1st_vaccination_arm_1'	Has a physical examination been done?	yesno, Required<br>1 Yes<br>0 No
161	[nano_po_physical_exam_done_why]<br>Show the field ONLY if:<br>([nano_po_physical_exam_done] = "0" and [event-name] <> "visit_3_phone_arm_1") or ([event-name] = "visit_3_phone_arm_1" and [nano_po_physical_exam_done] = "1")	Specify why	text, Required
162	[nano_po_height]<br>Show the field ONLY if:<br>[event-name] = 'screening_arm_1'	Body height<br>*cm*	text (integer, Min: 0, Max: 220), Required
163	[nano_po_weight]<br>Show the field ONLY if:<br>([event-name] = 'screening_arm_1' and [event-name] <> '2nd_vaccination_arm_1') or ([nano_po_physical_exam_done] = '1' and [event-name] <> '2nd_vaccination_arm_1')	Body weight<br>*kg*	text (number, Min: 0, Max: 150), Required

164	[nano_po_bmi]<br>Show the field ONLY if:<br>[event-name] = 'screening_arm_1'	BMI<br>*kg/m2*	calc<br>Calculation:<br>[nano_po_weight]/(([nano_po_height]/100)*([nano_po_height]/100))
165	[nano_po_temperature]<br>Show the field ONLY if:<br>([nano_po_physical_exam_done] = "1" or [event-name] = "2nd_vaccination_arm_1") or ([event-name] = "screening_arm_1") or ([event-name] = "1st_vaccination_arm_1")	Axillary temperature<br>*°C*	text (number, Min: 00, Max: 42), Required
166	[nano_po_sys_blood_press]<br>Show the field ONLY if:<br>([nano_po_physical_exam_done] = "1" or [event-name] = "2nd_vaccination_arm_1") or ([event-name] = "screening_arm_1") or ([event-name] = "1st_vaccination_arm_1")	Systolic blood pressure<br>*mmHg*	text (integer, Min: 00, Max: 230), Required
167	[nano_po_dia_blood_pressure]<br>Show the field ONLY if:<br>([nano_po_physical_exam_done] = "1" or [event-name] = "2nd_vaccination_arm_1") or ([event-name] = "screening_arm_1") or ([event-name] = "1st_vaccination_arm_1")	Diastolic blood pressure<br>*mmHg*	text (integer, Min: 00, Max: 150), Required
168	[nano_po_heart_rate]<br>Show the field ONLY if:<br>([nano_po_physical_exam_done] = "1" or [event-name] = "2nd_vaccination_arm_1") or ([event-name] = "screening_arm_1") or ([event-name] = "1st_vaccination_arm_1")	Heart rate<br>*bpm*	text (integer, Min: 00, Max: 150), Required
169	[dep_name16]<br>Show the field ONLY if:<br>[event-name] = "screening_arm_1" OR [event	Section Header: *Physical Examination*<br>Research Physician	descriptive

	-name] = "1st_vaccination_arm_1" OR [event-name] = "2nd_vaccination_arm_1"		
170	[nano_pe_cardiovascular] Show the field ONLY if: [nano_po_physical_exam_done] = '1' or [event-name] = 'screening_arm_1' or [event-name] = '2nd_vaccination_arm_1' or [event-name] = '1st_vaccination_arm_1'	Cardiovascular	notes, Required
171	[nano_pe_respiratory] Show the field ONLY if: [nano_po_physical_exam_done] = '1' or [event-name] = 'screening_arm_1' or [event-name] = '2nd_vaccination_arm_1' or [event-name] = '1st_vaccination_arm_1'	Respiratory	notes, Required
172	[nano_pe_gastro_intestinal] Show the field ONLY if: [nano_po_physical_exam_done] = '1' or [event-name] = 'screening_arm_1' or [event-name] = '2nd_vaccination_arm_1' or [event-name] = '1st_vaccination_arm_1'	Gastro-intestinal	notes, Required
173	[nano_pe_dermatological] Show the field ONLY if: [nano_po_physical_exam_done] = '1' or [event-name] = 'screening_arm_1' or [event-name] = '2nd_vaccination_arm_1' or [event-name] = '1st_vaccination_arm_1'	Dermatological	notes, Required
174	[nano_pe_specify] Show the field ONLY if: [nano_po_physical_exam_done] = '1' or [event	Other(s) : Specify	notes, Required

	-name] = 'screening_arm_1' or [event-name] = '2nd_vaccination_arm_1' or [event-name] = '1st_vaccination_arm_1'		
175	[physical_observations_complete]	Section Header: *Form Status*<br>Complete?	dropdown<br>0 Incomplete<br>1 Unverified<br>2 Complete
**Instrument: Laboratory analyses (laboratory_analyses)**			
176	[dep_name18]	Study nurse	descriptive
177	[nano_la_blood_sample_date_collect]	Section Header: *Samples*<br>Blood Sampling Date and Time	text (datetime_dmy), Required
178	[nano_la_blood_sample_routine]<br>Show the field ONLY if:<br>[event-name] = 'screening_arm_1' OR [event-name] = 'visit_4_arm_1' OR [event-name] = 'visit_5_arm_1' OR [event-name] = 'visit_8_arm_1' OR [event-name] = 'visit_9_arm_1' OR [event-name] = 'visit_11_arm_1'	Were the routine samples taken as per protocol ?	yesno, Required<br>1 Yes<br>0 No
179	[nano_la_rt_no]<br>Show the field ONLY if:<br>[nano_la_blood_sample_routine] = '0'	Specify why	text, Required
180	[nano_la_blood_sample_research]<br>Show the field ONLY if:<br>[event-name] = '1st_vaccination_arm_1' OR [event-name] = '2nd_vaccination_arm_1' OR [event-name] = 'visit_9_arm_1' OR [event-name] = 'visit_11_arm_1' OR [event-name] = 'visit_12_arm_1'	Were the research samples taken as per protocol ?	yesno, Required<br>1 Yes<br>0 No
181	[nano_la_rs_no]<br>Show the field ONLY if:<br>[nano_la_blood_sample_research] = '0'	Specify why	text, Required
182	[nano_la_deviation]	If necessary, please fill the following forms<br>Please fill the minor protocol deviation form	descriptive

	Show the field ONLY if: [nano_la_blood_sample_research] = '0'	- Please fill the major protocol deviation form	
183	[nano_la_poc_sarscov2_antigen] Show the field ONLY if: [event-name] = '1st_vaccination_arm_1' or [event-name] = '2nd_vaccination_arm_1'	Section Header: *POC tests results* SARS-CoV-2 rapid antigen test on nasopharyngeal swab	radio, Required<br>1 Positive<br>2 Negative
184	[nano_la_poc_sarscov2_antigen_2] Show the field ONLY if: [event-name] = '1st_vaccination_arm_1' or [event-name] = '2nd_vaccination_arm_1'	Oro-pharyngeal SARS-CoV-2 PCR test	radio, Required<br>1 Positive<br>2 Negative
185	[nano_la_poc_pregnancy] Show the field ONLY if: ([screening_arm_1][nano_vi_gender] = '2' and [event-name] = '1st_vaccination_arm_1') OR ([screening_arm_1][nano_vi_gender] = '2' and [event-name] = '2nd_vaccination_arm_1')	Pregnancy test result	radio, Required<br>1 Positive<br>2 Negative
186	[nano_la_poc_dengue_pos_3] Show the field ONLY if: [nano_la_poc_pregnancy] = '1' and [event-name] = 'screening_arm_1'	The volunteer CAN NOT BE ENROLLED in the trial, please complete the SCREEN FAILURE page	descriptive
187	[nano_la_poc_dengue_pos_2] Show the field ONLY if: ([nano_la_poc_pregnancy] = '1' and [event-name] = '2nd_vaccination_arm_1')	The volunteer CAN NOT BE VACCINATED. Please complete the END OF TRIAL page and the PREGNANCY form	descriptive
188	[nano_la_uri_hematuria] Show the field ONLY if: [event-name] = 'screening_arm_1'	Section Header: *Urine* Hematuria	radio, Required<br>a 0<br>b +<br>c ++<br>d +++

189	[nano_la_uri_proteins]<br>Show the field ONLY if:<br>[event-name] = 'screening_arm_1'	Proteins in urine	radio, Required<br>a 0<br>b +<br>c ++<br>d +++
190	[nano_la_uri_glucose]<br>Show the field ONLY if:<br>[event-name] = 'screening_arm_1'	Glucose in urine	radio, Required<br>a 0<br>b +<br>c ++<br>d +++
191	[nano_la_poc_urine_failure]<br>Show the field ONLY if:<br>[nano_la_uri_hematuria] = 'b' or [nano_la_uri_hematuria] = 'c' or [nano_la_uri_hematuria] = 'd' or [nano_la_uri_proteins] = 'b' or [nano_la_uri_proteins] = 'c' or [nano_la_uri_proteins] = 'd' or [nano_la_uri_glucose] = 'b' or [nano_la_uri_glucose] = 'c' or [nano_la_uri_glucose] = 'd'	Please repeat or, if clinically significant, directly complete the SCREEN FAILURE page	descriptive
192	[nano_la_uri_pregnancy]<br>Show the field ONLY if:<br>[nano_vi_gender] = '2' AND [event-name] = 'screening_arm_1'	Pregnancy test result	radio, Required<br>1 Positive<br>2 Negative
193	[nano_la_uri_pregnant]<br>Show the field ONLY if:<br>[nano_la_uri_pregnancy] = '1'	The volunteer CAN NOT BE ENROLLED in the trial, please complete the SCREEN FAILURE page	descriptive
194	[nano_la_hem_leukocythes]<br>Show the field ONLY if:<br>[event-name] = 'screening_arm_1' OR [event-name] = 'visit_4_arm_1' OR [event-name] = 'visit_5_arm_1' OR [event-name] = "visit_8_arm_1" or [event-name] = 'visit_9_arm_1'	Section Header: *Hematology*<br>Leukocytes<br>*G/l - REF RANGE: 4 - 10*	text (number, Min: 0, Max: 100), Required
195	[nano_la_oor]	Is this value out of range?	yesno, Required<br>1 Yes

	Show the field ONLY if: [event-name] = 'screening_arm_1' OR [event-name] = 'visit_4_arm_1' OR [event-name] = 'visit_5_arm_1' OR [event-name] = "visit_8_arm_1" or [event-name] = 'visit_9_arm_1'		0 No
196	[nano_la_oor_deviation] Show the field ONLY if: ([event-name] = 'visit_4_arm_1' OR [event-name] = 'visit_5_arm_1' OR [event-name] = "visit_8_arm_1" or [event-name] = 'visit_9_arm_1') and [nano_la_oor] = "1"	Is it a deviation listed here or a clinically significant deviation from normal ranges?	yesno, Required<br>1 Yes<br>0 No
197	[nano_la_oor_deviation_21] Show the field ONLY if: [event-name] = 'screening_arm_1' and [nano_la_oor] = "1"	Is it a clinically significant deviation from normal ranges?	yesno, Required<br>1 Yes<br>0 No
198	[nano_la_hem_erythrocythes] Show the field ONLY if: [event-name] = 'screening_arm_1' OR [event-name] = 'visit_4_arm_1' OR [event-name] = 'visit_5_arm_1' OR [event-name] = "visit_8_arm_1" or [event-name] = 'visit_9_arm_1'	Erythrocytes<br>*T/l - REF RANGE: 4.4 - 5.8 (Male) - 3.8 - 5.2 (Female)*	text (number, Min: 0, Max: 15), Required
199	[nano_la_oor_2] Show the field ONLY if: [event-name] = 'screening_arm_1' OR [event-name] = 'visit_4_arm_1' OR [event-name] = 'visit_5_arm_1' OR [event-name] = "visit_8_arm_1" or [event-name] = 'visit_9_arm_1'	Is this value out of range?	yesno, Required<br>1 Yes<br>0 No
200	[nano_la_oor_deviation_2] Show the field ONLY if: ([event-name] = 'visit_4_arm_1' OR [event-na	Is it a deviation listed here or a clinically significant deviation from normal ranges?	yesno, Required<br>1 Yes<br>0 No

	me] = 'visit_5_arm_1' OR [event-name] = "visit_8_arm_1" or [event-name] = 'visit_9_arm_1') and [nano_la_oor_2] = "1"		
201	[nano_la_oor_deviation_22]<br><br>Show the field ONLY if:<br>[event-name] = 'screening_arm_1' and [nano_la_oor_2] = "1"	Is it a clinically significant deviation from normal ranges?	yesno, Required<br>1 Yes<br>0 No
202	[nano_la_hem_hemoglobin]<br><br>Show the field ONLY if:<br>[event-name] = 'screening_arm_1' OR [event-name] = 'visit_4_arm_1' OR [event-name] = 'visit_5_arm_1' OR [event-name] = "visit_8_arm_1" or [event-name] = 'visit_9_arm_1'	Hemoglobin<br>*g/l - REF RANGE: 133 - 177 (male) - 117 - 157 (female)*	text (number), Required
203	[nano_la_oor_3]<br><br>Show the field ONLY if:<br>[event-name] = 'screening_arm_1' OR [event-name] = 'visit_4_arm_1' OR [event-name] = 'visit_5_arm_1' OR [event-name] = "visit_8_arm_1" or [event-name] = 'visit_9_arm_1'	Is this value out of range?	yesno, Required<br>1 Yes<br>0 No
204	[nano_la_oor_deviation_3]<br><br>Show the field ONLY if:<br>([event-name] = 'visit_4_arm_1' OR [event-name] = 'visit_5_arm_1' OR [event-name] = "visit_8_arm_1" or [event-name] = 'visit_9_arm_1') and [nano_la_oor_3] = "1"	Is it a deviation listed here or a clinically significant deviation from normal ranges?	yesno, Required<br>1 Yes<br>0 No
205	[nano_la_oor_deviation_23]<br><br>Show the field ONLY if:<br>[event-name] = 'screening_arm_1' and [nano_la_oor_3] = "1"	Is it a clinically significant deviation from normal ranges?	yesno, Required<br>1 Yes<br>0 No
206	[nano_la_hem_hematocrit]	Hematocrit<br>*% - REF RANGE: 40 - 52 (male) - 35 - 47 (female)*	text (number), Required

	Show the field ONLY if: [event-name] = 'screening_arm_1' OR [event-name] = 'visit_4_arm_1' OR [event-name] = 'visit_5_arm_1' OR [event-name] = "visit_8_arm_1" or [event-name] = 'visit_9_arm_1'		
207	[nano_la_oor_4] Show the field ONLY if: [event-name] = 'screening_arm_1' OR [event-name] = 'visit_4_arm_1' OR [event-name] = 'visit_5_arm_1' OR [event-name] = "visit_8_arm_1" or [event-name] = 'visit_9_arm_1'	Is this value out of range?	yesno, Required<br>1 Yes<br>0 No
208	[nano_la_oor_deviation_4] Show the field ONLY if: ([event-name] = 'visit_4_arm_1' OR [event-name] = 'visit_5_arm_1' OR [event-name] = "visit_8_arm_1" or [event-name] = 'visit_9_arm_1') and [nano_la_oor_4] = "1"	Is it a deviation listed here or a clinically significant deviation from normal ranges?	yesno, Required<br>1 Yes<br>0 No
209	[nano_la_oor_deviation_24] Show the field ONLY if: [event-name] = 'screening_arm_1' and [nano_la_oor_4] = "1"	Is it a clinically significant deviation from normal ranges?	yesno, Required<br>1 Yes<br>0 No
210	[nano_la_hem_mcv] Show the field ONLY if: [event-name] = 'screening_arm_1' OR [event-name] = 'visit_4_arm_1' OR [event-name] = 'visit_5_arm_1' OR [event-name] = "visit_8_arm_1" or [event-name] = 'visit_9_arm_1'	MCV (Mean corpuscular volume)<br>*fl - REF RANGE: 81 - 99*	text (number), Required
211	[nano_la_oor_5] Show the field ONLY if: [event-name] = 'screening_arm_1' OR [event-name] = 'visit_4_arm_	Is this value out of range?	yesno, Required<br>1 Yes<br>0 No

	1' OR [event-name] = 'visit_5_arm_1' OR [event-name] = "visit_8_arm_1" or [event-name] = 'visit_9_arm_1'		
212	[nano_la_oor_deviation_5]<br>Show the field ONLY if:<br>([event-name] = 'visit_4_arm_1' OR [event-name] = 'visit_5_arm_1' OR [event-name] = "visit_8_arm_1" or [event-name] = 'visit_9_arm_1') and [nano_la_oor_5] = "1"	Is it a deviation listed here or a clinically significant deviation from normal ranges?	yesno, Required<br>1 Yes<br>0 No
213	[nano_la_oor_deviation_25]<br>Show the field ONLY if:<br>[event-name] = 'screening_arm_1' and [nano_la_oor_5] = "1"	Is it a clinically significant deviation from normal ranges?	yesno, Required<br>1 Yes<br>0 No
214	[nano_la_hem_mch]<br>Show the field ONLY if:<br>[event-name] = 'screening_arm_1' OR [event-name] = 'visit_4_arm_1' OR [event-name] = 'visit_5_arm_1' OR [event-name] = "visit_8_arm_1" or [event-name] = 'visit_9_arm_1'	MCH (Mean cell hemoglobin)<br>*pg - REF RANGE: 27 - 34*	text (number, Min: 5, Max: 50), Required
215	[nano_la_oor_6]<br>Show the field ONLY if:<br>[event-name] = 'screening_arm_1' OR [event-name] = 'visit_4_arm_1' OR [event-name] = 'visit_5_arm_1' OR [event-name] = "visit_8_arm_1" or [event-name] = 'visit_9_arm_1'	Is this value out of range?	yesno, Required<br>1 Yes<br>0 No
216	[nano_la_oor_deviation_6]<br>Show the field ONLY if:<br>([event-name] = 'visit_4_arm_1' OR [event-name] = 'visit_5_arm_1' OR [event-name] = "visit_8_arm_1" or [event-name] = 'visit_9_arm_1') a	Is it a deviation listed here or a clinically significant deviation from normal ranges?	yesno, Required<br>1 Yes<br>0 No

	nd [nano_la_oor_6] = "1"		
217	[nano_la_oor_deviation_26]<br>Show the field ONLY if:<br>[event-name] = 'screening_arm_1' and [nano_la_oor_6] = "1"	Is it a clinically significant deviation from normal ranges?	yesno, Required<br>1 Yes<br>0 No
218	[nano_la_hem_mchc]<br>Show the field ONLY if:<br>[event-name] = 'screening_arm_1' OR [event-name] = 'visit_4_arm_1' OR [event-name] = 'visit_5_arm_1' OR [event-name] = "visit_8_arm_1" or [event-name] = 'visit_9_arm_1'	MCHC (Mean corpuscular hemoglobin concentration)<br>*g/l - REF RANGE: 310 - 360*	text (number), Required
219	[nano_la_oor_7]<br>Show the field ONLY if:<br>[event-name] = 'screening_arm_1' OR [event-name] = 'visit_4_arm_1' OR [event-name] = 'visit_5_arm_1' OR [event-name] = "visit_8_arm_1" or [event-name] = 'visit_9_arm_1'	Is this value out of range?	yesno, Required<br>1 Yes<br>0 No
220	[nano_la_oor_deviation_7]<br>Show the field ONLY if:<br>([event-name] = 'visit_4_arm_1' OR [event-name] = 'visit_5_arm_1' OR [event-name] = "visit_8_arm_1" or [event-name] = 'visit_9_arm_1') and [nano_la_oor_7] = "1"	Is it a deviation listed here or a clinically significant deviation from normal ranges?	yesno, Required<br>1 Yes<br>0 No
221	[nano_la_oor_deviation_27]<br>Show the field ONLY if:<br>[event-name] = 'screening_arm_1' and [nano_la_oor_7] = "1"	Is it a clinically significant deviation from normal ranges?	yesno, Required<br>1 Yes<br>0 No
222	[nano_la_hem_platelets]<br>Show the field ONLY if:<br>[event-name] = 'screening_arm_1' OR [event-	Platelets<br>*G/l - REF RANGE: 150 - 350*	text (integer), Required

	name] = 'visit_4_arm_1' OR [event-name] = 'visit_5_arm_1' OR [event-name] = "visit_8_arm_1" or [event-name] = 'visit_9_arm_1'		
223	[nano_la_oor_8]<br>Show the field ONLY if:<br>[event-name] = 'screening_arm_1' OR [event-name] = 'visit_4_arm_1' OR [event-name] = 'visit_5_arm_1' OR [event-name] = "visit_8_arm_1" or [event-name] = 'visit_9_arm_1'	Is this value out of range?	yesno, Required<br>1 Yes<br>0 No
224	[nano_la_oor_deviation_8]<br>Show the field ONLY if:<br>([event-name] = 'visit_4_arm_1' OR [event-name] = 'visit_5_arm_1' OR [event-name] = "visit_8_arm_1" or [event-name] = 'visit_9_arm_1') and [nano_la_oor_8] = "1"	Is it a deviation listed here or a clinically significant deviation from normal ranges?	yesno, Required<br>1 Yes<br>0 No
225	[nano_la_oor_deviation_28]<br>Show the field ONLY if:<br>[event-name] = 'screening_arm_1' and [nano_la_oor_8] = "1"	Is it a clinically significant deviation from normal ranges?	yesno, Required<br>1 Yes<br>0 No
226	[nano_la_hem_neutro]<br>Show the field ONLY if:<br>[event-name] = 'screening_arm_1' OR [event-name] = 'visit_4_arm_1' OR [event-name] = 'visit_5_arm_1' OR [event-name] = "visit_8_arm_1" or [event-name] = 'visit_9_arm_1'	Neutrophils<br>*G/l - REF RANGE: 1.8 - 7.5*	text (number), Required
227	[nano_la_oor_9]<br>Show the field ONLY if:<br>[event-name] = 'screening_arm_1' OR [event-name] = 'visit_4_arm_1' OR [event-name] = 'visit_5_arm_1' OR [event-name] = "visit_8_ar	Is this value out of range?	yesno, Required<br>1 Yes<br>0 No

	m_1" or [event-name] = 'visit_9_arm_1'		
228	[nano_la_oor_deviation_9]<br>Show the field ONLY if:<br>([event-name] = 'visit_4_arm_1' OR [event-name] = 'visit_5_arm_1' OR [event-name] = "visit_8_arm_1" or [event-name] = 'visit_9_arm_1') and [nano_la_oor_9] = "1"	Is it a deviation listed here or a clinically significant deviation from normal ranges?	yesno, Required<br>1 Yes<br>0 No
229	[nano_la_oor_deviation_29]<br>Show the field ONLY if:<br>[event-name] = 'screening_arm_1' and [nano_la_oor_9] = "1"	Is it a clinically significant deviation from normal ranges?	yesno, Required<br>1 Yes<br>0 No
230	[nano_la_hem_lympho]<br>Show the field ONLY if:<br>[event-name] = 'screening_arm_1' OR [event-name] = 'visit_4_arm_1' OR [event-name] = 'visit_5_arm_1' OR [event-name] = "visit_8_arm_1" or [event-name] = 'visit_9_arm_1'	Lymphocytes<br>*G/l - REF RANGE: 1.5 - 4*	text (number), Required
231	[nano_la_oor_10]<br>Show the field ONLY if:<br>[event-name] = 'screening_arm_1' OR [event-name] = 'visit_4_arm_1' OR [event-name] = 'visit_5_arm_1' OR [event-name] = "visit_8_arm_1" or [event-name] = 'visit_9_arm_1'	Is this value out of range?	yesno, Required<br>1 Yes<br>0 No
232	[nano_la_oor_deviation_10]<br>Show the field ONLY if:<br>([event-name] = 'visit_4_arm_1' OR [event-name] = 'visit_5_arm_1' OR [event-name] = "visit_8_arm_1" or [event-name] = 'visit_9_arm_1') and [nano_la_oor_10] = "1"	Is it a deviation listed here or a clinically significant deviation from normal ranges?	yesno, Required<br>1 Yes<br>0 No
233	[nano_la_oor_deviation_30]	Is it a clinically significant deviation from normal ranges?	yesno, Required

	Show the field ONLY if: [event-name] = 'screening_arm_1' and [nano_la_oor_10] = "1"		1 Yes<br>0 No
234	[nano_la_hem_mono]<br>Show the field ONLY if: [event-name] = 'screening_arm_1' OR [event-name] = 'visit_4_arm_1' OR [event-name] = 'visit_5_arm_1' OR [event-name] = "visit_8_arm_1" or [event-name] = 'visit_9_arm_1'	Monocytes<br>*G/l - REF RANGE: 0.2 - 0.8*	text (number), Required
235	[nano_la_oor_11]<br>Show the field ONLY if: [event-name] = 'screening_arm_1' OR [event-name] = 'visit_4_arm_1' OR [event-name] = 'visit_5_arm_1' OR [event-name] = "visit_8_arm_1" or [event-name] = 'visit_9_arm_1'	Is this value out of range?	yesno, Required<br>1 Yes<br>0 No
236	[nano_la_oor_deviation_11]<br>Show the field ONLY if: ([event-name] = 'visit_4_arm_1' OR [event-name] = 'visit_5_arm_1' OR [event-name] = "visit_8_arm_1" or [event-name] = 'visit_9_arm_1') and [nano_la_oor_11] = "1"	Is it a deviation listed here or a clinically significant deviation from normal ranges?	yesno, Required<br>1 Yes<br>0 No
237	[nano_la_oor_deviation_31]<br>Show the field ONLY if: [event-name] = 'screening_arm_1' and [nano_la_oor_11] = "1"	Is it a clinically significant deviation from normal ranges?	yesno, Required<br>1 Yes<br>0 No
238	[nano_la_hem_eosino]<br>Show the field ONLY if: [event-name] = 'screening_arm_1' OR [event-name] = 'visit_4_arm_1' OR [event-name] = 'visit_5_arm_1' OR [event-name] = "visit_8_arm_1" or [event-name] = 'visit_9_arm_1'	Eosinophils<br>*G/l - REF RANGE: 0.05 - 0.5*	text (number), Required

239	[nano_la_oor_12]<br>Show the field ONLY if:<br>[event-name] = 'screening_arm_1' OR [event-name] = 'visit_4_arm_1' OR [event-name] = 'visit_5_arm_1' OR [event-name] = "visit_8_arm_1" or [event-name] = 'visit_9_arm_1'	Is this value out of range?	yesno, Required<br>1 Yes<br>0 No
240	[nano_la_oor_deviation_12]<br>Show the field ONLY if:<br>([event-name] = 'visit_4_arm_1' OR [event-name] = 'visit_5_arm_1' OR [event-name] = "visit_8_arm_1" or [event-name] = 'visit_9_arm_1') and [nano_la_oor_12] = "1"	Is it a deviation listed here or a clinically significant deviation from normal ranges?	yesno, Required<br>1 Yes<br>0 No
241	[nano_la_oor_deviation_32]<br>Show the field ONLY if:<br>[event-name] = 'screening_arm_1' and [nano_la_oor_12] = "1"	Is it a clinically significant deviation from normal ranges?	yesno, Required<br>1 Yes<br>0 No
242	[nano_la_hem_baso]<br>Show the field ONLY if:<br>[event-name] = 'screening_arm_1' OR [event-name] = 'visit_4_arm_1' OR [event-name] = 'visit_5_arm_1' OR [event-name] = "visit_8_arm_1" or [event-name] = 'visit_9_arm_1'	Basophils<br>*G/l - REF RANGE: 0.01 - 0.05*	text (number), Required
243	[nano_la_oor_13]<br>Show the field ONLY if:<br>[event-name] = 'screening_arm_1' OR [event-name] = 'visit_4_arm_1' OR [event-name] = 'visit_5_arm_1' OR [event-name] = "visit_8_arm_1" or [event-name] = 'visit_9_arm_1'	Is this value out of range?	yesno, Required<br>1 Yes<br>0 No
244	[nano_la_oor_deviation_13]<br>Show the field ONLY if:	Is it a deviation listed here or a clinically significant deviation from normal ranges?	yesno, Required<br>1 Yes<br>0 No

	([event-name] = 'visit_4_arm_1' OR [event-name] = 'visit_5_arm_1' OR [event-name] = "visit_8_arm_1" or [event-name] = 'visit_9_arm_1') and [nano_la_oor_13] = "1"		
245	[nano_la_oor_deviation_33] Show the field ONLY if: [event-name] = 'screening_arm_1' and [nano_la_oor_13] = "1"	Is it a clinically significant deviation from normal ranges?	yesno, Required<br>1 Yes<br>0 No
246	[nano_la_bio_creatinine] Show the field ONLY if: [event-name] = 'screening_arm_1' OR [event-name] = 'visit_4_arm_1' OR [event-name] = 'visit_5_arm_1' OR [event-name] = "visit_8_arm_1" or [event-name] = 'visit_9_arm_1'	Section Header: *Biochemistry*<br>Creatinine<br>*µmol/ - REF RANGE: 62-106 (male) - 44 - 80 (female)*	text (integer), Required
247	[nano_la_oor_14] Show the field ONLY if: [event-name] = 'screening_arm_1' OR [event-name] = 'visit_4_arm_1' OR [event-name] = 'visit_5_arm_1' OR [event-name] = "visit_8_arm_1" or [event-name] = 'visit_9_arm_1'	Is this value out of range?	yesno, Required<br>1 Yes<br>0 No
248	[nano_la_oor_deviation_14] Show the field ONLY if: ([event-name] = 'visit_4_arm_1' OR [event-name] = 'visit_5_arm_1' OR [event-name] = "visit_8_arm_1" or [event-name] = 'visit_9_arm_1') and [nano_la_oor_14] = "1"	Is it a deviation listed here or a clinically significant deviation from normal ranges?	yesno, Required<br>1 Yes<br>0 No
249	[nano_la_oor_deviation_34] Show the field ONLY if: [event-name] = 'screening_arm_1' and [nano_la_oor_14] = "1"	Is it a clinically significant deviation from normal ranges?	yesno, Required<br>1 Yes<br>0 No

250	[nano_la_bio_bilirubin]<br>Show the field ONLY if:<br>[event-name] = 'screening_arm_1' OR [event-name] = 'visit_4_arm_1' OR [event-name] = 'visit_5_arm_1' OR [event-name] = "visit_8_arm_1" or [event-name] = 'visit_9_arm_1'	Total bilirubin<br>*µmol/l - REF RANGE: 0 - 21*	text (integer), Required
251	[nano_la_oor_19]<br>Show the field ONLY if:<br>[event-name] = 'screening_arm_1' OR [event-name] = 'visit_4_arm_1' OR [event-name] = 'visit_5_arm_1' OR [event-name] = "visit_8_arm_1" or [event-name] = 'visit_9_arm_1'	Is this value out of range?	yesno, Required<br>1 Yes<br>0 No
252	[nano_la_oor_deviation_19]<br>Show the field ONLY if:<br>([event-name] = 'visit_4_arm_1' OR [event-name] = 'visit_5_arm_1' OR [event-name] = "visit_8_arm_1" or [event-name] = 'visit_9_arm_1') and [nano_la_oor_19] = "1"	Is it a deviation listed here or a clinically significant deviation from normal ranges?	yesno, Required<br>1 Yes<br>0 No
253	[nano_la_oor_deviation_39]<br>Show the field ONLY if:<br>[event-name] = 'screening_arm_1' and [nano_la_oor_19] = "1"	Is it a clinically significant deviation from normal ranges?	yesno, Required<br>1 Yes<br>0 No
254	[nano_la_bio_c_react]<br>Show the field ONLY if:<br>[event-name] = 'screening_arm_1' OR [event-name] = 'visit_4_arm_1' OR [event-name] = 'visit_5_arm_1' OR [event-name] = "visit_8_arm_1" or [event-name] = 'visit_9_arm_1'	C reactive protein<br>*mg/l - REF RANGE: < 10*	text, Required
255	[nano_la_oor_20]<br>Show the field ONLY if:	Is this value out of range?	yesno, Required<br>1 Yes

	[event-name] = 'screening_arm_1' OR [event-name] = 'visit_4_arm_1' OR [event-name] = 'visit_5_arm_1' OR [event-name] = "visit_8_arm_1" or [event-name] = 'visit_9_arm_1'		0 No
256	[nano_la_oor_deviation_20]<br>Show the field ONLY if:<br>([event-name] = 'visit_4_arm_1' OR [event-name] = 'visit_5_arm_1' OR [event-name] = "visit_8_arm_1" or [event-name] = 'visit_9_arm_1') and [nano_la_oor_20] = "1"	Is it a deviation listed here or a clinically significant deviation from normal ranges?	yesno, Required<br>1 Yes<br>0 No
257	[nano_la_oor_deviation_40]<br>Show the field ONLY if:<br>[event-name] = 'screening_arm_1' and [nano_la_oor_20] = "1"	Is it a clinically significant deviation from normal ranges?	yesno, Required<br>1 Yes<br>0 No
258	[nano_la_bio_ast]<br>Show the field ONLY if:<br>[event-name] = 'screening_arm_1' OR [event-name] = 'visit_4_arm_1' OR [event-name] = 'visit_5_arm_1' OR [event-name] = "visit_8_arm_1" or [event-name] = 'visit_9_arm_1'	AST<br>*UI/l - REF RANGE: 9 - 50*	text (integer), Required
259	[nano_la_oor_15]<br>Show the field ONLY if:<br>[event-name] = 'screening_arm_1' OR [event-name] = 'visit_4_arm_1' OR [event-name] = 'visit_5_arm_1' OR [event-name] = "visit_8_arm_1" or [event-name] = 'visit_9_arm_1'	Is this value out of range?	yesno, Required<br>1 Yes<br>0 No
260	[nano_la_oor_deviation_15]<br>Show the field ONLY if:<br>([event-name] = 'visit_4_arm_1' OR [event-name] = 'visit_5_arm_1' OR [event-name] = "visit	Is it a deviation listed here or a clinically significant deviation from normal ranges?	yesno, Required<br>1 Yes<br>0 No

	_8_arm_1" or [event-name] = 'visit_9_arm_1') and [nano_la_oor_15] = "1"		
261	[nano_la_oor_deviation_35]<br>Show the field ONLY if:<br>[event-name] = 'screening_arm_1' and [nano_la_oor_15] = "1"	Is it a clinically significant deviation from normal ranges?	yesno, Required<br>1 Yes<br>0 No
262	[nano_la_bio_alt]<br>Show the field ONLY if:<br>[event-name] = 'screening_arm_1' OR [event-name] = 'visit_4_arm_1' OR [event-name] = 'visit_5_arm_1' OR [event-name] = "visit_8_arm_1" or [event-name] = 'visit_9_arm_1'	ALT<br>*UI/l - REF RANGE: 9 - 50*	text (integer), Required
263	[nano_la_oor_16]<br>Show the field ONLY if:<br>[event-name] = 'screening_arm_1' OR [event-name] = 'visit_4_arm_1' OR [event-name] = 'visit_5_arm_1' OR [event-name] = "visit_8_arm_1" or [event-name] = 'visit_9_arm_1'	Is this value out of range?	yesno, Required<br>1 Yes<br>0 No
264	[nano_la_oor_deviation_16]<br>Show the field ONLY if:<br>([event-name] = 'visit_4_arm_1' OR [event-name] = 'visit_5_arm_1' OR [event-name] = "visit_8_arm_1" or [event-name] = 'visit_9_arm_1') and [nano_la_oor_16] = "1"	Is it a deviation listed here or a clinically significant deviation from normal ranges?	yesno, Required<br>1 Yes<br>0 No
265	[nano_la_oor_deviation_36]<br>Show the field ONLY if:<br>[event-name] = 'screening_arm_1' and [nano_la_oor_16] = "1"	Is it a clinically significant deviation from normal ranges?	yesno, Required<br>1 Yes<br>0 No
266	[nano_la_bio_gct]<br>Show the field ONLY if:	GGT<br>*UI/l - REF RANGE: 6 - 42*	text (integer), Required

	[event-name] = 'screening_arm_1' OR [event-name] = 'visit_4_arm_1' OR [event-name] = 'visit_5_arm_1' OR [event-name] = "visit_8_arm_1" or [event-name] = 'visit_9_arm_1'		
267	[nano_la_oor_18]<br>Show the field ONLY if:<br>[event-name] = 'screening_arm_1' OR [event-name] = 'visit_4_arm_1' OR [event-name] = 'visit_5_arm_1' OR [event-name] = "visit_8_arm_1" or [event-name] = 'visit_9_arm_1'	Is this value out of range?	yesno, Required<br>1 Yes<br>0 No
268	[nano_la_oor_deviation_18]<br>Show the field ONLY if:<br>([event-name] = 'visit_4_arm_1' OR [event-name] = 'visit_5_arm_1' OR [event-name] = "visit_8_arm_1" or [event-name] = 'visit_9_arm_1') and [nano_la_oor_18] = "1"	Is it a deviation listed here or a clinically significant deviation from normal ranges?	yesno, Required<br>1 Yes<br>0 No
269	[nano_la_oor_deviation_38]<br>Show the field ONLY if:<br>[event-name] = 'screening_arm_1' and [nano_la_oor_18] = "1"	Is it a clinically significant deviation from normal ranges?	yesno, Required<br>1 Yes<br>0 No
270	[nano_la_bio_alka]<br>Show the field ONLY if:<br>[event-name] = 'screening_arm_1' OR [event-name] = 'visit_4_arm_1' OR [event-name] = 'visit_5_arm_1' OR [event-name] = "visit_8_arm_1" or [event-name] = 'visit_9_arm_1'	Alkaline phosphatase<br>*UI/l - REF RANGE: 36 - 120*	text (integer), Required
271	[nano_la_oor_17]<br>Show the field ONLY if:<br>[event-name] = 'screening_arm_1' OR [event-name] = 'visit_4_arm_1' OR [event-name] = 'visit_5_arm_1' OR [eve	Is this value out of range?	yesno, Required<br>1 Yes<br>0 No

	nt-name] = "visit_8_arm_1" or [event-name] = 'visit_9_arm_1'		
272	[nano_la_oor_deviation_17]<br>Show the field ONLY if:<br>([event-name] = 'visit_4_arm_1' OR [event-name] = 'visit_5_arm_1' OR [event-name] = "visit_8_arm_1" or [event-name] = 'visit_9_arm_1') and [nano_la_oor_17] = "1"	Is it a deviation listed here or a clinically significant deviation from normal ranges?	yesno, Required<br>1 Yes<br>0 No
273	[nano_la_oor_deviation_37]<br>Show the field ONLY if:<br>[event-name] = 'screening_arm_1' and [nano_la_oor_17] = "1"	Is it a clinically significant deviation from normal ranges?	yesno, Required<br>1 Yes<br>0 No
274	[nano_la_sero_aghbs]<br>Show the field ONLY if:<br>[event-name] = 'screening_arm_1'	Section Header: *Serologies*<br>AgHbs	radio, Required<br>1 Positive<br>2 Negative
275	[nano_la_sero_anti_hbc]<br>Show the field ONLY if:<br>[event-name] = 'screening_arm_1'	Antibodies anti-Hbc	radio, Required<br>1 Positive<br>2 Negative
276	[nano_la_sero_anti_hcv]<br>Show the field ONLY if:<br>[event-name] = 'screening_arm_1'	Anti-HCV antibodies	radio, Required<br>1 Positive<br>2 Negative
277	[nano_la_sero_hiv]<br>Show the field ONLY if:<br>[event-name] = 'screening_arm_1'	HIV antibodies	radio, Required<br>1 Positive<br>2 Negative
278	[nano_la_screen_failure]<br>Show the field ONLY if:<br>[nano_la_sero_aghbs] = "1" OR [nano_la_sero_anti_hbc] = "1" OR [nano_la_sero_anti_hcv] = "1" OR [nano_la_sero_hiv] = "1"	The volunteer CAN NOT BE ENROLLED in the trial, please complete the SCREEN FAILURE page	descriptive

279	[nano_la_anti_nuclear]<br>Show the field ONLY if:<br>[event-name] = 'screening_arm_1' or [event-name] = "visit_11_arm_1"	Section Header: *Autoimmune reactions*<br>Anti-nuclear antibodies<br>*Titer - REF RANGE < 1/80*	text, Required
280	[nano_la_oor_21]<br>Show the field ONLY if:<br>[event-name] = 'screening_arm_1' or [event-name] = "visit_11_arm_1"	Is this value out of range?	yesno, Required<br>1 Yes<br>0 No
281	[nano_la_oor_deviation_41]<br>Show the field ONLY if:<br>[event-name] = "visit_11_arm_1" and [nano_la_oor_21] = "1"	Is it a deviation listed here or a clinically significant deviation from normal ranges?	yesno, Required<br>1 Yes<br>0 No
282	[nano_la_oor_deviation_42]<br>Show the field ONLY if:<br>[event-name] = 'screening_arm_1' and [nano_la_oor_21] = "1"	Is it a clinically significant deviation from normal ranges?	yesno, Required<br>1 Yes<br>0 No
283	[dep_name8]<br>Show the field ONLY if:<br>[event-name] = 'screening_arm_1' OR [event-name] = 'visit_4_arm_1' OR [event-name] = 'visit_5_arm_1' OR [event-name] = "visit_8_arm_1" or [event-name] = 'visit_9_arm_1' or [event-name] = 'visit_11_arm_1'	Section Header: *Deviation from normal ranges*<br>Research Physician	descriptive
284	[nano_lab_severity_grade]<br>Show the field ONLY if:<br>[event-name] = 'screening_arm_1' OR [event-name] = 'visit_4_arm_1' OR [event-name] = 'visit_5_arm_1' OR [event-name] = "visit_8_arm_1" or [event-name] = 'visit_9_arm_1' or [event-name] = 'visit_11_arm_1'		descriptive<br>(Attachment: Severity grading for abnormal laboratory measures.pdf, Display format: Link)<br>Field Annotation: @HIDDEN

285	[nano_la_not_enrolled]<br><br>Show the field ONLY if:<br>([nano_la_oor_deviation] = "1" or [nano_la_oor_deviation_2] = "1" or [nano_la_oor_deviation_3] = "1" or [nano_la_oor_deviation_4] = "1" or [nano_la_oor_deviation_5] = "1" or [nano_la_oor_deviation_6] = "1" or [nano_la_oor_deviation_7] = "1" or [nano_la_oor_deviation_8] = "1" or [nano_la_oor_deviation_9] = "1" or [nano_la_oor_deviation_10] = "1" or [nano_la_oor_deviation_11] = "1" or [nano_la_oor_deviation_12] = "1" or [nano_la_oor_deviation_13] = "1" or [nano_la_oor_deviation_14] = "1" or [nano_la_oor_deviation_15] = "1" or [nano_la_oor_deviation_16] = "1" or [nano_la_oor_deviation_17] = "1" or [nano_la_oor_deviation_18] = "1" or [nano_la_oor_deviation_19] = "1" or [nano_la_oor_deviation_20] = "1" or [nano_la_oor_deviation_21] = "1" or [nano_la_oor_deviation_22] = "1" or [nano_la_oor_deviation_23] = "1" or [nano_la_oor_deviation_24] = "1" or [nano_la_oor_deviation_25] = "1" or [nano_la_oor_deviation_26] = "1" or [nano_la_oor_deviation_27] = "1" or [nano_la_oor_deviation_28] = "1" or [nano_la_oor_deviation_29] = "1" or [nano_la_oor_deviation_30] = "1" or [nano_la_oor_deviation_31] = "1" or [nano_la_oor_deviation_32] = "1" or [nano_la_oor_deviation_33] = "1" or [nano_la_oor_deviation_34] = "1" or [nano_la_oor_deviation_35] = "1" or [nano_la_oor_deviation_36] = "1" or [nano_la_oor_deviati	If an abnormal test result is deemed clinically significant it may be repeated to ensure it is not a single occurrence. If the test is repeated, please note the first value in the comment field below (the last value will be entered in the corresponding field above). If after evaluation, the participant CAN NOT BE ENROLLED in the trial, please complete the SCREEN FAILURE page	descriptive

	on_37] = "1" or [nano_la_oor_deviation_38] = "1" or [nano_la_oor_deviation_39] = "1" or [nano_la_oor_deviation_40] = "1") and [event-name] = "screening_arm_1"		
286	[nano_la_comment]	Any comment ?	radio<br>1 Yes {nano_la_comment_2}<br>2 No
287	[nano_la_comment_2]<br>Show the field ONLY if:<br>[nano_la_comment] = '1'		notes
288	[nano_la_list]<br>Show the field ONLY if:<br>([nano_la_oor_deviation] = "1" or [nano_la_oor_deviation_2] = "1" or [nano_la_oor_deviation_3] = "1" or [nano_la_oor_deviation_4] = "1" or [nano_la_oor_deviation_5] = "1" or [nano_la_oor_deviation_6] = "1" or [nano_la_oor_deviation_7] = "1" or [nano_la_oor_deviation_8] = "1" or [nano_la_oor_deviation_9] = "1" or [nano_la_oor_deviation_10] = "1" or [nano_la_oor_deviation_11] = "1" or [nano_la_oor_deviation_12] = "1" or [nano_la_oor_deviation_13] = "1" or [nano_la_oor_deviation_14] = "1" or [nano_la_oor_deviation_15] = "1" or [nano_la_oor_deviation_16] = "1" or [nano_la_oor_deviation_17] = "1" or [nano_la_oor_deviation_18] = "1" or [nano_la_oor_deviation_19] = "1" or [nano_la_oor_deviation_20] = "1" or [nano_la_oor_deviation_21] = "1" or [nano_la_oor_deviation_22] = "1" or [nano_la_oor_deviation_23] = "1" or [nano_la_oor_deviation_24] = "1" or [nano_la_oor_deviation_25] = "1" or [nano_la_oo	Complete/update the appropriate AE / SAE form(s) SAE form - AE form<br>Ensure to provide an appropriate clinical management<br>Assess if the patient is still eligible for the trial. If not, complete the END OF TRIAL page.	descriptive

	r_deviation_26] = "1" or [nano_la_oor_deviation_27] = "1" or [nano_la_oor_deviation_28] = "1" or [nano_la_oor_deviation_29] = "1" or [nano_la_oor_deviation_30] = "1" or [nano_la_oor_deviation_31] = "1" or [nano_la_oor_deviation_32] = "1" or [nano_la_oor_deviation_33] = "1" or [nano_la_oor_deviation_34] = "1" or [nano_la_oor_deviation_35] = "1" or [nano_la_oor_deviation_36] = "1" or [nano_la_oor_deviation_37] = "1" or [nano_la_oor_deviation_38] = "1" or [nano_la_oor_deviation_39] = "1" or [nano_la_oor_deviation_40] = "1") and [event-name] <> "screening_arm_1"		
289	[laboratory_analyses_complete]	Section Header: *Form Status*<br>Complete?	dropdown<br>0 Incomplete<br>1 Unverified<br>2 Complete
**Instrument: Contraception (contraception)**			
290	[dep_name9]<br>Show the field ONLY if:<br>[event-name] = "screening_arm_1" OR [event-name] = "1st_vaccination_arm_1" OR [event-name] = "2nd_vaccination_arm_1"	Research Physician	descriptive
291	[nano_sae_contraception]<br>Show the field ONLY if:<br>[screening_arm_1][nano_vi_gender] = '2'	Has the volunteer used a continuous effective contraception since the last visit ?	yesno, Required<br>1 Yes<br>0 No

292	[nano_sae_contraception_specify]<br><br>Show the field ONLY if:<br>[nano_sae_contraception] = "1"	Please specify	radio, Required<br>a oral, injected or implanted hormonal methods<br>b Intrauterine device (IUD)<br>c Barrier methods (condom or occlusive cap with spermicide)<br>d Male (partner) or female (self) sterilization.<br>e True abstinence
293	[nano_c_text_2]<br><br>Show the field ONLY if:<br>[nano_sae_contraception_specify] = "a" OR [nano_sae_contraception_specify] = "b"	Update the medication form if needed	descriptive
294	[nano_c_text]<br><br>Show the field ONLY if:<br>[nano_sae_contraception] = "1"	Contraception has to be continued until Day 90.	descriptive
295	[nano_c_screen_failure]<br><br>Show the field ONLY if:<br>[nano_sae_contraception] = "0" AND [event-name] = '1st _vaccination_arm_1'	If no contraception was used in the past 4 weeks, the subject cannot be vaccinated. Please consider postponement of vaccination or complete the SCREEN FAILURE page	descriptive
296	[nano_c_text_3]<br><br>Show the field ONLY if:<br>[nano_sae_contraception] = "0" AND [screening_arm_1][nano_vi_gender] = "2" AND [event-name] <> 'visit_12_arm_1'	Proceed to a pregnancy test and assess with the PI if the participant can continue the trial. If indicated, fill the END OF TRIAL form.<br>END OF TRIAL form	descriptive
297	[nano_sae_contraception_preg_test_date]<br><br>Show the field ONLY if:<br>[screening_arm_1][nano_vi_gender] = '2' and [event-name] <> '1st_vaccination_arm_1' and [nano_sae_contraception] = '0'	Pregnancy test Date	text (date_dmy), Required
298	[nano_sae_contraception_preg_test_result]<br><br>Show the field ONLY if:	Pregnancy test result	radio, Required<br>1 Positive<br>2 Negative

	[screening_arm_1][nano_vi_gender] = '2' and [event-name] <> '1st_vaccination_arm_1' and [nano_sae_contraception] = '0'		
299	[nano_c_text_4]<br>Show the field ONLY if:<br>[nano_sae_contraception_preg_test_result] = '1'	Please fill the the PREGNANCY form	descriptive
300	[contraception_complete]	Section Header: *Form Status*<br>Complete?	dropdown<br>0 Incomplete<br>1 Unverified<br>2 Complete
**Instrument: Eligibility (eligibility)**			
301	[dep_name10]<br>Show the field ONLY if:<br>[event-name] = "screening_arm_1" OR [event-name] = "1st_vaccination_arm_1" OR [event-name] = "2nd_vaccination_arm_1"	Research Physician (except prescription form)	descriptive
302	[nano_e_text]<br>Show the field ONLY if:<br>[event-name] = 'screening_arm_1'	Pre-eligibility	descriptive
303	[nano_e_text_2]<br>Show the field ONLY if:<br>[event-name] <> 'screening_arm_1' and [event-name] <> 'visit_11_arm_1' and [event-name] <> 'visit_5_arm_1'	Review of eligibility	descriptive
304	[nano_e_aged]<br>Show the field ONLY if:<br>[event-name] = "screening_arm_1" OR [event-name] = "1st_vaccination_arm_1" OR [event-name] = "2nd_vaccination_arm_1"	Section Header: *Inclusion criteria*<br>Aged 18 to 45 years	yesno, Required<br>1 Yes<br>0 No
305	[nano_e_consent]<br>Show the field ONLY if:<br>[event-name] = "screening_arm_1" OR [event-name] = "1st_vaccinati	Provide written informed consent	yesno, Required<br>1 Yes<br>0 No

	on_arm_1" OR [event-name] = "2nd_vaccination_arm_1"		
306	[nano_e_able_willing]<br>Show the field ONLY if:<br>[event-name] = "screening_arm_1" OR [event-name] = "1st_vaccination_arm_1" OR [event-name] = "2nd_vaccination_arm_1"	Residing in Switzerland	yesno, Required<br>1 Yes<br>0 No
307	[nano_e_failure]<br>Show the field ONLY if:<br>[nano_e_aged] = '0' or [nano_e_consent] = '0' or [nano_e_able_willing] = '0'	The volunteer CAN NOT BE ENROLLED in the trial, please complete the SCREEN FAILURE page	descriptive
308	[nano_e_chronic]<br>Show the field ONLY if:<br>[event-name] = "screening_arm_1" OR [event-name] = "1st_vaccination_arm_1" OR [event-name] = "2nd_vaccination_arm_1"	Section Header: *Exclusion criteria*<br>Chronic illness that is at a stage where it might interfere with trial conduct or completion	yesno, Required<br>1 Yes<br>0 No
309	[nano_e_addiction]<br>Show the field ONLY if:<br>[event-name] = "screening_arm_1" OR [event-name] = "1st_vaccination_arm_1" OR [event-name] = "2nd_vaccination_arm_1"	Current alcohol abuse or drug addiction (reported or suspected)	yesno, Required<br>1 Yes<br>0 No
310	[nano_e_congenital]<br>Show the field ONLY if:<br>[event-name] = "screening_arm_1" OR [event-name] = "1st_vaccination_arm_1" OR [event-name] = "2nd_vaccination_arm_1"	Known or suspected congenital or acquired immunodeficiency or receipt of immunosuppressive therapy	yesno, Required<br>1 Yes<br>0 No
311	[nano_e_hypersensitivity]<br>Show the field ONLY if:<br>[event-name] = "screening_arm_1" OR [event-name] = "1st_vaccination_arm_1" OR [event-	Known systemic hypersensitivity to any of the vaccine components (e.g. gold), or history of a life-threatening reaction to vaccines	yesno, Required<br>1 Yes<br>0 No

	name] = "2nd_vaccination_arm_1"		
312	[nano_e_trombo]<br>Show the field ONLY if:<br>[event-name] = "screening_arm_1" OR [event-name] = "1st_vaccination_arm_1" OR [event-name] = "2nd_vaccination_arm_1"	Thrombocytopenia or any coagulation disorder	yesno, Required<br>1 Yes<br>0 No
313	[nano_e_pregnancy]<br>Show the field ONLY if:<br>[event-name] = "screening_arm_1" OR [event-name] = "1st_vaccination_arm_1" OR [event-name] = "2nd_vaccination_arm_1"	Pregnancy or lactating or childbearing potential (NON-use of an effective method of contraception* from at least 4 weeks prior to the first vaccination until at least 10 weeks after the last vaccination)*Postmenopausal for at least 1 year, surgically sterile (female or male partner), use of oral, injected or implanted hormonal methods of contraception, placement of an intrauterine device (IUD) or intrauterine system (IUS), barrier methods of contraception (condom or occlusive cap with spermicide), true abstinence	yesno, Required<br>1 Yes<br>0 No
314	[nano_e_other_clini_trial]<br>Show the field ONLY if:<br>[event-name] = "screening_arm_1" OR [event-name] = "1st_vaccination_arm_1" OR [event-name] = "2nd_vaccination_arm_1"	Participation in another clinical trial investigating a vaccine, drug, medical device or medical procedure in the 4 weeks preceding the first vaccination, or planned participation during the study period	yesno, Required<br>1 Yes<br>0 No
315	[nano_e_covid]<br>Show the field ONLY if:<br>[event-name] = "screening_arm_1" OR [event-name] = "1st_vaccination_arm_1" OR [event-name] = "2nd_vaccination_arm_1"	SARS-CoV-2 clinical disease confirmed by antigenic or PCR test in the 4 weeks preceding the first trial vaccination	yesno, Required<br>1 Yes<br>0 No
316	[nano_e_other_vaccine]<br>Show the field ONLY if:<br>[event-name] = "screening_arm_1" OR [event-name] = "1st_vaccination_arm_1" OR [event-name] = "2nd_vaccination_arm_1"	Receipt of any vaccine (including vaccination against COVID) in the 4 weeks preceding the trial vaccination (excepting influenza vaccination, which may be received up to 2 weeks before first study vaccine) or planned receipt of any vaccine in the 4 weeks following each trial vaccination	yesno, Required<br>1 Yes<br>0 No
317	[nano_e_immuno]<br>Show the field ONLY if:<br>[event-name] = "screening_arm_1" OR [event	Receipt of immunoglobulins, blood or blood-derived products in the past 3 months	yesno, Required<br>1 Yes<br>0 No

	-name] = "1st_vaccination_arm_1" OR [event-name] = "2nd_vaccination_arm_1"		
318	[nano_e_hiv]<br>Show the field ONLY if:<br>[event-name] = "screening_arm_1" OR [event-name] = "1st_vaccination_arm_1" OR [event-name] = "2nd_vaccination_arm_1"	Self-reported or documented seropositivity for human immunodeficiency virus (HIV), hepatitis B natural infection (HBcAb positive serology), or hepatitis C	yesno, Required<br>1 Yes<br>0 No
319	[nano_e_linked_w_study]<br>Show the field ONLY if:<br>[event-name] = "screening_arm_1" OR [event-name] = "1st_vaccination_arm_1" OR [event-name] = "2nd_vaccination_arm_1"	Identified as an Investigator or employee of the Investigator or study centre with direct involvement in the proposed study, or identified as an immediate family member (i.e., parent, spouse, natural or adopted child) of the Investigator or employee with direct involvement in the proposed study (i.e. in the employment of the Tropivac clinic or DFRI unit at Unisanté)	yesno, Required<br>1 Yes<br>0 No
320	[nano_e_refusal]<br>Show the field ONLY if:<br>[event-name] = "screening_arm_1" OR [event-name] = "1st_vaccination_arm_1" OR [event-name] = "2nd_vaccination_arm_1"	Refusal to be informed in the event that relevant results concerning the participant's health are revealed.	yesno, Required<br>1 Yes<br>0 No
321	[nano_e_not_enrolled_2]<br>Show the field ONLY if:<br>([nano_e_chronic] = '1' or [nano_e_addiction] = '1' or [nano_e_congenital] = '1' or [nano_e_hypersensitivity] = '1' or [nano_e_trombo] = '1' or [nano_e_pregnancy] = '1' or [nano_e_other_clini_trial] = '1' or [nano_e_other_vaccine] = '1' or [nano_e_immuno] = '1' or [nano_e_hiv] = '1' or [nano_e_linked_w_study] = '1' or [nano_e_refusal] = '1' or [nano_e_covid] = '1') and [event-name] <> '2nd_vaccination_arm_1'	The volunteer CAN NOT BE ENROLLED in the trial, please complete the SCREEN FAILURE page	descriptive
322	[nano_e_not_enrolled_5]	The volunteer IS NOT ELIGIBLE anymore. Please complete the END OF TRIAL page.	descriptive

	Show the field ONLY if: ([nano_e_chronic] = '1' or [nano_e_addiction] = '1' or [nano_e_congenital] = '1' or [nano_e_hypersensitivity] = '1' or [nano_e_trombo] = '1' or [nano_e_pregnancy] = '1' or [nano_e_other_clini_trial] = '1' or [nano_e_other_vaccine] = '1' or [nano_e_immuno] = '1' or [nano_e_hiv] = '1' or [nano_e_linked_w_study] = '1' or [nano_e_refusal] = '1' or [nano_e_covid] = '1') and [event-name] <> '2nd_vaccination_arm_1'		
323	[nano_e_text_3] Show the field ONLY if: [event-name] = 'screening_arm_1'	Pre-eligibility	descriptive
324	[nano_e_text_4] Show the field ONLY if: [event-name] = "1st_vaccination_arm_1" OR [event-name] = "2nd_vaccination_arm_1"	Exclusion criteria at the time of vaccination	descriptive
325	[nano_e_pre_date] Show the field ONLY if: [event-name] = "screening_arm_1"	Date of pre-eligibility verification	text (date_dmy), Required
326	[nano_e_pre] Show the field ONLY if: [event-name] = "screening_arm_1"	Is the volunteer pre-eligible for this trial?	radio, Required<br>a Yes<br>b Volunteer is definitely not eligible<br>c All data are NOT yet available and eligibility will be re-considered before vaccination.
327	[nano_e_prescription_form_2] Show the field ONLY if: [nano_e_pre] = "a" and [event-name] = "screening_arm_1"	Go to Prescription form	descriptive
328	[nano_e_not_enrolled_3] Show the field ONLY if: [nano_e_pre] = 'b'	Please complete the SCREEN FAILURE page	descriptive

329	[nano_e_temp_above_375]<br>Show the field ONLY if:<br>[event-name] = "1st_vaccination_arm_1" OR [event-name] = "2nd_vaccination_arm_1"	Does the patient have a temperature of >37.5°C today ?	yesno, Required<br>1 Yes<br>0 No
330	[nano_e_other_disease]<br>Show the field ONLY if:<br>[event-name] = "1st_vaccination_arm_1" OR [event-name] = "2nd_vaccination_arm_1"	Does the patient have a moderate or severe acute disease today ?	yesno, Required<br>1 Yes<br>0 No
331	[nano_e_other_disease_specify]<br>Show the field ONLY if:<br>[event-name] = "1st_vaccination_arm_1" and [nano_e_other_disease] = '1'	Specify	text, Required
332	[nano_e_temp_above_375_message]<br>Show the field ONLY if:<br>[nano_e_temp_above_375] = '1' or [nano_e_other_disease] = '1'	Vaccination is contraindicated today. Assess if the subject may be vaccinated at a later date, or complete the SCREEN FAILURE page	descriptive
333	[nano_e_final_eligibility]<br>Show the field ONLY if:<br>[event-name] = "1st_vaccination_arm_1"	Section Header: *Final Eligibility*<br>For the subjects who were pre-eligible at Visit 1, is the volunteer still eligible to continue with the trial?	yesno, Required<br>1 Yes<br>0 No
334	[nano_e_final_eligibility_2]<br>Show the field ONLY if:<br>[event-name] = "2nd_vaccination_arm_1"	For the subjects who were eligible at Visit 2, is the volunteer still eligible to continue with the trial?	yesno, Required<br>1 Yes<br>0 No
335	[nano_e_not_enrolled]<br>Show the field ONLY if:<br>[nano_e_final_eligibility] = '0' AND [event-name] <> 'screening_arm_1' AND [event-name] <> '1st_vaccination_arm_1'	Complete the END OF TRIAL FORM	descriptive
336	[nano_e_not_enrolled_6]	The volunteer IS NOT ELIGIBLE anymore. Complete the END OF TRIAL page.	descriptive

	Show the field ONLY if: [nano_e_final_eligibility_2] = '0'		
337	[nano_e_not_final_eligibility]<br>Show the field ONLY if: [event-name] = "1st_vaccination_arm_1"	For the subjects who were not yet eligible at Visit 1, is the volunteer now eligible?	yesno, Required<br>1 Yes<br>0 No
338	[nano_e_not_enrolled_4]<br>Show the field ONLY if: [nano_e_final_eligibility] = '0' OR [nano_e_not_final_eligibility] = '0'	The volunteer CAN NOT BE ENROLLED in the trial, please STOP and complete the SCREEN FAILURE page	descriptive
339	[nano_e_proceed]<br>Show the field ONLY if: [nano_e_final_eligibility] = '1' AND [nano_e_not_final_eligibility] = '1'	Proceed to vaccination	descriptive
340	[nano_e_proceed_2]<br>Show the field ONLY if: [nano_e_final_eligibility_2] = '1'	Proceed to vaccination n°2.	descriptive
341	[nano_e_still_eligible]<br>Show the field ONLY if: [event-name] <> "1st_vaccination_arm_1" AND [event-name] <> "screening_arm_1" and [event-name] <> "2nd_vaccination_arm_1"	Is the volunteer still eligible to continue with the trial ?	yesno, Required<br>1 Yes<br>0 No
342	[nano_e_prescription_form]<br>Show the field ONLY if: [nano_e_still_eligible] = "1" and [event-name] = "visit_5_arm_1"	Go to Prescription form	descriptive
343	[nano_e_button_end]<br>Show the field ONLY if: [nano_e_still_eligible] = "0"	Complete the END OF TRIAL FORM	descriptive
344	[eligibility_complete]	Section Header: *Form Status*<br>Complete?	dropdown<br>0 Incomplete<br>1 Unverified

			2 Complete

Instrument: Vaccine administration (vaccine_administration)

345	[dep_name11]	Research Physician	descriptive
346	[nano_va_been_vac]	Has the volunteer been vaccinated ?	yesno, Required<br>1 Yes<br>0 No
347	[nano_va_been_vac_spec]<br>Show the field ONLY if:<br>[nano_va_been_vac] = '0'	Specify	text, Required
348	[nano_va_vac_batch_number]<br>Show the field ONLY if:<br>[nano_va_been_vac] = '1'	Lot number	text, Required
349	[nano_va_vac_number]<br>Show the field ONLY if:<br>[nano_va_been_vac] = '1'	Randomization number	text (integer), Required
350	[nano_va_vac_dose]<br>Show the field ONLY if:<br>[nano_va_been_vac] = '1'	Dose	radio<br>LD Low dose<br>HD High dose
351	[nano_va_vac_date]<br>Show the field ONLY if:<br>[nano_va_been_vac] = '1'	Date and Time of vaccination	text (datetime_dmy)
352	[nano_va_vac_arm]<br>Show the field ONLY if:<br>[nano_va_been_vac] = '1'	Injection site : arm (intradermal)	radio<br>1 Left<br>2 Right
353	[nano_va_vac_problem]<br>Show the field ONLY if:<br>[nano_va_been_vac] = '1'	Any problem with the injection?	yesno<br>1 Yes<br>0 No
354	[nano_va_vac_problem_specify]<br>Show the field ONLY if:<br>[nano_va_vac_problem] = "1"	Specify*<br>**No papule, IMP leakage from the needle...*	text

355	[vaccine_administration_complete]	Section Header: *Form Status*<br>Complete?	dropdown<br>0 Incomplete<br>1 Unverified<br>2 Complete
**Instrument: 60 minutes post vaccination assessment (minutes_post_vaccination_assessment)**			
356	[dep_name12]	Study nurse - Refer to Research Physician if needed.	descriptive
357	[nano_60_post_axillary]	Section Header: *Physical observation*<br>Axillary temperature	text (number, Min: 33, Max: 43)
358	[nano_60_post_systolic]	Systolic blood pressure	text (integer, Min: 50, Max: 230)
359	[nano_60_post_diastolic]	Diastolic blood pressure	text (integer, Min: 20, Max: 150)
360	[nano_60_post_heart]	Heart rate	text (integer, Min: 35, Max: 150)
361	[nano_60_post_title_2]	Adverse events	descriptive
362	[nano_60_post_sae]	Is there any adverse event (SAE)?	yesno<br>1 Yes<br>0 No
363	[post_sae_def]		descriptive<br>(Attachment: Definition_Serious Adverse Event.pdf, Display format: Link)
364	[nano_60_post_sae_y]<br>Show the field ONLY if:<br>[nano_60_post_sae] = "1"	Please fill a SAE form	descriptive
365	[nano_60_post_pain]	Section Header: *Solicited adverse event at injection site*<br>Pain	yesno<br>1 Yes<br>0 No
366	[nano_60_post_tenderness]	Tenderness	yesno<br>1 Yes<br>0 No
367	[nano_60_post_erythema]	Erythema	yesno<br>1 Yes<br>0 No
368	[nano_60_post_swelling]	Swelling	yesno<br>1 Yes<br>0 No
369	[nano_60_post_sae_y_2]<br>Show the field ONLY if:<br>[nano_60_post_pain] = '1' OR [nano_60_post_tenderness] = '1' OR [nano_60_post_erythem	Please fill a SAE form - Please fill a AE form	descriptive

	a] = '1' OR [nano_60_post_swelling] = '1'		
370	[nano_60_post_any_unsolicited]	Section Header: *Unsolicited adverse event at injection site*<br>Is there any unsolicited adverse event at injection site?	yesno<br>1 Yes<br>0 No
371	[nano_60_post_sae_y_4]<br>Show the field ONLY if:<br>[nano_60_post_any_unsolicited] = '1'	Please fill a SAE form - Please fill a AE form	descriptive
372	[nano_60_post_nausea]	Section Header: *Solicited systemic adverse event*<br>Nausea/vomiting	yesno<br>1 Yes<br>0 No
373	[nano_60_post_diarrhea]	Diarrhea	yesno<br>1 Yes<br>0 No
374	[nano_60_post_headache]	Headache	yesno<br>1 Yes<br>0 No
375	[nano_60_post_asthenia]	Fatigue	yesno<br>1 Yes<br>0 No
376	[nano_60_post_myalgia]	Myalgia	yesno<br>1 Yes<br>0 No
377	[nano_60_post_fever]	Fever	yesno<br>1 Yes<br>0 No
378	[nano_60_post_axillary_temp]<br>Show the field ONLY if:<br>[nano_60_post_fever] = "1"	Axillary temperature	text
379	[nano_60_post_sae_y_3]<br>Show the field ONLY if:<br>[nano_60_post_headache] = '1' or [nano_60_post_myalgia] = '1' or [nano_60_post_asthenia] = '1' or [nano_60_post_fever] = '1'	Please fill a SAE form - Please fill a AE form	descriptive
380	[nano_60_post_any_unsolicited_system]	Section Header: *Unsolicited systemic adverse event*<br>Is there any unsolicited systemic adverse event?	yesno<br>1 Yes

			0 No
381	[nano_60_post_sae_y_5]<br>Show the field ONLY if:<br>[nano_60_post_any_unsolicited_system] = '1'	Please fill a SAE form - Please fill a AE form	descriptive
382	[minutes_post_vaccination_assessment_complete]	Section Header: *Form Status*<br>Complete?	dropdown<br>0 Incomplete<br>1 Unverified<br>2 Complete
**Instrument: Diary card (diary_card)**			
383	[dep_name17]<br>Show the field ONLY if:<br>[event-name] = "screening_arm_1" OR [event-name] = "1st_vaccination_arm_1" OR [event-name] = "2nd_vaccination_arm_1"	Study nurse	descriptive
384	[nano_d_collect]<br>Show the field ONLY if:<br>[event-name] = "visit_5_arm_1" OR [event-name] = "visit_9_arm_1"	Collect the diary card	descriptive
385	[nano_d_diary_card_given]<br>Show the field ONLY if:<br>[event-name] = "1st_vaccination_arm_1"	Diary card given to the subject	yesno<br>1 Yes<br>0 No
386	[nano_d_specify]<br>Show the field ONLY if:<br>[nano_d_diary_card_given] = '0'	Specify why	text
387	[nano_d_diary_card_given_2]<br>Show the field ONLY if:<br>[event-name] = "2nd_vaccination_arm_1"	Diary card n°2 given to the subject	yesno<br>1 Yes<br>0 No
388	[nano_d_specify_2]<br>Show the field ONLY if:<br>[nano_d_diary_card_given_2] = '0'	Specify why	text
389	[nano_d_thermometer_given]	Thermometer given to te subject	yesno<br>1 Yes

	Show the field ONLY if: [event-name] = "1st_vaccination_arm_1"		0 No
390	[nano_d_ruler_given] Show the field ONLY if: [event-name] = "1st_vaccination_arm_1"	Ruler given to the subject	yesno<br>1 Yes<br>0 No
391	[nano_d_text] Show the field ONLY if: [event-name] <> 'visit_5_arm_1' and [event-name] <> 'visit_9_arm_1'	Remind the subject to measure axillary temperature, erythema or swelling at the injection site, to record adverse events and to bring daily diary at next visit.	descriptive
392	[diary_card_complete]	Section Header: *Form Status*<br>Complete?	dropdown<br>0 Incomplete<br>1 Unverified<br>2 Complete
**Instrument: Protocol deviation (protocol_deviation)**			
393	[dep_name13] Show the field ONLY if: [event-name] <> "screening_arm_1" OR [event-name] = "1st_vaccination_arm_1" OR [event-name] = "2nd_vaccination_arm_1"	Study nurse - Refer to the Research Physician if needed.	descriptive
394	[nano_e_protocol_deviation]	Section Header: *Protocol deviation*<br>Is there a protocol deviation ?	yesno<br>1 Yes<br>0 No
395	[nano_pd_button] Show the field ONLY if: [nano_e_protocol_deviation] = "1"	Please fill the minor protocol deviation form	descriptive
396	[nano_pd_button_2] Show the field ONLY if: [nano_e_protocol_deviation] = "1"	Please fill the major protocol deviation form	descriptive
397	[protocol_deviation_complete]	Section Header: *Form Status*<br>Complete?	dropdown<br>0 Incomplete<br>1 Unverified<br>2 Complete
**Instrument: Additionnal comment (additionnal_comment)**			
398	[dep_name14]	Section Header: *Additionnal comment*<br>Study Nurse	descriptive

	Show the field ONLY if: [event-name] = "screening_arm_1" OR [event-name] = "1st_vaccination_arm_1" OR [event-name] = "2nd_vaccination_arm_1"		
399	[nano_ac_add_comment]	Any additional comment ?	yesno<br>1 Yes<br>0 No
400	[nano_ac_add_comment_prec]<br>Show the field ONLY if: [nano_ac_add_comment] = '1'	Specify	notes
401	[additionnal_comment_complete]	Section Header: *Form Status*<br>Complete?	dropdown<br>0 Incomplete<br>1 Unverified<br>2 Complete
**Instrument: Compensation (compensation_43a693)**			
402	[nano_ac_chf]<br>Show the field ONLY if: [event-name]='1st_vaccination_arm_1' OR [event-name]='visit_4_arm_1' OR [event-name]='visit_5_arm_1' OR [event-name]='2nd_vaccination_arm_1' OR [event-name]='visit_8_arm_1' OR [event-name]='visit_9_arm_1' OR [event-name]='visit_11_arm_1' OR [event-name]='visit_12_arm_1'	Section Header: *Compensation*<br>Compensation given (100CHF) ?	yesno<br>1 Yes<br>0 No
403	[nano_ac_chf_date]<br>Show the field ONLY if: [nano_ac_chf] = '1'	Date	text (date_dmy)
404	[nano_ac_chf_reason]<br>Show the field ONLY if: [nano_ac_chf] = '0'	If no, why ?	text
405	[compensation_43a693_complete]	Section Header: *Form Status*<br>Complete?	dropdown<br>0 Incomplete<br>1 Unverified<br>2 Complete
**Instrument: Next visit (next_visit)**			

406	[dep_name15]<br>Show the field ONLY if:<br>[event-name] = "screening_arm_1" OR [event-name] = "1st_vaccination_arm_1" OR [event-name] = "2nd_vaccination_arm_1"	Section Header: *Next Visit*<br>Study nurse	descriptive
407	[nano_nv_date_next_visit]	Date of next visit	text (datetime_dmy)
408	[nano_nv_send_sms]	Send the reminder by SMS ?	yesno<br>1 Yes<br>0 No
409	[next_visit_complete]	Section Header: *Form Status*<br>Complete?	dropdown<br>0 Incomplete<br>1 Unverified<br>2 Complete
**Instrument: Adverse Event (adverse_event)**			
410	[nano_ae_custom_record_id]	Custom record id	text, Required<br>Field Annotation: @HIDDEN @HIDDEN-PDF
411	[nano_ae_number]	Adverse Event Number	text, Required
412	[nano_ae_id]	Inclusion ID	text, Required
413	[nano_ae_date_container]	Date of onset {nano_ae_date} {nano_ae_date_unknown}	descriptive
414	[nano_ae_date_unknown]	Date unknown	checkbox<br>1 nano_ae_date_unknown___1 Date unknown
415	[nano_ae_date]	Date of onset	text (date_dmy)
416	[nano_ae_term]	Section Header: *Adverse Event Description*<br>AE term	text
417	[nano_ae_description]	Adverse Event description	notes, Required
418	[nano_ae_sae]	Is this a serious adverse event* ?	yesno, Required<br>1 Yes<br>0 No
419	[nano_ae_sae_definition]		descriptive<br>(Attachment: Definition_Serious Adverse Event.pdf, Display format: Link)
420	[nano_ae_sae_todo]<br>Show the field ONLY if:<br>[nano_ae_sae] = "1"	Please complete a SAE form and do not complete this AE form.<br>SAE form	descriptive
421	[nano_ae_solicited]	Which type of AE is it ?	radio, Required<br>1 Solicited AE<br>2 Unsolicited AE related with an abnormal laboratory value

			3 Unsolicited AE
422	[nano_ae_toxicity] Show the field ONLY if: [nano_ae_solicited] = "1"	FDA toxicity grading scale term	dropdown, Required<br>1 Pain<br>2 Tenderness<br>3 Erythema/Redness<br>4 Induration/Swelling<br>5 Nausae/Vomiiting<br>6 Diarrhea<br>7 Headache<br>8 Fatigue<br>9 Myalgia<br>10 Fever
423	[nano_ae_fda_tox_grade_2] Show the field ONLY if: [nano_ae_solicited] = "1"	AE grade according to adapted FDA toxicity grading scale	radio, Required<br>1 Grade 1<br>2 Grade 2<br>3 Grade 3
424	[nano_ae_call_doctor] Show the field ONLY if: [nano_ae_solicited] = "2" OR [nano_ae_solicited] = "3" OR [nano_ae_fda_tox_grade_2] = "2" OR [nano_ae_fda_tox_grade_2] = "3"	Call the research physician. The study nurse can only complete AEs forms for solicited local grade 1 AEs.	descriptive
425	[nano_ae_fda_tox_grade] Show the field ONLY if: [nano_ae_solicited] = "1"		descriptive (Attachment: naNO-COVID_FDA_adapted (4).pdf, Display format: Link)
426	[nano_ae_ctcae_6] Show the field ONLY if: [nano_ae_solicited] = "2"	AE grade according to adapted CTCAE v5.0	radio, Required<br>1 Grade 1<br>2 Grade 2<br>3 Grade 3<br>4 Grade 4<br>5 Grade 5
427	[nano_ae_ctcae_grade_v7] Show the field ONLY if: [nano_ae_solicited] = "2"		descriptive (Attachment: naNO-COVID_CTCAE_adapted_lab (2).pdf, Display format: Link)
428	[nano_ae_ctcae]	CTCAE term for grading AE	text, Required<br>BIOPORTAL:CTCAE BIOPORTAL:CTCAE

	Show the field ONLY if: [nano_ae_solicited] = "3"		
429	[nano_ae_spec_ctcae] Show the field ONLY if: [nano_ae_solicited] = "3"	If needed, please specify	text, Required
430	[nano_ae_ctcae_5] Show the field ONLY if: [nano_ae_solicited] = "3"	AE grade according to CTCAE v5.0	radio, Required<br>1 Grade 1<br>2 Grade 2<br>3 Grade 3<br>4 Grade 4<br>5 Grade 5
431	[nano_p_ae_interest]	Is this an adverse event of special interest ?	yesno, Required<br>1 Yes<br>0 No
432	[nano_aesi_def]		descriptive<br>(Attachment: Definition of Adverse Event of Special Interest.pdf, Display format: Link)
433	[nano_ae_sae_info] Show the field ONLY if: [nano_p_ae_interest] = "1" OR [nano_ae_ctcae_5] = "3" OR [nano_ae_ctcae_5]= "4" OR [nano_ae_ctcae_5]= "5"	Please complete a SAE form and do not complete this AE form.<br>SAE form	descriptive
434	[nano_ae_causality]	Causality to the study vaccine	radio, Required<br>1 No relationship<br>2 Possible<br>3 Probable<br>4 Definite
435	[nano_ae_causality_2]		descriptive<br>(Attachment: Guidelines for assessing the relationship of vaccine administration to an adverse event (2).pdf, Display format: Link)
436	[nano_ae_new_event]	Already experienced this event prior to study enrollment?	radio, Required<br>1 Yes<br>2 No<br>3 Not known
437	[nano_ae_comment_event] Show the field ONLY if:	Please comment	text, Required

	[nano_ae_new_event] = "1"		
438	[nano_ae_doses]	Section Header: *IMP 1st vaccine dose +/- 2nd vaccine dose*<br>Were one or two doses administred ?	radio, Required<br>1 One<br>2 Two
439	[nano_ae_random_number]	Randomization number	text (integer), Required
440	[nano_ae_code_broken]	Was the code broken ?	yesno, Required<br>1 Yes<br>0 No
441	[nano_ae_detail]<br>Show the field ONLY if:<br>[nano_ae_code_broken] = "1"	Please detail the reason for unblinding, the person who proceeded to unblinding and the date.	notes, Required
442	[nano_ae_imp_name]	Name of IMP : PepGNP-COVID / Base-GNP	descriptive
443	[nano_ae_dose]	Dose	radio, Required<br>1 Low dose (14.8 ug of gold nanoparticules +/- 2.5 nmol dengue peptides)<br>2 High dose (44.5 ug of gold nanoparticules +/- 7.5 nmol dengue peptides)
444	[nano_ae_route]	Route : Intradermal injection	descriptive
445	[nano_ae_date_administred]	Administration date dose 1	text (date_dmy), Required
446	[nano_ae_date_administred_2]<br>Show the field ONLY if:<br>[nano_ae_doses] = "2"	Administration date dose 2	text (date_dmy), Required
447	[nano_ae_tests_performed]	Section Header: *Action taken*<br>Have some paraclinical tests been performed ?	yesno, Required<br>1 Yes<br>0 No
448	[nano_ae_test_labo]<br>Show the field ONLY if:<br>[nano_ae_tests_performed] = "1"	Laboratory tests	yesno, Required<br>1 Yes<br>0 No
449	[nano_ae_test_radio]<br>Show the field ONLY if:<br>[nano_ae_tests_performed] = "1"	Radiological exams	yesno, Required<br>1 Yes<br>0 No
450	[nano_ae_main_result]<br>Show the field ONLY if:	Please provide the main results of the exams performed	notes, Required

	[nano_ae_tests_performed] = "1"		
451	[nano_ae_do_not_forget]<br>Show the field ONLY if:<br>[nano_ae_test_radio] = "1" OR [nano_ae_test_labo] = "1" OR [nano_ae_tests_performed] = "1"	Do not forget to collect complementary exam, remove identifying data, code them and register them as source documents.	descriptive
452	[nano_ae_action_taken_imp]	Which action(s) was/were taken with IMP ? (Several possible)	checkbox, Required<br>a | nano_ae_action_taken_imp___a | No acti<br>b | nano_ae_action_taken_imp___b | Dose n change<br>c | nano_ae_action_taken_imp___c | Dose reduce<br>d | nano_ae_action_taken_imp___d | Dose increas<br>e | nano_ae_action_taken_imp___e | Drug tempo interru<br>f | nano_ae_action_taken_imp___f | Drug withra
453	[nano_ae_action_taken]	Which action(s) was/were taken with the participant? (Several possible)	checkbox, Required<br>7 | nano_ae_action_taken___7 | Medicatic treatmen<br>8 | nano_ae_action_taken___8 | Non medicatic treatmen<br>9 | nano_ae_action_taken___9 | Hospitalis<br>10 | nano_ae_action_taken___10 | No action
454	[nano_ae_specify]<br>Show the field ONLY if:<br>[nano_ae_action_taken(8)]	Specify	text, Required
455	[nano_ae_text]<br>Show the field ONLY if:<br>[nano_ae_action_taken(7)]	Please fill the medication form	descriptive
456	[nano_ae_reaction_reappear]	Did the reaction reappear after the second dose of the vaccination ?	radio, Required<br>1 | Yes<br>2 | No<br>3 | Not applicable
457	[nano_ae_text_sae]<br>Show the field ONLY if:	Fill the SAE form	descriptive

	[nano_ae_action_taken (9)]		
458	[nano_ae_withdrawn]	Withdrawn from the study because of this AE ?	yesno, Required<br>1 Yes<br>0 No
459	[nano_end_of_trial]<br>Show the field ONLY if:<br>[nano_ae_withdrawn] = "1"	Complete the END OF TRIAL FORM	descriptive
460	[nano_ae_outcome]	Section Header: *Outcome*<br>Outcome of the adverse event	radio, Required<br>1 Resolved (for date, last day of symptoms)<br>2 Resolved with sequelae<br>3 Ongoing<br>4 Stabilisation<br>5 Improvement<br>6 Agravation<br>7 Fatal<br>8 Unknown
461	[nano_ae_date_outcome]	Date of the outcome evaluation	text (date_dmy)
462	[nano_ae_last_day_sympt]<br>Show the field ONLY if:<br>[nano_ae_outcome] = '1' or [nano_ae_outcome] = '2'	Date of resolution	text (date_dmy), Required
463	[nano_ae_death_date]<br>Show the field ONLY if:<br>[nano_ae_outcome] = '7'	Date of death	text (date_dmy), Required
464	[nano_ae_evolved]	Has this AE evolved in a SAE/AESI/SUSAR ?	yesno, Required<br>1 Yes<br>0 No
465	[nano_ae_sae_number]<br>Show the field ONLY if:<br>[nano_ae_evolved] = "1"	Specify the SAE number related	text, Required
466	[nano_ae_text_sae_2]<br>Show the field ONLY if:<br>[nano_ae_evolved] = "1"	Fill the SAE form	descriptive
467	[nano_ae_comment]	Add comments	yesno, Required

			1 Yes<br>0 No
468	[nano_ae_comments]<br>Show the field ONLY if:<br>[nano_ae_comment] = "1"	Comments	notes, Required
469	[adverse_event_complete]	Section Header: *Form Status*<br>Complete?	dropdown<br>0 Incomplete<br>1 Unverified<br>2 Complete
**Instrument: Screen Failure (screen_failure)**			
470	[nano_sf_date]	Date of screenfailure (if applicable)	text (date_dmy)
471	[nano_sf_date_last_contact]	Date of last contact	text (date_dmy)
472	[nano_sf_specify]	Specify reason for screen failure	radio<br>a Age < 18 or > 45 years<br>b Written consent not provided/consent withdrawal<br>c Not able/do not want to attend all scheduled visit/to comply with all study requirments<br>d Chronic illness that might interfere with trial conduct<br>e Current alcohol abuse or drug addiction (reported or suspected)<br>f Known or suspected congenital or acquired immunodeficiency; or receipt of immunosuppressive therapy<br>g Known systemic hypersensitivity to any of the vaccine components (e.g. gold), or history of a life-threatening heaction to vaccines<br>h Thrombocytopenia or any coagulation disorder<br>i Pregnancy or lactating<br>j Non-use of an effective method of contraception from at least 4 weeks prior to the first vaccination<br>k Participation in another clinical trial investigating a vaccine, drug, medical device or medical procedure in the 4 weeks preceding the first vaccination, or planned participation during the study period<br>l Receipt of any vaccine in the 4 weeks preceding the trial vaccination (excepting influenza vaccination, which may be received

			up to 2 weeks before first study vaccine) or planned receipt of any vaccine in the 4 weeks following trial vaccination<br>m Receipt of immunoglobulins, blood or blood-derived products in the past 3 months<br>n Positive SARS-CoV-2 test in the 4 weeks preceding the first trial vaccination<br>p Self-reported or documented seropositivity for human immunodeficiency virus (HIV), hepatitis B natural infection (HBcAb positive serology), or hepatitis C<br>s Identified as an Investigator or employee of the Investigator or study centre with direct involvement in the proposed study, or identified as an immediate family member (i.e., parent, spouse, natural or adopted child) of the Investigator or employee with direct involvement in the proposed study<br>t Refusal to be informed in the event that relevant results concerning the participant's health are revealed.<br>u Abnormal laboratory (blood/urine) value<br>v Lost to follow up<br>w Other
473	[nano_sf_specify_fail]<br><br>Show the field ONLY if:<br>[nano_sf_specify] = "c" or [nano_sf_specify] = "d" or [nano_sf_specify] = "f" or [nano_sf_specify] = "g" or [nano_sf_specify] = "h" or [nano_sf_specify] = "j" or [nano_sf_specify] = "l" or [nano_sf_specify] = "m" or [nano_sf_specify] = "m" or [nano_sf_specify] = "o" or [nano_sf_specify] = "p" or [nano_sf_specify] = "q" or [nano_sf_specify] = "r" or [nano_sf_specify] = "u" or [nano_sf_specify] = "v" or [nano_sf_specify] = "w"	Specify	text
474	[nano_sf_comment_yn]	Any additional comment?	yesno

			1 Yes<br>0 No
475	[nano_sf_comment]<br>Show the field ONLY if:<br>[nano_sf_comment_yn] = "1"	Specify	notes
476	[screen_failure_complete]	Section Header: *Form Status*<br>Complete?	dropdown<br>0 Incomplete<br>1 Unverified<br>2 Complete
**Instrument: Unscheduled visit (unscheduled_visit)**			
477	[nano_uv_date_visit]	Section Header: *Reason of visit*<br>Date of visit	text (date_dmy)
478	[nano_uv_reason]	Reason of visit	radio<br>a New adverse event<br>b Follow up of an adverse event<br>c Other
479	[nano_uv_reason_specify]<br>Show the field ONLY if:<br>[nano_uv_reason] = "c"	Specify	notes
480	[nano_uv_number]<br>Show the field ONLY if:<br>[nano_uv_reason] = "a" or [nano_uv_reason] = "b"	Event number	sql<br>SELECT instance, value from redcap_data where project_id = 341 and field_name = 'nano_ae_number' and record = [record-name] order by value
481	[nano_uv_med_event]	Section Header: *Medical history*<br>Any relevant medical event since the last visit ?	yesno<br>1 Yes<br>0 No
482	[nano_uv_event_med_specify]<br>Show the field ONLY if:<br>[nano_uv_med_event] = "1"	Specify	notes
483	[nano_uv_new_ae]<br>Show the field ONLY if:<br>[event-name] <> 'visit_11_arm_1' and [event-name] <> 'visit_12_arm_1'	Is there any new adverse event ?	yesno, Required<br>1 Yes<br>0 No
484	[nano_sae_button_7]<br>Show the field ONLY if:	Please fill a AE form	descriptive

	[nano_uv_new_ae] = '1'		
485	[nano_uv_new_sae]	Is there any serious adverse event (SAE) since last visit?	yesno, Required<br>1 Yes<br>0 No
486	[nano_sae_definition_2]		descriptive<br>(Attachment: Definition_Serious Adverse Event.pdf, Display format: Link)
487	[nano_sae_button_8]<br>Show the field ONLY if:<br>[nano_uv_new_sae] = '1'	Please fill a SAE form	descriptive
488	[nano_uv_med_change]	Section Header: *Current medication*<br>Is there any new or changed medication since the last visit?	yesno<br>1 Yes<br>0 No
489	[nano_uv_med_form]<br>Show the field ONLY if:<br>[nano_uv_med_change] = "1"	Please fill the medication form	descriptive
490	[nano_uv_vac]	Is there any vaccination since the last visit?	yesno<br>1 Yes<br>0 No
491	[nano_uv_vaccine_name]<br>Show the field ONLY if:<br>[nano_uv_vac] = "1"	Specify vaccine name	text
492	[nano_uv_vaccine_date]<br>Show the field ONLY if:<br>[nano_uv_vac] = "1"	Date of vaccination	text (date_dmy)
493	[nano_uv_physical_exam]	Section Header: *Physical exam*<br>Has a physical examination been done ?	yesno<br>1 Yes<br>0 No
494	[nano_uv_phy_exam_specify]<br>Show the field ONLY if:<br>[nano_uv_physical_exam] = "1"	Specify why	text
495	[nano_uv_body_weight]<br>Show the field ONLY if:<br>[nano_uv_physical_exam] = "1"	Section Header: *Physical observations*<br>Body weight	text (integer, Min: 0, Max: 150)
496	[nano_uv_axillary_t]	Axillary temperature	text (number_1dp, Min: 00, Max: 42)

	Show the field ONLY if: [nano_uv_physical_exam] = "1"		
497	[nano_uv_heart_rate] Show the field ONLY if: [nano_uv_physical_exam] = "1"	Heart rate	text (integer, Min: 00, Max: 150)
498	[nano_uv_systolic_pressure] Show the field ONLY if: [nano_uv_physical_exam] = "1"	Systolic blood pressure	text (integer, Min: 00, Max: 230)
499	[nano_uv_diastolic_pressure] Show the field ONLY if: [nano_uv_physical_exam] = "1"	Diastolic blood pressure	text (integer, Min: 00, Max: 150)
500	[nano_uv_cardiovascular] Show the field ONLY if: [nano_uv_physical_exam] = "1"	Section Header: *Physical examination* Cardiovascular	notes
501	[nano_uv_respiratory] Show the field ONLY if: [nano_uv_physical_exam] = "1"	Respiratory	notes
502	[nano_uv_gastro_intestinal] Show the field ONLY if: [nano_uv_physical_exam] = "1"	Gastro-intestinal	notes
503	[nano_uv_dermatological] Show the field ONLY if: [nano_uv_physical_exam] = "1"	Dermatological	notes
504	[nano_uv_others] Show the field ONLY if: [nano_uv_physical_exam] = "1"	Other(s) : Specify	notes

505	[nano_uv_paraclinical_perf]	Section Header: *Action taken*<br>Have some paraclinical tests been performed ?	yesno<br>1 Yes<br>0 No
506	[nano_uv_labo_tests]<br>Show the field ONLY if:<br>[nano_uv_paraclinical_perf] = "1"	Laboratory tests*	yesno<br>1 Yes<br>0 No
507	[nano_uv_which_test]<br>Show the field ONLY if:<br>[nano_uv_labo_tests] = "1"	What test has been done ?	checkbox<br>1 nano_uv_which_test___1 Hemoglobi<br>2 nano_uv_which_test___2 Hematocrit<br>3 nano_uv_which_test___3 Erythrocyte<br>4 nano_uv_which_test___4 MCV (Mean corpuscular volume)<br>5 nano_uv_which_test___5 MCH (Mean cell hemoglobi<br>6 nano_uv_which_test___6 MCHC (Mea corpuscular hemoglobi concentrati<br>7 nano_uv_which_test___7 Platelets<br>8 nano_uv_which_test___8 Leukocytes<br>9 nano_uv_which_test___9 Neutrophils<br>10 nano_uv_which_test___10 Lymphocyte<br>11 nano_uv_which_test___11 Monocytes<br>12 nano_uv_which_test___12 Eosinophils<br>13 nano_uv_which_test___13 Basophils<br>15 nano_uv_which_test___15 Creatinine<br>16 nano_uv_which_test___16 AST<br>17 nano_uv_which_test___17 ALT<br>18 nano_uv_which_test___18 Alkaline phosphatas<br>19 nano_uv_which_test___19 GGT<br>20 nano_uv_which_test___20 Total bilirub<br>21 nano_uv_which_test___21 C reactive protein<br>23 nano_uv_which_test___23 Urine dipsti<br>24 nano_uv_which_test___24 Pregnancy test<br>22 nano_uv_which_test___22 Other
508	[nano_uv_leukocytes]<br>Show the field ONLY if:<br>[nano_uv_which_test(8)]	Leukocytes<br>*G/l - REF RANGE: 4 - 10*	text

509	[nano_uv_oor] Show the field ONLY if: [nano_uv_which_test(8)]	Is this value out of range?	yesno<br>1 Yes<br>0 No
510	[nano_uv_oor_deviation] Show the field ONLY if: [nano_uv_oor] = '1'	Is it a deviation listed here or a clinically significant deviation from normal ranges?	yesno<br>1 Yes<br>0 No
511	[nano_uv_erythrocytes] Show the field ONLY if: [nano_uv_which_test(3)]	Erythrocytes<br>*T/l - REF RANGE: 4.4 - 5.8 (Male) - 3.8 - 5.2 (Female)*	text
512	[nano_uv_oor_2] Show the field ONLY if: [nano_uv_which_test(3)]	Is this value out of range?	yesno<br>1 Yes<br>0 No
513	[nano_uv_oor_deviation_2] Show the field ONLY if: [nano_uv_oor_2] = '1'	Is it a deviation listed here or a clinically significant deviation from normal ranges?	yesno<br>1 Yes<br>0 No
514	[nano_uv_hemoglobin] Show the field ONLY if: [nano_uv_which_test(1)]	Hemoglobin<br>*g/l - REF RANGE: 133 - 177 (male) - 117 - 157 (female)*	text
515	[nano_uv_oor_3] Show the field ONLY if: [nano_uv_which_test(1)]	Is this value out of range?	yesno<br>1 Yes<br>0 No
516	[nano_uv_oor_deviation_3] Show the field ONLY if: [nano_uv_oor_3] = '1'	Is it a deviation listed here or a clinically significant deviation from normal ranges?	yesno<br>1 Yes<br>0 No
517	[nano_uv_hematocrit] Show the field ONLY if: [nano_uv_which_test(2)]	Hematocrit<br>*% - REF RANGE: 40 - 52 (male) - 35 - 47 (female)*	text
518	[nano_uv_oor_4] Show the field ONLY if: [nano_uv_which_test(2)]	Is this value out of range?	yesno<br>1 Yes<br>0 No

519	[nano_uv_oor_deviation_4]<br>Show the field ONLY if:<br>[nano_uv_oor_4] = '1'	Is it a deviation listed here or a clinically significant deviation from normal ranges?	yesno<br>1 Yes<br>0 No
520	[nano_uv_mcv]<br>Show the field ONLY if:<br>[nano_uv_which_test(4)]	MCV (Mean corpuscular volume)<br>*fl - REF RANGE: 81 - 99*	text
521	[nano_uv_oor_5]<br>Show the field ONLY if:<br>[nano_uv_which_test(4)]	Is this value out of range?	yesno<br>1 Yes<br>0 No
522	[nano_uv_oor_deviation_5]<br>Show the field ONLY if:<br>[nano_uv_oor_5] = '1'	Is it a deviation listed here or a clinically significant deviation from normal ranges?	yesno<br>1 Yes<br>0 No
523	[nano_uv_mch]<br>Show the field ONLY if:<br>[nano_uv_which_test(5)]	MCH (Mean cell hemoglobin)<br>*pg - REF RANGE: 27 - 34*	text
524	[nano_uv_oor_6]<br>Show the field ONLY if:<br>[nano_uv_which_test(5)]	Is this value out of range?	yesno<br>1 Yes<br>0 No
525	[nano_uv_oor_deviation_6]<br>Show the field ONLY if:<br>[nano_uv_oor_6] = '1'	Is it a deviation listed here or a clinically significant deviation from normal ranges?	yesno<br>1 Yes<br>0 No
526	[nano_uv_mchc]<br>Show the field ONLY if:<br>[nano_uv_which_test(6)]	MCHC (Mean corpuscular hemoglobin concentration)<br>*g/l - REF RANGE: 310 - 360*	text
527	[nano_uv_oor_7]<br>Show the field ONLY if:<br>[nano_uv_which_test(6)]	Is this value out of range?	yesno<br>1 Yes<br>0 No
528	[nano_uv_oor_deviation_7]<br>Show the field ONLY if:<br>[nano_uv_oor_7] = '1'	Is it a deviation listed here or a clinically significant deviation from normal ranges?	yesno<br>1 Yes<br>0 No
529	[nano_uv_platelets]	Platelets<br>*G/l - REF RANGE: 150 - 350*	text

	Show the field ONLY if: [nano_uv_which_test(7)]		
530	[nano_uv_oor_8] Show the field ONLY if: [nano_uv_which_test(7)]	Is this value out of range?	yesno<br>1 Yes<br>0 No
531	[nano_uv_oor_deviation_8] Show the field ONLY if: [nano_uv_oor_8] = '1'	Is it a deviation listed here or a clinically significant deviation from normal ranges?	yesno<br>1 Yes<br>0 No
532	[nano_uv_neutrophils] Show the field ONLY if: [nano_uv_which_test(9)]	Neutrophils<br>*G/l - REF RANGE: 1.8 - 7.5*	text
533	[nano_uv_oor_9] Show the field ONLY if: [nano_uv_which_test(9)]	Is this value out of range?	yesno<br>1 Yes<br>0 No
534	[nano_uv_oor_deviation_9] Show the field ONLY if: [nano_uv_oor_9] = '1'	Is it a deviation listed here or a clinically significant deviation from normal ranges?	yesno<br>1 Yes<br>0 No
535	[nano_uv_lymphocytes] Show the field ONLY if: [nano_uv_which_test(10)]	Lymphocytes<br>*G/l - REF RANGE: 1.5 - 4*	text
536	[nano_uv_oor_10] Show the field ONLY if: [nano_uv_which_test(10)]	Is this value out of range?	yesno<br>1 Yes<br>0 No
537	[nano_uv_oor_deviation_10] Show the field ONLY if: [nano_uv_oor_10] = '1'	Is it a deviation listed here or a clinically significant deviation from normal ranges?	yesno<br>1 Yes<br>0 No
538	[nano_uv_monocytes] Show the field ONLY if: [nano_uv_which_test(11)]	Monocytes<br>*G/l - REF RANGE: 0.2 - 0.8*	text
539	[nano_uv_oor_11]	Is this value out of range?	yesno

	Show the field ONLY if: [nano_uv_which_test(11)]		1 Yes<br>0 No
540	[nano_uv_oor_deviation_11]<br>Show the field ONLY if: [nano_uv_oor_11] = '1'	Is it a deviation listed here or a clinically significant deviation from normal ranges?	yesno<br>1 Yes<br>0 No
541	[nano_uv_eosinophils]<br>Show the field ONLY if: [nano_uv_which_test(12)]	Eosinophils<br>*G/l - REF RANGE: 0.05 - 0.5*	text
542	[nano_uv_oor_12]<br>Show the field ONLY if: [nano_uv_which_test(12)]	Is this value out of range?	yesno<br>1 Yes<br>0 No
543	[nano_uv_oor_deviation_12]<br>Show the field ONLY if: [nano_uv_oor_12] = '1'	Is it a deviation listed here or a clinically significant deviation from normal ranges?	yesno<br>1 Yes<br>0 No
544	[nano_uv_basophils]<br>Show the field ONLY if: [nano_uv_which_test(13)]	Basophils<br>*G/l - REF RANGE: 0.01 - 0.05*	text
545	[nano_uv_oor_13]<br>Show the field ONLY if: [nano_uv_which_test(13)]	Is this value out of range?	yesno<br>1 Yes<br>0 No
546	[nano_uv_oor_deviation_13]<br>Show the field ONLY if: [nano_uv_oor_13] = '1'	Is it a deviation listed here or a clinically significant deviation from normal ranges?	yesno<br>1 Yes<br>0 No
547	[nano_uv_creatinine]<br>Show the field ONLY if: [nano_uv_which_test(15)]	Creatinine<br>*µmol/ - REF RANGE: 62-106 (male) - 44 - 80 (female)*	text
548	[nano_uv_oor_14]<br>Show the field ONLY if: [nano_uv_which_test(15)]	Is this value out of range?	yesno<br>1 Yes<br>0 No
549	[nano_uv_oor_deviation_14]	Is it a deviation listed here or a clinically significant deviation from normal ranges?	yesno<br>1 Yes

	Show the field ONLY if: [nano_uv_oor_14] = '1'		0 No
550	[nano_uv_ast] Show the field ONLY if: [nano_uv_which_test(16)]	AST *UI/l - REF RANGE: 9 - 50*	text
551	[nano_uv_oor_15] Show the field ONLY if: [nano_uv_which_test(16)]	Is this value out of range?	yesno 1 Yes 0 No
552	[nano_uv_oor_deviation_15] Show the field ONLY if: [nano_uv_oor_15] = '1'	Is it a deviation listed here or a clinically significant deviation from normal ranges?	yesno 1 Yes 0 No
553	[nano_uv_alt] Show the field ONLY if: [nano_uv_which_test(17)]	ALT *UI/l - REF RANGE: 9 - 50*	text
554	[nano_uv_oor_16] Show the field ONLY if: [nano_uv_which_test(17)]	Is this value out of range?	yesno 1 Yes 0 No
555	[nano_uv_oor_deviation_16] Show the field ONLY if: [nano_uv_oor_16] = '1'	Is it a deviation listed here or a clinically significant deviation from normal ranges?	yesno 1 Yes 0 No
556	[nano_uv_alkaline] Show the field ONLY if: [nano_uv_which_test(18)]	Alkaline phosphatase *UI/l - REF RANGE: 36 - 120*	text
557	[nano_uv_oor_17] Show the field ONLY if: [nano_uv_which_test(18)]	Is this value out of range?	yesno 1 Yes 0 No
558	[nano_uv_oor_deviation_17] Show the field ONLY if: [nano_uv_oor_17] = '1'	Is it a deviation listed here or a clinically significant deviation from normal ranges?	yesno 1 Yes 0 No
559	[nano_uv_ggt] Show the field ONLY if:	GGT *UI/l - REF RANGE: 6 - 42*	text

	[nano_uv_which_test(19)]		
560	[nano_uv_oor_18]<br>Show the field ONLY if:<br>[nano_uv_which_test(19)]	Is this value out of range?	yesno<br>1 Yes<br>0 No
561	[nano_uv_oor_deviation_18]<br>Show the field ONLY if:<br>[nano_uv_oor_18] = '1'	Is it a deviation listed here or a clinically significant deviation from normal ranges?	yesno<br>1 Yes<br>0 No
562	[nano_uv_bilirubin]<br>Show the field ONLY if:<br>[nano_uv_which_test(20)]	Total bilirubin<br>*µmol/l - REF RANGE: 0 - 21*	text
563	[nano_uv_oor_19]<br>Show the field ONLY if:<br>[nano_uv_which_test(20)]	Is this value out of range?	yesno<br>1 Yes<br>0 No
564	[nano_uv_oor_deviation_19]<br>Show the field ONLY if:<br>[nano_uv_oor_19] = '1'	Is it a deviation listed here or a clinically significant deviation from normal ranges?	yesno<br>1 Yes<br>0 No
565	[nano_uv_creactiveprotein]<br>Show the field ONLY if:<br>[nano_uv_which_test(21)]	C reactive protein<br>*mg/l - REF RANGE: < 10*	text
566	[nano_uv_oor_20]<br>Show the field ONLY if:<br>[nano_uv_which_test(21)]	Is this value out of range?	yesno<br>1 Yes<br>0 No
567	[nano_uv_oor_deviation_20]<br>Show the field ONLY if:<br>[nano_uv_oor_20] = '1'	Is it a deviation listed here or a clinically significant deviation from normal ranges?	yesno<br>1 Yes<br>0 No
568	[nano_uv_hematuria]<br>Show the field ONLY if:<br>[nano_uv_which_test(23)]	Hematuria	radio<br>a 0<br>b +<br>c ++<br>d +++
569	[nano_uv_proteins]<br>Show the field ONLY if:	Proteins in urine	radio<br>a 0

	[nano_uv_which_test(23)]		b +<br>c ++<br>d +++
570	[nano_uv_glucose]<br>Show the field ONLY if:<br>[nano_uv_which_test(23)]	Glucose in urine	radio<br>a 0<br>b +<br>c ++<br>d +++
571	[nano_uv_pregnancy]<br>Show the field ONLY if:<br>[nano_uv_which_test(24)]	Pregnancy test result	yesno<br>1 Yes<br>0 No
572	[nano_uv_other]<br>Show the field ONLY if:<br>[nano_uv_which_test(22)]	Other, specify	text
573	[nano_uv_radio_exams]<br>Show the field ONLY if:<br>[nano_uv_paraclinical_perf] = "1"	Radiological exams*	yesno<br>1 Yes<br>0 No
574	[nano_uv_other_exams]<br>Show the field ONLY if:<br>[nano_uv_paraclinical_perf] = "1"	Other exam	yesno<br>1 Yes<br>0 No
575	[nano_uv_para_specifiy]<br>Show the field ONLY if:<br>[nano_uv_other_exams] = "1" OR [nano_uv_radio_exams] = "1"	Specify	text
576	[nano_uv_text]<br>Show the field ONLY if:<br>[nano_uv_paraclinical_perf] = "1" or [nano_uv_radio_exams] = "1" or [nano_uv_labo_tests] = "1"	* Do not forget to collect complementary exam, remove identifying data, code them and register them as source documents.	descriptive
577	[nano_uv_eligible]	Section Header: *Continuation of trial - Review of eligibility*<br>Is the volunteer still eligible to continue with the trial ?	yesno<br>1 Yes<br>0 No
578	[nano_uv_text_2]	Complete the END OF TRIAL FORM	descriptive

	Show the field ONLY if: [nano_uv_eligible] = "0"		
579	[nano_uv_date_protocol_deviation] Show the field ONLY if: [nano_uv_eligible] = "1"	Date of next visit	text (date_dmy)
580	[nano_uv_comment]	Section Header: *Additional comment* Any additional comment?	yesno<br>1 Yes<br>0 No
581	[nano_uv_comment_specify] Show the field ONLY if: [nano_uv_comment] = "1"	Specify	notes
582	[unscheduled_visit_complete]	Section Header: *Form Status* Complete?	dropdown<br>0 Incomplete<br>1 Unverified<br>2 Complete
**Instrument: Prescription form (prescription_form)**			
583	[nano_pf_title]	FORMULAIRE DE PRESCRIPTION	descriptive
584	[nano_pf_nano_id]	Screening ID	text<br>Field Annotation: @DEFAULT='[record-name]' @READONLY
585	[nano_pf_nano_id_2]	Inclusion ID	text<br>Field Annotation: @DEFAULT='[screening_arm_1][nano_vi_full_id]' @READONLY
586	[nano_pf_ipp]	IPP	text<br>Field Annotation: @DEFAULT='[screening_arm_1][nano_vi_ipp]' @READONLY
587	[nano_pf_dose]		radio<br>1 Low dose (vera / placebo)<br>2 High dose (vera/placebo)
588	[nano_pf_first_vac]		radio<br>1 1ère vaccination<br>2 2ème vaccination
589	[nano_pf_date]	Section Header: *1ère vaccination* Date d'administration souhaitée pour la première vaccination :	text (date_dmy)
590	[nano_pf_hour]	Heure souhaitée pour la première vaccination :	text (time)
591	[nano_pf_name_date]	Date de la demande pour la première vaccination	text (date_dmy)

592	[nano_pf_name]	Nom du demandeur pour la première vaccination	text
593	[nano_pf_signature]	Signature pour la première vaccination	file (signature)
594	[nano_pf_send]	Envoi de la demande pour la première vaccination par mail à la pharmacie ?	yesno<br>1 Yes<br>0 No
595	[nano_pf_date_2]<br>Show the field ONLY if:<br>[nano_pf_first_vac] = "2"	Section Header: *2è vaccination*<br>Date d'administration souhaitée pour la deuxième vaccination :	text (date_dmy)
596	[nano_pf_hour_2]<br>Show the field ONLY if:<br>[nano_pf_first_vac] = "2"	Heure souhaitée pour la deuxième vaccination :	text (time)
597	[nano_pf_name_date_2]<br>Show the field ONLY if:<br>[nano_pf_first_vac] = "2"	Date de la demande pour la deuxième vaccination	text (date_dmy)
598	[nano_pf_name_2]<br>Show the field ONLY if:<br>[nano_pf_first_vac] = "2"	Nom du demandeur pour la deuxième vaccination	text
599	[nano_pf_signature_2]<br>Show the field ONLY if:<br>[nano_pf_first_vac] = "2"	Signature pour la deuxième vaccination	file (signature)
600	[nano_pf_send_2]<br>Show the field ONLY if:<br>[nano_pf_first_vac] = "2"	Envoi de la demande pour la deuxième vaccination par mail à la pharmacie ?	yesno<br>1 Yes<br>0 No
601	[prescription_form_complete]	Section Header: *Form Status*<br>Complete?	dropdown<br>0 Incomplete<br>1 Unverified<br>2 Complete
**Instrument: Pregnancy form (pregnancy_form)**			
602	[nano_pr_title]	Title of research project	notes<br>Custom alignment: RH<br>Field Annotation: @DEFAULT = 'naNO-DENGUE: A Phase-I study of a nanoparticle-based peptide vaccine against Dengue virus'

603	[nano_pr_sponsor_name]	Name of Sponsor	text<br>Custom alignment: RH<br>Field Annotation: @DEFAULT = 'Emergex Vaccines Holding Limited'
604	[nano_pr_sponsor_adress]	Adress of Sponsor	notes<br>Custom alignment: RH<br>Field Annotation: @DEFAULT = '4 & 5 Dunmore Court Wootton Road, Abingdon, Oxfordshire, England, OX13 6BH'
605	[nano_pr_pi_name]	Name of Principal Investigator	text<br>Custom alignment: RH<br>Field Annotation: @DEFAULT = 'Prof. Blaise Genton'
606	[nano_pr_pi_adress]	Adress of Principal Investigator	notes<br>Custom alignment: RH<br>Field Annotation: @DEFAULT = 'Unisanté, Département Formation, recherche et innovation Policlinique de médecine tropicale, voyages et vaccinations Rue du Bugnon 44 1011 Lausanne, Suisse Blaise.genton@unisante.ch M :+41 79 556 58 68'
607	[nano_pr_product_name]	Investigational Medicinal Product Name	text<br>Field Annotation: @DEFAULT="PepGNP-DENGUE vaccine"
608	[nano_pr_project_number]	BASEC research project number (Swissethics)	text<br>Field Annotation: @DEFAULT="2020-02258"
609	[nano_pr_notif_number]	Notification n° (Swissmedic)	text
610	[nano_pr_yob]	Section Header: *Maternal information*<br>Year of birth	text (integer, Min: 1900, Max: 2100)
611	[nano_pr_height]	Height	text (integer, Min: 100, Max: 220)
612	[nano_pr_weight]	Weight	text (integer, Min: 25, Max: 170)
613	[nano_pr_contraception_met]	Section Header: *Contraception*<br>Method of contraception	text
614	[nano_pr_contra_used]	Contraception used as instructed	yesno<br>1 Yes<br>0 No
615	[nano_pr_risk]	Section Header: *Medical history*<br>Patient past history (include information on familial disorders, known risk factors or conditions that may affect the outcome of the pregnancy e.g. alcohol, smoking, other substance consumption, hypertension, eclampsia, diabetes including gestational, infections during pregnancy, environmental or occupational exposure that may pose a risk factor).	text
616	[nano_pr_prev_pregn]	Previous pregnancies (including abortion or stillbirth)	yesno<br>1 Yes<br>0 No

617	[nano_pr_count_gest]<br>Show the field ONLY if:<br>[nano_pr_prev_pregn] = "1"	How many gestation ?	dropdown<br>1 1<br>2 2<br>3 3
618	[nano_pr_week_gestation]<br>Show the field ONLY if:<br>[nano_pr_prev_pregn] = "1" and [nano_pr_count_gest] > 0	Gestation week	text (number)
619	[nano_pr_outcome]<br>Show the field ONLY if:<br>[nano_pr_prev_pregn] = "1" and [nano_pr_count_gest] > 0	Outcome including any abnormalites	text
620	[nano_pr_week_gestation_2]<br>Show the field ONLY if:<br>[nano_pr_prev_pregn] = "1" and [nano_pr_count_gest] > 1	Gestation week	text (number)
621	[nano_pr_outcome_2]<br>Show the field ONLY if:<br>[nano_pr_prev_pregn] = "1" and [nano_pr_count_gest] > 1	Outcome including any abnormalites	text
622	[nano_pr_week_gestation_3]<br>Show the field ONLY if:<br>[nano_pr_prev_pregn] = "1" and [nano_pr_count_gest] > 2	Gestation week	text (number)
623	[nano_pr_outcome_3]<br>Show the field ONLY if:<br>[nano_pr_prev_pregn] = "1" and [nano_pr_count_gest] > 2	Outcome including any abnormalites	text
624	[nano_pr_concom_drug]	Section Header: *Relevant medication*<br>Any concomittant drug(s)?	yesno<br>1 Yes<br>0 No
625	[nano_pr_count_conc]<br>Show the field ONLY if:<br>[nano_pr_concom_drug] = "1"	How many concomittant drug(s) ?	dropdown<br>1 1<br>2 2<br>3 3

			4 4<br>5 5
626	[nano_pr_med_name]<br>Show the field ONLY if:<br>[nano_pr_concom_drug] = "1" and [nano_pr_count_conc] > 0	Medication Name (Commercial name)	text
627	[nano_pr_med_int_name]<br>Show the field ONLY if:<br>[nano_pr_concom_drug] = "1" and [nano_pr_count_conc] > 0	Medication Name (International nonproprietary name)	text
628	[nano_pr_dose]<br>Show the field ONLY if:<br>[nano_pr_concom_drug] = "1" and [nano_pr_count_conc] > 0	Dose	text (integer, Min: 0, Max: 9999999)
629	[nano_pr_dose_unit]<br>Show the field ONLY if:<br>[nano_pr_concom_drug] = "1" and [nano_pr_count_conc] > 0	Dose unit	radio<br>a Microgram<br>b Miligram<br>c Gram<br>d Mililiter<br>e Other (Specify)<br>f Unknown
630	[nano_pr_med_frequency]<br>Show the field ONLY if:<br>[nano_pr_concom_drug] = "1" and [nano_pr_count_conc] > 0	Frequency	radio<br>a Daily<br>b Twice daily<br>c Three times a day<br>d Four times a day<br>e 4-6 hours<br>f 6-8 hours<br>g PRN (ad needed)<br>h Other (specify)
631	[nano_pr_med_route]<br>Show the field ONLY if:<br>[nano_pr_concom_drug] = "1" and [nano_pr_count_conc] > 0	Route	radio<br>a Oral<br>b Topical<br>c Subcutaneous injection<br>d Intraveinous injection<br>e Intramuscular injection<br>f Rectal<br>g Nasal<br>h Inhaled

			i Transdermal<br>j Vaginal<br>k Other (Specify)
632	[nano_pr_med_indication]<br>Show the field ONLY if:<br>[nano_pr_concom_drug] = "1" and [nano_pr_count_conc] > 0	Indication(s) for use	text
633	[nano_pr_med_start_date]<br>Show the field ONLY if:<br>[nano_pr_concom_drug] = "1" and [nano_pr_count_conc] > 0	Start Date	text (date_dmy)
634	[nano_pr_med_ongoing]<br>Show the field ONLY if:<br>[nano_pr_concom_drug] = "1" and [nano_pr_count_conc] > 0	Ongoing	yesno<br>1 Yes<br>0 No
635	[nano_pr_med_end_date]<br>Show the field ONLY if:<br>[nano_pr_med_ongoing] = '0' and [nano_pr_concom_drug] = "1" and [nano_pr_count_conc] > 0	End date	text (date_dmy)
636	[nano_pr_med_name_2]<br>Show the field ONLY if:<br>[nano_pr_concom_drug] = "1" and [nano_pr_count_conc] > 1	Medication Name (Commercial name)	text
637	[nano_pr_med_int_name_2]<br>Show the field ONLY if:<br>[nano_pr_concom_drug] = "1" and [nano_pr_count_conc] > 1	Medication Name (International nonproprietary name)	text
638	[nano_pr_dose_2]<br>Show the field ONLY if:<br>[nano_pr_concom_drug] = "1" and [nano_pr_count_conc] > 1	Dose	text (integer, Min: 0, Max: 9999999)

639	[nano_pr_dose_unit_2]<br>Show the field ONLY if:<br>[nano_pr_concom_drug] = "1" and [nano_pr_count_conc] > 1	Dose unit	radio<br>a Microgram<br>b Miligram<br>c Gram<br>d Mililiter<br>e Other (Specify)<br>f Unknown
640	[nano_pr_med_frequency_2]<br>Show the field ONLY if:<br>[nano_pr_concom_drug] = "1" and [nano_pr_count_conc] > 1	Frequency	radio<br>a Daily<br>b Twice daily<br>c Three times a day<br>d Four times a day<br>e 4-6 hours<br>f 6-8 hours<br>g PRN (ad needed)<br>h Other (specify)
641	[nano_pr_med_route_2]<br>Show the field ONLY if:<br>[nano_pr_concom_drug] = "1" and [nano_pr_count_conc] > 1	Route	radio<br>a Oral<br>b Topical<br>c Subcutaneous injection<br>d Intraveinous injection<br>e Intramuscular injection<br>f Rectal<br>g Nasal<br>h Inhaled<br>i Transdermal<br>j Vaginal<br>k Other (Specify)
642	[nano_pr_med_indication_2]<br>Show the field ONLY if:<br>[nano_pr_concom_drug] = "1" and [nano_pr_count_conc] > 1	Indication(s) for use	text
643	[nano_pr_med_start_date_2]<br>Show the field ONLY if:<br>[nano_pr_concom_drug] = "1" and [nano_pr_count_conc] > 1	Start Date	text (date_dmy)
644	[nano_pr_med_ongoing_2]<br>Show the field ONLY if:	Ongoing	yesno<br>1 Yes<br>0 No

	[nano_pr_concom_drug] = "1" and [nano_pr_count_conc] > 1		
645	[nano_pr_med_end_date_2]<br>Show the field ONLY if:<br>[nano_pr_med_ongoing] = '0' and [nano_pr_concom_drug] = "1" and [nano_pr_count_conc] > 1	End date	text (date_dmy)
646	[nano_pr_med_name_3]<br>Show the field ONLY if:<br>[nano_pr_concom_drug] = "1" and [nano_pr_count_conc] > 2	Medication Name (Commercial name)	text
647	[nano_pr_med_int_name_3]<br>Show the field ONLY if:<br>[nano_pr_concom_drug] = "1" and [nano_pr_count_conc] > 2	Medication Name (International nonproprietary name)	text
648	[nano_pr_dose_3]<br>Show the field ONLY if:<br>[nano_pr_concom_drug] = "1" and [nano_pr_count_conc] > 2	Dose	text (integer, Min: 0, Max: 9999999)
649	[nano_pr_dose_unit_3]<br>Show the field ONLY if:<br>[nano_pr_concom_drug] = "1" and [nano_pr_count_conc] > 2	Dose unit	radio<br>a Microgram<br>b Miligram<br>c Gram<br>d Mililiter<br>e Other (Specify)<br>f Unknown
650	[nano_pr_med_frequency_3]<br>Show the field ONLY if:<br>[nano_pr_concom_drug] = "1" and [nano_pr_count_conc] > 2	Frequency	radio<br>a Daily<br>b Twice daily<br>c Three times a day<br>d Four times a day<br>e 4-6 hours<br>f 6-8 hours<br>g PRN (ad needed)<br>h Other (specify)
651	[nano_pr_med_route_3]	Route	radio<br>a Oral

	Show the field ONLY if: [nano_pr_concom_drug] = "1" and [nano_pr_count_conc] > 2		b Topical<br>c Subcutaneous injection<br>d Intraveinous injection<br>e Intramuscular injection<br>f Rectal<br>g Nasal<br>h Inhaled<br>i Transdermal<br>j Vaginal<br>k Other (Specify)
652	[nano_pr_med_indication_3]<br>Show the field ONLY if: [nano_pr_concom_drug] = "1" and [nano_pr_count_conc] > 2	Indication(s) for use	text
653	[nano_pr_med_start_date_3]<br>Show the field ONLY if: [nano_pr_concom_drug] = "1" and [nano_pr_count_conc] > 2	Start Date	text (date_dmy)
654	[nano_pr_med_ongoing_3]<br>Show the field ONLY if: [nano_pr_concom_drug] = "1" and [nano_pr_count_conc] > 2	Ongoing	yesno<br>1 Yes<br>0 No
655	[nano_pr_med_end_date_3]<br>Show the field ONLY if: [nano_pr_med_ongoing] = '0' and [nano_pr_concom_drug] = "1" and [nano_pr_count_conc] > 2	End date	text (date_dmy)
656	[nano_pr_med_name_4]<br>Show the field ONLY if: [nano_pr_concom_drug] = "1" and [nano_pr_count_conc] > 3	Medication Name (Commercial name)	text
657	[nano_pr_med_int_name_4]<br>Show the field ONLY if:	Medication Name (International nonproprietary name)	text

	[nano_pr_concom_drug] = "1" and [nano_pr_count_conc] > 3		
658	[nano_pr_dose_4]<br>Show the field ONLY if:<br>[nano_pr_concom_drug] = "1" and [nano_pr_count_conc] > 3	Dose	text (integer, Min: 0, Max: 9999999)
659	[nano_pr_dose_unit_4]<br>Show the field ONLY if:<br>[nano_pr_concom_drug] = "1" and [nano_pr_count_conc] > 3	Dose unit	radio<br>a Microgram<br>b Miligram<br>c Gram<br>d Mililiter<br>e Other (Specify)<br>f Unknown
660	[nano_pr_med_frequency_4]<br>Show the field ONLY if:<br>[nano_pr_concom_drug] = "1" and [nano_pr_count_conc] > 3	Frequency	radio<br>a Daily<br>b Twice daily<br>c Three times a day<br>d Four times a day<br>e 4-6 hours<br>f 6-8 hours<br>g PRN (ad needed)<br>h Other (specify)
661	[nano_pr_med_route_4]<br>Show the field ONLY if:<br>[nano_pr_concom_drug] = "1" and [nano_pr_count_conc] > 3	Route	radio<br>a Oral<br>b Topical<br>c Subcutaneous injection<br>d Intraveinous injection<br>e Intramuscular injection<br>f Rectal<br>g Nasal<br>h Inhaled<br>i Transdermal<br>j Vaginal<br>k Other (Specify)
662	[nano_pr_med_indication_4]<br>Show the field ONLY if:<br>[nano_pr_concom_drug] = "1" and [nano_pr_count_conc] > 3	Indication(s) for use	text
663	[nano_pr_med_start_date_4]	Start Date	text (date_dmy)

	Show the field ONLY if: [nano_pr_concom_drug] = "1" and [nano_pr_count_conc] > 3		
664	[nano_pr_med_ongoing_4] Show the field ONLY if: [nano_pr_concom_drug] = "1" and [nano_pr_count_conc] > 3	Ongoing	yesno<br>1 Yes<br>0 No
665	[nano_pr_med_end_date_4] Show the field ONLY if: [nano_pr_med_ongoing] = '0' and [nano_pr_concom_drug] = "1" and [nano_pr_count_conc] > 3	End date	text (date_dmy)
666	[nano_pr_med_name_5] Show the field ONLY if: [nano_pr_concom_drug] = "1" and [nano_pr_count_conc] > 4	Medication Name (Commercial name)	text
667	[nano_pr_med_int_name_5] Show the field ONLY if: [nano_pr_concom_drug] = "1" and [nano_pr_count_conc] > 4	Medication Name (International nonproprietary name)	text
668	[nano_pr_dose_5] Show the field ONLY if: [nano_pr_concom_drug] = "1" and [nano_pr_count_conc] > 4	Dose	text (integer, Min: 0, Max: 9999999)
669	[nano_pr_dose_unit_5] Show the field ONLY if: [nano_pr_concom_drug] = "1" and [nano_pr_count_conc] > 4	Dose unit	radio<br>a Microgram<br>b Miligram<br>c Gram<br>d Mililiter<br>e Other (Specify)<br>f Unknown
670	[nano_pr_med_frequency_5] Show the field ONLY if: [nano_pr_concom_drug] = "1" and [nano_pr_	Frequency	radio<br>a Daily<br>b Twice daily<br>c Three times a day

	count_conc] > 4		d Four times a day<br>e 4-6 hours<br>f 6-8 hours<br>g PRN (ad needed)<br>h Other (specify)
671	[nano_pr_med_route_5]<br>Show the field ONLY if:<br>[nano_pr_concom_drug] = "1" and [nano_pr_count_conc] > 4	Route	radio<br>a Oral<br>b Topical<br>c Subcutaneous injection<br>d Intraveinous injection<br>e Intramuscular injection<br>f Rectal<br>g Nasal<br>h Inhaled<br>i Transdermal<br>j Vaginal<br>k Other (Specify)
672	[nano_pr_med_indication_5]<br>Show the field ONLY if:<br>[nano_pr_concom_drug] = "1" and [nano_pr_count_conc] > 4	Indication(s) for use	text
673	[nano_pr_med_start_date_5]<br>Show the field ONLY if:<br>[nano_pr_concom_drug] = "1" and [nano_pr_count_conc] > 4	Start Date	text (date_dmy)
674	[nano_pr_med_ongoing_5]<br>Show the field ONLY if:<br>[nano_pr_concom_drug] = "1" and [nano_pr_count_conc] > 4	Ongoing	yesno<br>1 Yes<br>0 No
675	[nano_pr_med_end_date_5]<br>Show the field ONLY if:<br>[nano_pr_med_ongoing] = '0' and [nano_pr_concom_drug] = "1" and [nano_pr_count_conc] > 4	End date	text (date_dmy)
676	[nano_pr_tests_perf]	Section Header: *Pregnancy informations*	yesno<br>1 Yes

		Have any specific tests, e.g. amniocentesis, ultrasound, maternal serum AFP, been performed during the pregnancy so far?	0 No
677	[nano_pr_specify] Show the field ONLY if: [nano_pr_tests_perf] = "1"	Specify	text
678	[nano_pr_delivery]	Delivery	yesno<br>1 Yes<br>0 No
679	[nano_pr_delivery_spec] Show the field ONLY if: [nano_pr_delivery] = "1"	Specify	radio<br>1 Normal<br>2 Forceps/Ventouse<br>3 Caesarean section
680	[nano_pr_problems] Show the field ONLY if: [nano_pr_delivery] = "1"	Maternal complicatons or problems related to birth	yesno<br>1 Yes<br>0 No
681	[nano_pr_problems_specify] Show the field ONLY if: [nano_pr_problems] = "1"	Specify	text
682	[nano_pr_abortion]	Abortion	yesno<br>1 Yes<br>0 No
683	[nano_pr_abortion_date] Show the field ONLY if: [nano_pr_delivery] = "1" or [nano_pr_abortion] = "1"	Date	text (date_dmy)
684	[nano_pr_abortion_week] Show the field ONLY if: [nano_pr_delivery] = "1" or [nano_pr_abortion] = "1"	At week	text (integer)
685	[nano_pr_abortion_specify] Show the field ONLY if: [nano_pr_abortion] = "1"	Specify	radio<br>1 Therapeutic<br>2 Planned<br>3 Spontaneous

686	[nano_pr_specify_abnorm]<br>Show the field ONLY if:<br>[nano_pr_abortion] = "1"	Specify reason and any abnormalities (if known)	text (integer)
687	[nano_pr_neonate]	Section Header: *Child informations*<br>Neonate	radio<br>a Normal<br>b Abnormal<br>c Stillbirth
688	[nano_pr_neonate_specify]<br>Show the field ONLY if:<br>[nano_pr_neonate] = "b" or [nano_pr_neonate] = "c"	Specify	text
689	[nano_pr_child_gender]	Gender	radio<br>1 Male<br>2 Female
690	[nano_pr_child_height]	Height	text (integer, Min: 0, Max: 70)
691	[nano_pr_child_weight]	Weight	text (integer, Min: 0, Max: 7)
692	[nano_pr_child_head]	Head circumference	text (integer, Min: 0, Max: 70)
693	[nano_pr_child_apgar_1]	Apgar score 1 min	text (integer, Min: 0, Max: 10)
694	[nano_pr_child_apgar_5]	Apgar score 5 min	text (integer, Min: 0, Max: 10)
695	[nano_pr_child_apgar_10]	Apgar score 10 min	text (integer, Min: 0, Max: 10)
696	[nano_pr_seriousness]	Section Header: *Assessment of pregnancy outcome*<br>Seriousness criteria	radio<br>1 Non serious<br>2 Mother died<br>3 Stillbirth/ Neonate died<br>4 Hospitalisation<br>5 Persistent or significant disability/incapacity<br>6 Congenital anormality/birth defect<br>7 Life-threatening<br>8 Other significant medical events

697	[nano_pr_causality]	Assessment of causality with study IMP	radio<br>1 No relationship *<br>2 Unlikely *<br>3 Possible *<br>4 Probable *<br>5 Definite *
698	[nano_pr_comments]	Section Header: *Additional informations*<br>Comments	text
699	[nano_pr_investigator_name]	Section Header: *Investigator signature*<br>Name of investigator	text
700	[nano_pr_approved_pi]	Section Header: *Pregnancy report PI evaluation*<br>Pregnancy report approved by PI	yesno<br>1 Yes<br>0 No
701	[nano_pr_investigator_date]	Place, date and signature of investigator	text
702	[nano_pr_add_comment]	Additionnal comment	yesno<br>1 Yes<br>0 No
703	[nano_pr_text]	All pregnancies must be reported to the Principal Investigator	descriptive
704	[nano_pr_add_comments]<br>Show the field ONLY if:<br>[nano_pr_add_comment] = "1"	Specify	text
705	[nano_pr_pi_validation]	Send the report to the PI for modification/validation	yesno<br>1 Yes<br>0 No
706	[nano_pr_pi_name_2]	Name of PI	text<br>Field Annotation: @DEFAULT="Prof Blaise Genton"
707	[nano_pr_pi_date]	Place, date and signature of PI	text<br>Field Annotation: @DEFAULT="Prof Blaise Genton"
708	[nano_pr_text_2]	All pregnancies must be reported immediately and within a maximum of 24 hours to the Sponsor	descriptive
709	[nano_pr_lock_report]	Lock the report	yesno<br>1 Yes<br>0 No
710	[nano_pr_send_report_sponsor]	Send the PDF report to the Sponsor	yesno<br>1 Yes<br>0 No
711	[pregnancy_form_complete]	Section Header: *Form Status*<br>Complete?	dropdown<br>0 Incomplete

			1 Unverified<br>2 Complete
**Instrument: End Of Trial / Early Termination (end_of_trial_early_termination)**			
712	[nano_end_date_completion]	Date of end of trial	text (date_dmy)
713	[nano_end_trial_period]	Did the volunteer complete the 6 months trial period ?	yesno<br>1 Yes<br>0 No
714	[nano_end_date_last]<br>Show the field ONLY if:<br>[nano_end_trial_period]='0'	Date of last contact	text (date_dmy)
715	[nano_end_reason]<br>Show the field ONLY if:<br>[nano_end_trial_period]='0'	Specify reason for early termination	radio<br>a Not eligible<br>b Consent withdrawal<br>c Lost to follow up<br>d Death<br>e Adverse Event<br>f Violation of protocol<br>g Other
716	[nano_end_specify]<br>Show the field ONLY if:<br>[nano_end_reason]='b' or [nano_end_reason]='f' or [nano_end_reason]='g'	Specify	text
717	[nano_end_fill_ae_form]<br>Show the field ONLY if:<br>[nano_end_reason]='d' or [nano_end_reason] = 'e'	Please fill a SAE form - Please fill a AE form	descriptive
718	[nano_end_rando_broken]	Has the randomization code been broken ?	yesno<br>1 Yes<br>0 No
719	[nano_end_date_broken]<br>Show the field ONLY if:<br>[nano_end_rando_broken]='1'	Date	text (date_dmy)
720	[nano_end_allocation]<br>Show the field ONLY if:	Allocation	radio<br>1 Vaccine vera<br>2 Vehicle control

	[nano_end_rando_broken]='1'		
721	[nano_end_additional_comm]	Any additional comment?	yesno<br>1 Yes<br>0 No
722	[nano_end_add_comm_spec]<br>Show the field ONLY if:<br>[nano_end_additional_comm]='1'	Specify	text
723	[end_of_trial_early_termination_complete]	Section Header: *Form Status*<br>Complete?	dropdown<br>0 Incomplete<br>1 Unverified<br>2 Complete
**Instrument: Medication Form (medication_form)**			
724	[nano_mf_med_name]	Medication Name (Commercial name)	text
725	[nano_mf_med_int_name]	Medication Name (International nonproprietary name)	text
726	[nano_mf_dose]	Dose	text (number, Min: 0, Max: 9999999)
727	[nano_mf_dose_unit]	Dose unit	radio<br>a Microgram<br>b Miligram<br>c Gram<br>d Mililiter<br>e Other (Specify)<br>f Unknown
728	[nano_mf_specify]<br>Show the field ONLY if:<br>[nano_mf_dose_unit] = "e"	Specify	text
729	[nano_mf_med_frequency]	Frequency	radio<br>a Daily<br>b Twice daily<br>c Three times a day<br>d Four times a day<br>e 4-6 hours<br>f 6-8 hours<br>g PRN (as needed)<br>h Other (specify)

730	[nano_mf_specify_2]<br>Show the field ONLY if:<br>[nano_mf_med_frequency] = "h"	Specify	text
731	[nano_mf_med_route]	Route	radio<br>a Oral<br>b Topical<br>c Subcutaneous injection<br>d Intraveinous injection<br>e Intramuscular injection<br>f Rectal<br>g Nasal<br>h Inhaled<br>i Transdermal<br>j Vaginal<br>k Other (Specify)
732	[nano_mf_specify_3]<br>Show the field ONLY if:<br>[nano_mf_med_route] = "k"	Specify	text
733	[nano_mf_med_indication]	Indication(s) for use	text
734	[nano_mf_med_start_date]	Start Date	text (date_dmy)
735	[nano_mf_med_ongoing]	Ongoing	yesno<br>1 Yes<br>0 No
736	[nano_mf_med_end_date]<br>Show the field ONLY if:<br>[nano_mf_med_ongoing] = '0'	End date	text (date_dmy)
737	[nano_mf_med_prescribed]	Was this medication prescribed for an AE ?	yesno<br>1 Yes<br>0 No
738	[nano_mf_med_ae_n]<br>Show the field ONLY if:<br>[nano_mf_med_prescribed] = '1'	AE number	sql<br>SELECT instance, value from redcap_data where project_id = 341 and field_name = 'nano_ae_number' and record = [record-name] order by value
739	[medication_form_complete]	Section Header: *Form Status*<br>Complete?	dropdown<br>0 Incomplete<br>1 Unverified

			2 Complete

Instrument: Screening and enrolment log (screening_and_enrolment_log)

740	[nano_sel_sr_id]	Screening ID	text<br>Field Annotation: @READONLY
741	[nano_sel_id]	Inclusion ID	text<br>Field Annotation: @READONLY
742	[nano_sel_date_icf]	Date of ICF signature	text (datetime_dmy)<br>Field Annotation: @READONLY
743	[nano_sel_date_screening]	Date of screening	text (date_dmy)<br>Field Annotation: @READONLY
744	[nano_sel_date_sf]	Date of screenfailure (if applicable)	text (date_dmy)<br>Field Annotation: @READONLY
745	[nano_sel_sf_specify]	Specify reason for screen failure	radio<br>a Age < 18 or > 45 years<br>b Written consent not provided/consent withdrawal<br>c Not able/do not want to attend all scheduled visit/to comply with all study requirments<br>d Chronic illness that might interfere with trial conduct<br>e Current alcohol abuse or drug addiction (reported or suspected)<br>f Known or suspected congenital or acquired immunodeficiency; or receipt of immunosuppressive therapy<br>g Known systemic hypersensitivity to any of the vaccine components (e.g. gold), or history of a life-threatening heaction to vaccines, or to a vaccine containing any of the same substances<br>h Thrombocytopenia or any coagulation disorder<br>i Pregnancy or lactating<br>j Non-use of an effective method of contraception from at least 4 weeks prior to the first vaccination<br>k Participation in another clinical trial investigating a vaccine, drug, medical device or medical procedure in the 4 weeks preceding the first vaccination, or planned participation during the study period<br>l Receipt of any vaccine in the 4 weeks preceding the trial vaccination (excepting influenza vaccination, which may be received up to 2 weeks before study vaccines) or planned receipt of any

			vaccine in the 4 weeks following trial vaccination<br>m — Receipt of immunoglobulins, blood or blood-derived products in the past 3 months<br>n — Previous vaccination against Japanese encephalitis, Yellow Fever, or any dengue virus vaccine<br>o — Self-reported or documented history of flavivirus infection*, confirmed either clinically or serologically<br>p — Self-reported or documented seropositivity for human immunodeficiency virus (HIV), hepatitis B natural infection (HBcAb positive serology), or hepatitis C<br>q — Previous residence for more than 12months in, or travel in the last 30 days to flavivirus-endemic regions<br>r — At high risk for dengue infection during the trial<br>s — Identified as an Investigator or employee of the Investigator or study centre with direct involvement in the proposed study, or identified as an immediate family member (i.e., parent, spouse, natural or adopted child) of the Investigator or employee with direct involvement in the proposed study<br>t — Refusal to be informed in the event that relevant results concerning the participant's health are revealed.<br>u — Abnormal laboratory (blood/urine) value<br>v — Lost to follow up<br>w — Other<br><br>Field Annotation: @READONLY
746	[nano_sel_vac]	Vaccinated (1st dose)?	yesno<br>1 — Yes<br>0 — No<br><br>Field Annotation: @READONLY
747	[nano_sel_reason_no_vac]	If no, reason for non-eligiblity for dose 1	text<br>Field Annotation: @READONLY
748	[nano_sel_date_v1]	Date of vaccination 1	text (datetime_dmy)<br>Field Annotation: @READONLY
749	[nano_sel_vac_2]	Vaccinated (2nd dose)?	yesno<br>1 — Yes<br>0 — No

			Field Annotation: @READONLY
750	[nano_sel_reason_no_vac_2]	If no, reason for non-eligiblity for dose 2	text<br>Field Annotation: @READONLY
751	[nano_sel_date_v2]	Date of vaccination 2	text (datetime_dmy)<br>Field Annotation: @READONLY
752	[nano_sel_date_end]	Date of end of trial :	text (date_dmy)<br>Field Annotation: @READONLY
753	[nano_sel_date_end_6m]	Did the volunteer complete the 6 months trial period ?	yesno<br>1 Yes<br>0 No<br>Field Annotation: @READONLY
754	[nano_sel_date_end_2]	If early termination, reason for end of trial :	radio<br>a Not eligible<br>b Consent withdrawal<br>c Lost to follow up<br>d Death<br>e Adverse Event<br>f Violation of protocol<br>g Other<br>Field Annotation: @READONLY
755	[screening_and_enrolment_log_complete]	Section Header: *Form Status*<br>Complete?	dropdown<br>0 Incomplete<br>1 Unverified<br>2 Complete
**Instrument: Compensation log (compensation_log)**			
756	[nano_comp_id]	Inclusion ID	text<br>Field Annotation: @READONLY
757	[nano_comp]	1st vaccination<br>Compensation received ?	yesno<br>1 Yes<br>0 No<br>Field Annotation: @READONLY
758	[nano_comp_chf_date]	Date	text (date_dmy)<br>Field Annotation: @READONLY
759	[nano_comp_reason]	If no, reason	text<br>Field Annotation: @READONLY
760	[nano_comp_2]	Visit 4<br>Compensation received ?	yesno<br>1 Yes<br>0 No<br>Field Annotation: @READONLY
761	[nano_comp_chf_date_2]	Date	text (date_dmy)<br>Field Annotation: @READONLY

762	[nano_comp_reason_2]	If no, reason	text<br>Field Annotation: @READONLY
763	[nano_comp_3]	Visit 5<br>Compensation received ?	yesno<br>1 Yes<br>0 No<br>Field Annotation: @READONLY
764	[nano_comp_chf_date_3]	Date	text (date_dmy)<br>Field Annotation: @READONLY
765	[nano_comp_reason_3]	If no, reason	text<br>Field Annotation: @READONLY
766	[nano_comp_4]	2nd vaccination<br>Compensation received ?	yesno<br>1 Yes<br>0 No<br>Field Annotation: @READONLY
767	[nano_comp_chf_date_4]	Date	text (date_dmy)<br>Field Annotation: @READONLY
768	[nano_comp_reason_4]	If no, reason	text<br>Field Annotation: @READONLY
769	[nano_comp_5]	Visit 8<br>Compensation received ?	yesno<br>1 Yes<br>0 No<br>Field Annotation: @READONLY
770	[nano_comp_chf_date_5]	Date	text (date_dmy)<br>Field Annotation: @READONLY
771	[nano_comp_reason_5]	If no, reason	text<br>Field Annotation: @READONLY
772	[nano_comp_6]	Visit 9<br>Compensation received ?	yesno<br>1 Yes<br>0 No<br>Field Annotation: @READONLY
773	[nano_comp_chf_date_6]	Date	text (date_dmy)<br>Field Annotation: @READONLY
774	[nano_comp_reason_6]	If no, reason	text<br>Field Annotation: @READONLY
775	[nano_comp_7]	Visit 11<br>Compensation received ?	yesno<br>1 Yes<br>0 No<br>Field Annotation: @READONLY
776	[nano_comp_chf_date_7]	Date	text (date_dmy)<br>Field Annotation: @READONLY
777	[nano_comp_reason_7]	If no, reason	text<br>Field Annotation: @READONLY
778	[nano_comp_8]	Visit 12<br>Compensation received ?	yesno<br>1 Yes

			0 No<br><br>Field Annotation: @READONLY
779	[nano_comp_chf_date_8]	Date	text (date_dmy)<br>Field Annotation: @READONLY
780	[nano_comp_reason_8]	If no, reason	text<br>Field Annotation: @READONLY
781	[compensation_log_complete]	Section Header: *Form Status*<br>Complete?	dropdown<br>0 Incomplete<br>1 Unverified<br>2 Complete

Data Dictionary Codebook

naNO-COVID - Pharmacovigilance (PID: 342)

01/10/2024 9:10am

#	Variable / Field Name	Field Label<br>*Field Note*	Field Attributes (Field Type, Validation, Choices, Calculations, etc.)
Instrument: **Serious Adverse Event Form** (serious_adverse_event_form)			
1	[record_id]	Record id	text, Required<br>Field Annotation: @HIDDEN-PDF
2	[nano_sae_sae_number]	SAE number	text, Required
3	[nano_sae_custom_record_id]	Custom record id	text, Required<br>Field Annotation: @HIDDEN @HIDDEN-PDF
4	[nano_sae_title]	Title of research project	notes, Required<br>Custom alignment: RH<br>Field Annotation: @DEFAULT = 'naNO-DENGUE: A Phase-I study of a nanoparticle-based peptide vaccine against Dengue virus'
5	[nano_sae_sponsor_name]	Name of Sponsor	text, Required<br>Custom alignment: RH<br>Field Annotation: @DEFAULT = 'Emergex Vaccines Holding Limited'
6	[nano_sae_sponsor_adress]	Address of Sponsor	notes, Required<br>Custom alignment: RH<br>Field Annotation: @DEFAULT = '4 & 5 Dunmore Court Wootton Road, Abingdon, Oxfordshire, England, OX13 6BH'
7	[nano_sae_pi_name]	Name of Principal Investigator	text, Required<br>Custom alignment: RH<br>Field Annotation: @DEFAULT = 'Prof. Blaise Genton'
8	[nano_sae_product_name]	Investigational Medicinal Product Name	text, Required<br>Field Annotation: @DEFAULT="PepGNP-DENGUE vaccine"
9	[nano_sae_pi_adress]	Adress of Principal Investigator	notes, Required<br>Custom alignment: RH<br>Field Annotation: @DEFAULT = 'Unisanté, Département Formation, recherche et innovation Policlinique de médecine tropicale, voyages et vaccinations Rue du Bugnon 44 1011 Lausanne, Suisse Blaise.genton@unisante.ch M :+41 79 556 58 68'
10	[nano_sae_project_number]	BASEC research project number (Swissethics)	text, Required<br>Field Annotation: @DEFAULT="2020-02258"
11	[nano_sae_notif_number]	Notification n° (Swissmedic)	text, Required<br>Field Annotation: @DEFAULT="2021DR1042"
12	[nano_sae_id]	Section Header: *SAE information*<br>Inclusion ID	text, Required<br>Custom alignment: RH
13	[nano_sae_dob]	Year of birth	text (integer), Required<br>Custom alignment: RH
14	[nano_sae_sex]	Sex	radio, Required<br>1 Male<br>2 Female

			Custom alignment: RH
15	[nano_sae_ethnicity]	Ethnicity (Several possible)	checkbox, Required<br>1 nano_sae_ethnicity___1 Caucasian<br>2 nano_sae_ethnicity___2 Asian<br>3 nano_sae_ethnicity___3 Black<br>4 nano_sae_ethnicity___4 Carribean<br>5 nano_sae_ethnicity___5 Indian<br>6 nano_sae_ethnicity___6 Arab<br>7 nano_sae_ethnicity___7 Other
16	[nano_sae_ethnicity_specify]<br>Show the field ONLY if: [nano_sae_ethnicity(7)] = '1'	Specify	text, Required
17	[nano_sae_date_container]	Date of onset {nano_sae_ae_date} {nano_sae_date_unknown}	descriptive
18	[nano_sae_ae_date]	AE onset date	text (date_dmy)
19	[nano_sae_date_unknown]	Date unknown	checkbox<br>1 nano_sae_date_unknown___1 Date unknown
20	[nano_sae_ae_awareness]	AE awareness date	text (date_dmy), Required
21	[nano_sae_ae_evolution]	Does this SAE form report the evolution of a previously reported AE ?	yesno, Required<br>1 Yes<br>0 No
22	[nano_sae_ae_number]<br>Show the field ONLY if: [nano_sae_ae_evolution] = "1"	Specify AE number	text, Required
23	[nano_sae_ae_description]	Section Header: *General*<br>Describe the AE and the connection to project procedures (including relevant test/lab data)	notes, Required
24	[nano_sae_ae_solicited]	Which type of AE is it ?	radio, Required<br>1 Solicited AE<br>2 Unsolicited AE related with an abnormal laboratory value<br>3 Unsolicited AE
25	[nano_sae_fda_toxicity]<br>Show the field ONLY if: [nano_sae_ae_solicited] = "1"	FDA toxicity grading scale term	dropdown, Required<br>1 Pain<br>2 Tenderness<br>3 Erythema/Redness<br>4 Induration/Swelling<br>5 Nausae/Vomiiting<br>6 Diarrhea<br>7 Headache<br>8 Fatigue

			9 Myalgia<br>10 Fever
26	[nano_sae_fda_tox_grade_2]<br>Show the field ONLY if:<br>[nano_sae_ae_solicited] = "1"	AE grade according to adapted FDA toxicity grading scale	radio, Required<br>1 Grade 1<br>2 Grade 2<br>3 Grade 3
27	[nano_sae_fda_tox_grade]<br>Show the field ONLY if:<br>[nano_sae_ae_solicited] = "1"		descriptive<br>(Attachment: naNO-COVID_FDA_adapted.pdf, Display format: Link)<br>Field Annotation: @HIDDEN-PDF
28	[nano_sae_labo_ctcae_term]<br>Show the field ONLY if:<br>[nano_sae_ae_solicited] = "2"	AE term	text, Required
29	[nano_sae_ctcae_grade_v6]<br>Show the field ONLY if:<br>[nano_sae_ae_solicited] = "2"	AE grade according to adapted CTCAE v5.0	radio, Required<br>1 Grade 1<br>2 Grade 2<br>3 Grade 3<br>4 Grade 4<br>5 Grade 5
30	[nano_sae_ctcae_grade_v7]<br>Show the field ONLY if:<br>[nano_sae_ae_solicited] = "2"		descriptive<br>(Attachment: naNO-COVID_CTCAE_adapted_lab.pdf, Display format: Link)<br>Field Annotation: @HIDDEN-PDF
31	[nano_sae_ctcae_grade]<br>Show the field ONLY if:<br>[nano_sae_ae_solicited] = "3"	CTCAE term for grading AE	text, Required<br>BIOPORTAL:CTCAE BIOPORTAL:CTCAE
32	[nano_sae_spec_ctcae]<br>Show the field ONLY if:<br>[nano_sae_ae_solicited] = "3"	If needed, please specify	text, Required
33	[nano_sae_ctcae_grade_v5]<br>Show the field ONLY if:<br>[nano_sae_ae_solicited] = "3"	AE grade according to CTCAE v5.0	radio, Required<br>1 Grade 1<br>2 Grade 2<br>3 Grade 3<br>4 Grade 4<br>5 Grade 5
34	[nano_sae_yes_no]	Is this a serious adverse event ?	yesno, Required<br>1 Yes<br>0 No
35	[nano_sae_type]<br>Show the field ONLY if:	Check SAE (seriousness definition)	radio, Required<br>a Death

	[nano_sae_yes_no] = "1"		b Life-threatening event<br>c Important medical event (i.e. jeopardises the patient or may require an intervention to prevent death or life threatening event)<br>d Persistent or significant disability or incapacity<br>e Hospitalisation or prolongation of an existing hospitalisation<br>f Congenital anomaly or birth defect (in offspring of the subject)
36	[nano_sae_interest]	Is this an adverse event of special interest ?	yesno, Required<br>1 Yes<br>0 No
37	[nano_sae_aesi_def]		descriptive<br>(Attachment: Definition of Adverse Event of Special Interest.pdf, Display format: Link)<br>Field Annotation: @HIDDEN-PDF
38	[nano_sae_unexpected_ae]	Was this an unexpected AE ?	yesno, Required<br>1 Yes<br>0 No
39	[nano_sae_doses]	Section Header: *IMP*<br>Were one or two doses administred ?	radio, Required<br>1 One<br>2 Two
40	[nano_sae_random_number]	Randomisation number	text (integer), Required
41	[nano_sae_code_broken]	Was the code broken ?	yesno, Required<br>1 Yes<br>0 No
42	[nano_sae_detail]<br>Show the field ONLY if:<br>[nano_sae_code_broken] = "1"	Please detail the reason for unblinding, the person who proceeded to unblinding and the date.	notes, Required
43	[nano_sae_imp_name]	Name of IMP : PepGNP-Covid19 or vehicle-GNP	descriptive
44	[nano_sae_dose]	Dose	radio, Required<br>1 Low dose (12.8 ug of gold nanoparticules +/- 2.5 nmol SARSCoV2 peptides)<br>2 High dose (38.3 ug of gold nanoparticules +/- 7.5 nmol SARSCoV2 peptides)
45	[nano_sae_route]	Route : Intradermal injection	descriptive
46	[nano_sae_date_administred]	Administration date dose 1	text (date_dmy), Required
47	[nano_sae_date_administred_2]<br>Show the field ONLY if:<br>[nano_sae_doses] = "2"	Administration date dose 2	text (date_dmy), Required
48	[nano_sae_causality]	Section Header: *AE Causality*	radio, Required

		Causality of event to intervention	1 No relationship<br>2 Possible<br>3 Probable<br>4 Definite
49	[nano_sae_causality_def]		descriptive<br>(Attachment: Guidelines for assessing the relationship of vaccine administration to an adverse event.pdf, Display format: Link)<br>Field Annotation: @HIDDEN-PDF
50	[nano_sae_table]	Section Header: *Relevant medical history*<br>Disease / Intervention /Allergy Start date Ongoing Stop date {nano_sae_disease} {nano_sae_date_disease} {nano_sae_disease_ongoing} {nano_sae_disease_stop} {nano_sae_disease_1} {nano_sae_date_disease_1} {nano_sae_disease_ongoing_1} {nano_sae_disease_stop_1} {nano_sae_disease_2} {nano_sae_date_disease_2} {nano_sae_disease_ongoing_2} {nano_sae_disease_stop_2} {nano_sae_disease_3} {nano_sae_date_disease_3} {nano_sae_disease_ongoing_3} {nano_sae_disease_stop_3} {nano_sae_disease_4} {nano_sae_date_disease_4} {nano_sae_disease_ongoing_4} {nano_sae_disease_stop_4}	descriptive
51	[nano_sae_disease]	Disease / Intervention /Allergy	text
52	[nano_sae_date_disease]	Start date	text
53	[nano_sae_disease_ongoing]	Ongoing ?	yesno<br>1 Yes<br>0 No
54	[nano_sae_disease_stop]	Stop date	text
55	[nano_sae_disease_1]	Disease / Intervention /Allergy	text
56	[nano_sae_date_disease_1]	Start date	text
57	[nano_sae_disease_ongoing_1]	Ongoing ?	yesno<br>1 Yes<br>0 No
58	[nano_sae_disease_stop_1]	Stop date	text
59	[nano_sae_disease_2]	Disease / Intervention /Allergy	text
60	[nano_sae_date_disease_2]	Start date	text
61	[nano_sae_disease_ongoing_2]	Ongoing ?	yesno<br>1 Yes<br>0 No
62	[nano_sae_disease_stop_2]	Stop date	text
63	[nano_sae_disease_3]	Disease / Intervention /Allergy	text

64	[nano_sae_date_disease_3]	Start date	text
65	[nano_sae_disease_ongoing_3]	Ongoing ?	yesno<br>1 Yes<br>0 No
66	[nano_sae_disease_stop_3]	Stop date	text
67	[nano_sae_disease_4]	Disease / Intervention /Allergy	text
68	[nano_sae_date_disease_4]	Start date	text
69	[nano_sae_disease_ongoing_4]	Ongoing ?	yesno<br>1 Yes<br>0 No
70	[nano_sae_disease_stop_4]	Stop date	text
71	[nano_sae_drug]	Section Header: *Concomitant medications*<br>Concomitant drug(s) (exclude those to treat event)	yesno, Required<br>1 Yes<br>0 No
72	[nano_sae_conc_count]<br>Show the field ONLY if:<br>[nano_sae_drug] ="1"	How many concomitant drug ?	dropdown, Required<br>1 1<br>2 2<br>3 3<br>4 4<br>5 5<br>6 6
73	[nano_sae_med_name]<br>Show the field ONLY if:<br>[nano_sae_drug] ="1" and [nano_sae_conc_count] > 0	Medication Name (Commercial name)	text, Required
74	[nano_sae_med_name_inter]<br>Show the field ONLY if:<br>[nano_sae_drug] ="1" and [nano_sae_conc_count] > 0	Medication Name (International nonproprietary name)	text, Required
75	[nano_sae_med_dose]<br>Show the field ONLY if:<br>[nano_sae_drug] ="1" and [nano_sae_conc_count] > 0	Dose	text (number), Required

76	[nano_sae_med_dose_unit]<br>Show the field ONLY if:<br>[nano_sae_drug] ="1" and [nano_sae_conc_count] > 0	Dose unit	radio, Required<br>1 Microgram<br>2 Miligram<br>3 Gram<br>4 Mililiter<br>5 Other (Specify)<br>6 Unknown
77	[nano_sae_med_dose_spec]<br>Show the field ONLY if:<br>[nano_sae_med_dose_unit] = "5"	Specify	text, Required
78	[nano_sae_med_dose_freq]<br>Show the field ONLY if:<br>[nano_sae_drug] ="1" and [nano_sae_conc_count] > 0	Frequency	radio, Required<br>1 Daily<br>2 Twice daily<br>3 Three times a day<br>4 Four times a day<br>5 4-6 hours<br>6 6-8 hours<br>7 PRN (ad needed)<br>8 Other (specify)
79	[nano_sae_med_freq_spec]<br>Show the field ONLY if:<br>[nano_sae_med_dose_freq] = "8"	Specify	text, Required
80	[nano_sae_med_route]<br>Show the field ONLY if:<br>[nano_sae_drug] ="1" and [nano_sae_conc_count] > 0	Route	radio, Required<br>1 Oral<br>2 Topical<br>3 Subcutaneous injection<br>4 Intravenous injection<br>5 Intramuscular injection<br>6 Rectal<br>7 Nasal<br>8 Inhaled<br>9 Transdermal<br>10 Vaginal<br>11 Other (Specify)
81	[nano_sae_med_route_spec]<br>Show the field ONLY if:<br>[nano_sae_med_route] = "11"	Specify	text, Required
82	[nano_sae_med_use]<br>Show the field ONLY if:<br>[nano_sae_drug] ="1" and [nano_sae_conc_cou	Indication(s) for use	text, Required

	nt] > 0		
83	[nano_sae_med_start]<br>Show the field ONLY if:<br>[nano_sae_drug] ="1" and [nano_sae_conc_count] > 0	Start Date	text (date_dmy), Required
84	[nano_sae_med_ongoing]<br>Show the field ONLY if:<br>[nano_sae_drug] ="1" and [nano_sae_conc_count] > 0	Ongoing	yesno, Required<br>1 Yes<br>0 No
85	[nano_sae_med_end_date]<br>Show the field ONLY if:<br>[nano_sae_drug] ="1" AND [nano_sae_med_ongoing] = "0" and [nano_sae_conc_count] > 0	End date	text (date_dmy), Required
86	[nano_sae_med_name_1]<br>Show the field ONLY if:<br>[nano_sae_drug] ="1" and [nano_sae_conc_count] > 1	Medication Name (Commercial name)	text, Required
87	[nano_sae_med_name_inter_1]<br>Show the field ONLY if:<br>[nano_sae_drug] ="1" and [nano_sae_conc_count] > 1	Medication Name (International nonproprietary name)	text, Required
88	[nano_sae_med_dose_1]<br>Show the field ONLY if:<br>[nano_sae_drug] ="1" and [nano_sae_conc_count] > 1	Dose	text (number, Min: 0, Max: 9999999), Required
89	[nano_sae_med_dose_unit_1]<br>Show the field ONLY if:<br>[nano_sae_drug] ="1" and [nano_sae_conc_count] > 1	Dose unit	radio, Required<br>1 Microgram<br>2 Miligram<br>3 Gram<br>4 Mililiter<br>5 Other (Specify)<br>6 Unknown
90	[nano_sae_med_dose_spec_1]<br>Show the field ONLY if:<br>[nano_sae_med_dose_unit_1] = "5"	Specify	text, Required
91	[nano_sae_med_dose_freq_1]<br>Show the field ONLY if:<br>[nano_sae_drug] ="1" and [nano_sae_conc_count] > 1	Frequency	radio, Required<br>1 Daily<br>2 Twice daily<br>3 Three times a day

			4 Four times a day<br>5 4-6 hours<br>6 6-8 hours<br>7 PRN (ad needed)<br>8 Other (specify)
92	[nano_sae_med_freq_spec_1]<br>Show the field ONLY if:<br>[nano_sae_med_dose_freq_1] = "8"	Specify	text, Required
93	[nano_sae_med_route_1]<br>Show the field ONLY if:<br>[nano_sae_drug] ="1" and [nano_sae_conc_count] > 1	Route	radio, Required<br>1 Oral<br>2 Topical<br>3 Subcutaneous injection<br>4 Intravenous injection<br>5 Intramuscular injection<br>6 Rectal<br>7 Nasal<br>8 Inhaled<br>9 Transdermal<br>10 Vaginal<br>11 Other (Specify)
94	[nano_sae_med_route_spec_1]<br>Show the field ONLY if:<br>[nano_sae_med_route_1] = "11"	Specify	text, Required
95	[nano_sae_med_use_1]<br>Show the field ONLY if:<br>[nano_sae_drug] ="1" and [nano_sae_conc_count] > 1	Indication(s) for use	text, Required
96	[nano_sae_med_start_1]<br>Show the field ONLY if:<br>[nano_sae_drug] ="1" and [nano_sae_conc_count] > 1	Start Date	text (date_dmy), Required
97	[nano_sae_med_ongoing_1]<br>Show the field ONLY if:<br>[nano_sae_drug] ="1" and [nano_sae_conc_count] > 1	Ongoing	yesno, Required<br>1 Yes<br>0 No
98	[nano_sae_med_end_date_1]<br>Show the field ONLY if:<br>[nano_sae_drug] ="1" AND [nano_sae_med_on	End date	text (date_dmy), Required

	going_1] = "0" and [nano_sae_conc_count] > 1		
99	[nano_sae_med_name_2]<br>Show the field ONLY if:<br>[nano_sae_drug] ="1" and [nano_sae_conc_count] > 2	Medication Name (Commercial name)	text, Required
100	[nano_sae_med_name_inter_2]<br>Show the field ONLY if:<br>[nano_sae_drug] ="1" and [nano_sae_conc_count] > 2	Medication Name (International nonproprietary name)	text, Required
101	[nano_sae_med_dose_2]<br>Show the field ONLY if:<br>[nano_sae_drug] ="1" and [nano_sae_conc_count] > 2	Dose	text (integer, Min: 0, Max: 9999999), Required
102	[nano_sae_med_dose_unit_2]<br>Show the field ONLY if:<br>[nano_sae_drug] ="1" and [nano_sae_conc_count] > 2	Dose unit	radio, Required<br>1 Microgram<br>2 Miligram<br>3 Gram<br>4 Mililiter<br>5 Other (Specify)<br>6 Unknown
103	[nano_sae_med_dose_spec_2]<br>Show the field ONLY if:<br>[nano_sae_med_dose_unit_2] = "5"	Specify	text, Required
104	[nano_sae_med_dose_freq_2]<br>Show the field ONLY if:<br>[nano_sae_drug] ="1" and [nano_sae_conc_count] > 2	Frequency	radio, Required<br>1 Daily<br>2 Twice daily<br>3 Three times a day<br>4 Four times a day<br>5 4-6 hours<br>6 6-8 hours<br>7 PRN (ad needed)<br>8 Other (specify)
105	[nano_sae_med_freq_spec_2]<br>Show the field ONLY if:<br>[nano_sae_med_dose_freq_2] = "8"	Specify	text, Required
106	[nano_sae_med_route_2]<br>Show the field ONLY if:<br>[nano_sae_drug] ="1" and [nano_sae_conc_count] > 2	Route	radio, Required<br>1 Oral<br>2 Topical<br>3 Subcutaneous injection<br>4 Intravenous injection

			5 Intramuscular injection<br>6 Rectal<br>7 Nasal<br>8 Inhaled<br>9 Transdermal<br>10 Vaginal<br>11 Other (Specify)
107	[nano_sae_med_route_spec_2]<br>Show the field ONLY if:<br>[nano_sae_med_route_2] = "11"	Specify	text, Required
108	[nano_sae_med_use_2]<br>Show the field ONLY if:<br>[nano_sae_drug] ="1" and [nano_sae_conc_count] > 2	Indication(s) for use	text, Required
109	[nano_sae_med_start_2]<br>Show the field ONLY if:<br>[nano_sae_drug] ="1" and [nano_sae_conc_count] > 2	Start Date	text (date_dmy), Required
110	[nano_sae_med_ongoing_2]<br>Show the field ONLY if:<br>[nano_sae_drug] ="1" and [nano_sae_conc_count] > 2	Ongoing	yesno, Required<br>1 Yes<br>0 No
111	[nano_sae_med_end_date_2]<br>Show the field ONLY if:<br>[nano_sae_drug] ="1" AND [nano_sae_med_ongoing_2] = "0" and [nano_sae_conc_count] > 2	End date	text (date_dmy), Required
112	[nano_sae_med_name_3]<br>Show the field ONLY if:<br>[nano_sae_drug] ="1" and [nano_sae_conc_count] > 3	Medication Name (Commercial name)	text, Required
113	[nano_sae_med_name_inter_3]<br>Show the field ONLY if:<br>[nano_sae_drug] ="1" and [nano_sae_conc_count] > 3	Medication Name (International nonproprietary name)	text, Required
114	[nano_sae_med_dose_3]<br>Show the field ONLY if:<br>[nano_sae_drug] ="1" and [nano_sae_conc_count] > 3	Dose	text (number, Min: 0, Max: 9999999), Required

115	[nano_sae_med_dose_unit_3]<br><br>Show the field ONLY if:<br>[nano_sae_drug] ="1" and [nano_sae_conc_count] > 3	Dose unit	radio, Required<br>1 Microgram<br>2 Miligram<br>3 Gram<br>4 Mililiter<br>5 Other (Specify)<br>6 Unknown
116	[nano_sae_med_dose_spec_3]<br><br>Show the field ONLY if:<br>[nano_sae_med_dose_unit_3] = "5"	Specify	text, Required
117	[nano_sae_med_dose_freq_3]<br><br>Show the field ONLY if:<br>[nano_sae_drug] ="1" and [nano_sae_conc_count] > 3	Frequency	radio, Required<br>1 Daily<br>2 Twice daily<br>3 Three times a day<br>4 Four times a day<br>5 4-6 hours<br>6 6-8 hours<br>7 PRN (ad needed)<br>8 Other (specify)
118	[nano_sae_med_freq_spec_3]<br><br>Show the field ONLY if:<br>[nano_sae_med_dose_freq_3] = "8"	Specify	text, Required
119	[nano_sae_med_route_3]<br><br>Show the field ONLY if:<br>[nano_sae_drug] ="1" and [nano_sae_conc_count] > 3	Route	radio, Required<br>1 Oral<br>2 Topical<br>3 Subcutaneous injection<br>4 Intravenous injection<br>5 Intramuscular injection<br>6 Rectal<br>7 Nasal<br>8 Inhaled<br>9 Transdermal<br>10 Vaginal<br>11 Other (Specify)
120	[nano_sae_med_route_spec_3]<br><br>Show the field ONLY if:<br>[nano_sae_med_route_3] = "11"	Specify	text, Required
121	[nano_sae_med_use_3]<br><br>Show the field ONLY if:<br>[nano_sae_drug] ="1" and [nano_sae_conc_cou	Indication(s) for use	text, Required

	nt] > 3		
122	[nano_sae_med_start_3]<br>Show the field ONLY if:<br>[nano_sae_drug] ="1" and [nano_sae_conc_count] > 3	Start Date	text (date_dmy), Required
123	[nano_sae_med_ongoing_3]<br>Show the field ONLY if:<br>[nano_sae_drug] ="1" and [nano_sae_conc_count] > 3	Ongoing	yesno, Required<br>1 Yes<br>0 No
124	[nano_sae_med_end_date_3]<br>Show the field ONLY if:<br>[nano_sae_drug] ="1" AND [nano_sae_med_ongoing_3] = "0" and [nano_sae_conc_count] > 3	End date	text (date_dmy), Required
125	[nano_sae_med_name_4]<br>Show the field ONLY if:<br>[nano_sae_drug] ="1" and [nano_sae_conc_count] > 4	Medication Name (Commercial name)	text, Required
126	[nano_sae_med_name_inter_4]<br>Show the field ONLY if:<br>[nano_sae_drug] ="1" and [nano_sae_conc_count] > 4	Medication Name (International nonproprietary name)	text, Required
127	[nano_sae_med_dose_4]<br>Show the field ONLY if:<br>[nano_sae_drug] ="1" and [nano_sae_conc_count] > 4	Dose	text (number, Min: 0, Max: 9999999), Required
128	[nano_sae_med_dose_unit_4]<br>Show the field ONLY if:<br>[nano_sae_drug] ="1" and [nano_sae_conc_count] > 4	Dose unit	radio, Required<br>1 Microgram<br>2 Miligram<br>3 Gram<br>4 Mililiter<br>5 Other (Specify)<br>6 Unknown
129	[nano_sae_med_dose_spec_4]<br>Show the field ONLY if:<br>[nano_sae_med_dose_unit_4] = "5"	Specify	text, Required
130	[nano_sae_med_dose_freq_4]<br>Show the field ONLY if:<br>[nano_sae_drug] ="1" and [nano_sae_conc_cou	Frequency	radio, Required<br>1 Daily<br>2 Twice daily<br>3 Three times a day

	nt] > 4		4 Four times a day<br>5 4-6 hours<br>6 6-8 hours<br>7 PRN (ad needed)<br>8 Other (specify)
131	[nano_sae_med_freq_spec_4]<br><br>Show the field ONLY if:<br>[nano_sae_med_dose_freq_4] = "8"	Specify	text, Required
132	[nano_sae_med_route_4]<br><br>Show the field ONLY if:<br>[nano_sae_drug] ="1" and [nano_sae_conc_count] > 4	Route	radio, Required<br>1 Oral<br>2 Topical<br>3 Subcutaneous injection<br>4 Intravenous injection<br>5 Intramuscular injection<br>6 Rectal<br>7 Nasal<br>8 Inhaled<br>9 Transdermal<br>10 Vaginal<br>11 Other (Specify)
133	[nano_sae_med_route_spec_4]<br><br>Show the field ONLY if:<br>[nano_sae_med_route_4] = "11"	Specify	text, Required
134	[nano_sae_med_use_4]<br><br>Show the field ONLY if:<br>[nano_sae_drug] ="1" and [nano_sae_conc_count] > 4	Indication(s) for use	text, Required
135	[nano_sae_med_start_4]<br><br>Show the field ONLY if:<br>[nano_sae_drug] ="1" and [nano_sae_conc_count] > 4	Start Date	text (date_dmy), Required
136	[nano_sae_med_ongoing_4]<br><br>Show the field ONLY if:<br>[nano_sae_drug] ="1" and [nano_sae_conc_count] > 4	Ongoing	yesno, Required<br>1 Yes<br>0 No
137	[nano_sae_med_end_date_4]<br><br>Show the field ONLY if:<br>[nano_sae_drug] ="1" AND [nano_sae_med_on	End date	text (date_dmy), Required

	going_4] = "0" and [nano_sae_conc_count] > 4		
138	[nano_sae_med_name_5]<br>Show the field ONLY if:<br>[nano_sae_drug] ="1" and [nano_sae_conc_count] > 5	Medication Name (Commercial name)	text, Required
139	[nano_sae_med_name_inter_5]<br>Show the field ONLY if:<br>[nano_sae_drug] ="1" and [nano_sae_conc_count] > 5	Medication Name (International nonproprietary name)	text, Required
140	[nano_sae_med_dose_5]<br>Show the field ONLY if:<br>[nano_sae_drug] ="1" and [nano_sae_conc_count] > 5	Dose	text (number, Min: 0, Max: 9999999), Required
141	[nano_sae_med_dose_unit_5]<br>Show the field ONLY if:<br>[nano_sae_drug] ="1" and [nano_sae_conc_count] > 5	Dose unit	radio, Required<br>1 Microgram<br>2 Miligram<br>3 Gram<br>4 Mililiter<br>5 Other (Specify)<br>6 Unknown
142	[nano_sae_med_dose_spec_5]<br>Show the field ONLY if:<br>[nano_sae_med_dose_unit_5] = "5"	Specify	text, Required
143	[nano_sae_med_dose_freq_5]<br>Show the field ONLY if:<br>[nano_sae_drug] ="1" and [nano_sae_conc_count] > 5	Frequency	radio, Required<br>1 Daily<br>2 Twice daily<br>3 Three times a day<br>4 Four times a day<br>5 4-6 hours<br>6 6-8 hours<br>7 PRN (ad needed)<br>8 Other (specify)
144	[nano_sae_med_freq_spec_5]<br>Show the field ONLY if:<br>[nano_sae_med_dose_freq_5] = "8"	Specify	text, Required
145	[nano_sae_med_route_5]<br>Show the field ONLY if:<br>[nano_sae_drug] ="1" and [nano_sae_conc_count] > 5	Route	radio, Required<br>1 Oral<br>2 Topical<br>3 Subcutaneous injection<br>4 Intravenous injection

			5 Intramuscular injection<br>6 Rectal<br>7 Nasal<br>8 Inhaled<br>9 Transdermal<br>10 Vaginal<br>11 Other (Specify)
146	[nano_sae_med_route_spec_5]<br>Show the field ONLY if:<br>[nano_sae_med_route_5] = "11"	Specify	text, Required
147	[nano_sae_med_use_5]<br>Show the field ONLY if:<br>[nano_sae_drug] ="1" and [nano_sae_conc_count] > 5	Indication(s) for use	text, Required
148	[nano_sae_med_start_5]<br>Show the field ONLY if:<br>[nano_sae_drug] ="1" and [nano_sae_conc_count] > 5	Start Date	text (date_dmy), Required
149	[nano_sae_med_ongoing_5]<br>Show the field ONLY if:<br>[nano_sae_drug] ="1" and [nano_sae_conc_count] > 5	Ongoing	yesno, Required<br>1 Yes<br>0 No
150	[nano_sae_med_end_date_5]<br>Show the field ONLY if:<br>[nano_sae_drug] ="1" AND [nano_sae_med_ongoing_5] = "0" and [nano_sae_conc_count] > 5	End date	text (date_dmy), Required
151	[nano_sae_test_performed]	Section Header: *Relevant paraclinic tests*<br>Have some paraclinical tests/procedures been performed ?	radio, Required<br>1 Yes<br>2 No<br>3 Not known
152	[nano_sae_lab_tests]<br>Show the field ONLY if:<br>[nano_sae_test_performed] = "1"	Laboratory tests	radio, Required<br>1 Yes<br>2 No<br>3 Not known
153	[nano_sae_radio_tests]<br>Show the field ONLY if:<br>[nano_sae_test_performed] = "1"	Radiological exams	radio, Required<br>1 Yes<br>2 No<br>3 Not known

154	[nano_sae_relevant_proced]<br>Show the field ONLY if:<br>[nano_sae_test_performed] = "1"	Other relevant diagnostic procedure(s)*	radio, Required<br>1 Yes<br>2 No<br>3 Not known
155	[nano_sae_specify]<br>Show the field ONLY if:<br>[nano_sae_relevant_proced] = "1"	Specify	text, Required
156	[nano_sae_main_result]<br>Show the field ONLY if:<br>[nano_sae_test_performed] = "1"	Please provide the main results of the exams performed	notes, Required
157	[nano_sae_text]<br>Show the field ONLY if:<br>[nano_sae_relevant_proced] = "1" OR [nano_sae_radio_tests] = "1" OR [nano_sae_lab_tests] = "1"	Do not forget to collect complementary exam, remove identifying data, code them, register them as source documents and send them to the PI in a separate email.	descriptive<br>Field Annotation: @HIDDEN-PDF
158	[nano_sae_action_taken_imp]	Section Header: *Actions taken and outcome*<br>Which action(s) was/were taken with IMP ? (Several possible)	checkbox, Required<br>a nano_sae_action_taken_imp___a No acti[illegible]<br>b nano_sae_action_taken_imp___b Dose n[illegible] change<br>c nano_sae_action_taken_imp___c Dose reduce[illegible]<br>d nano_sae_action_taken_imp___d Dose increas[illegible]<br>e nano_sae_action_taken_imp___e Drug tempor[illegible] interru[illegible]<br>f nano_sae_action_taken_imp___f Drug withdra[illegible]
159	[nano_sae_action_taken_imp_sp]<br>Show the field ONLY if:<br>[nano_sae_action_taken_imp(c)] = '1' or [nano_sae_action_taken_imp(d)] = '1' or [nano_sae_action_taken_imp(e)] = '1'	Specify	text, Required
160	[nano_sae_action_taken]	Which action(s) was/were taken with the participant ? (Several possible)	checkbox, Required<br>f nano_sae_action_taken___f No action<br>g nano_sae_action_taken___g Medication treatments<br>h nano_sae_action_taken___h Non medication treatments
161	[nano_sae_action_taken_spec]<br>Show the field ONLY if:	Specify	text, Required

	[nano_sae_action_taken(h)] = '1'		
162	[nano_sae_count]<br>Show the field ONLY if:<br>[nano_sae_action_taken(g)] = '1'	How many medication ?	dropdown, Required<br>1 1<br>2 2<br>3 3<br>4 4<br>5 5<br>6 6<br>7 7<br>8 8<br>9 9
163	[nano_sae_action_med_name]<br>Show the field ONLY if:<br>[nano_sae_action_taken(g)] and [nano_sae_count] > 0	Medication Name (Commercial name)	text, Required
164	[nano_sae_action_med_name_inter]<br>Show the field ONLY if:<br>[nano_sae_action_taken(g)] and [nano_sae_count] > 0	Medication Name (International nonproprietary name)	text, Required
165	[nano_sae_action_med_dose]<br>Show the field ONLY if:<br>[nano_sae_action_taken(g)] and [nano_sae_count] > 0	Dose	text (number, Min: 0, Max: 9999999), Required
166	[nano_sae_action_med_unit]<br>Show the field ONLY if:<br>[nano_sae_action_taken(g)] and [nano_sae_count] > 0	Dose unit	radio, Required<br>1 Microgram<br>2 Miligram<br>3 Gram<br>4 Mililiter<br>5 Other (Specify)<br>6 Unknown
167	[nano_sae_action_med_unit_spec]<br>Show the field ONLY if:<br>[nano_sae_action_med_unit] = "5"	Specify	text, Required
168	[nano_sae_action_med_freq]<br>Show the field ONLY if:<br>[nano_sae_action_taken(g)] and [nano_sae_count] > 0	Frequency	radio, Required<br>1 Daily<br>2 Twice daily<br>3 Three times a day<br>4 Four times a day<br>5 4-6 hours

			6 6-8 hours<br>7 PRN (ad needed)<br>8 Other (specify)
169	[nano_sae_action_med_freq_spec]<br>Show the field ONLY if:<br>[nano_sae_action_med_freq] = "8"	Specify	text, Required
170	[nano_sae_action_med_route]<br>Show the field ONLY if:<br>[nano_sae_action_taken(g)] and [nano_sae_count] > 0	Route	radio, Required<br>1 Oral<br>2 Topical<br>3 Subcutaneous injection<br>4 Intravenous injection<br>5 Intramuscular injection<br>6 Rectal<br>7 Nasal<br>8 Inhaled<br>9 Transdermal<br>10 Vaginal<br>11 Other (Specify)
171	[nano_sae_action_med_route_spec]<br>Show the field ONLY if:<br>[nano_sae_action_med_route] = "11"	Specify	text, Required
172	[nano_sae_action_med_date]<br>Show the field ONLY if:<br>[nano_sae_action_taken(g)] and [nano_sae_count] > 0	Start Date	text (date_dmy), Required
173	[nano_sae_action_med_ongoing]<br>Show the field ONLY if:<br>[nano_sae_action_taken(g)] and [nano_sae_count] > 0	Ongoing	yesno, Required<br>1 Yes<br>0 No
174	[nano_sae_action_med_end]<br>Show the field ONLY if:<br>[nano_sae_action_taken(g)] and [nano_sae_count] > 0 and [nano_sae_action_med_ongoing] = "0"	End date	text (date_dmy), Required
175	[nano_sae_action_med_name_1]<br>Show the field ONLY if:<br>[nano_sae_action_taken(g)] and [nano_sae_count] > 1	Medication Name (Commercial name)	text, Required

176	[nano_sae_action_med_name_inter_1]<br><br>Show the field ONLY if:<br>[nano_sae_action_taken(g)] and [nano_sae_count] > 1	Medication Name (International nonproprietary name)	text, Required
177	[nano_sae_action_med_dose_1]<br><br>Show the field ONLY if:<br>[nano_sae_action_taken(g)] and [nano_sae_count] > 1	Dose	text (number, Min: 0, Max: 9999999), Required
178	[nano_sae_action_med_unit_1]<br><br>Show the field ONLY if:<br>[nano_sae_action_taken(g)] and [nano_sae_count] > 1	Dose unit	radio, Required<br>1 Microgram<br>2 Miligram<br>3 Gram<br>4 Mililiter<br>5 Other (Specify)<br>6 Unknown
179	[nano_sae_action_med_unit_spec_1]<br><br>Show the field ONLY if:<br>[nano_sae_action_med_unit_1] = "5"	Specify	text, Required
180	[nano_sae_action_med_freq_1]<br><br>Show the field ONLY if:<br>[nano_sae_action_taken(g)] and [nano_sae_count] > 1	Frequency	radio, Required<br>1 Daily<br>2 Twice daily<br>3 Three times a day<br>4 Four times a day<br>5 4-6 hours<br>6 6-8 hours<br>7 PRN (ad needed)<br>8 Other (specify)
181	[nano_sae_med_action_freq_spec_1]<br><br>Show the field ONLY if:<br>[nano_sae_action_med_freq_1] = "8"	Specify	text, Required
182	[nano_sae_action_med_route_1]<br><br>Show the field ONLY if:<br>[nano_sae_action_taken(g)] and [nano_sae_count] > 1	Route	radio, Required<br>1 Oral<br>2 Topical<br>3 Subcutaneous injection<br>4 Intravenous injection<br>5 Intramuscular injection<br>6 Rectal<br>7 Nasal<br>8 Inhaled<br>9 Transdermal

			10 Vaginal<br>11 Other (Specify)
183	[nano_sae_med_action_route_spec_1]<br>Show the field ONLY if:<br>[nano_sae_action_med_route_1] = "11"	Specify	text, Required
184	[nano_sae_action_med_date_1]<br>Show the field ONLY if:<br>[nano_sae_action_taken(g)] and [nano_sae_count] > 1	Start Date	text (date_dmy), Required
185	[nano_sae_action_med_ongoing_1]<br>Show the field ONLY if:<br>[nano_sae_action_taken(g)] and [nano_sae_count] > 1	Ongoing	yesno, Required<br>1 Yes<br>0 No
186	[nano_sae_action_med_end_1]<br>Show the field ONLY if:<br>[nano_sae_action_taken(g)] and [nano_sae_count] > 1 and [nano_sae_action_med_ongoing_1] = "0"	End date	text (date_dmy), Required
187	[nano_sae_action_med_name_2]<br>Show the field ONLY if:<br>[nano_sae_action_taken(g)] and [nano_sae_count] > 2	Medication Name (Commercial name)	text, Required
188	[nano_sae_action_med_name_inter_2]<br>Show the field ONLY if:<br>[nano_sae_action_taken(g)] and [nano_sae_count] > 2	Medication Name (International nonproprietary name)	text, Required
189	[nano_sae_action_med_dose_2]<br>Show the field ONLY if:<br>[nano_sae_action_taken(g)] and [nano_sae_count] > 2	Dose	text (number, Min: 0, Max: 9999999), Required
190	[nano_sae_action_med_unit_2]<br>Show the field ONLY if:<br>[nano_sae_action_taken(g)] and [nano_sae_count] > 2	Dose unit	radio, Required<br>1 Microgram<br>2 Miligram<br>3 Gram<br>4 Mililiter<br>5 Other (Specify)<br>6 Unknown

191	[nano_sae_action_med_unit_spec_2]<br><br>Show the field ONLY if:<br>[nano_sae_action_med_unit_2] = "5"	Specify	text, Required
192	[nano_sae_action_med_freq_2]<br><br>Show the field ONLY if:<br>[nano_sae_action_taken(g)] and [nano_sae_count] > 2	Frequency	radio, Required<br>1 Daily<br>2 Twice daily<br>3 Three times a day<br>4 Four times a day<br>5 4-6 hours<br>6 6-8 hours<br>7 PRN (ad needed)<br>8 Other (specify)
193	[nano_sae_med_action_freq_spec_2]<br><br>Show the field ONLY if:<br>[nano_sae_action_med_freq_2] = "8"	Specify	text, Required
194	[nano_sae_action_med_route_2]<br><br>Show the field ONLY if:<br>[nano_sae_action_taken(g)] and [nano_sae_count] > 2	Route	radio, Required<br>1 Oral<br>2 Topical<br>3 Subcutaneous injection<br>4 Intravenous injection<br>5 Intramuscular injection<br>6 Rectal<br>7 Nasal<br>8 Inhaled<br>9 Transdermal<br>10 Vaginal<br>11 Other (Specify)
195	[nano_sae_med_action_route_spec_2]<br><br>Show the field ONLY if:<br>[nano_sae_action_med_route_2] = "11"	Specify	text, Required
196	[nano_sae_action_med_date_2]<br><br>Show the field ONLY if:<br>[nano_sae_action_taken(g)] and [nano_sae_count] > 2	Start Date	text (date_dmy), Required
197	[nano_sae_action_med_ongoing_2]<br><br>Show the field ONLY if:<br>[nano_sae_action_taken(g)] and [nano_sae_count] > 2	Ongoing	yesno, Required<br>1 Yes<br>0 No

198	[nano_sae_action_med_end_2]<br><br>Show the field ONLY if:<br>[nano_sae_action_taken(g)] and [nano_sae_count] > 2 and [nano_sae_action_med_ongoing_2] = "0"	End date	text (date_dmy), Required
199	[nano_sae_action_med_name_3]<br><br>Show the field ONLY if:<br>[nano_sae_action_taken(g)] and [nano_sae_count] > 3	Medication Name (Commercial name)	text, Required
200	[nano_sae_action_med_name_inter_3]<br><br>Show the field ONLY if:<br>[nano_sae_action_taken(g)] and [nano_sae_count] > 3	Medication Name (International nonproprietary name)	text, Required
201	[nano_sae_action_med_dose_3]<br><br>Show the field ONLY if:<br>[nano_sae_action_taken(g)] and [nano_sae_count] > 3	Dose	text (number, Min: 0, Max: 9999999), Required
202	[nano_sae_action_med_unit_3]<br><br>Show the field ONLY if:<br>[nano_sae_action_taken(g)] and [nano_sae_count] > 3	Dose unit	radio, Required<br>1 Microgram<br>2 Miligram<br>3 Gram<br>4 Mililiter<br>5 Other (Specify)<br>6 Unknown
203	[nano_sae_action_med_unit_spec_3]<br><br>Show the field ONLY if:<br>[nano_sae_action_med_unit_3] = "5"	Specify	text, Required
204	[nano_sae_action_med_freq_3]<br><br>Show the field ONLY if:<br>[nano_sae_action_taken(g)] and [nano_sae_count] > 3	Frequency	radio, Required<br>1 Daily<br>2 Twice daily<br>3 Three times a day<br>4 Four times a day<br>5 4-6 hours<br>6 6-8 hours<br>7 PRN (ad needed)<br>8 Other (specify)
205	[nano_sae_med_action_freq_spec_3]<br><br>Show the field ONLY if:	Specify	text, Required

	[nano_sae_action_med_freq_3] = "8"		
206	[nano_sae_action_med_route_3]<br>Show the field ONLY if:<br>[nano_sae_action_taken(g)] and [nano_sae_count] > 3	Route	radio, Required<br>1 Oral<br>2 Topical<br>3 Subcutaneous injection<br>4 Intravenous injection<br>5 Intramuscular injection<br>6 Rectal<br>7 Nasal<br>8 Inhaled<br>9 Transdermal<br>10 Vaginal<br>11 Other (Specify)
207	[nano_sae_med_action_route_spec_3]<br>Show the field ONLY if:<br>[nano_sae_action_med_route_3] = "11"	Specify	text, Required
208	[nano_sae_action_med_date_3]<br>Show the field ONLY if:<br>[nano_sae_action_taken(g)] and [nano_sae_count] > 3	Start Date	text (date_dmy), Required
209	[nano_sae_action_med_ongoing_3]<br>Show the field ONLY if:<br>[nano_sae_action_taken(g)] and [nano_sae_count] > 3	Ongoing	yesno, Required<br>1 Yes<br>0 No
210	[nano_sae_action_med_end_3]<br>Show the field ONLY if:<br>[nano_sae_action_taken(g)] and [nano_sae_count] > 3 and [nano_sae_action_med_ongoing_3] = "0"	End date	text (date_dmy), Required
211	[nano_sae_action_med_name_4]<br>Show the field ONLY if:<br>[nano_sae_action_taken(g)] and [nano_sae_count] > 4	Medication Name (Commercial name)	text, Required
212	[nano_sae_action_med_name_inter_4]<br>Show the field ONLY if:<br>[nano_sae_action_taken(g)] and [nano_sae_count] > 4	Medication Name (International nonproprietary name)	text, Required

213	[nano_sae_action_med_dose_4]<br><br>Show the field ONLY if:<br>[nano_sae_action_taken(g)] and [nano_sae_count] > 4	Dose	text (number, Min: 0, Max: 9999999), Required
214	[nano_sae_action_med_unit_4]<br><br>Show the field ONLY if:<br>[nano_sae_action_taken(g)] and [nano_sae_count] > 4	Dose unit	radio, Required<br>1 Microgram<br>2 Miligram<br>3 Gram<br>4 Mililiter<br>5 Other (Specify)<br>6 Unknown
215	[nano_sae_action_med_unit_spec_4]<br><br>Show the field ONLY if:<br>[nano_sae_action_med_unit_4] = "5"	Specify	text, Required
216	[nano_sae_action_med_freq_4]<br><br>Show the field ONLY if:<br>[nano_sae_action_taken(g)] and [nano_sae_count] > 4	Frequency	radio, Required<br>1 Daily<br>2 Twice daily<br>3 Three times a day<br>4 Four times a day<br>5 4-6 hours<br>6 6-8 hours<br>7 PRN (ad needed)<br>8 Other (specify)
217	[nano_sae_med_action_freq_spec_4]<br><br>Show the field ONLY if:<br>[nano_sae_action_med_freq_4] = "8"	Specify	text, Required
218	[nano_sae_action_med_route_4]<br><br>Show the field ONLY if:<br>[nano_sae_action_taken(g)] and [nano_sae_count] > 4	Route	radio, Required<br>1 Oral<br>2 Topical<br>3 Subcutaneous injection<br>4 Intravenous injection<br>5 Intramuscular injection<br>6 Rectal<br>7 Nasal<br>8 Inhaled<br>9 Transdermal<br>10 Vaginal<br>11 Other (Specify)
219	[nano_sae_med_action_route_spec_4]<br><br>Show the field ONLY if:	Specify	text, Required

	[nano_sae_action_med_route_4] = "11"		
220	[nano_sae_action_med_date_4]<br><br>Show the field ONLY if:<br>[nano_sae_action_taken(g)] and [nano_sae_count] > 4	Start Date	text (date_dmy), Required
221	[nano_sae_action_med_ongoing_4]<br><br>Show the field ONLY if:<br>[nano_sae_action_taken(g)] and [nano_sae_count] > 4	Ongoing	yesno, Required<br>1 Yes<br>0 No
222	[nano_sae_action_med_end_4]<br><br>Show the field ONLY if:<br>[nano_sae_action_taken(g)] and [nano_sae_count] > 4 and [nano_sae_action_med_ongoing_4] = "0"	End date	text (date_dmy), Required
223	[nano_sae_action_med_name_5]<br><br>Show the field ONLY if:<br>[nano_sae_action_taken(g)] and [nano_sae_count] > 5	Medication Name (Commercial name)	text, Required
224	[nano_sae_action_med_name_inter_5]<br><br>Show the field ONLY if:<br>[nano_sae_action_taken(g)] and [nano_sae_count] > 5	Medication Name (International nonproprietary name)	text, Required
225	[nano_sae_action_med_dose_5]<br><br>Show the field ONLY if:<br>[nano_sae_action_taken(g)] and [nano_sae_count] > 5	Dose	text (number, Min: 0, Max: 9999999), Required
226	[nano_sae_action_med_unit_5]<br><br>Show the field ONLY if:<br>[nano_sae_action_taken(g)] and [nano_sae_count] > 5	Dose unit	radio, Required<br>1 Microgram<br>2 Miligram<br>3 Gram<br>4 Mililiter<br>5 Other (Specify)<br>6 Unknown
227	[nano_sae_action_med_unit_spec_5]<br><br>Show the field ONLY if:<br>[nano_sae_action_med_unit_5] = "5"	Specify	text, Required

228	[nano_sae_action_med_freq_5]<br>Show the field ONLY if:<br>[nano_sae_action_taken(g)] and [nano_sae_count] > 5	Frequency	radio, Required<br>1 Daily<br>2 Twice daily<br>3 Three times a day<br>4 Four times a day<br>5 4-6 hours<br>6 6-8 hours<br>7 PRN (ad needed)<br>8 Other (specify)
229	[nano_sae_med_action_freq_spec_5]<br>Show the field ONLY if:<br>[nano_sae_action_med_freq_5] = "8"	Specify	text, Required
230	[nano_sae_action_med_route_5]<br>Show the field ONLY if:<br>[nano_sae_action_taken(g)] and [nano_sae_count] > 5	Route	radio, Required<br>1 Oral<br>2 Topical<br>3 Subcutaneous injection<br>4 Intravenous injection<br>5 Intramuscular injection<br>6 Rectal<br>7 Nasal<br>8 Inhaled<br>9 Transdermal<br>10 Vaginal<br>11 Other (Specify)
231	[nano_sae_med_action_route_spec_5]<br>Show the field ONLY if:<br>[nano_sae_action_med_route_5] = "11"	Specify	text, Required
232	[nano_sae_action_med_date_5]<br>Show the field ONLY if:<br>[nano_sae_action_taken(g)] and [nano_sae_count] > 5	Start Date	text (date_dmy), Required
233	[nano_sae_action_med_ongoing_5]<br>Show the field ONLY if:<br>[nano_sae_action_taken(g)] and [nano_sae_count] > 5	Ongoing	yesno, Required<br>1 Yes<br>0 No
234	[nano_sae_action_med_end_5]<br>Show the field ONLY if:<br>[nano_sae_action_taken(g)] and [nano_sae_count] > 5 and [nano_sae_a	End date	text (date_dmy), Required

	ction_med_ongoing_5] = "0"		
235	[nano_sae_action_med_name_6]<br>Show the field ONLY if:<br>[nano_sae_action_taken(g)] and [nano_sae_count] > 6	Medication Name (Commercial name)	text, Required
236	[nano_sae_action_med_name_inter_6]<br>Show the field ONLY if:<br>[nano_sae_action_taken(g)] and [nano_sae_count] > 6	Medication Name (International nonproprietary name)	text, Required
237	[nano_sae_action_med_dose_6]<br>Show the field ONLY if:<br>[nano_sae_action_taken(g)] and [nano_sae_count] > 6	Dose	text (number, Min: 0, Max: 9999999), Required
238	[nano_sae_action_med_unit_6]<br>Show the field ONLY if:<br>[nano_sae_action_taken(g)] and [nano_sae_count] > 6	Dose unit	radio, Required<br>1 Microgram<br>2 Miligram<br>3 Gram<br>4 Mililiter<br>5 Other (Specify)<br>6 Unknown
239	[nano_sae_action_med_unit_spec_6]<br>Show the field ONLY if:<br>[nano_sae_action_med_unit_6] = "5"	Specify	text, Required
240	[nano_sae_action_med_freq_6]<br>Show the field ONLY if:<br>[nano_sae_action_taken(g)] and [nano_sae_count] > 6	Frequency	radio, Required<br>1 Daily<br>2 Twice daily<br>3 Three times a day<br>4 Four times a day<br>5 4-6 hours<br>6 6-8 hours<br>7 PRN (ad needed)<br>8 Other (specify)
241	[nano_sae_med_action_freq_spec_6]<br>Show the field ONLY if:<br>[nano_sae_action_med_freq_6] = "8"	Specify	text, Required
242	[nano_sae_action_med_route_6]<br>Show the field ONLY if:	Route	radio, Required<br>1 Oral<br>2 Topical

	[nano_sae_action_taken(g)] and [nano_sae_count] > 6		3 Subcutaneous injection<br>4 Intravenous injection<br>5 Intramuscular injection<br>6 Rectal<br>7 Nasal<br>8 Inhaled<br>9 Transdermal<br>10 Vaginal<br>11 Other (Specify)
243	[nano_sae_med_action_route_spec_6]<br><br>Show the field ONLY if:<br>[nano_sae_action_med_route_6] = "11"	Specify	text, Required
244	[nano_sae_action_med_date_6]<br><br>Show the field ONLY if:<br>[nano_sae_action_taken(g)] and [nano_sae_count] > 6	Start Date	text (date_dmy), Required
245	[nano_sae_action_med_ongoing_6]<br><br>Show the field ONLY if:<br>[nano_sae_action_taken(g)] and [nano_sae_count] > 6	Ongoing	yesno, Required<br>1 Yes<br>0 No
246	[nano_sae_action_med_end_6]<br><br>Show the field ONLY if:<br>[nano_sae_action_taken(g)] and [nano_sae_count] > 6 and [nano_sae_action_med_ongoing_6] = "0"	End date	text (date_dmy), Required
247	[nano_sae_action_med_name_7]<br><br>Show the field ONLY if:<br>[nano_sae_action_taken(g)] and [nano_sae_count] > 7	Medication Name (Commercial name)	text, Required
248	[nano_sae_action_med_name_inter_7]<br><br>Show the field ONLY if:<br>[nano_sae_action_taken(g)] and [nano_sae_count] > 7	Medication Name (International nonproprietary name)	text, Required
249	[nano_sae_action_med_dose_7]<br><br>Show the field ONLY if:<br>[nano_sae_action_taken(g)] and [nano_sae_count] > 7	Dose	text (number, Min: 0, Max: 9999999), Required

250	[nano_sae_action_med_unit_7]<br>Show the field ONLY if:<br>[nano_sae_action_taken(g)] and [nano_sae_count] > 7	Dose unit	radio, Required<br>1 Microgram<br>2 Miligram<br>3 Gram<br>4 Mililiter<br>5 Other (Specify)<br>6 Unknown
251	[nano_sae_action_med_unit_spec_7]<br>Show the field ONLY if:<br>[nano_sae_action_med_unit_7] = "5"	Specify	text, Required
252	[nano_sae_action_med_freq_7]<br>Show the field ONLY if:<br>[nano_sae_action_taken(g)] and [nano_sae_count] > 7	Frequency	radio, Required<br>1 Daily<br>2 Twice daily<br>3 Three times a day<br>4 Four times a day<br>5 4-6 hours<br>6 6-8 hours<br>7 PRN (ad needed)<br>8 Other (specify)
253	[nano_sae_med_action_freq_spec_7]<br>Show the field ONLY if:<br>[nano_sae_action_med_freq_7] = "8"	Specify	text, Required
254	[nano_sae_action_med_route_7]<br>Show the field ONLY if:<br>[nano_sae_action_taken(g)] and [nano_sae_count] > 7	Route	radio, Required<br>1 Oral<br>2 Topical<br>3 Subcutaneous injection<br>4 Intravenous injection<br>5 Intramuscular injection<br>6 Rectal<br>7 Nasal<br>8 Inhaled<br>9 Transdermal<br>10 Vaginal<br>11 Other (Specify)
255	[nano_sae_med_action_route_spec_7]<br>Show the field ONLY if:<br>[nano_sae_action_med_route_7] = "11"	Specify	text, Required
256	[nano_sae_action_med_date_7]<br>Show the field ONLY if:	Start Date	text (date_dmy), Required

	[nano_sae_action_taken(g)] and [nano_sae_count] > 7		
257	[nano_sae_action_med_ongoing_7]<br><br>Show the field ONLY if:<br>[nano_sae_action_taken(g)] and [nano_sae_count] > 7	Ongoing	yesno, Required<br>1 Yes<br>0 No
258	[nano_sae_action_med_end_7]<br><br>Show the field ONLY if:<br>[nano_sae_action_taken(g)] and [nano_sae_count] > 7 and [nano_sae_action_med_ongoing_7] = "0"	End date	text (date_dmy), Required
259	[nano_sae_action_med_name_8]<br><br>Show the field ONLY if:<br>[nano_sae_action_taken(g)] and [nano_sae_count] > 8	Medication Name (Commercial name)	text, Required
260	[nano_sae_action_med_name_inter_8]<br><br>Show the field ONLY if:<br>[nano_sae_action_taken(g)] and [nano_sae_count] > 8	Medication Name (International nonproprietary name)	text, Required
261	[nano_sae_action_med_dose_8]<br><br>Show the field ONLY if:<br>[nano_sae_action_taken(g)] and [nano_sae_count] > 8	Dose	text (number, Min: 0, Max: 9999999), Required
262	[nano_sae_action_med_unit_8]<br><br>Show the field ONLY if:<br>[nano_sae_action_taken(g)] and [nano_sae_count] > 8	Dose unit	radio, Required<br>1 Microgram<br>2 Miligram<br>3 Gram<br>4 Mililiter<br>5 Other (Specify)<br>6 Unknown
263	[nano_sae_action_med_unit_spec_8]<br><br>Show the field ONLY if:<br>[nano_sae_action_med_unit_8] = "5"	Specify	text, Required
264	[nano_sae_action_med_freq_8]<br><br>Show the field ONLY if:<br>[nano_sae_action_taken(g)] and [nano_sae_count] > 8	Frequency	radio, Required<br>1 Daily<br>2 Twice daily<br>3 Three times a day<br>4 Four times a day

			5 4-6 hours<br>6 6-8 hours<br>7 PRN (ad needed)<br>8 Other (specify)
265	[nano_sae_med_action_freq_spec_8]<br><br>Show the field ONLY if:<br>[nano_sae_action_med_freq_8] = "8"	Specify	text, Required
266	[nano_sae_action_med_route_8]<br><br>Show the field ONLY if:<br>[nano_sae_action_taken(g)] and [nano_sae_count] > 8	Route	radio, Required<br>1 Oral<br>2 Topical<br>3 Subcutaneous injection<br>4 Intravenous injection<br>5 Intramuscular injection<br>6 Rectal<br>7 Nasal<br>8 Inhaled<br>9 Transdermal<br>10 Vaginal<br>11 Other (Specify)
267	[nano_sae_med_action_route_spec_8]<br><br>Show the field ONLY if:<br>[nano_sae_action_med_route_8] = "11"	Specify	text, Required
268	[nano_sae_action_med_date_8]<br><br>Show the field ONLY if:<br>[nano_sae_action_taken(g)] and [nano_sae_count] > 8	Start Date	text (date_dmy), Required
269	[nano_sae_action_med_ongoing_8]<br><br>Show the field ONLY if:<br>[nano_sae_action_taken(g)] and [nano_sae_count] > 8	Ongoing	yesno, Required<br>1 Yes<br>0 No
270	[nano_sae_action_med_end_8]<br><br>Show the field ONLY if:<br>[nano_sae_action_taken(g)] and [nano_sae_count] > 8 and [nano_sae_action_med_ongoing_8] = "0"	End date	text (date_dmy), Required
271	[nano_sae_react_abate]	Did the reaction abate after stopping the vaccination ?	radio, Required<br>1 Yes<br>2 No

			3 Not applicable
272	[nano_sae_react_reappear]	Did the reaction reappear after the second dose of the vaccination ?	radio, Required<br>1 Yes<br>2 No<br>3 Not applicable
273	[nano_sae_outcome]	Issue of event	radio, Required<br>1 Resolved (for date, last day of symptoms)<br>2 Resolved with sequelae<br>3 Ongoing<br>4 Stabilisation<br>5 Improvement<br>6 Agravation<br>7 Fatal<br>8 Unknown
274	[nano_sae_outcome_date]<br>Show the field ONLY if:<br>[nano_sae_outcome] = '2' or [nano_sae_outcome] = '1' or [nano_sae_outcome] = '7'	Date	text (date_dmy), Required
275	[nano_sae_outcome_spec]<br>Show the field ONLY if:<br>[nano_sae_outcome] = '8'	Specify	text, Required
276	[nano_sae_autopsy]<br>Show the field ONLY if:<br>[nano_sae_outcome] = '7'	Autopsy performed	radio, Required<br>1 Yes<br>2 No<br>3 Not known
277	[nano_sae_autopsy_report]<br>Show the field ONLY if:<br>[nano_sae_autopsy] = '1'	Autopsy report available	radio, Required<br>1 Yes<br>2 No<br>3 Not known
278	[nano_sae_text_2]<br>Show the field ONLY if:<br>[nano_sae_autopsy_report] = '1'	Do not forget to collect the report, remove identifying data, code it, register it as source documents and send it to the PI in a separate email.	descriptive<br>Field Annotation: @HIDDEN-PDF
279	[nano_sae_doc_sent]	Source document sent ?	yesno, Required<br>1 Yes<br>0 No
280	[nano_sae_doc_spec]<br>Show the field ONLY if:<br>[nano_sae_doc_sent] = "1"	Specify	text, Required

281	[nano_sae_investigator_name]	Section Header: *Investigator Signature*<br>Name of investigator	text, Required
282	[nano_sae_inv_sign_date]	Date	text (date_dmy), Required
283	[nano_sae_investigator_sign]	Signature of investigator	file (signature), Required
284	[nano_sae_send_report]	Send the report to the PI for modification/validation	yesno, Required<br>1 Yes<br>0 No<br>Field Annotation: @HIDDEN-PDF
285	[nano_sae_approved]	Section Header: *SAE report PI evaluation*<br>SAE report approved by PI	yesno, Required<br>1 Yes<br>0 No<br>Field Annotation: @HIDE_BY_ROLE=PI
286	[nano_sae_add_comment]	Additionnal comment	yesno, Required<br>1 Yes<br>0 No<br>Field Annotation: @HIDE_BY_ROLE=PI
287	[nano_sae_comment_specify]<br>Show the field ONLY if:<br>[nano_sae_add_comment] = "1" or [nano_sae_add_comment] = "1"	Specify	text, Required<br>Field Annotation: @HIDE_BY_ROLE=PI
288	[nano_sae_pi_name_bottom]	Name of PI	text, Required<br>Field Annotation: @HIDE_BY_ROLE=PI
289	[nano_sae_pi_sign_date]	Date	text (date_dmy), Required<br>Field Annotation: @HIDE_BY_ROLE=PI
290	[nano_sae_sign_pi]	Signature of PI	file (signature), Required<br>Field Annotation: @HIDE_BY_ROLE=PI
291	[nano_sae_text_3]	All SAE and AESI must be reported immediately and within a maximum of 24 hours to the Sponsor	descriptive<br>Field Annotation: @HIDE_BY_ROLE=PI @HIDDEN-PDF
292	[nano_sae_pdf]	Generated PDF that will be sent to sponsor	file, Required<br>Field Annotation: @HIDE_BY_ROLE=PI @HIDDEN-PDF @HIDDEN
293	[nano_sae_send_sponsor]	Send the PDF report to the Sponsor	yesno, Required<br>1 Yes<br>0 No<br>Field Annotation: @HIDE_BY_ROLE=PI @HIDDEN-PDF
294	[serious_adverse_event_form_complete]	Section Header: *Form Status*<br>Complete?	dropdown<br>0 Incomplete<br>1 Unverified<br>2 Complete

Instrument: Serious Adverse Event Form Followup (serious_adverse_event_form_followup)

295	[nano_sae_sae_number_fup]	SAE number	text, Required
296	[nano_sae_custom_record_id_fup]	Custom record id	text, Required<br>Field Annotation: @HIDDEN @HIDDEN-PDF
297	[nano_sae_title_fup]	Title of research project	notes, Required<br>Custom alignment: RH<br>Field Annotation: @DEFAULT = 'naNO-DENGUE: A Phase-I study of a nanoparticle-based peptide vaccine against Dengue virus'
298	[nano_sae_sponsor_name_fup]	Name of Sponsor	text, Required<br>Custom alignment: RH<br>Field Annotation: @DEFAULT = 'Emergex Vaccines Holding Limited'
299	[nano_sae_sponsor_adress_fup]	Address of Sponsor	notes, Required<br>Custom alignment: RH<br>Field Annotation: @DEFAULT = '4 & 5 Dunmore Court Wootton Road, Abingdon, Oxfordshire, England, OX13 6BH'
300	[nano_sae_pi_name_fup]	Name of Principal Investigator	text, Required<br>Custom alignment: RH<br>Field Annotation: @DEFAULT = 'Prof. Blaise Genton'
301	[nano_sae_product_name_fup]	Investigational Medicinal Product Name	text, Required<br>Field Annotation: @DEFAULT="PepGNP-DENGUE vaccine"
302	[nano_sae_pi_adress_fup]	Adress of Principal Investigator	notes, Required<br>Custom alignment: RH<br>Field Annotation: @DEFAULT = 'Unisanté, Département Formation, recherche et innovation Policlinique de médecine tropicale, voyages et vaccinations Rue du Bugnon 44 1011 Lausanne, Suisse Blaise.genton@unisante.ch M :+41 79 556 58 68'
303	[nano_sae_project_number_fup]	BASEC research project number (Swissethics)	text, Required<br>Field Annotation: @DEFAULT="2020-02258"
304	[nano_sae_notif_number_fup]	Notification n° (Swissmedic)	text, Required<br>Field Annotation: @DEFAULT="2021DR1042"
305	[nano_sae_id_fup]	Section Header: *SAE information*<br>Inclusion ID	text, Required<br>Custom alignment: RH
306	[nano_sae_dob_fup]	Year of birth	text (integer), Required<br>Custom alignment: RH
307	[nano_sae_sex_fup]	Sex	radio, Required<br>1 Male<br>2 Female<br>Custom alignment: RH
308	[nano_sae_ethnicity_fup]	Ethnicity (Several possible)	checkbox, Required<br>1 nano_sae_ethnicity_fup___1 Caucasian<br>2 nano_sae_ethnicity_fup___2 Asian<br>3 nano_sae_ethnicity_fup___3 Black<br>4 nano_sae_ethnicity_fup___4 Carribean

			5 nano_sae_ethnicity_fup___5 Indian<br>6 nano_sae_ethnicity_fup___6 Arab<br>7 nano_sae_ethnicity_fup___7 Other
309	[nano_sae_ethnicity_specify_fup]<br>Show the field ONLY if: [nano_sae_ethnicity_fup(7)] = '1'	Specify	text, Required
310	[nano_sae_date_container_fup]	Date of onset {nano_sae_ae_date_fup} {nano_sae_date_unknown_fup}	descriptive
311	[nano_sae_ae_date_fup]	AE onset date	text (date_dmy)
312	[nano_sae_date_unknown_fup]	Date unknown	checkbox<br>1 nano_sae_date_unknown_fup___1 Date unkn[illegible]
313	[nano_sae_ae_awareness_fup]	AE awareness date	text (date_dmy), Required
314	[nano_sae_ae_evolution_fup]	Does this SAE form report the evolution of a previously reported AE ?	yesno, Required<br>1 Yes<br>0 No
315	[nano_sae_ae_number_fup]<br>Show the field ONLY if: [nano_sae_ae_evolution_fup] = "1"	Specify AE number	text, Required
316	[nano_sae_ae_description_fup]	Section Header: *General*<br>Describe the AE and the connection to project procedures (including relevant test/lab data)	notes, Required
317	[nano_sae_ae_solicited_fup]	Which type of AE is it ?	radio, Required<br>1 Solicited AE<br>2 Unsolicited AE related with an abnormal laboratory value<br>3 Unsolicited AE
318	[nano_sae_fda_toxicity_fup]<br>Show the field ONLY if: [nano_sae_ae_solicited_fup] = "1"	FDA toxicity grading scale term	dropdown, Required<br>1 Pain<br>2 Tenderness<br>3 Erythema/Redness<br>4 Induration/Swelling<br>5 Nausae/Vomiiting<br>6 Diarrhea<br>7 Headache<br>8 Fatigue<br>9 Myalgia<br>10 Fever
319	[nano_sae_fda_tox_grade_2_fup]<br>Show the field ONLY if:	AE grade according to adapted FDA toxicity grading scale	radio, Required<br>1 Grade 1<br>2 Grade 2

	[nano_sae_ae_solicited_fup] = "1"		3 Grade 3
320	[nano_sae_fda_tox_grade_fup]<br><br>Show the field ONLY if:<br>[nano_sae_ae_solicited_fup] = "1"		descriptive<br>(Attachment: naNO-COVID_FDA_adapted.pdf, Display format: Link)<br>Field Annotation: @HIDDEN-PDF
321	[nano_sae_labo_ctcae_term_fup]<br><br>Show the field ONLY if:<br>[nano_sae_ae_solicited_fup] = "2"	AE term	text, Required
322	[nano_sae_ctcae_grade_v6_fup]<br><br>Show the field ONLY if:<br>[nano_sae_ae_solicited_fup] = "2"	AE grade according to adapted CTCAE v5.0	radio, Required<br>1 Grade 1<br>2 Grade 2<br>3 Grade 3<br>4 Grade 4<br>5 Grade 5
323	[nano_sae_ctcae_grade_v7_fup]<br><br>Show the field ONLY if:<br>[nano_sae_ae_solicited_fup] = "2"		descriptive<br>(Attachment: naNO-COVID_CTCAE_adapted_lab.pdf, Display format: Link)<br>Field Annotation: @HIDDEN-PDF
324	[nano_sae_ctcae_grade_fup]<br><br>Show the field ONLY if:<br>[nano_sae_ae_solicited_fup] = "3"	CTCAE term for grading AE	text, Required<br>BIOPORTAL:CTCAE BIOPORTAL:CTCAE
325	[nano_sae_spec_ctcae_fup]<br><br>Show the field ONLY if:<br>[nano_sae_ae_solicited_fup] = "3"	If needed, please specify	text, Required
326	[nano_sae_ctcae_grade_v5_fup]<br><br>Show the field ONLY if:<br>[nano_sae_ae_solicited_fup] = "3"	AE grade according to CTCAE v5.0	radio, Required<br>1 Grade 1<br>2 Grade 2<br>3 Grade 3<br>4 Grade 4<br>5 Grade 5
327	[nano_sae_yes_no_fup]	Is this a serious adverse event ?	yesno, Required<br>1 Yes<br>0 No
328	[nano_sae_type_fup]<br><br>Show the field ONLY if:<br>[nano_sae_yes_no_fup] = "1"	Check SAE (seriousness definition)	radio, Required<br>a Death<br>b Life-threatening event<br>c Important medical event (i.e. jeopardises the patient or may require an intervention to prevent death or life threatening event)

			d: Persistent or significant disability or incapacity<br>e: Hospitalisation or prolongation of an existing hospitalisation<br>f: Congenital anomaly or birth defect (in offspring of the subject)
329	[nano_sae_interest_fup]	Is this an adverse event of special interest ?	yesno, Required<br>1: Yes<br>0: No
330	[nano_sae_aesi_def_fup]		descriptive<br>(Attachment: Definition of Adverse Event of Special Interest.pdf, Display format: Link)<br>Field Annotation: @HIDDEN-PDF
331	[nano_sae_unexpected_ae_fup]	Was this an unexpected AE ?	yesno, Required<br>1: Yes<br>0: No
332	[nano_sae_doses_fup]	Section Header: *IMP*<br>Were one or two doses administred ?	radio, Required<br>1: One<br>2: Two
333	[nano_sae_random_number_fup]	Randomisation number	text (integer), Required
334	[nano_sae_code_broken_fup]	Was the code broken ?	yesno, Required<br>1: Yes<br>0: No
335	[nano_sae_detail_fup]<br>Show the field ONLY if:<br>[nano_sae_code_broken_fup] = "1"	Please detail the reason for unblinding, the person who proceeded to unblinding and the date.	notes, Required
336	[nano_sae_imp_name_fup]	Name of IMP : PepGNP-Covid19 or vehicle-GNP	descriptive
337	[nano_sae_dose_fup]	Dose	radio, Required<br>1: Low dose (12.8 ug of gold nanoparticules +/- 2.5 nmol SARSCoV2 peptides)<br>2: High dose (38.3 ug of gold nanoparticules +/- 7.5 nmol SARSCoV2 peptides)
338	[nano_sae_route_fup]	Route : Intradermal injection	descriptive
339	[nano_sae_date_administred_fup]	Administration date	text (date_dmy), Required
340	[nano_sae_imp_name_2_fup]<br>Show the field ONLY if:<br>[nano_sae_doses_fup] = "2"	Name of IMP : PepGNP-Covid19 or vehicle-GNP	descriptive
341	[nano_sae_dose_2_fup]<br>Show the field ONLY if:<br>[nano_sae_doses_fup] = "2"	Dose	radio, Required<br>1: Low dose (12.8 ug of gold nanoparticules +/- 2.5 nmol SARSCoV2 peptides)

			2 High dose (38.3 ug of gold nanoparticules +/- 7.5 nmol SARSCoV2 peptides)
342	[nano_sae_route_2_fup]<br>Show the field ONLY if:<br>[nano_sae_doses_fup] = "2"	Route : Intradermal injection	descriptive
343	[nano_sae_date_administred_2_fup]<br>Show the field ONLY if:<br>[nano_sae_doses_fup] = "2"	Administration date	text (date_dmy), Required
344	[nano_sae_causality_fup]	Section Header: *AE Causality*<br>Causality of event to intervention	radio, Required<br>1 No relationship<br>2 Possible<br>3 Probable<br>4 Definite
345	[nano_sae_causality_def_fup]		descriptive<br>(Attachment: Guidelines for assessing the relationship of vaccine administration to an adverse event.pdf, Display format: Link)<br>Field Annotation: @HIDDEN-PDF
346	[nano_sae_table_fup]	Section Header: *Relevant medical history*<br>Disease / Intervention /Allergy Start date Ongoing Stop date {nano_sae_disease_fup} {nano_sae_date_disease_fup} {nano_sae_disease_ongoing_fup} {nano_sae_disease_stop_fup} {nano_sae_disease_1_fup} {nano_sae_date_disease_1_fup} {nano_sae_disease_ongoing_1_fup} {nano_sae_disease_stop_1_fup} {nano_sae_disease_2_fup} {nano_sae_date_disease_2_fup} {nano_sae_disease_ongoing_2_fup} {nano_sae_disease_stop_2_fup} {nano_sae_disease_3_fup} {nano_sae_date_disease_3_fup} {nano_sae_disease_ongoing_3_fup} {nano_sae_disease_stop_3_fup} {nano_sae_disease_4_fup} {nano_sae_date_disease_4_fup} {nano_sae_disease_ongoing_4_fup} {nano_sae_disease_stop_4_fup}	descriptive
347	[nano_sae_disease_fup]	Disease / Intervention /Allergy	text
348	[nano_sae_date_disease_fup]	Start date	text
349	[nano_sae_disease_ongoing_fup]	Ongoing ?	yesno<br>1 Yes<br>0 No
350	[nano_sae_disease_stop_fup]	Stop date	text

351	[nano_sae_disease_1_fup]	Disease / Intervention /Allergy	text
352	[nano_sae_date_disease_1_fup]	Start date	text
353	[nano_sae_disease_ongoing_1_fup]	Ongoing ?	yesno<br>1 Yes<br>0 No
354	[nano_sae_disease_stop_1_fup]	Stop date	text
355	[nano_sae_disease_2_fup]	Disease / Intervention /Allergy	text
356	[nano_sae_date_disease_2_fup]	Start date	text
357	[nano_sae_disease_ongoing_2_fup]	Ongoing ?	yesno<br>1 Yes<br>0 No
358	[nano_sae_disease_stop_2_fup]	Stop date	text
359	[nano_sae_disease_3_fup]	Disease / Intervention /Allergy	text
360	[nano_sae_date_disease_3_fup]	Start date	text
361	[nano_sae_disease_ongoing_3_fup]	Ongoing ?	yesno<br>1 Yes<br>0 No
362	[nano_sae_disease_stop_3_fup]	Stop date	text
363	[nano_sae_disease_4_fup]	Disease / Intervention /Allergy	text
364	[nano_sae_date_disease_4_fup]	Start date	text
365	[nano_sae_disease_ongoing_4_fup]	Ongoing ?	yesno<br>1 Yes<br>0 No
366	[nano_sae_disease_stop_4_fup]	Stop date	text
367	[nano_sae_drug_fup]	Section Header: *Concomitant medications*<br>Concomitant drug(s) (exclude those to treat event)	yesno, Required<br>1 Yes<br>0 No
368	[nano_sae_conc_count_fup]<br>Show the field ONLY if:<br>[nano_sae_drug_fup] ="1"	How many concomitant drug ?	dropdown, Required<br>1 1<br>2 2<br>3 3<br>4 4<br>5 5<br>6 6

369	[nano_sae_med_name_fup]<br>Show the field ONLY if:<br>[nano_sae_drug_fup] ="1" and [nano_sae_conc_count_fup] > 0	Medication Name (Commercial name)	text, Required
370	[nano_sae_med_name_inter_fup]<br>Show the field ONLY if:<br>[nano_sae_drug_fup] ="1" and [nano_sae_conc_count_fup] > 0	Medication Name (International nonproprietary name)	text, Required
371	[nano_sae_med_dose_fup]<br>Show the field ONLY if:<br>[nano_sae_drug_fup] ="1" and [nano_sae_conc_count_fup] > 0	Dose	text (number), Required
372	[nano_sae_med_dose_unit_fup]<br>Show the field ONLY if:<br>[nano_sae_drug_fup] ="1" and [nano_sae_conc_count_fup] > 0	Dose unit	radio, Required<br>1 Microgram<br>2 Miligram<br>3 Gram<br>4 Mililiter<br>5 Other (Specify)<br>6 Unknown
373	[nano_sae_med_dose_spec_fup]<br>Show the field ONLY if:<br>[nano_sae_med_dose_unit_fup] = "5"	Specify	text, Required
374	[nano_sae_med_dose_freq_fup]<br>Show the field ONLY if:<br>[nano_sae_drug_fup] ="1" and [nano_sae_conc_count_fup] > 0	Frequency	radio, Required<br>1 Daily<br>2 Twice daily<br>3 Three times a day<br>4 Four times a day<br>5 4-6 hours<br>6 6-8 hours<br>7 PRN (ad needed)<br>8 Other (specify)
375	[nano_sae_med_freq_spec_fup]<br>Show the field ONLY if:<br>[nano_sae_med_dose_freq_fup] = "8"	Specify	text, Required
376	[nano_sae_med_route_fup]<br>Show the field ONLY if:<br>[nano_sae_drug_fup] ="1" and [nano_sae_conc_count_fup] > 0	Route	radio, Required<br>1 Oral<br>2 Topical<br>3 Subcutaneous injection<br>4 Intravenous injection

			5 Intramuscular injection<br>6 Rectal<br>7 Nasal<br>8 Inhaled<br>9 Transdermal<br>10 Vaginal<br>11 Other (Specify)
377	[nano_sae_med_route_spec_fup]<br>Show the field ONLY if:<br>[nano_sae_med_route_fup] = "11"	Specify	text, Required
378	[nano_sae_med_use_fup]<br>Show the field ONLY if:<br>[nano_sae_drug_fup] ="1" and [nano_sae_conc_count_fup] > 0	Indication(s) for use	text, Required
379	[nano_sae_med_start_fup]<br>Show the field ONLY if:<br>[nano_sae_drug_fup] ="1" and [nano_sae_conc_count_fup] > 0	Start Date	text (date_dmy), Required
380	[nano_sae_med_ongoing_fup]<br>Show the field ONLY if:<br>[nano_sae_drug_fup] ="1" and [nano_sae_conc_count_fup] > 0	Ongoing	yesno, Required<br>1 Yes<br>0 No
381	[nano_sae_med_end_date_fup]<br>Show the field ONLY if:<br>[nano_sae_drug_fup] ="1" AND [nano_sae_med_ongoing_fup] = "0" and [nano_sae_conc_count_fup] > 0	End date	text (date_dmy), Required
382	[nano_sae_med_name_1_fup]<br>Show the field ONLY if:<br>[nano_sae_drug_fup] ="1" and [nano_sae_conc_count_fup] > 1	Medication Name (Commercial name)	text, Required
383	[nano_sae_med_name_inter_1_fup]<br>Show the field ONLY if:<br>[nano_sae_drug_fup] ="1" and [nano_sae_conc_count_fup] > 1	Medication Name (International nonproprietary name)	text, Required
384	[nano_sae_med_dose_1_fup]<br>Show the field ONLY if:	Dose	text (number, Min: 0, Max: 9999999), Required

	[nano_sae_drug_fup] ="1" and [nano_sae_conc_count_fup] > 1		
385	[nano_sae_med_dose_unit_1_fup]<br>Show the field ONLY if: [nano_sae_drug_fup] ="1" and [nano_sae_conc_count_fup] > 1	Dose unit	radio, Required<br>1 Microgram<br>2 Miligram<br>3 Gram<br>4 Mililiter<br>5 Other (Specify)<br>6 Unknown
386	[nano_sae_med_dose_spec_1_fup]<br>Show the field ONLY if: [nano_sae_med_dose_unit_1_fup] = "5"	Specify	text, Required
387	[nano_sae_med_dose_freq_1_fup]<br>Show the field ONLY if: [nano_sae_drug_fup] ="1" and [nano_sae_conc_count_fup] > 1	Frequency	radio, Required<br>1 Daily<br>2 Twice daily<br>3 Three times a day<br>4 Four times a day<br>5 4-6 hours<br>6 6-8 hours<br>7 PRN (ad needed)<br>8 Other (specify)
388	[nano_sae_med_freq_spec_1_fup]<br>Show the field ONLY if: [nano_sae_med_dose_freq_1_fup] = "8"	Specify	text, Required
389	[nano_sae_med_route_1_fup]<br>Show the field ONLY if: [nano_sae_drug_fup] ="1" and [nano_sae_conc_count_fup] > 1	Route	radio, Required<br>1 Oral<br>2 Topical<br>3 Subcutaneous injection<br>4 Intravenous injection<br>5 Intramuscular injection<br>6 Rectal<br>7 Nasal<br>8 Inhaled<br>9 Transdermal<br>10 Vaginal<br>11 Other (Specify)
390	[nano_sae_med_route_spec_1_fup]<br>Show the field ONLY if: [nano_sae_med_route_1_fup] = "11"	Specify	text, Required

391	[nano_sae_med_use_1_fup]<br><br>Show the field ONLY if:<br>[nano_sae_drug_fup] ="1" and [nano_sae_conc_count_fup] > 1	Indication(s) for use	text, Required
392	[nano_sae_med_start_1_fup]<br><br>Show the field ONLY if:<br>[nano_sae_drug_fup] ="1" and [nano_sae_conc_count_fup] > 1	Start Date	text (date_dmy), Required
393	[nano_sae_med_ongoing_1_fup]<br><br>Show the field ONLY if:<br>[nano_sae_drug_fup] ="1" and [nano_sae_conc_count_fup] > 1	Ongoing	yesno, Required<br>1 Yes<br>0 No
394	[nano_sae_med_end_date_1_fup]<br><br>Show the field ONLY if:<br>[nano_sae_drug_fup] ="1" AND [nano_sae_med_ongoing_1_fup] = "0" and [nano_sae_conc_count_fup] > 1	End date	text (date_dmy), Required
395	[nano_sae_med_name_2_fup]<br><br>Show the field ONLY if:<br>[nano_sae_drug_fup] ="1" and [nano_sae_conc_count_fup] > 2	Medication Name (Commercial name)	text, Required
396	[nano_sae_med_name_inter_2_fup]<br><br>Show the field ONLY if:<br>[nano_sae_drug_fup] ="1" and [nano_sae_conc_count_fup] > 2	Medication Name (International nonproprietary name)	text, Required
397	[nano_sae_med_dose_2_fup]<br><br>Show the field ONLY if:<br>[nano_sae_drug_fup] ="1" and [nano_sae_conc_count_fup] > 2	Dose	text (integer, Min: 0, Max: 9999999), Required
398	[nano_sae_med_dose_unit_2_fup]<br><br>Show the field ONLY if:<br>[nano_sae_drug_fup] ="1" and [nano_sae_conc_count_fup] > 2	Dose unit	radio, Required<br>1 Microgram<br>2 Miligram<br>3 Gram<br>4 Mililiter<br>5 Other (Specify)<br>6 Unknown
399	[nano_sae_med_dose_spec_2_fup]	Specify	text, Required

	Show the field ONLY if: [nano_sae_med_dose_unit_2_fup] = "5"		
400	[nano_sae_med_dose_freq_2_fup] Show the field ONLY if: [nano_sae_drug_fup] ="1" and [nano_sae_conc_count_fup] > 2	Frequency	radio, Required<br>1 Daily<br>2 Twice daily<br>3 Three times a day<br>4 Four times a day<br>5 4-6 hours<br>6 6-8 hours<br>7 PRN (ad needed)<br>8 Other (specify)
401	[nano_sae_med_freq_spec_2_fup] Show the field ONLY if: [nano_sae_med_dose_freq_2_fup] = "8"	Specify	text, Required
402	[nano_sae_med_route_2_fup] Show the field ONLY if: [nano_sae_drug_fup] ="1" and [nano_sae_conc_count_fup] > 2	Route	radio, Required<br>1 Oral<br>2 Topical<br>3 Subcutaneous injection<br>4 Intravenous injection<br>5 Intramuscular injection<br>6 Rectal<br>7 Nasal<br>8 Inhaled<br>9 Transdermal<br>10 Vaginal<br>11 Other (Specify)
403	[nano_sae_med_route_spec_2_fup] Show the field ONLY if: [nano_sae_med_route_2_fup] = "11"	Specify	text, Required
404	[nano_sae_med_use_2_fup] Show the field ONLY if: [nano_sae_drug_fup] ="1" and [nano_sae_conc_count_fup] > 2	Indication(s) for use	text, Required
405	[nano_sae_med_start_2_fup] Show the field ONLY if: [nano_sae_drug_fup] ="1" and [nano_sae_conc_count_fup] > 2	Start Date	text (date_dmy), Required
406	[nano_sae_med_ongoing_2_fup] Show the field ONLY if:	Ongoing	yesno, Required<br>1 Yes

	[nano_sae_drug_fup] ="1" and [nano_sae_conc_count_fup] > 2		0 No
407	[nano_sae_med_end_date_2_fup]<br><br>Show the field ONLY if:<br>[nano_sae_drug_fup] ="1" AND [nano_sae_med_ongoing_2_fup] = "0" and [nano_sae_conc_count_fup] > 2	End date	text (date_dmy), Required
408	[nano_sae_med_name_3_fup]<br><br>Show the field ONLY if:<br>[nano_sae_drug_fup] ="1" and [nano_sae_conc_count_fup] > 3	Medication Name (Commercial name)	text, Required
409	[nano_sae_med_name_inter_3_fup]<br><br>Show the field ONLY if:<br>[nano_sae_drug_fup] ="1" and [nano_sae_conc_count_fup] > 3	Medication Name (International nonproprietary name)	text, Required
410	[nano_sae_med_dose_3_fup]<br><br>Show the field ONLY if:<br>[nano_sae_drug_fup] ="1" and [nano_sae_conc_count_fup] > 3	Dose	text (number, Min: 0, Max: 9999999), Required
411	[nano_sae_med_dose_unit_3_fup]<br><br>Show the field ONLY if:<br>[nano_sae_drug_fup] ="1" and [nano_sae_conc_count_fup] > 3	Dose unit	radio, Required<br>1 Microgram<br>2 Miligram<br>3 Gram<br>4 Mililiter<br>5 Other (Specify)<br>6 Unknown
412	[nano_sae_med_dose_spec_3_fup]<br><br>Show the field ONLY if:<br>[nano_sae_med_dose_unit_3_fup] = "5"	Specify	text, Required
413	[nano_sae_med_dose_freq_3_fup]<br><br>Show the field ONLY if:<br>[nano_sae_drug_fup] ="1" and [nano_sae_conc_count_fup] > 3	Frequency	radio, Required<br>1 Daily<br>2 Twice daily<br>3 Three times a day<br>4 Four times a day<br>5 4-6 hours<br>6 6-8 hours<br>7 PRN (ad needed)<br>8 Other (specify)

414	[nano_sae_med_freq_spec_3_fup]<br><br>Show the field ONLY if:<br>[nano_sae_med_dose_freq_3_fup] = "8"	Specify	text, Required
415	[nano_sae_med_route_3_fup]<br><br>Show the field ONLY if:<br>[nano_sae_drug_fup] ="1" and [nano_sae_conc_count_fup] > 3	Route	radio, Required<br>1 Oral<br>2 Topical<br>3 Subcutaneous injection<br>4 Intravenous injection<br>5 Intramuscular injection<br>6 Rectal<br>7 Nasal<br>8 Inhaled<br>9 Transdermal<br>10 Vaginal<br>11 Other (Specify)
416	[nano_sae_med_route_spec_3_fup]<br><br>Show the field ONLY if:<br>[nano_sae_med_route_3_fup] = "11"	Specify	text, Required
417	[nano_sae_med_use_3_fup]<br><br>Show the field ONLY if:<br>[nano_sae_drug_fup] ="1" and [nano_sae_conc_count_fup] > 3	Indication(s) for use	text, Required
418	[nano_sae_med_start_3_fup]<br><br>Show the field ONLY if:<br>[nano_sae_drug_fup] ="1" and [nano_sae_conc_count_fup] > 3	Start Date	text (date_dmy), Required
419	[nano_sae_med_ongoing_3_fup]<br><br>Show the field ONLY if:<br>[nano_sae_drug_fup] ="1" and [nano_sae_conc_count_fup] > 3	Ongoing	yesno, Required<br>1 Yes<br>0 No
420	[nano_sae_med_end_date_3_fup]<br><br>Show the field ONLY if:<br>[nano_sae_drug_fup] ="1" AND [nano_sae_med_ongoing_3_fup] = "0" and [nano_sae_conc_count_fup] > 3	End date	text (date_dmy), Required
421	[nano_sae_med_name_4_fup]<br><br>Show the field ONLY if:	Medication Name (Commercial name)	text, Required

	[nano_sae_drug_fup] ="1" and [nano_sae_conc_count_fup] > 4		
422	[nano_sae_med_name_inter_4_fup]<br><br>Show the field ONLY if:<br>[nano_sae_drug_fup] ="1" and [nano_sae_conc_count_fup] > 4	Medication Name (International nonproprietary name)	text, Required
423	[nano_sae_med_dose_4_fup]<br><br>Show the field ONLY if:<br>[nano_sae_drug_fup] ="1" and [nano_sae_conc_count_fup] > 4	Dose	text (number, Min: 0, Max: 9999999), Required
424	[nano_sae_med_dose_unit_4_fup]<br><br>Show the field ONLY if:<br>[nano_sae_drug_fup] ="1" and [nano_sae_conc_count_fup] > 4	Dose unit	radio, Required<br>1 Microgram<br>2 Miligram<br>3 Gram<br>4 Mililiter<br>5 Other (Specify)<br>6 Unknown
425	[nano_sae_med_dose_spec_4_fup]<br><br>Show the field ONLY if:<br>[nano_sae_med_dose_unit_4_fup] = "5"	Specify	text, Required
426	[nano_sae_med_dose_freq_4_fup]<br><br>Show the field ONLY if:<br>[nano_sae_drug_fup] ="1" and [nano_sae_conc_count_fup] > 4	Frequency	radio, Required<br>1 Daily<br>2 Twice daily<br>3 Three times a day<br>4 Four times a day<br>5 4-6 hours<br>6 6-8 hours<br>7 PRN (ad needed)<br>8 Other (specify)
427	[nano_sae_med_freq_spec_4_fup]<br><br>Show the field ONLY if:<br>[nano_sae_med_dose_freq_4_fup] = "8"	Specify	text, Required
428	[nano_sae_med_route_4_fup]<br><br>Show the field ONLY if:<br>[nano_sae_drug_fup] ="1" and [nano_sae_conc_count_fup] > 4	Route	radio, Required<br>1 Oral<br>2 Topical<br>3 Subcutaneous injection<br>4 Intravenous injection<br>5 Intramuscular injection<br>6 Rectal

			7 Nasal<br>8 Inhaled<br>9 Transdermal<br>10 Vaginal<br>11 Other (Specify)
429	[nano_sae_med_route_spec_4_fup]<br>Show the field ONLY if:<br>[nano_sae_med_route_4_fup] = "11"	Specify	text, Required
430	[nano_sae_med_use_4_fup]<br>Show the field ONLY if:<br>[nano_sae_drug_fup] ="1" and [nano_sae_conc_count_fup] > 4	Indication(s) for use	text, Required
431	[nano_sae_med_start_4_fup]<br>Show the field ONLY if:<br>[nano_sae_drug_fup] ="1" and [nano_sae_conc_count_fup] > 4	Start Date	text (date_dmy), Required
432	[nano_sae_med_ongoing_4_fup]<br>Show the field ONLY if:<br>[nano_sae_drug_fup] ="1" and [nano_sae_conc_count_fup] > 4	Ongoing	yesno, Required<br>1 Yes<br>0 No
433	[nano_sae_med_end_date_4_fup]<br>Show the field ONLY if:<br>[nano_sae_drug_fup] ="1" AND [nano_sae_med_ongoing_4_fup] = "0" and [nano_sae_conc_count_fup] > 4	End date	text (date_dmy), Required
434	[nano_sae_med_name_5_fup]<br>Show the field ONLY if:<br>[nano_sae_drug_fup] ="1" and [nano_sae_conc_count_fup] > 5	Medication Name (Commercial name)	text, Required
435	[nano_sae_med_name_inter_5_fup]<br>Show the field ONLY if:<br>[nano_sae_drug_fup] ="1" and [nano_sae_conc_count_fup] > 5	Medication Name (International nonproprietary name)	text, Required
436	[nano_sae_med_dose_5_fup]<br>Show the field ONLY if:<br>[nano_sae_drug_fup] ="1" and [nano_sae_conc_count_fup] > 5	Dose	text (number, Min: 0, Max: 9999999), Required

437	[nano_sae_med_dose_unit_5_fup]<br>Show the field ONLY if:<br>[nano_sae_drug_fup] ="1" and [nano_sae_conc_count_fup] > 5	Dose unit	radio, Required<br>1 Microgram<br>2 Miligram<br>3 Gram<br>4 Mililiter<br>5 Other (Specify)<br>6 Unknown
438	[nano_sae_med_dose_spec_5_fup]<br>Show the field ONLY if:<br>[nano_sae_med_dose_unit_5_fup] = "5"	Specify	text, Required
439	[nano_sae_med_dose_freq_5_fup]<br>Show the field ONLY if:<br>[nano_sae_drug_fup] ="1" and [nano_sae_conc_count_fup] > 5	Frequency	radio, Required<br>1 Daily<br>2 Twice daily<br>3 Three times a day<br>4 Four times a day<br>5 4-6 hours<br>6 6-8 hours<br>7 PRN (ad needed)<br>8 Other (specify)
440	[nano_sae_med_freq_spec_5_fup]<br>Show the field ONLY if:<br>[nano_sae_med_dose_freq_5_fup] = "8"	Specify	text, Required
441	[nano_sae_med_route_5_fup]<br>Show the field ONLY if:<br>[nano_sae_drug_fup] ="1" and [nano_sae_conc_count_fup] > 5	Route	radio, Required<br>1 Oral<br>2 Topical<br>3 Subcutaneous injection<br>4 Intravenous injection<br>5 Intramuscular injection<br>6 Rectal<br>7 Nasal<br>8 Inhaled<br>9 Transdermal<br>10 Vaginal<br>11 Other (Specify)
442	[nano_sae_med_route_spec_5_fup]<br>Show the field ONLY if:<br>[nano_sae_med_route_5_fup] = "11"	Specify	text, Required
443	[nano_sae_med_use_5_fup]<br>Show the field ONLY if:	Indication(s) for use	text, Required

	[nano_sae_drug_fup] ="1" and [nano_sae_conc_count_fup] > 5		
444	[nano_sae_med_start_5_fup]<br>Show the field ONLY if: [nano_sae_drug_fup] ="1" and [nano_sae_conc_count_fup] > 5	Start Date	text (date_dmy), Required
445	[nano_sae_med_ongoing_5_fup]<br>Show the field ONLY if: [nano_sae_drug_fup] ="1" and [nano_sae_conc_count_fup] > 5	Ongoing	yesno, Required<br>1 Yes<br>0 No
446	[nano_sae_med_end_date_5_fup]<br>Show the field ONLY if: [nano_sae_drug_fup] ="1" AND [nano_sae_med_ongoing_5_fup] = "0" and [nano_sae_conc_count_fup] > 5	End date	text (date_dmy), Required
447	[nano_sae_test_performed_fup]	Section Header: *Relevant paraclinic tests*<br>Have some paraclinical tests/procedures been performed ?	radio, Required<br>1 Yes<br>2 No<br>3 Not known
448	[nano_sae_lab_tests_fup]<br>Show the field ONLY if: [nano_sae_test_performed_fup] = "1"	Laboratory tests	radio, Required<br>1 Yes<br>2 No<br>3 Not known
449	[nano_sae_radio_tests_fup]<br>Show the field ONLY if: [nano_sae_test_performed_fup] = "1"	Radiological exams	radio, Required<br>1 Yes<br>2 No<br>3 Not known
450	[nano_sae_relevant_proced_fup]<br>Show the field ONLY if: [nano_sae_test_performed_fup] = "1"	Other relevant diagnostic procedure(s)*	radio, Required<br>1 Yes<br>2 No<br>3 Not known
451	[nano_sae_specify_fup]<br>Show the field ONLY if: [nano_sae_relevant_proced_fup] = "1"	Specify	text, Required
452	[nano_sae_main_result_fup]<br>Show the field ONLY if: [nano_sae_test_performed_fup] = "1"	Please provide the main results of the exams performed	notes, Required
453	[nano_sae_text_fup]	Do not forget to collect complementary exam, remove identifying data, code them, register them	descriptive<br>Field Annotation: @HIDDEN-PDF

	Show the field ONLY if: [nano_sae_relevant_proced_fup] = "1" OR [nano_sae_radio_tests_fup] = "1" OR [nano_sae_lab_tests_fup] = "1"	as source documents and send them to the PI in a separate email.	
454	[nano_sae_action_taken_imp_fup]	Section Header: *Actions taken and outcome*<br>Which action(s) was/were taken with IMP ? (Several possible)	checkbox, Required<br>a | nano_sae_action_taken_imp_fup___a | No<br>b | nano_sae_action_taken_imp_fup___b | Do cha<br>c | nano_sae_action_taken_imp_fup___c | Do rec<br>d | nano_sae_action_taken_imp_fup___d | Do inc<br>e | nano_sae_action_taken_imp_fup___e | Dru ten inte<br>f | nano_sae_action_taken_imp_fup___f | Dru wit
455	[nano_sae_action_taken_imp_sp_fup]<br>Show the field ONLY if: [nano_sae_action_taken_imp_fup(c)] = '1' or [nano_sae_action_taken_imp_fup(d)] = '1' or [nano_sae_action_taken_imp_fup(e)] = '1'	Specify	text, Required
456	[nano_sae_action_taken_fup]	Which action(s) was/were taken with the participant ? (Several possible)	checkbox, Required<br>f | nano_sae_action_taken_fup___f | No actic<br>g | nano_sae_action_taken_fup___g | Medicat treatme<br>h | nano_sae_action_taken_fup___h | Non medicat treatme
457	[nano_sae_action_taken_spec_fup]<br>Show the field ONLY if: [nano_sae_action_taken_fup(h)] = '1'	Specify	text, Required
458	[nano_sae_count_fup]<br>Show the field ONLY if: [nano_sae_action_taken_fup(g)] = '1'	How many medication ?	dropdown, Required<br>1 | 1<br>2 | 2<br>3 | 3<br>4 | 4<br>5 | 5<br>6 | 6<br>7 | 7<br>8 | 8<br>9 | 9
459	[nano_sae_action_med_name_fup]	Medication Name (Commercial name)	text, Required

	Show the field ONLY if: [nano_sae_action_taken_fup(g)] and [nano_sae_count_fup] > 0		
460	[nano_sae_action_med_name_inter_fup] Show the field ONLY if: [nano_sae_action_taken_fup(g)] and [nano_sae_count_fup] > 0	Medication Name (International nonproprietary name)	text, Required
461	[nano_sae_action_med_dose_fup] Show the field ONLY if: [nano_sae_action_taken_fup(g)] and [nano_sae_count_fup] > 0	Dose	text (number, Min: 0, Max: 9999999), Required
462	[nano_sae_action_med_unit_fup] Show the field ONLY if: [nano_sae_action_taken_fup(g)] and [nano_sae_count_fup] > 0	Dose unit	radio, Required<br>1 Microgram<br>2 Miligram<br>3 Gram<br>4 Mililiter<br>5 Other (Specify)<br>6 Unknown
463	[nano_sae_action_med_unit_spec_fup] Show the field ONLY if: [nano_sae_action_med_unit_fup] = "5"	Specify	text, Required
464	[nano_sae_action_med_freq_fup] Show the field ONLY if: [nano_sae_action_taken_fup(g)] and [nano_sae_count_fup] > 0	Frequency	radio, Required<br>1 Daily<br>2 Twice daily<br>3 Three times a day<br>4 Four times a day<br>5 4-6 hours<br>6 6-8 hours<br>7 PRN (ad needed)<br>8 Other (specify)
465	[nano_sae_action_med_freq_spec_fup] Show the field ONLY if: [nano_sae_action_med_freq_fup] = "8"	Specify	text, Required
466	[nano_sae_action_med_route_fup] Show the field ONLY if: [nano_sae_action_taken_fup(g)] and [nano_sae_count_fup] > 0	Route	radio, Required<br>1 Oral<br>2 Topical<br>3 Subcutaneous injection<br>4 Intravenous injection<br>5 Intramuscular injection

			6 Rectal<br>7 Nasal<br>8 Inhaled<br>9 Transdermal<br>10 Vaginal<br>11 Other (Specify)
467	[nano_sae_action_med_route_spec_fup]<br>Show the field ONLY if:<br>[nano_sae_action_med_route_fup] = "11"	Specify	text, Required
468	[nano_sae_action_med_date_fup]<br>Show the field ONLY if:<br>[nano_sae_action_taken_fup(g)] and [nano_sae_count_fup] > 0	Start Date	text (date_dmy), Required
469	[nano_sae_action_med_ongoing_fup]<br>Show the field ONLY if:<br>[nano_sae_action_taken_fup(g)] and [nano_sae_count_fup] > 0	Ongoing	yesno, Required<br>1 Yes<br>0 No
470	[nano_sae_action_med_end_fup]<br>Show the field ONLY if:<br>[nano_sae_action_taken_fup(g)] and [nano_sae_count_fup] > 0 and [nano_sae_action_med_ongoing_fup] = "0"	End date	text (date_dmy), Required
471	[nano_sae_action_med_name_1_fup]<br>Show the field ONLY if:<br>[nano_sae_action_taken_fup(g)] and [nano_sae_count_fup] > 1	Medication Name (Commercial name)	text, Required
472	[nano_sae_action_med_name_inter_1_fup]<br>Show the field ONLY if:<br>[nano_sae_action_taken_fup(g)] and [nano_sae_count_fup] > 1	Medication Name (International nonproprietary name)	text, Required
473	[nano_sae_action_med_dose_1_fup]<br>Show the field ONLY if:<br>[nano_sae_action_taken_fup(g)] and [nano_sae_count_fup] > 1	Dose	text (number, Min: 0, Max: 9999999), Required
474	[nano_sae_action_med_unit_1_fup]<br>Show the field ONLY if:	Dose unit	radio, Required<br>1 Microgram<br>2 Miligram

	[nano_sae_action_taken_fup(g)] and [nano_sae_count_fup] > 1		3 Gram<br>4 Mililiter<br>5 Other (Specify)<br>6 Unknown
475	[nano_sae_action_med_unit_spec_1_fup]<br>Show the field ONLY if:<br>[nano_sae_action_med_unit_1_fup] = "5"	Specify	text, Required
476	[nano_sae_action_med_freq_1_fup]<br>Show the field ONLY if:<br>[nano_sae_action_taken_fup(g)] and [nano_sae_count_fup] > 1	Frequency	radio, Required<br>1 Daily<br>2 Twice daily<br>3 Three times a day<br>4 Four times a day<br>5 4-6 hours<br>6 6-8 hours<br>7 PRN (ad needed)<br>8 Other (specify)
477	[nano_sae_med_action_freq_spec_1_fup]<br>Show the field ONLY if:<br>[nano_sae_action_med_freq_1_fup] = "8"	Specify	text, Required
478	[nano_sae_action_med_route_1_fup]<br>Show the field ONLY if:<br>[nano_sae_action_taken_fup(g)] and [nano_sae_count_fup] > 1	Route	radio, Required<br>1 Oral<br>2 Topical<br>3 Subcutaneous injection<br>4 Intravenous injection<br>5 Intramuscular injection<br>6 Rectal<br>7 Nasal<br>8 Inhaled<br>9 Transdermal<br>10 Vaginal<br>11 Other (Specify)
479	[nano_sae_med_action_route_spec_1_fup]<br>Show the field ONLY if:<br>[nano_sae_action_med_route_1_fup] = "11"	Specify	text, Required
480	[nano_sae_action_med_date_1_fup]<br>Show the field ONLY if:<br>[nano_sae_action_taken_fup(g)] and [nano_sae_count_fup] > 1	Start Date	text (date_dmy), Required

481	[nano_sae_action_med_ongoing_1_fup]<br>Show the field ONLY if:<br>[nano_sae_action_taken_fup(g)] and [nano_sae_count_fup] > 1	Ongoing	yesno, Required<br>1 Yes<br>0 No
482	[nano_sae_action_med_end_1_fup]<br>Show the field ONLY if:<br>[nano_sae_action_taken_fup(g)] and [nano_sae_count_fup] > 1 and [nano_sae_action_med_ongoing_1_fup] = "0"	End date	text (date_dmy), Required
483	[nano_sae_action_med_name_2_fup]<br>Show the field ONLY if:<br>[nano_sae_action_taken_fup(g)] and [nano_sae_count_fup] > 2	Medication Name (Commercial name)	text, Required
484	[nano_sae_action_med_name_inter_2_fup]<br>Show the field ONLY if:<br>[nano_sae_action_taken_fup(g)] and [nano_sae_count_fup] > 2	Medication Name (International nonproprietary name)	text, Required
485	[nano_sae_action_med_dose_2_fup]<br>Show the field ONLY if:<br>[nano_sae_action_taken_fup(g)] and [nano_sae_count_fup] > 2	Dose	text (number, Min: 0, Max: 9999999), Required
486	[nano_sae_action_med_unit_2_fup]<br>Show the field ONLY if:<br>[nano_sae_action_taken_fup(g)] and [nano_sae_count_fup] > 2	Dose unit	radio, Required<br>1 Microgram<br>2 Miligram<br>3 Gram<br>4 Mililiter<br>5 Other (Specify)<br>6 Unknown
487	[nano_sae_action_med_unit_spec_2_fup]<br>Show the field ONLY if:<br>[nano_sae_action_med_unit_2_fup] = "5"	Specify	text, Required
488	[nano_sae_action_med_freq_2_fup]<br>Show the field ONLY if:<br>[nano_sae_action_taken_fup(g)] and [nano_sae_count_fup] > 2	Frequency	radio, Required<br>1 Daily<br>2 Twice daily<br>3 Three times a day<br>4 Four times a day<br>5 4-6 hours<br>6 6-8 hours

			7 PRN (ad needed)<br>8 Other (specify)
489	[nano_sae_med_action_freq_spec_2_fup]<br>Show the field ONLY if:<br>[nano_sae_action_med_freq_2_fup] = "8"	Specify	text, Required
490	[nano_sae_action_med_route_2_fup]<br>Show the field ONLY if:<br>[nano_sae_action_taken_fup(g)] and [nano_sae_count_fup] > 2	Route	radio, Required<br>1 Oral<br>2 Topical<br>3 Subcutaneous injection<br>4 Intravenous injection<br>5 Intramuscular injection<br>6 Rectal<br>7 Nasal<br>8 Inhaled<br>9 Transdermal<br>10 Vaginal<br>11 Other (Specify)
491	[nano_sae_med_action_route_spec_2_fup]<br>Show the field ONLY if:<br>[nano_sae_action_med_route_2_fup] = "11"	Specify	text, Required
492	[nano_sae_action_med_date_2_fup]<br>Show the field ONLY if:<br>[nano_sae_action_taken_fup(g)] and [nano_sae_count_fup] > 2	Start Date	text (date_dmy), Required
493	[nano_sae_action_med_ongoing_2_fup]<br>Show the field ONLY if:<br>[nano_sae_action_taken_fup(g)] and [nano_sae_count_fup] > 2	Ongoing	yesno, Required<br>1 Yes<br>0 No
494	[nano_sae_action_med_end_2_fup]<br>Show the field ONLY if:<br>[nano_sae_action_taken_fup(g)] and [nano_sae_count_fup] > 2 and [nano_sae_action_med_ongoing_2_fup] = "0"	End date	text (date_dmy), Required
495	[nano_sae_action_med_name_3_fup]<br>Show the field ONLY if:<br>[nano_sae_action_taken_fup(g)] and [nano_sae_count_fup] > 3	Medication Name (Commercial name)	text, Required

496	[nano_sae_action_med_name_inter_3_fup]<br>Show the field ONLY if:<br>[nano_sae_action_taken_fup(g)] and [nano_sae_count_fup] > 3	Medication Name (International nonproprietary name)	text, Required
497	[nano_sae_action_med_dose_3_fup]<br>Show the field ONLY if:<br>[nano_sae_action_taken_fup(g)] and [nano_sae_count_fup] > 3	Dose	text (number, Min: 0, Max: 9999999), Required
498	[nano_sae_action_med_unit_3_fup]<br>Show the field ONLY if:<br>[nano_sae_action_taken_fup(g)] and [nano_sae_count_fup] > 3	Dose unit	radio, Required<br>1 Microgram<br>2 Miligram<br>3 Gram<br>4 Mililiter<br>5 Other (Specify)<br>6 Unknown
499	[nano_sae_action_med_unit_spec_3_fup]<br>Show the field ONLY if:<br>[nano_sae_action_med_unit_3_fup] = "5"	Specify	text, Required
500	[nano_sae_action_med_freq_3_fup]<br>Show the field ONLY if:<br>[nano_sae_action_taken_fup(g)] and [nano_sae_count_fup] > 3	Frequency	radio, Required<br>1 Daily<br>2 Twice daily<br>3 Three times a day<br>4 Four times a day<br>5 4-6 hours<br>6 6-8 hours<br>7 PRN (ad needed)<br>8 Other (specify)
501	[nano_sae_med_action_freq_spec_3_fup]<br>Show the field ONLY if:<br>[nano_sae_action_med_freq_3_fup] = "8"	Specify	text, Required
502	[nano_sae_action_med_route_3_fup]<br>Show the field ONLY if:<br>[nano_sae_action_taken_fup(g)] and [nano_sae_count_fup] > 3	Route	radio, Required<br>1 Oral<br>2 Topical<br>3 Subcutaneous injection<br>4 Intravenous injection<br>5 Intramuscular injection<br>6 Rectal<br>7 Nasal<br>8 Inhaled<br>9 Transdermal

			10 Vaginal<br>11 Other (Specify)
503	[nano_sae_med_action_route_spec_3_fup]<br>Show the field ONLY if:<br>[nano_sae_action_med_route_3_fup] = "11"	Specify	text, Required
504	[nano_sae_action_med_date_3_fup]<br>Show the field ONLY if:<br>[nano_sae_action_taken_fup(g)] and [nano_sae_count_fup] > 3	Start Date	text (date_dmy), Required
505	[nano_sae_action_med_ongoing_3_fup]<br>Show the field ONLY if:<br>[nano_sae_action_taken_fup(g)] and [nano_sae_count_fup] > 3	Ongoing	yesno, Required<br>1 Yes<br>0 No
506	[nano_sae_action_med_end_3_fup]<br>Show the field ONLY if:<br>[nano_sae_action_taken_fup(g)] and [nano_sae_count_fup] > 3 and [nano_sae_action_med_ongoing_3_fup] = "0"	End date	text (date_dmy), Required
507	[nano_sae_action_med_name_4_fup]<br>Show the field ONLY if:<br>[nano_sae_action_taken_fup(g)] and [nano_sae_count_fup] > 4	Medication Name (Commercial name)	text, Required
508	[nano_sae_action_med_name_inter_4_fup]<br>Show the field ONLY if:<br>[nano_sae_action_taken_fup(g)] and [nano_sae_count_fup] > 4	Medication Name (International nonproprietary name)	text, Required
509	[nano_sae_action_med_dose_4_fup]<br>Show the field ONLY if:<br>[nano_sae_action_taken_fup(g)] and [nano_sae_count_fup] > 4	Dose	text (number, Min: 0, Max: 9999999), Required
510	[nano_sae_action_med_unit_4_fup]<br>Show the field ONLY if:<br>[nano_sae_action_taken_fup(g)] and [nano_sae_count_fup] > 4	Dose unit	radio, Required<br>1 Microgram<br>2 Miligram<br>3 Gram<br>4 Mililiter<br>5 Other (Specify)<br>6 Unknown

511	[nano_sae_action_med_unit_spec_4_fup]<br><br>Show the field ONLY if:<br>[nano_sae_action_med_unit_4_fup] = "5"	Specify	text, Required
512	[nano_sae_action_med_freq_4_fup]<br><br>Show the field ONLY if:<br>[nano_sae_action_taken_fup(g)] and [nano_sae_count_fup] > 4	Frequency	radio, Required<br>1 Daily<br>2 Twice daily<br>3 Three times a day<br>4 Four times a day<br>5 4-6 hours<br>6 6-8 hours<br>7 PRN (ad needed)<br>8 Other (specify)
513	[nano_sae_med_action_freq_spec_4_fup]<br><br>Show the field ONLY if:<br>[nano_sae_action_med_freq_4_fup] = "8"	Specify	text, Required
514	[nano_sae_action_med_route_4_fup]<br><br>Show the field ONLY if:<br>[nano_sae_action_taken_fup(g)] and [nano_sae_count_fup] > 4	Route	radio, Required<br>1 Oral<br>2 Topical<br>3 Subcutaneous injection<br>4 Intravenous injection<br>5 Intramuscular injection<br>6 Rectal<br>7 Nasal<br>8 Inhaled<br>9 Transdermal<br>10 Vaginal<br>11 Other (Specify)
515	[nano_sae_med_action_route_spec_4_fup]<br><br>Show the field ONLY if:<br>[nano_sae_action_med_route_4_fup] = "11"	Specify	text, Required
516	[nano_sae_action_med_date_4_fup]<br><br>Show the field ONLY if:<br>[nano_sae_action_taken_fup(g)] and [nano_sae_count_fup] > 4	Start Date	text (date_dmy), Required
517	[nano_sae_action_med_ongoing_4_fup]<br><br>Show the field ONLY if:<br>[nano_sae_action_taken_fup(g)] and [nano_sae_count_fup] > 4	Ongoing	yesno, Required<br>1 Yes<br>0 No

518	[nano_sae_action_med_end_4_fup]<br>Show the field ONLY if:<br>[nano_sae_action_taken_fup(g)] and [nano_sae_count_fup] > 4 and [nano_sae_action_med_ongoing_4_fup] = "0"	End date	text (date_dmy), Required
519	[nano_sae_action_med_name_5_fup]<br>Show the field ONLY if:<br>[nano_sae_action_taken_fup(g)] and [nano_sae_count_fup] > 5	Medication Name (Commercial name)	text, Required
520	[nano_sae_action_med_name_inter_5_fup]<br>Show the field ONLY if:<br>[nano_sae_action_taken_fup(g)] and [nano_sae_count_fup] > 5	Medication Name (International nonproprietary name)	text, Required
521	[nano_sae_action_med_dose_5_fup]<br>Show the field ONLY if:<br>[nano_sae_action_taken_fup(g)] and [nano_sae_count_fup] > 5	Dose	text (number, Min: 0, Max: 9999999), Required
522	[nano_sae_action_med_unit_5_fup]<br>Show the field ONLY if:<br>[nano_sae_action_taken_fup(g)] and [nano_sae_count_fup] > 5	Dose unit	radio, Required<br>1 Microgram<br>2 Miligram<br>3 Gram<br>4 Mililiter<br>5 Other (Specify)<br>6 Unknown
523	[nano_sae_action_med_unit_spec_5_fup]<br>Show the field ONLY if:<br>[nano_sae_action_med_unit_5_fup] = "5"	Specify	text, Required
524	[nano_sae_action_med_freq_5_fup]<br>Show the field ONLY if:<br>[nano_sae_action_taken_fup(g)] and [nano_sae_count_fup] > 5	Frequency	radio, Required<br>1 Daily<br>2 Twice daily<br>3 Three times a day<br>4 Four times a day<br>5 4-6 hours<br>6 6-8 hours<br>7 PRN (ad needed)<br>8 Other (specify)
525	[nano_sae_med_action_freq_spec_5_fup]<br>Show the field ONLY if:	Specify	text, Required

	[nano_sae_action_med_freq_5_fup] = "8"		
526	[nano_sae_action_med_route_5_fup]<br><br>Show the field ONLY if:<br>[nano_sae_action_taken_fup(g)] and [nano_sae_count_fup] > 5	Route	radio, Required<br>1 Oral<br>2 Topical<br>3 Subcutaneous injection<br>4 Intravenous injection<br>5 Intramuscular injection<br>6 Rectal<br>7 Nasal<br>8 Inhaled<br>9 Transdermal<br>10 Vaginal<br>11 Other (Specify)
527	[nano_sae_med_action_route_spec_5_fup]<br><br>Show the field ONLY if:<br>[nano_sae_action_med_route_5_fup] = "11"	Specify	text, Required
528	[nano_sae_action_med_date_5_fup]<br><br>Show the field ONLY if:<br>[nano_sae_action_taken_fup(g)] and [nano_sae_count_fup] > 5	Start Date	text (date_dmy), Required
529	[nano_sae_action_med_ongoing_5_fup]<br><br>Show the field ONLY if:<br>[nano_sae_action_taken_fup(g)] and [nano_sae_count_fup] > 5	Ongoing	yesno, Required<br>1 Yes<br>0 No
530	[nano_sae_action_med_end_5_fup]<br><br>Show the field ONLY if:<br>[nano_sae_action_taken_fup(g)] and [nano_sae_count_fup] > 5 and [nano_sae_action_med_ongoing_5_fup] = "0"	End date	text (date_dmy), Required
531	[nano_sae_action_med_name_6_fup]<br><br>Show the field ONLY if:<br>[nano_sae_action_taken_fup(g)] and [nano_sae_count_fup] > 6	Medication Name (Commercial name)	text, Required
532	[nano_sae_action_med_name_inter_6_fup]<br><br>Show the field ONLY if:<br>[nano_sae_action_taken_fup(g)] and [nano_sae_count_fup] > 6	Medication Name (International nonproprietary name)	text, Required

533	[nano_sae_action_med_dose_6_fup]<br><br>Show the field ONLY if:<br>[nano_sae_action_taken_fup(g)] and [nano_sae_count_fup] > 6	Dose	text (number, Min: 0, Max: 9999999), Required
534	[nano_sae_action_med_unit_6_fup]<br><br>Show the field ONLY if:<br>[nano_sae_action_taken_fup(g)] and [nano_sae_count_fup] > 6	Dose unit	radio, Required<br>1 Microgram<br>2 Miligram<br>3 Gram<br>4 Mililiter<br>5 Other (Specify)<br>6 Unknown
535	[nano_sae_action_med_unit_spec_6_fup]<br><br>Show the field ONLY if:<br>[nano_sae_action_med_unit_6_fup] = "5"	Specify	text, Required
536	[nano_sae_action_med_freq_6_fup]<br><br>Show the field ONLY if:<br>[nano_sae_action_taken_fup(g)] and [nano_sae_count_fup] > 6	Frequency	radio, Required<br>1 Daily<br>2 Twice daily<br>3 Three times a day<br>4 Four times a day<br>5 4-6 hours<br>6 6-8 hours<br>7 PRN (ad needed)<br>8 Other (specify)
537	[nano_sae_med_action_freq_spec_6_fup]<br><br>Show the field ONLY if:<br>[nano_sae_action_med_freq_6_fup] = "8"	Specify	text, Required
538	[nano_sae_action_med_route_6_fup]<br><br>Show the field ONLY if:<br>[nano_sae_action_taken_fup(g)] and [nano_sae_count_fup] > 6	Route	radio, Required<br>1 Oral<br>2 Topical<br>3 Subcutaneous injection<br>4 Intravenous injection<br>5 Intramuscular injection<br>6 Rectal<br>7 Nasal<br>8 Inhaled<br>9 Transdermal<br>10 Vaginal<br>11 Other (Specify)
539	[nano_sae_med_action_route_spec_6_fup]<br><br>Show the field ONLY if:	Specify	text, Required

	[nano_sae_action_med_route_6_fup] = "11"		
540	[nano_sae_action_med_date_6_fup]<br><br>Show the field ONLY if:<br>[nano_sae_action_taken_fup(g)] and [nano_sae_count_fup] > 6	Start Date	text (date_dmy), Required
541	[nano_sae_action_med_ongoing_6_fup]<br><br>Show the field ONLY if:<br>[nano_sae_action_taken_fup(g)] and [nano_sae_count_fup] > 6	Ongoing	yesno, Required<br>1 Yes<br>0 No
542	[nano_sae_action_med_end_6_fup]<br><br>Show the field ONLY if:<br>[nano_sae_action_taken_fup(g)] and [nano_sae_count_fup] > 6 and [nano_sae_action_med_ongoing_6_fup] = "0"	End date	text (date_dmy), Required
543	[nano_sae_action_med_name_7_fup]<br><br>Show the field ONLY if:<br>[nano_sae_action_taken_fup(g)] and [nano_sae_count_fup] > 7	Medication Name (Commercial name)	text, Required
544	[nano_sae_action_med_name_inter_7_fup]<br><br>Show the field ONLY if:<br>[nano_sae_action_taken_fup(g)] and [nano_sae_count_fup] > 7	Medication Name (International nonproprietary name)	text, Required
545	[nano_sae_action_med_dose_7_fup]<br><br>Show the field ONLY if:<br>[nano_sae_action_taken_fup(g)] and [nano_sae_count_fup] > 7	Dose	text (number, Min: 0, Max: 9999999), Required
546	[nano_sae_action_med_unit_7_fup]<br><br>Show the field ONLY if:<br>[nano_sae_action_taken_fup(g)] and [nano_sae_count_fup] > 7	Dose unit	radio, Required<br>1 Microgram<br>2 Miligram<br>3 Gram<br>4 Mililiter<br>5 Other (Specify)<br>6 Unknown
547	[nano_sae_action_med_unit_spec_7_fup]<br><br>Show the field ONLY if:<br>[nano_sae_action_med_unit_7_fup] = "5"	Specify	text, Required

548	[nano_sae_action_med_freq_7_fup]<br>Show the field ONLY if:<br>[nano_sae_action_taken_fup(g)] and [nano_sae_count_fup] > 7	Frequency	radio, Required<br>1 Daily<br>2 Twice daily<br>3 Three times a day<br>4 Four times a day<br>5 4-6 hours<br>6 6-8 hours<br>7 PRN (ad needed)<br>8 Other (specify)
549	[nano_sae_med_action_freq_spec_7_fup]<br>Show the field ONLY if:<br>[nano_sae_action_med_freq_7_fup] = "8"	Specify	text, Required
550	[nano_sae_action_med_route_7_fup]<br>Show the field ONLY if:<br>[nano_sae_action_taken_fup(g)] and [nano_sae_count_fup] > 7	Route	radio, Required<br>1 Oral<br>2 Topical<br>3 Subcutaneous injection<br>4 Intravenous injection<br>5 Intramuscular injection<br>6 Rectal<br>7 Nasal<br>8 Inhaled<br>9 Transdermal<br>10 Vaginal<br>11 Other (Specify)
551	[nano_sae_med_action_route_spec_7_fup]<br>Show the field ONLY if:<br>[nano_sae_action_med_route_7_fup] = "11"	Specify	text, Required
552	[nano_sae_action_med_date_7_fup]<br>Show the field ONLY if:<br>[nano_sae_action_taken_fup(g)] and [nano_sae_count_fup] > 7	Start Date	text (date_dmy), Required
553	[nano_sae_action_med_ongoing_7_fup]<br>Show the field ONLY if:<br>[nano_sae_action_taken_fup(g)] and [nano_sae_count_fup] > 7	Ongoing	yesno, Required<br>1 Yes<br>0 No
554	[nano_sae_action_med_end_7_fup]<br>Show the field ONLY if:<br>[nano_sae_action_taken_fup(g)] and [nano_sae_count_fup] > 7 and [nan	End date	text (date_dmy), Required

	o_sae_action_med_ongoing_7_fup] = "0"		
555	[nano_sae_action_med_name_8_fup]<br>Show the field ONLY if:<br>[nano_sae_action_taken_fup(g)] and [nano_sae_count_fup] > 8	Medication Name (Commercial name)	text, Required
556	[nano_sae_action_med_name_inter_8_fup]<br>Show the field ONLY if:<br>[nano_sae_action_taken_fup(g)] and [nano_sae_count_fup] > 8	Medication Name (International nonproprietary name)	text, Required
557	[nano_sae_action_med_dose_8_fup]<br>Show the field ONLY if:<br>[nano_sae_action_taken_fup(g)] and [nano_sae_count_fup] > 8	Dose	text (number, Min: 0, Max: 9999999), Required
558	[nano_sae_action_med_unit_8_fup]<br>Show the field ONLY if:<br>[nano_sae_action_taken_fup(g)] and [nano_sae_count_fup] > 8	Dose unit	radio, Required<br>1 Microgram<br>2 Miligram<br>3 Gram<br>4 Mililiter<br>5 Other (Specify)<br>6 Unknown
559	[nano_sae_action_med_unit_spec_8_fup]<br>Show the field ONLY if:<br>[nano_sae_action_med_unit_8_fup] = "5"	Specify	text, Required
560	[nano_sae_action_med_freq_8_fup]<br>Show the field ONLY if:<br>[nano_sae_action_taken_fup(g)] and [nano_sae_count_fup] > 8	Frequency	radio, Required<br>1 Daily<br>2 Twice daily<br>3 Three times a day<br>4 Four times a day<br>5 4-6 hours<br>6 6-8 hours<br>7 PRN (ad needed)<br>8 Other (specify)
561	[nano_sae_med_action_freq_spec_8_fup]<br>Show the field ONLY if:<br>[nano_sae_action_med_freq_8_fup] = "8"	Specify	text, Required
562	[nano_sae_action_med_route_8_fup]<br>Show the field ONLY if:	Route	radio, Required<br>1 Oral<br>2 Topical

	[nano_sae_action_taken_fup(g)] and [nano_sae_count_fup] > 8		3 Subcutaneous injection<br>4 Intravenous injection<br>5 Intramuscular injection<br>6 Rectal<br>7 Nasal<br>8 Inhaled<br>9 Transdermal<br>10 Vaginal<br>11 Other (Specify)
563	[nano_sae_med_action_route_spec_8_fup]<br>Show the field ONLY if:<br>[nano_sae_action_med_route_8_fup] = "11"	Specify	text, Required
564	[nano_sae_action_med_date_8_fup]<br>Show the field ONLY if:<br>[nano_sae_action_taken_fup(g)] and [nano_sae_count_fup] > 8	Start Date	text (date_dmy), Required
565	[nano_sae_action_med_ongoing_8_fup]<br>Show the field ONLY if:<br>[nano_sae_action_taken_fup(g)] and [nano_sae_count_fup] > 8	Ongoing	yesno, Required<br>1 Yes<br>0 No
566	[nano_sae_action_med_end_8_fup]<br>Show the field ONLY if:<br>[nano_sae_action_taken_fup(g)] and [nano_sae_count_fup] > 8 and [nano_sae_action_med_ongoing_8_fup] = "0"	End date	text (date_dmy), Required
567	[nano_sae_react_abate_fup]	Did the reaction abate after stopping the vaccination ?	radio, Required<br>1 Yes<br>2 No<br>3 Not applicable
568	[nano_sae_react_reappear_fup]	Did the reaction reappear after the second dose of the vaccination ?	radio, Required<br>1 Yes<br>2 No<br>3 Not applicable
569	[nano_sae_outcome_fup]	Issue of event	radio, Required<br>1 Resolved (for date, last day of symptoms)<br>2 Resolved with sequelae<br>3 Ongoing<br>4 Stabilisation<br>5 Improvement

			6 Agravation<br>7 Fatal<br>8 Unknown
570	[nano_sae_outcome_date_fup]<br>Show the field ONLY if:<br>[nano_sae_outcome_fup] = '2' or [nano_sae_outcome_fup] = '1' or [nano_sae_outcome_fup] = '7'	Date	text (date_dmy), Required
571	[nano_sae_outcome_spec_fup]<br>Show the field ONLY if:<br>[nano_sae_outcome_fup] = '8'	Specify	text, Required
572	[nano_sae_autopsy_fup]<br>Show the field ONLY if:<br>[nano_sae_outcome_fup] = '7'	Autopsy performed	radio, Required<br>1 Yes<br>2 No<br>3 Not known
573	[nano_sae_autopsy_report_fup]<br>Show the field ONLY if:<br>[nano_sae_autopsy_fup] = '1'	Autopsy report available	radio, Required<br>1 Yes<br>2 No<br>3 Not known
574	[nano_sae_text_2_fup]<br>Show the field ONLY if:<br>[nano_sae_autopsy_report_fup] = '1'	Do not forget to collect the report, remove identifying data, code it, register it as source documents and send it to the PI in a separate email.	descriptive<br>Field Annotation: @HIDDEN-PDF
575	[nano_sae_doc_sent_fup]	Source document sent ?	yesno, Required<br>1 Yes<br>0 No
576	[nano_sae_doc_spec_fup]<br>Show the field ONLY if:<br>[nano_sae_doc_sent_fup] = "1"	Specify	text, Required
577	[nano_sae_investigator_name_fup]	Section Header: *Investigator Signature*<br>Name of investigator	text, Required
578	[nano_sae_inv_sign_date_fup]	Date	text (date_dmy), Required
579	[nano_sae_investigator_sign_fup]	Signature of investigator	file (signature), Required
580	[nano_sae_send_report_fup]	Send the report to the PI for modification/validation	yesno, Required<br>1 Yes<br>0 No<br>Field Annotation: @HIDDEN-PDF
581	[nano_sae_approved_fup]	Section Header: *SAE report PI evaluation*<br>SAE report approved by PI	yesno, Required<br>1 Yes

			0 No<br>Field Annotation: @HIDE_BY_ROLE=PI
582	[nano_sae_add_comment_fup]	Additionnal comment	yesno, Required<br>1 Yes<br>0 No<br>Field Annotation: @HIDE_BY_ROLE=PI
583	[nano_sae_comment_specify_fup]<br>Show the field ONLY if:<br>[nano_sae_add_comment_fup] = "1" or [nano_sae_add_comment_fup] = "1"	Specify	text, Required<br>Field Annotation: @HIDE_BY_ROLE=PI
584	[nano_sae_pi_name_bottom_fup]	Name of PI	text, Required<br>Field Annotation: @HIDE_BY_ROLE=PI
585	[nano_sae_pi_sign_date_fup]	Date	text (date_dmy), Required<br>Field Annotation: @HIDE_BY_ROLE=PI
586	[nano_sae_sign_pi_fup]	Signature of PI	file (signature), Required<br>Field Annotation: @HIDE_BY_ROLE=PI
587	[nano_sae_text_3_fup]	All SAE and AESI must be reported immediately and within a maximum of 24 hours to the Sponsor	descriptive<br>Field Annotation: @HIDE_BY_ROLE=PI @HIDDEN-PDF
588	[nano_sae_pdf_fup]	Generated PDF that will be sent to sponsor	file, Required<br>Field Annotation: @HIDE_BY_ROLE=PI @HIDDEN-PDF @HIDDEN
589	[nano_sae_send_sponsor_fup]	Send the PDF report to the Sponsor	yesno, Required<br>1 Yes<br>0 No<br>Field Annotation: @HIDE_BY_ROLE=PI @HIDDEN-PDF
590	[serious_adverse_event_form_followup_complete]	Section Header: *Form Status*<br>Complete?	dropdown<br>0 Incomplete<br>1 Unverified<br>2 Complete
**Instrument: Major Protocol Deviation Form (major_protocol_deviation_form)**			
591	[nano_p_screening_id]	Section Header: *General informations*<br>Screening ID	text, Required
592	[nano_p_number]	Protocol deviation/violation number	text, Required
593	[nano_p_date]	Date of the event	text (date_dmy), Required
594	[nano_p_date_intern]	Date of its discovery by research personnel	text (date_dmy), Required
595	[nano_p_date_report]	Date of the report	text (date_dmy), Required
596	[nano_p_type]	Section Header: *Event details*<br>Deviation type	radio, Required<br>a Informed Consent<br>b Eligibility Criteria<br>c Study Procedures - Screening<br>d Study Procedures - Study visits

			e Study Procedures - Laboratory Tests<br>f Withdrawal/Discontinuation from study<br>g Storage conditions not met<br>h Incorrect study treatment /administration /dose<br>i Excluded medication or vaccine<br>j Randomisation<br>k Blinding procedures<br>l Safety reporting<br>m Other
597	[nano_p_deviation_cat]<br>Show the field ONLY if:<br>[nano_p_type] = "a"	Choose category of deviation	radio, Required<br>1 Informed Consent Form not signed prior to any study procedure
598	[nano_p_text_sponsor]<br>Show the field ONLY if:<br>[nano_p_deviation_cat] = '1'	Report to sponsor immediately.	descriptive
599	[nano_p_deviation_cat_1]<br>Show the field ONLY if:<br>[nano_p_type] = 'b'	Choose category of deviation	radio<br>a Eligibility criteria not met
600	[nano_p_text_cat_1]<br>Show the field ONLY if:<br>[nano_p_deviation_cat_1] = 'a'	Refer to section 7.1 in the protocol for detailed inclusion/exclusion criterias.	descriptive
601	[nano_p_deviation_cat_2]<br>Show the field ONLY if:<br>[nano_p_type] = "c"	Choose category of deviation	radio, Required<br>a Medical history not completed prior to vaccination<br>b Concomitant medications information not completed prior to vaccination<br>c One or more aspects of physical exam completely missed<br>d Urinary protein/blood/glucose completely missed<br>e Pregnancy test missed at screening and before vaccination
602	[nano_p_deviation_cat_d]<br>Show the field ONLY if:<br>[nano_p_type] = 'd'	Choose category of deviation	radio<br>a Vaccination study visits (visit 2 & 6) missed over and above the tolerance window<br>b Participant Lost to follow up (LTFU)<br>c Pregnancy Test missed at Vaccination 1 and/or 2<br>d Body temperature not taken/ not recorded prior to vaccination<br>e COVID PCR/rapid test not done prior to vaccinations

603	[nano_p_deviation_text_d2]<br>Show the field ONLY if:<br>[nano_p_deviation_cat_d] = 'c'	If uncertain continuous contraception was adhered to, follow up with participant to come back into clinic (or take test at home) to confirm not pregnant.	descriptive
604	[nano_p_deviation_cat_e]<br>Show the field ONLY if:<br>[nano_p_type] = 'e'	Choose category of deviation	radio<br>a All haematology tests completely missed<br>b All biochemistry tests completely missed
605	[nano_p_deviation_cat_f]<br>Show the field ONLY if:<br>[nano_p_type] = 'f'	Choose category of deviation	radio<br>a Participant not withdrawn/discontinued from study after developing withdrawal criteria
606	[nano_p_deviation_cat_3]<br>Show the field ONLY if:<br>[nano_p_type] = "g"	Choose category of deviation	radio, Required<br>a Storage of IMP and GNP at control less than -20°C<br>b Prepared syringe kept at room temperature >4 hours
607	[nano_p_deviation_cat_4]<br>Show the field ONLY if:<br>[nano_p_type] = "h"	Choose category of deviation	radio, Required<br>a Incorrect dose given to patient - first dose (D0)<br>b Incorrect dose given to patient - second dose (D21)<br>c Missed second dose<br>d Incorrect site or route of injection<br>e No monitoring after vaccination for adverse reactions
608	[nano_p_deviation_cat_i]<br>Show the field ONLY if:<br>[nano_p_type] = 'i'	Choose category of deviation	radio<br>a Vaccinations excluded (including vaccination against COVID) by the protocol received 28 days prior or after PepGNP-Covid 19 injection
609	[nano_p_deviation_text_i]<br>Show the field ONLY if:<br>[nano_p_deviation_cat_i] = 'a'	For influenza, this period is reduced to 14 days (2 weeks) preceding the IMP and 28 days afterward	descriptive
610	[nano_p_deviation_cat_j]<br>Show the field ONLY if:<br>[nano_p_type] = 'j'	Choose category of deviation	radio<br>a Randomisation not performed
611	[nano_p_deviation_cat_7]<br>Show the field ONLY if:<br>[nano_p_type] = "k"	Choose category of deviation	radio, Required<br>a Participant is inadvertently unblinded<br>b Investigator/ Nurse inadvertently unblinded
612	[nano_p_text]<br>Show the field ONLY if:<br>[nano_p_deviation_cat_7] = "a" OR [nano_p_deviation_cat_7] = "b"	Corrective action : continue to follow usual trial, report case in study safety and trial reports.	descriptive

613	[nano_p_deviation_cat_6]<br>Show the field ONLY if:<br>[nano_p_type] = "l"	Choose category of deviation	radio, Required<br>a SAE/AE not reported<br>b Safety assessment not done at all<br>c Reporting deadlines not followed
614	[nano_p_specify]<br>Show the field ONLY if:<br>[nano_p_type] = "m"	Specify	text, Required
615	[nano_p_description]	Describe the protocol deviation/violation	notes, Required
616	[nano_p_reason]	Please explain the reason for the deviation/violation:	notes, Required
617	[nano_p_result_ae]	Did the deviation result in adverse event ?	yesno, Required<br>1 Yes<br>0 No
618	[nano_p_continuation]	Did the participant continue the study ?	yesno, Required<br>1 Yes<br>0 No
619	[nano_p_deviation_step_taken]	Describe the steps taken to avoid reccurence of the deviation/violation	notes, Required
620	[nano_p_name_investigator]	Section Header: *Investigator signature*<br>Name of investigator	text, Required
621	[nano_p_date_2]	Date	text (datetime_dmy), Required
622	[nano_p_sign_2]	Signature of investigator	file (signature), Required
623	[nano_p_send_report]	Send the report to the PI and the Sponsor ?	yesno, Required<br>1 Yes<br>0 No
624	[nano_p_pdf]	Generated pdf	file, Required<br>Field Annotation: @HIDDEN @HIDDEN-PDF
625	[major_protocol_deviation_form_complete]	Section Header: *Form Status*<br>Complete?	dropdown<br>0 Incomplete<br>1 Unverified<br>2 Complete
**Instrument: Minor Protocol Deviation Form (minor_protocol_deviation_form)**			
626	[nano_mp_screening_id]	Section Header: *General informations*<br>Screening ID	text, Required
627	[nano_mp_number]	Protocol deviation number	text, Required
628	[nano_mp_date]	Date of the event	text (date_dmy), Required
629	[nano_mp_date_discover]	Date of its discovery by research personnel	text (date_dmy), Required
630	[nano_mp_date_report]	Date of the report	text (date_dmy), Required
631	[nano_mp_deviation]	Section Header: *Event details*<br>Deviation type	radio, Required<br>a Informed consent<br>b Study Procedures - Screening<br>c Study Procedures - Study visits<br>d Study Procedures - Laboratory Tests

			e: Incorrect study treatment /administration /dose<br>f: Data monitoring<br>i: Other
632	[nano_mp_deviation_cat]<br>Show the field ONLY if: [nano_mp_deviation] ="a"	Choose category of deviation	radio, Required<br>a: Incorrect version of Informed Consent signed<br>b: Other
633	[nano_mp_text]<br>Show the field ONLY if: [nano_mp_deviation_cat] = "a"	Corrective action: note in ISF / patient folder and ask patient to sign current version at next study visit	descriptive
634	[nano_mp_deviation_cat_2]<br>Show the field ONLY if: [nano_mp_deviation] ="b"	Choose category of deviation	radio, Required<br>a: Medical history incomplete<br>b: Concomitant medications information missed<br>c: One or more aspects of physical exam missed<br>d: Urinary protein/blood/glucose missed<br>e: Other
635	[nano_mp_text_2]<br>Show the field ONLY if: [nano_mp_deviation_cat_2] = "a" OR [nano_mp_deviation_cat_2] = "b" OR [nano_mp_deviation_cat_2] = "c" OR [nano_mp_deviation_cat_2] = "d"	Corrective action: complete/perform prior to vaccination. Major protocol deviation if not complete/perform at all.	descriptive
636	[nano_mp_deviation_cat_3]<br>Show the field ONLY if: [nano_mp_deviation] = "c"	Choose category of deviation	radio, Required<br>a: Study visits (excluding visit 2 & 6) missed over and above the tolerance window<br>b: Other
637	[nano_mp_deviation_cat_4]<br>Show the field ONLY if: [nano_mp_deviation] = "d"	Choose category of deviation	radio, Required<br>a: One or more haematology tests missed<br>b: One or more biochemistry tests missed<br>c: Samples for immunogenicity studies (PBMC / Serum) not taken<br>d: One or more secondary immunogenicity tests missed (Anti-SARS-CoV-2 IgG, CD8 and Activation Markers)<br>e: One or more exploratory immunogenicity missed (Ig assay and T & B Cell responses)<br>f: HLA Typing missed<br>g: Other

638	[nano_mp_text_5]<br>Show the field ONLY if:<br>[nano_mp_deviation_cat_4] = "a" OR [nano_mp_deviation_cat_4] = "b"	Corrective action: complete/perform prior to vaccination. Major protocol deviation if not complete/perform at all.	descriptive
639	[nano_mp_text_6]<br>Show the field ONLY if:<br>[nano_mp_deviation_cat_4] = "c" OR [nano_mp_deviation_cat_4] = "d" OR [nano_mp_deviation_cat_4] = "e"	Corrective action : to be recorded and follow up. Follow protocol provided instructions for next blood draw and testing.	descriptive
640	[nano_mp_deviation_cat_8]<br>Show the field ONLY if:<br>[nano_mp_deviation] = 'e'	Choose category of deviation	radio<br>a: < 60 min monitoring after vaccination for adverse reactions<br>b: Other
641	[nano_mp_text_10]<br>Show the field ONLY if:<br>[nano_mp_deviation_cat_8] = 'a'	Major if completely missed otherwise minor.	descriptive
642	[nano_mp_deviation_cat_5]<br>Show the field ONLY if:<br>[nano_mp_deviation] = "f"	Choose category of deviation	radio, Required<br>a: CRFs have incomplete information for test results<br>b: Date / time not entered for preparation of dose<br>c: Date / time not entered for administration of dose<br>d: Other
643	[nano_mp_text_7]<br>Show the field ONLY if:<br>[nano_mp_deviation_cat_5] = "a"	Corrective action : follow up with patient / medical records / lab where applicable to complete prior to patient's last visit	descriptive
644	[nano_mp_text_8]<br>Show the field ONLY if:<br>[nano_mp_deviation_cat_5] = "b" OR [nano_mp_deviation_cat_5] = "c"	Corrective action : monitor to raise with site staff and ensure completed	descriptive
645	[nano_mp_specify]<br>Show the field ONLY if:<br>[nano_mp_deviation] = "g" OR [nano_mp_deviation_cat] = "b" OR [nano_mp_deviation_cat_2] = "e" OR [nano_mp_deviation_cat_3] = "b" OR [nano_mp_deviation_cat_8] = "b" OR [nano_mp_deviation_cat_4] = "g" OR [nano_mp_deviation_cat_5] = "d" OR [nano_mp_deviation] = "i"	Specify	notes, Required
646	[nano_mp_description]	Describe the protocol deviation	notes, Required
647	[nano_mp_reason]	Please explain the reason for the deviation	notes, Required

648	[nano_mp_result_ae]	Did the deviation result in adverse event ?	yesno, Required<br>1 Yes<br>0 No
649	[nano_mp_continue_study]	Did the participant continue the study ?	yesno, Required<br>1 Yes<br>0 No
650	[nano_mp_avoid_recurrence]	Describe the steps taken to avoid recurrence of the deviation	notes, Required
651	[nano_mp_pi_name]	Section Header: *Investigator signature*<br>Name of investigator	text, Required
652	[nano_mp_pi_date]	Date	text (datetime_dmy), Required
653	[nano_mp_pi_sign]	Signature of investigator	file (signature), Required
654	[minor_protocol_deviation_form_complete]	Section Header: *Form Status*<br>Complete?	dropdown<br>0 Incomplete<br>1 Unverified<br>2 Complete
